# The Effectiveness of Artificial Intelligence in Undergraduate Health Professions Education: Systematic Review and Meta-Analysis of Randomized Controlled Trials

**DOI:** 10.2196/88933

**Published:** 2026-05-05

**Authors:** Nai Ming Lai, Yin Sear Lim, Min Thein Win, Prabal Bhargava, Paraidathathu Thomas, Qi Chwen Ong

**Affiliations:** 1 School of Medicine, Faculty of Health and Medical Sciences, Taylor's University, Subang Jaya, Selangor, Malaysia; 2 Faculty of Health and Medical Sciences, Taylor's University, Subang Jaya, Selangor, Malaysia; 3 Division of Cardiovascular Medicine, Radcliffe Department of Medicine, John Radcliffe Hospital, University of Oxford, Level 6, West Wing, John Radcliffe Hospital, Headington, Oxford, England, OX3 9DU, United Kingdom, 44 01865234657

**Keywords:** health professions education, undergraduate, artificial intelligence, meta-analysis, educational resource, large languge model

## Abstract

**Background:**

Health professions education faces increasing challenges from rising health care complexity, pedagogical shifts, and constrained curricular space, and rapidly expanding knowledge and technological advances. While artificial intelligence (AI) shows promise for transforming health professions education, evidence of its effectiveness remains unclear.

**Objective:**

This study synthesized evidence from randomized controlled trials (RCTs) on the effectiveness of AI in undergraduate health professions education.

**Methods:**

We included RCTs, randomized crossover trials, and cluster RCTs comparing AI against standard educational interventions at the undergraduate level. We excluded quasi-experimental studies and those without clear AI components. We searched PubMed, Cochrane, Embase, Educational Resources Information Center, and Web of Science up to January 26, 2026. Outcomes were categorized according by Kirkpatrick levels; risk of bias was assessed using the Risk Of Bias Instrument for Use in Systematic Reviews for Randomised Controlled Trials tool; random-effects meta-analysis was conducted in RevMan (Cochrane); and certainty of evidence was rated using the Grading of Recommendations, Assessment, Development, and Evaluation approach. AI interventions were subcategorized by technology type and educational functions, yielding 13 subcategories.

**Results:**

Of 39,783 records identified, 66 RCTs (N=4911 participants; 2020-2026) were included. Subcategorized analyses across 7 outcome domains yielded 48 comparisons. Most studies had high risk of bias, mainly due to poor allocation concealment and blinding, and certainty of evidence ranged from low to very low. Large language model (LLM)–based personalized learning aids comprised the largest evidence base and showed positive effects for satisfaction (standardized mean difference [SMD] 0.93, 95% CI 0.40-1.46; 7 studies; 430 participants; *I*²=74%), confidence (SMD 0.91, 95% CI 0.54-1.29; 7 studies; 609 participants; *I*²=64%), and theoretical knowledge (SMD 0.53, 95% CI 0.13-0.94; 12 studies; 955 participants; *I*²=86%), all with very low certainty. Other AI subtypes, including LLM content generators, natural language processing (NLP) chatbots, and non-LLM adaptive learning platforms, showed generally favorable point estimates but substantial heterogeneity and wide CIs, often included no effect. Prediction intervals frequently crossed the null, indicating uncertainty across educational setting. No studies assessed Kirkpatrick levels 3 or 4.

**Conclusions:**

This review synthesized RCT evidence on AI in undergraduate health professions education by technology type and function, incorporating evidence certainty. Despite the large number of included studies, evidence remains insufficient to inform educational practice. Some AI interventions may improve some learning outcomes, but effects are inconsistent and not reliably reproducible. High risk of bias, heterogeneity, imprecision, and absence of higher-level outcomes limit conclusions. AI applications should therefore be used cautiously and on a trial basis.

## Introduction

### Background

Health professions education is rapidly expanding, with an estimated growth rate of 13.3% per year in the global health education market from 2024, reaching US $264 billion by 2030, according to a market analysis report [1]. This growth reflects rising health care complexity, expanding knowledge bases, increasing pedagogical demands, evolving learner expectations, and technological capabilities [2,3]. Traditional health professions education approaches struggle to keep pace with these changes because of challenges in incorporating innovations within the constraints of curricular space [4] and resistance from educators and stakeholders [5]. Educators face substantial pressure to deliver comprehensive curricular content while ensuring understanding and practical competency in a personalized, student-directed learning paradigm [6]. The challenge is compounded by the lack of strong evidence linking the adoption of any educational approach to consistent improvements in a demonstrated chain of relevant outcomes, from learning perception and knowledge to real-life practice and eventual health outcomes [7,8]. The COVID-19 pandemic accelerated the adoption of digital technologies in health professions education but also highlighted their limitations [9]. Artificial intelligence (AI), which has been applied in health professions education for over 2 decades [10], is now seen as a potentially powerful option to address these challenges given its recent rapid advancements.

A widely accepted definition of AI is the “science of making machines do things that would require intelligence if done by humans” [11]. AI in its many forms, including machine learning (ML), deep learning (DL), and artificial neural networks, has been used to analyze data patterns and make predictions with efficiency and consistency unmatched by humans [12]. Generative AI, a form of DL, has rapidly gained traction across a wide range of fields, including education, following the introduction of ChatGPT (OpenAI), a large language model (LLM), in November 2022 [13].

In health professions education, AI has been used in teaching-learning and assessment for over 2 decades [10]. AI-powered systems for teaching-learning include intelligent tutoring systems [14], adaptive learning platforms [15], and surgical training simulators such as the virtual operative assistant [16]. In assessment, AI has been used to automate student scoring and predict future performance in health disciplines [17]. To this end, LLMs have emerged as particularly influential, supporting clinical learning note compilation and summarization, virtual patient simulation, and personalized mentoring while facilitating self-directed learning and writing assistance [18]. While some studies have reported impressive performance of LLMs in undergraduate and postgraduate medical examinations [19], others have shown considerable gaps between LLMs and human experts in clinical diagnostic abilities [20]. The findings raise concerns about the appropriate use and limitations of LLMs in contemporary medical education [21].

### Rationale

Despite extensive AI use in health professions education, fundamental questions about effectiveness remain unclear. While one recent meta-analysis examined early studies on the use of generative AI in medical education [22], no prior review has comprehensively synthesized randomized controlled trial (RCT) evidence across all forms of AI, including ML, DL, and generative AI, specifically in undergraduate health professions education, which differs from postgraduate training in pedagogical approaches [23]. Published reviews on AI in health professions education are mostly scoping or narrative reviews that are useful in providing guidance on the extent, facilitators, and barriers of AI. However, they do not provide quantitative effect estimates, which are essential for educators and researchers who seek to understand the magnitude of benefits or harm from AI applications, particularly for undergraduate education. The surge in AI publications following ChatGPT’s November 2022 introduction [13] has created a critical mass of RCT evidence that is rapidly expanding, enabling meaningful meta-analysis.

### Objectives

This systematic review and meta-analysis aimed to synthesize evidence on the effects of AI educational applications, compared with standard teaching methods, on learning outcomes among undergraduate health professions students.

## Methods

### Eligibility Criteria

We included RCTs, randomized crossover, and cluster-randomized trials that enrolled undergraduate students of health sciences, including medicine, pharmacy, nursing, and allied health. We accepted studies conducted in any health training setting, including clinical teaching that involved patient engagement, skills training, classroom learning in the form of problem-based, task-based, or case-based learning, written assignments, as well as any form of assessment across all periods of follow-up. We excluded studies that evaluated postgraduate or residency training to limit the scope of our review, given the sufficient number of studies published at the undergraduate level. We imposed no restrictions on publication year, language, or report format (abstract or full paper).

In terms of intervention, we accepted studies that explicitly reported the use of AI, which included ML, DL, or generative AI, chiefly LLMs, used for teaching and assessment purposes, either as the main or supplementary tool. We only included studies that evaluated robotics and virtual or augmented reality with a clear description of AI involvement, as these applications may or may not involve an operating AI component [24]. We classified AI interventions as generative AI if they were explicitly described as using LLMs or other generative architectures capable of producing novel text, images, or dialogue in response to user input (eg, ChatGPT, GPT-4–based tutors (OpenAI), and LLM-driven virtual patients). We classified all remaining AI interventions as nongenerative AI, including ML and DL applications that analyze or classify data without generating novel output (eg, imaging diagnostic aids, automated scoring systems, and rule-based chatbots). The comparison included conventional methods of teaching and assessment involving direct human face-to-face input, as well as the use of various teaching-learning technologies except AI.

The review outcomes included any form of learning gain, evaluated using various scales in accordance with the levels depicted in the Kirkpatrick Model of Evaluation, a framework developed by Kirkpatrick in 1959 [25] and that has since been widely used in evaluating all forms of training. The list of outcomes is detailed under the subsequent subheading of Data Items. We included studies that fulfilled the criteria in terms of population and intervention, regardless of whether the study reported relevant outcomes or produced suitable outcome data for our meta-analysis.

### Information Sources

We searched MEDLINE (PubMed), the Cochrane Central Register of Controlled Trials (which included records from PubMed, Embase, CINAHL, and trial registers, including the World Health Organization (WHO) International Trials Registry Platform and ClinicalTrials.gov), Embase, Web of Science (Ovid), and Educational Resources Information Center for published studies and trial register records up to January 26, 2026. We searched the reference lists of relevant reviews for additional studies but did not identify any. We did not search conferences or other online sources or contact authors or personnel for additional studies.

### Search Strategies

We report the search strategies following the PRISMA-S (Preferred Reporting Items for Systematic Reviews and Meta-Analyses–Search extension) guideline [26]. The search strategies were developed by an author (NML) who has extensive systematic review experience, and were reviewed by another author (QCO) with systematic review experience (refer to Part 2 in Multimedia Appendix 1 for search strategies). We searched without applying any preset search filter or any language and publication type restriction.

### Data Collection Process

Two authors (NML and YSL) independently screened titles and abstracts for shortlisting and evaluated shortlisted papers in full text to determine eligibility, after deduplication using EndNote (version 19; Clarivate). One author (NML) used Claude Sonnet (Anthropic) [27], an LLM application, to perform preliminary data extraction, including population, intervention, comparison, and outcomes, and verified accuracy against the full texts. We provided the LLM with structured prompts that included the overall context, aided by the background information (background and methodology of the current review) as well as a data extraction spreadsheet template with desired headings, assigning the LLM a role as an expert in systematic review methodology, and instructing the platform to read and extract stipulated data types in a step-by-step manner, pointing out the location of the data source to facilitate human verification (examples of prompts are available in Part 11 in Multimedia Appendix 1), and manually transcribed the verified data into an Excel (Microsoft) spreadsheet, while a second author (QCO) cross-checked the extracted data by NML against the full texts. We assessed multiple publications of the same study via the setting and characteristics as enumerated above and only selected the reference with the most complete description of the study as the primary reference. We resolved disagreements by discussion leading to a consensus, with referral to the third author (MTW) as required.

### Data Items

We extracted participant characteristics, including setting (field of health professions education, eg, medicine, nursing, and dentistry), specific subject or skills evaluated (eg, clinical skills, ophthalmology, radiograph interpretation, and denture mounting), the region where the study was conducted, intervention technologies (eg, LLM or non-LLM and specific technology used, including the main purpose of teaching and learning vs assessment), frequency and duration of the intervention, assessment and follow-up period, outcomes reported, and funding sources.

We categorized the outcomes, based on the Kirkpatrick Model of Training Evaluation into perceptionor satisfaction and self-efficacy or confidence (level 1), knowledge gain (theoretical knowledge, clinical skills, practical skills [including competence and task efficiency], and generic or personal skills) (level 2), behavioral change assessed from real-life practice (level 3), and improvement in outcomes at the level of the recipient, namely, patient health outcomes (level 4) [28].

### Assessment of Missing Data

If we found a significant dropout rate (>20%), we would judge the study at high risk of bias in terms of missing outcome data. We did not contact any author to request further information, as we did not consider any missing data to be critical for our meta-analysis.

### Study Risk-of-Bias Assessment

Two authors (NML and QCO) independently assessed risk of bias using the ROBUST-RCT (Risk Of Bias Instrument for Use in Systematic Reviews for Randomised Controlled Trials) tool, developed by Wang et al [29], with adaptation in the domain of blinding from “health providers” to “education providers.” The tool consisted of 6 core domains (random sequence generation, allocation concealment, blinding of participants, blinding of educational providers, blinding of outcome assessors, and outcome data not included in the analysis), with 8 optional domains that we did not assess. Detailed guidance, including suggested rules and a decision table of the tool, is freely available in the additional material of the paper by Wang et al [29].

Risk of bias was assessed separately for subjective and objective outcomes, particularly for items related to blinding of education providers. For subjective outcomes, we split blinding of providers (Item 4) into 2 subdomains. Item 4a (perception and satisfaction) was rated probably low risk, as anonymous self-reporting by adult learners is unlikely to be systematically influenced by unblinded providers in the absence of evidence of coercion or deliberate influence on ratings. Item 4b (confidence and self-perceived competence) was rated probably high risk by default, as unblinded providers can plausibly influence learners’ self-appraisals through differential feedback, encouragement, and validation beyond the educational content delivered; exceptions were made for programs that were largely self-directed or used highly standardized provider-learner interactions. For objective outcomes, Item 4 was similarly rated probably high risk by default, as differential input from unblinded providers can affect actual competence even when outcomes are objectively scored, with exceptions for standardized or self-directed programs.

In ROBUST-RCT, there is no explicit rule provided in assigning the overall risk-of-bias status. Consequently, we described the risk of bias per study per domain and evaluated the overall degree of concern regarding risk of bias in our certainty of evidence rating by judging the proportion of high-risk domains in the body of evidence, as detailed in Part 1 in Multimedia Appendix 1. For example, if the majority of the included studies for the outcome assessed had a single high-risk domain (with blinding as a whole considered as 1 domain for this purpose), we would consider the body of evidence to have serious concerns in terms of risk of bias, and downgrade the certainty of the evidence by 1 level due to study limitations. However, if the majority of the included studies had 2 or more high-risk domains, we would consider the body of evidence to have very serious concerns in terms of risk of bias and downgrade the certainty of the evidence by 2 levels due to study limitations.

### Effect Measures

For continuous outcomes, we used standardized mean difference (SMD) to pool the study results, as each included study reported its outcome data using different scales. We used Cohen benchmarks as a descriptive reference (SMD <0.2 small, 0.2‐0.5 small to medium, >0.5‐0.8 medium to large, and >0.8 large) [30], while recognizing that these thresholds were not developed for educational intervention contexts and should not be interpreted as definitive indices of educational importance [31]. Accordingly, we contextualized effect sizes using a default minimally important difference of SMD 0.5, consistent with the empirically derived half-standard-deviation rule [32], applying this threshold in our interpretation of synthesis results. For dichotomous outcomes, we reported the results using relative risk (RR).

### Synthesis Methods

Studies with suitable numerical outcome data, either reported in text, table or derivable from figures were eligible for synthesis; those presenting results only narratively were excluded. We derived missing SD by multiplying the SE with the square root of the sample size in the corresponding group. For studies that reported their results in median and IQR, we approximated the median as the mean and obtained an estimate of the SD by dividing the IQR by 1.35, as recommended by Hozo et al [33]. Examples of studies from which we derived outcome data are available in Part 1 in Multimedia Appendix 1. For multiarm studies, we selected the relevant AI and control arms. In studies with multiple control groups, we selected the group representing the current standard educational intervention. We ensured each study appeared only once per analysis to avoid counting participants multiple times. For randomized crossover studies, we planned to include outcome data from the first period only, before the crossover, following one of the approaches suggested in the Cochrane Handbook [34]. If first-period data were not available, we would accept the data as reported by the authors and conduct sensitivity analysis to assess the impact of these crossover studies on the pooled results.

We tabulated major components of the intervention and comparison in each study in the characteristics of included studies table (Part 5 in Multimedia Appendix 1). We performed separate comparisons for each subcategory of AI application and divided the outcomes according to the levels of the Kirkpatrick Model of Training Evaluation [25], with further division for different subdomains based on the characteristics of the outcomes assessed, for example, self-efficacy and attitude (level 1) and theoretical knowledge, clinical, and practical skills (level 2), following discussion among the review authors.

If studies reported multiple outcomes in the same domain, we exercised our judgment in selecting the most suitable outcomes for our meta-analysis following a discussion among the review authors. For example, we used a combined score in preference to scores for individual components, and if no combined scores were reported, we selected one component that was most relevant to the domain; and if there were multiple components of equal relevance, we manually derived the combined mean and SD following the formula recommended in Chapter 10 of the Cochrane Handbook [35].

We presented our meta-analysis results graphically using forest plots. However, because of the large number of meta-analyses performed, we presented the synthesis results numerically in Table 1 and kept all the analysis details, including the forest plots in MultimediaAppendices 1  2.

**Table 1.** The full analysis results for each comparison between artificial intelligence (AI) subcategories and controlto with prediction intervals where applicable and certainty of evidence.

Outcome and specific comparison	Studies (participants)	*I*²	Results (point estimate with 95% CI)	95% prediction interval	Certainty of evidence (GRADE^a^)
Kirkpatrick level 1: satisfaction or motivation					
LLM^b^ content generator vs control	5 (509)	96%	SMD^c^ 0.65 (−0.73 to 2.04)	−2.67 to 3.98	Very low
LLM personalized learning aid vs control	7 (430)	74%	SMD 0.93 (0.40 to 1.46)	−0.40 to 2.26	Very low
LLM virtual patient vs control	3 (127)	64%	SMD 0.69 (−0.83 to 2.21)	−1.86 to 3.24	Very low
LLM content generator + LLM virtual patient + LLM personalized learning aid vs control	1 (88)	N/A	SMD −0.02 (−0.44 to 0.40)	N/A^h^	Very low
LLM-integrated curriculum vs control	1 (96)	N/A	SMD 1.31 (0.87 to 1.76)	N/A	Very low
Non-LLM AI^e^-moderated adaptive learning platform vs control	2 (143)	0%	SMD 0.55 (−1.00 to 2.11)	−1.00 to 2.11	Very low
NLP^f^ rule-based chatbot vs control	2 (146)	74%	SMD 0.74 (−3.65 to 5.13)	−6.18 to 7.66	Very low
NLP rule-based chatbot + rule based virtual patient vs control	1 (61)	N/A	SMD 0.17 (−0.33 to 0.67)	N/A	Very low
NLP rule-based virtual patient vs control	1 (79)	N/A	SMD 0.75 (0.29 to 1.20)	N/A	Low
Kirkpatrick level 1: self-efficacy or confidence					
LLM personalized learning aid vs control	7 (609)	64%	SMD 0.91 (0.54 to 1.29)	0.05 to 1.77	Very low
LLM virtual patient vs control	2 (100)	91%	SMD 1.36 (−8.09 to 10.81)	−14.49 to 17.20	Very low
LLM-integrated curriculum vs control	1 (96)	N/A	SMD 1.23 (0.79 to 1.67)	N/A	Very low
Non-LLM AI procedure assistant vs control	1 (40)	N/A	SMD 0.00 (−0.62 to 0.62)	N/A	Very low
Non-LLM AI moderated adaptive learning platform vs control	1 (40)	N/A	SMD 2.45 (1.61 to 3.28)	N/A	Low
NLP rule-based chatbot vs control	2 (146)	43%	SMD 0.87 (−2.11 to 3.86)	−3.21 to 4.96	Very low
Non-LLM AI-VR^g^ virtual doctor vs control	1 (64)	N/A	SMD −0.68 (−1.18 to−0.17)	N/A	Very low
AI procedure assistant + adaptive learning platform vs control	1 (20)	N/A	SMD 0.55 (−0.34 to 1.45)	N/A	Very low
AI procedure assistant (non-LLM) vs control (proportion of participants confident in echocardiography view)	1 (43)	N/A	RR 1.26 (0.45 to 3.50)	N/A	Very low
Kirkpatrick level 2: theoretical knowledge score					
LLM content generator vs control	3 (359)	93%	SMD 0.99 (−1.04 to 3.01)	−2.96 to 4.93	Very low
LLM gamification tool vs control	1 (48)	N/A	SMD 0.79 (0.20 to 1.38)	N/A	Low
LLM personalized learning aid vs control	12 (955)	86%	SMD 0.53 (0.13 to 0.94)	−0.81 to 1.88	Very low
Non-LLM AI moderated adaptive learning platform vs control	1 (40)	N/A	SMD 0.68 (0.04 to 1.32)	N/A	Low
NLP rule-based chatbot vs control	3 (530)	97%	SMD 1.06 (−2.19 to 4.32)	−5.28 to 7.41	Very low
Non-LLM AI imaging diagnostic aid vs control	2 (69)	78%	SMD 1.26 (−6.23 to 8.74)	−10.73 to 13.24	Very low
Non-LLM AI-VR virtual doctor vs control	1 (64)	N/A	SMD 0.67 (0.16 to 1.17)	N/A	Very low
Proportion with grade A or B: non-LLM AI gamification tool vs control	1 (73)	N/A	RR 1.33 (1.01 to 1.74)	N/A	Very low
Kirkpatrick level 2: clinical skills					
LLM content generator vs control	2 (295)	97%	SMD 0.52 (−8.66 to 9.69)	−15.22 to 16.25	Very low
LLM personalized learning aid vs control	9 (609)	83%	SMD 0.49 (0.00 to 0.97)	−0.93 to 1.90	Very low
LLM virtual patient vs control	1 (56)	N/A	SMD 2.53 (1.82 to 3.25)	N/A	Low
LLM content generator + LLM virtual patient + LLM personalized learning aid vs control	1 (88)	N/A	SMD −0.11 (−0.53 to 0.31)	N/A	Very low
LLM virtual patient + LLM personalized learning aid vs control	3 (124)	95%	SMD 1.82 (−3.63 to 7.27)	−8.61 to 12.24	Very low
Non-LLM AI imaging diagnostic aid vs control	4 (176)	62%	SMD 0.43 (−0.41 to 1.27)	−1.13 to 1.99	Very low
Non-LLM AI moderated adaptive learning platform vs control	2 (139)	98%	SMD 0.59 (−19.98 to 21.17)	−34.78 to 35.97	Very low
NLP rule-based virtual patient vs control	1 (79)	N/A	SMD 2.03 (1.49 to 2.58)	N/A	Moderate
NLP rule-based chatbot + virtual patient vs control	1 (61)	N/A	SMD 0.24 (−0.26 to 0.74)	N/A	Very low
AI-VR virtual doctor vs control	1 (64)	N/A	SMD 0.21 (−0.28 to 0.71)	N/A	Very low
AI procedure assistant + AI-moderated adaptive learning platform vs control	1 (20)	N/A	SMD 0.80 (−0.12 to 1.72)	N/A	Very low
Kirkpatrick level 2: practical skills					
LLM personalized learning aid vs control	1 (187)	N/A	SMD 0.67 (0.37 to 0.96)	N/A	Low
Non-LLM AI procedure assistant vs control	6 (305)	92%	SMD 0.18 (−0.97 to 1.34)	−2.77 to 3.13	Low
Kirkpatrick level 2: task efficiency (time taken to perform tasks)					
LLM personalized learning aid vs control	2 (100)	47%	SMD −0.15 (−4.24 to 3.95)	−5.99 to 5.69	Very low
Non-LLM AI imaging diagnostic aid vs control	1 (40)	N/A	SMD 2.70 (1.82 to 3.58)	N/A	Very low
Non-LLM AI procedure assistant vs control	2 (52)	88%	SMD −1.26 (−14.65 to 12.12)	−23.49 to 20.96	Very low
Non-LLM AI procedure assistant + AI-moderated adaptive learning platform vs control	1 (20)	N/A	SMD −0.87 (−1.80 to 0.05)	N/A	Very low
Kirkpatrick level 2: generic or personal skills					
LLM personalized learning aid vs control	1 (101)	N/A	SMD 0.45 (0.05 to 0.84)	N/A	Low
LLM virtual patient vs control	1 (27)	N/A	SMD 0.00 (−1.06 to 1.06)	N/A	Very low
LLM-integrated curriculum vs control	1 (96)	N/A	SMD 0.60 (0.19 to 1.01)	N/A	Moderate
Non-LLM AI communication analysis vs control	1 (25)	N/A	SMD 1.85 (0.88 to 2.81)	N/A	Low
AI-VR virtual doctor vs control	1 (64)	N/A	SMD 0.31 (−0.18 to 0.81)	N/A	Low

a GRADE: Grading of Recommendations, Assessment, Development, and Evaluation.

b LLM: large language model.

c SMD: standardized mean difference.

d Not applicable.

e AI: artificial intelligence.

f NLP: natural language processing.

g AI-VR: artificial intelligence–virtual reality.

We performed random-effects meta-analysis via the inverse-variance method using Review Manager (RevMan) online software (Cochrane), with between-study variance estimated using the restricted maximum likelihood method. We presented all point estimates with their 95% CIs, generated using the Hartung-Knapp-Sidik-Jonkman method, as simulation studies suggest this approach provides more appropriate coverage probabilities, particularly when heterogeneity is substantial [36,37]. To identify and quantify heterogeneity, we visually inspected the forest plots and used the *I*^2^ statistic (detailed below in the “Assessment of Heterogeneity” subheading), with generation of a 95% prediction interval (PI) as an indication of the likely range of effects in individual studies [35].

### Assessment of Heterogeneity

We evaluated heterogeneity statistically and explored plausible explanations in terms of educational characteristics of the studies [38]. We used the *I*^2^ statistic with a cutoff of 50% to indicate substantial heterogeneity [38]. If substantial heterogeneity was found, we explored educational characteristics via subgroup analyses in terms of the field or program evaluated, the region where the study was conducted, the category of AI intervention delivered (divided broadly into LLM and non-LLM), the major purpose of intervention (teaching-learning or assessment), and the frequency of intervention (single or multiple sessions across a period of time). As part of heterogeneity exploration, we generated a 95% PI, which indicated the expected range of true effects in similar individual studies [39]. AI subcategories and outcome types were not part of the heterogeneity exploration, as we had already separated the analyses according to these subcategories, as well as grouped the outcomes according to Kirkpatrick levels.

### Sensitivity Analysis

We planned to conduct sensitivity analyses by restricting analyses to studies at low or probably low risk of bias for each domain where at least 5 studies were available in each risk stratum, consistent with Cochrane guidance [35]. However, sensitivity analysis was only feasible based on 2 risk-of-bias domains and for only 1 analysis, as detailed below in the Results section.

### Publication and Reporting Bias Assessment

We created funnel plots to screen for small-study effects, a possible reason for publication bias, for outcomes in which there were ≥10 studies. If a funnel plot showed significant asymmetry, we would downgrade the certainty of the evidence based on a strong suspicion of publication bias from small-study effects [40]. However, in this review, the substantial heterogeneity observed across outcomes rendered funnel plot interpretation unreliable, especially when asymmetry is present [41]. Nonetheless, the single funnel plot generated in our review did not show significant asymmetry. We did not specifically assess the risk of bias from missing results in a meta-analysis (ie, reporting biases), using recently introduced risk-of-bias tools, such as the RoB-ME tool developed by Page et al [42].

### Certainty of Evidence Rating

Two authors (NML and QCO) independently assessed the certainty of the evidence for 7 major outcomes for each comparison, namely, self-efficacy or confidence, attitude or satisfaction, theoretical knowledge, clinical skills, practical skills, task efficiency, and generic or personal skills using the Grading of Recommendations, Assessment, Development, and Evaluation approach [43]. We considered evidence from RCTs as high certainty to begin with, downgrading 1 level for serious (or 2 levels for very serious) limitations based upon 5 considerations: risk of bias, inconsistency across studies, indirectness of the evidence, imprecision of estimates, and publication bias (refer to Part 1 in Multimedia Appendix 1 for details). We used GRADEpro GDT (The GRADE Working Group) to create the summary of findings table (Part 3 in Multimedia Appendix 1).

### Ethical Considerations

This work is a systematic review and meta-analysis of previously published studies; therefore, no ethics approval or consent to participate was required. The review was registered in PROSPERO (CRD42021243832). We conducted the review following Cochrane methods [38] and reported according to the PRISMA (Preferred Reporting Items for Systematic Reviews and Meta-Analyses) 2020 guidelines [44] (Checklist 1), with particular reference to the PRISMA 2020 expanded checklist [45] in the structure of our paper. Additional details on our methods, including changes from the protocol, are described in Part 1 in Multimedia Appendix 1.

## Results

### Study Selection

**Figure 1.**
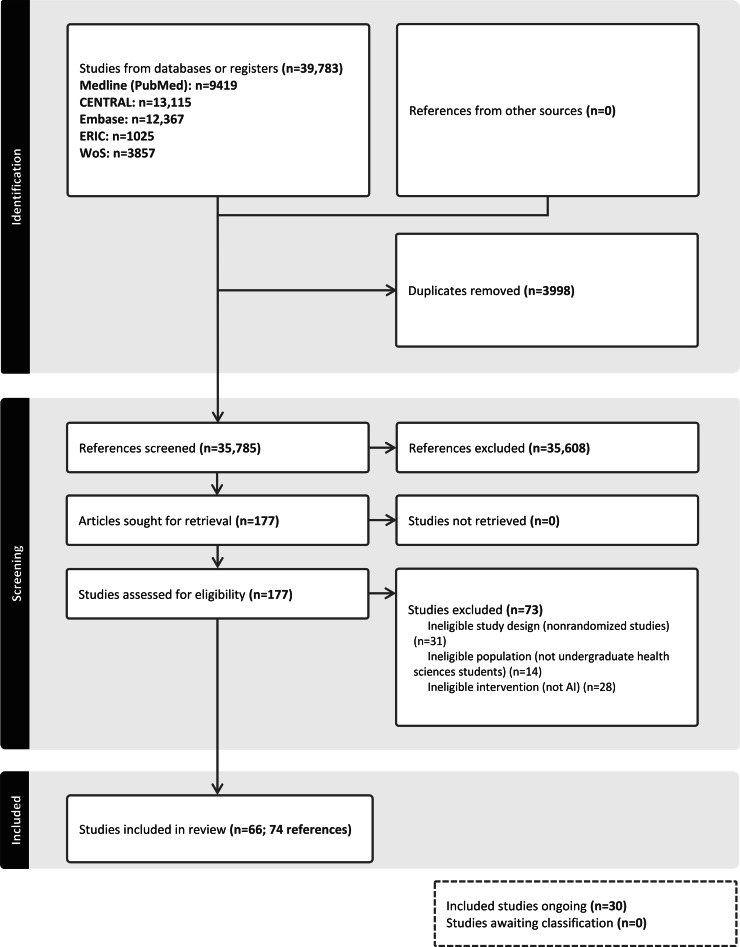
PRISMA (Preferred Reporting Items for Systematic Reviews and Meta-Analyses) flow diagram depicting the process from initial screening to final inclusion. AI: artificial intelligence.

The initial search identified 39,783 records, with 35,785 records remaining after deduplication. The vast majority of the initial records (n=35,608) were clearly nonrelevant from an inspection of the title or abstract, and these were rejected outright. We shortlisted 177 papers that appeared relevant and assessed their full texts in depth, excluding an additional 73 papers due to mismatch in study design (n=31, 42.5% papers), population (not undergraduate students; n=14, 19.2% papers), or intervention (not AI; n=28, 38.4% papers) (refer to Figure 1 for reasons for exclusion and Part 4 in Multimedia Appendix 1 for the full list). We also identified 30 relevant ongoing studies. Finally, we included 74 records that described 66 studies in our analysis, as 3 studies had multiple records, including 3 with 2 records [46-48], 1 with 3 records [49], and 1 with 4 records [50]. The flow diagram of the studies from the initial search to meta-analysis is shown in Figure 1.

### Study Characteristics

The characteristics of included studies are summarized as follows (refer to Part 5 in Multimedia Appendix 1 for a detailed tabulation).

### Design and Setting

Out of 66 included studies (N=4911 participants) published between 2020 and 2026 [46-49,51-116], 58 (87.9%) were parallel RCTs, 7 (10.6%) were randomized crossover trials, and 1 (1.5%) was a cluster-randomized trial. Most studies were conducted in China (n=14, 21.2%), followed by Turkey (n=11, 16.7%), the United States (n=5, 7.6%), Canada (n=4, 6.1%), Germany and Taiwan (n=3 each, 4.5%), and Italy, South Korea, Hong Kong, Morocco, Singapore and the United Kingdom (n=2 each, 3%), with a single study conducted in each of Colombia, Denmark, France, India, Ireland, Israel, Japan, New Zealand, Norway, Pakistan, Spain, and Thailand, with 2 multicenter studies involving different countries [66,84]. The number of participants per study ranged from 16 [47,79] to 426 [97], with 63 [82,102] as the median sample size.

### Population and Educational Field

Over half of the included studies enrolled students from medicine (n=35, 53%), followed by nursing (n=15, 22.7%), dentistry (n=9, 13.6%), physiotherapy, pharmacy, and health sciences (n=2 each, 3%), and optometry (n=1, 1.5%). Within each program, there was a wide range of disciplines assessed. In medicine, these included preclinical sciences (physiology, anatomy, pathology, pharmacology, biochemistry, immunology, microbiology, and histology), as well as clinical specialties such as internal medicine, cardiorespiratory medicine, gastroenterology, pulmonology, obstetrics and gynecology, ophthalmology, urology, radiology, neurosurgery, surgery, and emergency and critical care medicine; generic skills such as clinical reasoning, evidence-based medicine, and clinical decision-making were also represented. In nursing, subfields included surgical nursing, critical care nursing, sepsis care, clinical skills, communication skills, medical terminology, and patient education. In dentistry, areas covered included dental radiology, endodontics, restorative and operative skills, and prosthodontics. Physiotherapy studies addressed clinical reasoning and chronic low back pain rehabilitation, while health sciences studies covered infection control and chronic disease management. Specific practical skills assessed across fields included simulated brain tumor resection, cardiac ultrasonography, and dental access cavity preparation.

### Intervention

The studies used AI mainly as teaching-learning enhancement tools (n=54, 81.8%), as an assessment tool only (n=1, 1.5%) [104], or both (11, 16.7%). LLM applications were used in 38 (57.6%) studies, primarily featuring ChatGPT (versions 3.5 and 4.0; Open AI), alongside Gemini (Google), Copilot (Microsoft), Perplexity (Perplexity AI, Inc), Claude (Anthropic), and specialized chatbots. The remaining 28 (42.4%) studies used non-LLM AI systems composed of specific algorithms such as convolutional neural networks, linear support vector machines, NLP rule-based architectures, AI-powered virtual doctor or diagnostic systems, or intelligent monitoring and assessment systems, without any explicit use of LLM.

The AI applications were further categorized according to their predominant functions as described in the study. Among LLM studies, the applications included personalized learning aids (n=20), clinical content generators (n=6), virtual patients (n=6), combined virtual patient and personalized learning aid (n=3), a multifunction LLM combining content generation, virtual patient simulation, and personalized feedback (n=1), a gamification tool (n=1), and 1 study described as a general “LLM-integrated curriculum.”

Among non-LLM studies, applications included AI procedure assistant (n=7), AI imaging diagnostic aid (n=7), NLP rule-based chatbot (n=4), and AI-moderated adaptive learning platform (n=4), as well as an artificial intelligence–virtual reality (AI-VR) virtual doctor, AI communication analysis system, AI gamification tool, rule-based virtual patient, a combined NLP rule-based chatbot and virtual patient, and a combined procedure assistant with adaptive learning platform (n=1 each). These applications aimed to assist in achieving competencies from foundational knowledge to complex clinical decision-making. A working description of each AI subcategory is available in Table 2. This categorization recognizes potential overlap between subcategories, as some studies incorporate multiple AI functionalities or serve overlapping educational purposes.

**Table 2.** Working description of artificial intelligence (AI) subcategories for the purpose of classification in this review.

AI^a^ subcategory	Description
LLM^b^-based interventions	
LLM content generator	LLM-based applications generating educational materials (clinical vignettes, questions, explanatory text, case presentations, and learning guides).
LLM gamification tool	LLM-powered applications incorporating game-based elements (escape rooms and interactive challenges) to enhance learning engagement.
LLM personalized learning aid	LLM-based conversational tutors providing on-demand responses, explanations, postsimulation debriefing, and individualized feedback.
LLM virtual patient	LLM applications simulating patient encounters for clinical history-taking and communication skills practice.
LLM-integrated curriculum	Structured program with LLMs systematically embedded throughout curriculum delivery without specifying any component in learning.
Non-LLM AI interventions	
AI imaging diagnostic aid	AI systems using CNNs^c^ or machine learning to analyze medical images for diagnostic support, lesion detection, or educational annotation.
AI gamification tool	AI-powered applications incorporating game design elements using specialized algorithms for interactive skill-based learning.
AI procedure assistant	AI systems providing real-time guidance, quality assessment, or feedback during practical skills training using machine learning classifiers or computer vision.
AI-moderated adaptive learning platform	Intelligent tutoring systems using algorithms (Deep Q-Networks and sentiment analysis) to personalize learning pathways and optimize content delivery.
AI-VR^d^ virtual doctor	AI-powered virtual physician characters in VR simulations generating dynamic clinical responses through AI algorithms.
Natural language processing (NLP) rule-based chatbot	NLP chatbots using structured decision trees and preprogrammed dialogue patterns to deliver content and provide feedback.
Rule-based virtual patient	Virtual patient systems using decision tree algorithms to simulate patient responses through predetermined pathways.
AI communication analysis	AI systems using computer vision or behavioral analysis to assess and provide feedback on learner communication skills.

a AI: artificial intelligence.

b LLM: large language model.

c CNN: convolutional neural network.

d VR: virtual reality.

Study durations varied considerably, from single sessions of under 3 hours (n=24, 36.4%), short-term from 1 day to 3 weeks (n=19, 28.8%), medium-term from 1 to 2 months (n=13, 19.7%), and long-term from 2 to 6 months (n=5, 7.6%), with 5 studies not specifying duration.

### Comparison

Over half of the studies (n=39, 59.1%) reported the control group as having received standard methods of learning as implemented in the existing curriculum, including student presentations, lectures, web-based tools, standard assessment procedures with existing technologies led by human tutors, and human-based simulation methods such as standardized patients, peer role-play, and real patient-based training. In 18 (27.3%) studies, authors reported that participants in the control group received the same educational content without AI tools. Four studies [53,72,79,100] included 2 control groups that used different sources of evidence: 1 from standard institutional resources and the other from external resources via online searches. In these studies, we selected the group that used standard institutional resources as the control group for our analysis.

### Outcome Assessment

According to the Kirkpatrick Model of Evaluation [28], all studies measured outcomes either in level 1 (reaction) or level 2 (knowledge), and none reported outcomes in level 3 (real-life practice) or level 4 (health outcomes). The outcomes reported are summarized as follows:

- Level 1: 36 (54.5%) studies reported attitude, perception, or satisfaction, and 21 (31.8%) studies reported self-efficacy or confidence.
- Level 2: 26 (39.4%) studies reported theoretical knowledge, 30 (45.5%) studies reported clinical skills, 8 (12.1%) studies reported practical skills, 6 (9.1%) studies reported task efficiency, and 5 (7.6%) studies reported generic or personal skills.

Most outcomes were measured immediately after the intervention. Five studies [56,62,67,86,90] in addition reported outcomes at intermediate time points, ranging from 1 week to 3 months after the intervention.

### Funding

Nine (13.6%) studies received funding from government-linked agencies, including national research councils and government ministries. Eighteen (27.2%) studies were funded by universities, hospitals, or dedicated research institutes. Three studies received contributions from commercial companies: 2 received in-kind contributions in the form of AI platform or device access [55,82], and 1 received direct financial sponsorship from a commercial AI company [98]. Seventeen (25.8%) studies declared no funding, while 19 (28.8%) did not provide any funding statement.

### Risk of Bias in Studies

The full risk-of-bias assessment is shown in Part 6 in Multimedia Appendix 1. There are significant methodological limitations across the included studies, particularly in allocation concealment and blinding. Random sequence generation was adequate in 44 (66.7%) studies, but allocation concealment was adequate in only 15 (22.7%) studies. Nonblinding of participants resulted in high or probably high risk of bias from differential participant expectations in 55 (83.3%) studies.

Blinding of education providers was similarly not achieved in all but 4 studies. The risk implications, however, differed by outcome type. For participants’ perception or satisfaction (assessed in 47 studies), nonblinding of providers was judged probably low risk in 40 (85.1%) studies, on the basis that anonymous self-reported evaluations of a teaching program by adult learners were unlikely to be materially influenced by unblinded providers. For self-efficacy or confidence (assessed in 24 studies), the majority (n=20, 83.3%) were judged probably high risk, as provider awareness of group allocation could plausibly influence student confidence through differential feedback, encouragement, and validation beyond the educational content itself; this concern did not apply to the 4 remaining studies, which used largely self-directed learning formats [47,68,70,80]. For objective outcomes, 40 (66.7%) studies were judged probably high risk and 20 (33.3%) probably low risk, the latter reflecting programs that were largely self-directed or sufficiently standardized to limit meaningful provider influence.

While none of the assessors for subjective, self-reported outcomes were blinded, blinding of outcome assessors was achieved in 29 out of 60 (48.3%) studies that evaluated objective outcomes, but almost all studies were judged to have low or probably low risk of bias because of clear documentation of objective assessment criteria, with only 2 studies [110,113] judged as probably high risk due to a lack of description of the scoring criteria despite reporting numerical checklists. In terms of missing outcome data, most studies (n=59, 89.4%) demonstrated low risk of bias.

### Results of Individual Studies

Overall, 60 studies (90.9%; n=4506 participants) contributed suitable data for meta-analysis. The large number of AI subcategories resulted in numerous comparisons, most comprising few studies with small sample sizes. The results of individual studies are presented in the forest plots in Multimedia Appendix 2. None of the 7 randomized crossover trials provided separate data for the first period, and so we included data as reported in the paper where applicable.

### Results of Synthesis

A full display of the synthesis results is shown numerically in Table 1 and graphically in Multimedia Appendix 2. Substantial heterogeneity was present across almost all pooled estimates and was not adequately explained by subgroup analyses based on field and region of study, LLM vs non-LLM, teaching-learning vs assessment, and single vs multiple sessions (Multimedia Appendix 2). Consequently, the certainty of evidence was rated low or very low for most comparisons (Table 1). Ninety-five percent PIs, reported for all comparisons with 2 or more studies, were wide throughout and almost universally included the null value, indicating highly uncertain effects of AI interventions in any new study setting.

The results are summarized below, organized by Kirkpatrick level.

### Kirkpatrick Level 1: Participant Perception or Satisfaction

Twenty-three (1679 participants) studies assessed participant perception or satisfaction across 9 AI subtype comparisons (Table 1).

**Figure 2.**
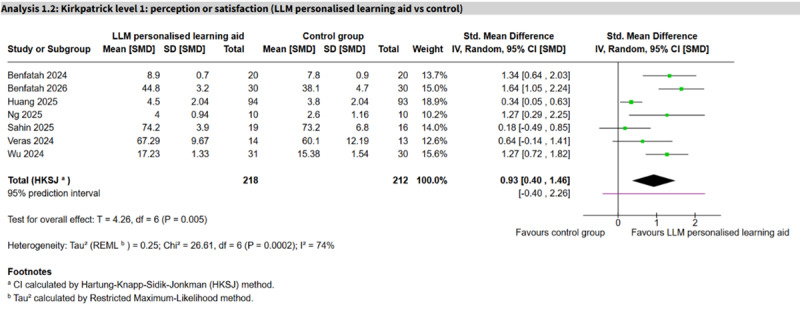
Forest plot for perception or satisfaction (large language model [LLM] personalized learning aid vs control) [48,70,80,84,113,115,116].

**Figure 3.**
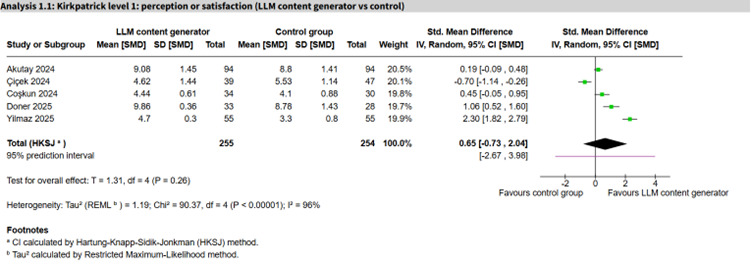
Forest plot for perception or satisfaction (large language model [LLM] content generator vs control) [49,51,61,62,100].

Among LLM-based comparisons, LLM personalized learning aids appeared to show an important positive effect (7 studies, n=430; SMD 0.93, 95% CI 0.40 to 1.46; *I*²=74%; 95% PI −0.40 to 2.26; Figure 2 [48,70,80,84,113,115,116]), whereas LLM content generators (5 studies, n=509; SMD 0.65, 95% CI −0.73 to 2.04; *I*²=96%; 95% PI −2.67 to 3.98; Figure 3 [49,51,61,62,100]) and LLM virtual patients (3 studies, n=127; SMD 0.69, 95% CI −0.83 to 2.21; *I*²=64%, 95% PI −1.86 to 3.24) showed no clear difference, with substantial heterogeneity and wide PIs spanning the null. The remaining comparisons each involved 1 or 2 studies and were imprecise (Tables1  2).

### Self-Efficacy or Confidence

Sixteen studies (1115 participants) assessed self-efficacy or confidence across 9 AI subtype comparisons, and 1 additional study reported a dichotomous outcome (Table 1).

**Figure 4.**
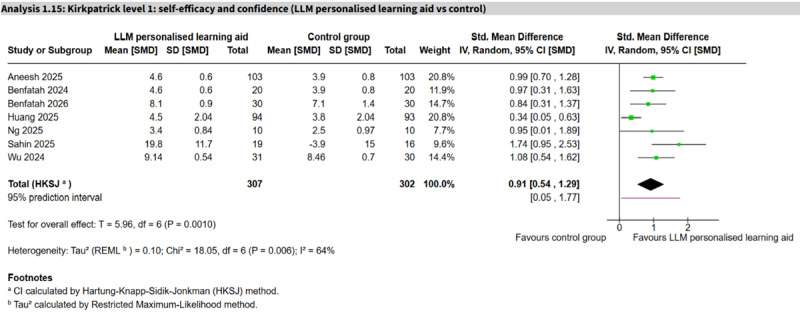
Forest plot for self-efficacy or confidence (large language model [LLM] personalized learning aid vs control) [53,70,80,84,113,115,116].

The largest comparison was LLM personalized learning aids, which appeared to show an important positive effect (7 studies, n=609; SMD 0.91, 95% CI 0.54 to 1.29; *I*²=64%, 95% PI 0.05 to 1.77; very low certainty; Figure 4 [53,70,80,84,113,115,116]). All other comparisons were based on 1 or 2 studies with no clear differences between groups. One notable single-study finding was a non-LLM AI-VR virtual doctor that showed reduced self-efficacy compared with control (SMD −0.68, 95% CI −1.18 to −0.17; very low certainty). A separate single study reported the proportion of participants confident in echocardiography views using a non-LLM AI procedure assistant, with no clear difference (RR 1.26, 95% CI 0.45 to 3.50; very low certainty).

### Kirkpatrick Level 2: Theoretical Knowledge

Twenty-four studies (2126 participants) assessed theoretical knowledge across 7 AI subtype comparisons, and 1 additional study reported a dichotomous outcome (Table 1).

**Figure 5.**
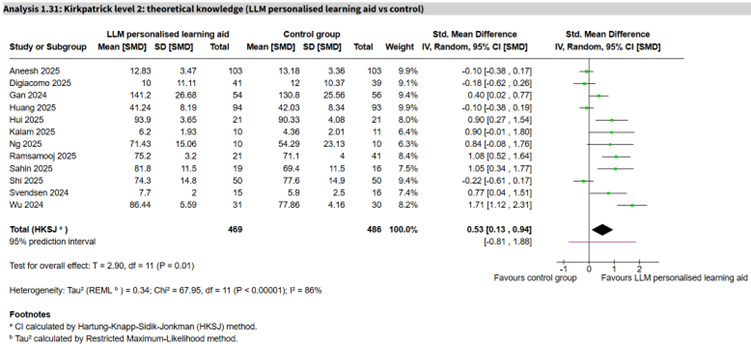
Forest plot for theoretical knowledge (large language model [LLM] personalized learning aid vs control) [53,63,67,70-72,80,82,84,86,90,113].

The most informative subtype comparison was LLM personalized learning aids, which appeared to show an important positive effect but with substantial heterogeneity (12 studies, n=955; SMD 0.53, 95% CI 0.13 to 0.94; *I*²=86%, 95% PI −0.81 to 1.88; very low certainty; Figure 5 [53,63,67,70-72,80,82,84,86,90,113]; publication bias not suggested from funnel plot, Part 8 in Multimedia Appendix 1). LLM content generators (3 studies, n=359; SMD 0.99, 95% CI −1.04 to 3.01; *I*²=80%, 95% PI −2.96 to 4.93) and NLP rule-based chatbots (3 studies, n=530; SMD 1.06, 95% CI −2.19 to 4.32; *I*²=65%, 95% PI −5.28 to 7.41) showed imprecise results with substantial heterogeneity and very wide PIs, all of very low certainty. Single-study comparisons of LLM gamification, non-LLM AI-moderated adaptive learning, and AI-VR virtual doctor each appeared to favor AI, while a non-LLM AI imaging diagnostic aid comparison (2 studies) was inconclusive (Table 1). One small study reported the proportion achieving grade A or B with a non-LLM AI gamification tool (RR 1.33, 95% CI 1.01 to 1.74; very low certainty).

### Clinical Skills

**Figure 6.**
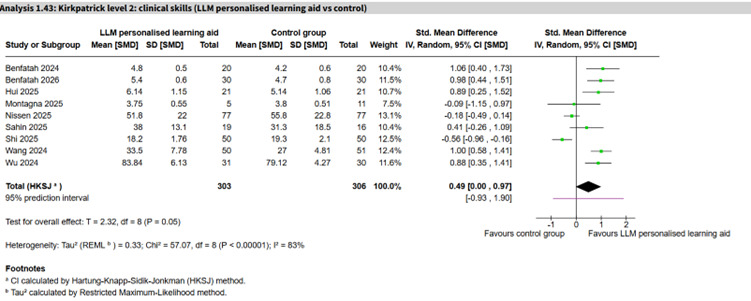
Forest plot for clinical skills (large language model [LLM] personalized learning aid vs control) [71,81,84,86,91,113,115,116].

**Figure 7.**
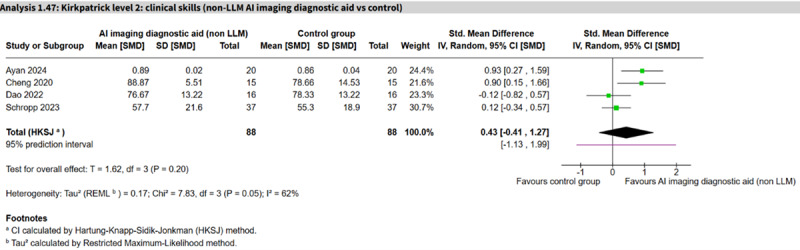
Forest plot for clinical skills (non–large language model [LLM] imaging diagnostic aid vs control) [56,60,85,99].

Twenty-six studies (n=1711) assessed clinical skills across 11 AI subtype comparisons (Table 1). The largest comparisons were LLM personalized learning aids (9 studies, n=609; SMD 0.49, 95% CI 0.00 to 0.97; *I*²=83%, 95% PI −0.93 to 1.90; very low certainty; Figure 6 [71,81,84,86,91,113,115,116]) and non-LLM imaging diagnostic aid (4 studies, n=176; SMD 0.43, 95% CI −0.41 to 1.27; *I*²=62%, 95% PI −1.13 to 1.99; very low certainty; Figure 7 [56,60,85,99]), which showed no clear differences between groups and substantial heterogeneity. A single study of a NLP rule-based virtual patient showed a large effect (SMD 2.03, 95% CI 1.49 to 2.58; moderate certainty), and a single LLM virtual patient study similarly showed a large effect (SMD 2.53, 95% CI 1.82 to 3.25; low certainty). The rest of the comparisons were inconclusive.

### Practical Skills

**Figure 8.**
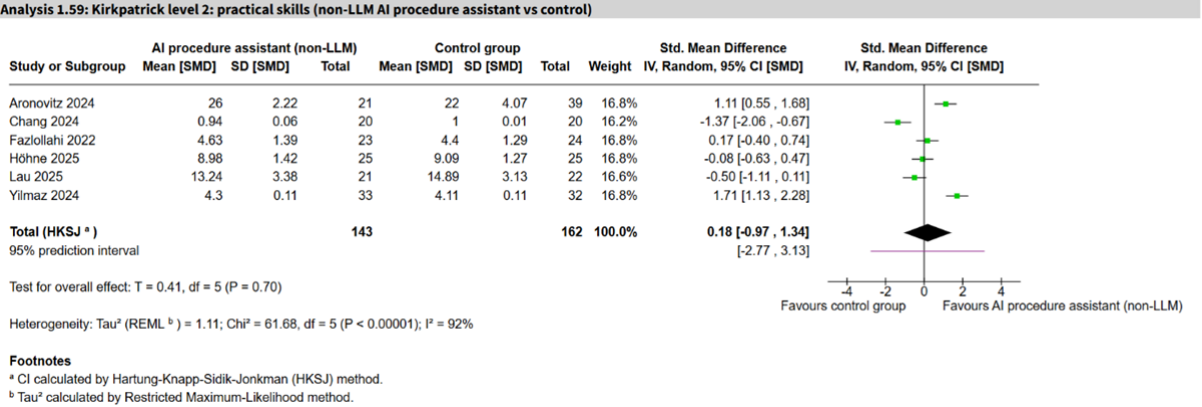
Forest plot for practical skills (non–large language model [LLM] artificial intelligence [AI] procedure assistant vs control) [49,50,55,58,69,105].

Seven studies (492 participants) assessed practical skills (Table 1). One LLM personalized learning aid study (187 participants) appeared to show an important improvement (SMD 0.67, 95% CI 0.37 to 0.96; low certainty), whereas 6 studies of non-LLM AI procedure assistants (=305 participants) showed no clear difference but substantial heterogeneity (SMD 0.18, 95% CI −0.97 to 1.34; *I*²=92%, 95% PI −2.77 to 3.13; low certainty; Figure 8 [49,50,55,58,69,105]).

### Task Efficiency

Six studies (212 participants) measured task efficiency, defined as time taken to complete tasks, across 4 subtype comparisons (Table 1). A single study of a non-LLM AI imaging diagnostic aid showed markedly increased time required to complete task (SMD 2.70, 95% CI 1.82 to 3.58), whereas other comparisons showed no clear differences, all with very low certainty.

### Generic or Personal Skills

Five studies (313 participants) assessed generic or personal skills across 5 single-study comparisons (Table 1). A single LLM-integrated curriculum study appeared to show an important improvement (SMD 0.60, 95% CI 0.19-1.01; moderate certainty), as did a single non-LLM AI communication analysis study (SMD 1.85, 95% CI 0.88 to 2.81; low certainty). A single LLM personalized learning aid study showed a small improvement (SMD 0.45, 95% CI 0.05 to 0.84; low certainty). The remaining comparisons showed no clear differences.

### Investigation of Heterogeneity

A detailed report on the results of our heterogeneity exploration is available in Part 7 in Multimedia Appendix 1. In summary, there was a very high degree of heterogeneity across almost all outcomes (*I*²: 86%‐94%) with no consistent studies or group of studies identified as the chief contributors. Subgroup analyses (by field and region of study, LLM vs non-LLM, predominant function, and single vs multiple sessions), conducted for all outcomes with sufficient studies (Multimedia Appendix 2), revealed no consistent explanation of heterogeneity, as none of these factors consistently moderated the direction or magnitude of the pooled estimates.

### Sensitivity Analysis

Sensitivity analysis was only feasible for the domains of random sequence generation and allocation concealment, and for the analysis of theoretical knowledge (comparing LLM personalized learning aid against control), as this was the only analysis with at least 5 studies in more than 1 risk stratum. In this analysis, after removing 7 studies with high risk of bias in allocation concealment, the pooled estimate of 5 remaining studies changed from showing a significant improvement in knowledge to no significant difference between groups. This might indicate that low-risk studies tended to show more conservative estimates, or reflected reduced statistical power from the smaller number of remaining studies. We could not perform sensitivity analysis to exclude crossover trials because in none of the analyses where these trials were included, there were sufficient studies in each stratum to enable a meaningful analysis.

### Certainty of Evidence

Certainty of evidence was low to very low for most outcomes, due mainly to study limitations (high risk of bias across multiple domains), inconsistency (high degree of heterogeneity with wide 95% PIs), and imprecision (wide 95% CIs crossing multiple effect thresholds; for details refer to Part 3 in Multimedia Appendix 1).

## Discussion

### Principal Findings

This review offers the first comprehensive RCT synthesis of AI in undergraduate health professions education, with granular subcategorization by technology type and educational function across diverse global settings. However, the wide diversity of AI subcategories produced numerous specific analyses, most containing few studies with imprecise, very-low-certainty estimates, leaving the body of evidence far from sufficient to inform educational practice, despite the overall large number of included RCTs.

Based predominantly on very low-certainty evidence, the findings suggest that certain AI intervention types may improve specific educational outcomes, but the pattern of benefit is inconsistent. LLM-based personalized learning aids, with comparatively the largest volume of evidence across outcomes, show a possible positive effect in perception, confidence, and knowledge, but the PIs of these outcomes were wide and mostly crossing the null, indicating substantial uncertainty about the likely effect in future educational contexts.

Across all other AI subcategories, point estimates were generally positive but CIs and PIs were wide and typically crossed the null, precluding firm conclusions. No studies assessed Kirkpatrick Level 3 (real-world practice change) or Level 4 (health outcomes), where evidence would be most compelling for educational policy.

The subcategorized analyses also clarify which comparisons remain informationally sparse. Several AI subtypes, particularly multicomponent interventions and novel tool combinations, contributed only single-study estimates, highlighting where future research is most needed.

### Comparison With Prior Work

Our findings build on and extend previous syntheses in this field. Before this work, approximately 50 reviews examined AI in health education (Part 9 in Multimedia Appendix 1), but the majority are scoping or narrative reviews, and most express cautious optimism without quantitative evidence from RCTs. Two meta-analyses evaluating LLMs in national licensing examinations reported a wide performance range and concluded that further model development and evaluation are needed before LLMs can be recommended as primary teaching tools [117,118]. A scoping review of AI across medical education similarly concluded that AI applications show promise but require rigorous evaluation [10].

Most directly comparable to our work is a recent meta-analysis of 11 RCTs (786 participants) examining generative AI in medical education, which reported no clear difference in overall knowledge acquisition but improved practical skills and satisfaction [22]. Our review extends this in several important respects: a more recent search (January 2026 vs January 2025), broader scope (all AI applications and health disciplines, not only generative AI in medicine), larger evidence base (60 RCTs, 4506 participants in meta-analysis), systematic subcategorization of AI intervention types, outcome classification using Kirkpatrick framework, risk of bias assessment using ROBUST-RCT, and, crucially, Grading of Recommendations, Assessment, Development, and Evaluation certainty-of-evidence ratings, absent from all previously published reviews of this topic. Without certainty ratings, positive point estimates risk being interpreted as practice-ready evidence; yet, in almost every comparison, certainty is low or very low, and PIs indicate that effects may not reliably reproduce across settings.

### Limitations

We acknowledge several limitations of the evidence gathered and the review process. In terms of the evidence gathered, despite comprehensive and up-to-date searches, several limitations require acknowledgment. First, even after subcategorization, substantial residual heterogeneity remained within several comparisons (*I*²: 64%‐98%), which was not adequately explained by the study characteristics reported. This heterogeneity likely reflects the inherently context-dependent nature of educational interventions, where learner characteristics, instructor expertise, institutional culture, implementation fidelity, and assessment approaches interact in ways that are rarely reported in sufficient detail [119,120]. Rather than invalidating the findings, this suggests the effectiveness of AI tools may be context-sensitive, shaped by local factors rarely reported in sufficient detail. The consistent direction of effect (most studies favoring AI) provides a preliminary signal of benefit, but the magnitude of any effect in a given setting remains uncertain.

Second, granular subcategorization, while educationally more informative, resulted in many comparisons based on only 1 or 2 studies, which are too imprecise to provide actionable guidance for practice.

Next, we included 7 crossover trials [47,48,53,66,74,80,81], which may have additional concerns in their study design that we have not adequately addressed, because the newly established ROBUST-RCT tool has not been expanded to cover crossover design [29]. Additionally, none of these studies provided data separately for the first period, as desired. However, given that most included studies were already rated at high risk of bias for allocation concealment and blinding and the evidence certainty was very low for most outcomes, this limitation is unlikely to substantially alter the overall certainty assessments, but cannot be dismissed.

In terms of the review process, despite screening nearly 40,000 records and shortlisting 177 potentially eligible records, we may have missed studies evaluating AI-enabled tools (such as robotic or VR-based systems) not described as AI interventions in their reports. Our risk of bias assessment and decisions on which outcomes to include for meta-analysis, although transparent, involved some subjective judgment and might have been influenced by personal biases. Pooling continuous outcomes using SMD across heterogeneous instruments, particularly for subjective outcomes such as satisfaction and self-efficacy, is another limitation of this review; however, we incorporated an SMD of 0.5 as a default minimally important difference and PIs to improve interpretability in educational practice. Finally, our subcategorization of specific AI applications was made for the purpose of the current review, and our decision to assign one study to one subcategory or another, although largely reliant on the information provided by the study, involved subjective judgment. However, we hope this taxonomy provides a working framework for future syntheses as the evidence base grows.

### Conclusions and Implications

Early evidence from RCTs suggests that the effects of AI applications are neither uniform across AI types nor consistent across settings. Although certain AI intervention types used in undergraduate health professions education appeared to improve some educational outcomes, particularly satisfaction and theoretical knowledge with LLM-based personalized learning aids, the findings are highly heterogeneous with low- to very low–certainty evidence and are far from sufficient in informing educational practice. No studies have yet assessed real-world practice change or health-related outcomes, which are the levels of evidence most relevant to educational policy. We recommend that AI applications in undergraduate health education continue to be used on a trial basis. Future RCTs should be adequately powered and use robust allocation concealment, such as central randomization or online systems with real-time allocation, with transparent documentation. Standardized outcome measures should be used to minimize performance and detection bias, with clear reporting of implementation details to support meaningful subgroup and heterogeneity analyses. Studies should also begin to assess longer-term Kirkpatrick Level 3 and 4 outcomes, which reflect real-world behavior change and patient health outcomes, as these would particularly strengthen the evidence base for educational guideline adoption.

## Supplementary material

10.2196/88933 Multimedia Appendix 1 Compilation of parts 1-11.

10.2196/88933 Multimedia Appendix 2 Full analyses with forest plots.

10.2196/88933 Checklist 1 PRISMA 2020 checklist.

10.2196/88933 Checklist PRISMA-S checklist.

## Abbreviations

AI: artificial intelligence
AI-VR: artificial intelligence–virtual reality
DL: deep learning
LLM: large language model
ML: machine learning
PI: prediction interval
PRISMA: Preferred Reporting Items for Systematic Reviews and Meta-Analyses
PRISMA-S: Preferred Reporting Items for Systematic Reviews and Meta-Analyses–Search extension
RCT: randomized controlled trial
ROBUST-RCT: Risk Of Bias Instrument for Use in Systematic Reviews for Randomised Controlled Trials
RR: relative risk
SMD: standardized mean difference
WHO: World Health Organization

## References

[1] (2024). Healthcare education market (2025-2033) size, share & trends analysis report by providers (continuing medical education providers, educational platforms, learning management systems, universities & academic centers), by application, by delivery mode, by end use, by region, and segment forecasts. https://www.grandviewresearch.com/industry-analysis/healthcare-education-market-report.

[2] Alotaibi N, Wilson CB, Traynor M (2025). Enhancing digital readiness and capability in healthcare: a systematic review of interventions, barriers, and facilitators. BMC Health Serv Res.

[3] Grainger R, Liu Q, Gladman T (2024). Learning technology in health professions education: realising an (un)imagined future. Med Educ.

[4] Slavin S, D’Eon MF (2021). Overcrowded curriculum is an impediment to change (Part A). Can Med Educ J.

[5] Talwar S, Dhir A, Islam N, Kaur P, Almusharraf A (2023). Resistance of multiple stakeholders to e-health innovations: integration of fundamental insights and guiding research paths. J Bus Res.

[6] Caverzagie KJ, Nousiainen MT, Ferguson PC (2017). Overarching challenges to the implementation of competency-based medical education. Med Teach.

[7] Kumaravel B, Hearn JH, Jahangiri L, Pollard R, Stocker CJ, Nunan D (2020). A systematic review and taxonomy of tools for evaluating evidence-based medicine teaching in medical education. Syst Rev.

[8] McCluskey A, Lovarini M (2005). Providing education on evidence-based practice improved knowledge but did not change behaviour: a before and after study. BMC Med Educ.

[9] Salehi S, Ballen CJ, Bolander Laksov K (2023). Global perspectives of the impact of the COVID-19 pandemic on learning science in higher education. PLoS One.

[10] Gordon M, Daniel M, Ajiboye A (2024). A scoping review of artificial intelligence in medical education: BEME Guide No. 84. Med Teach.

[11] McCarthy J, Minsky ML, Rochester N, Shannon CE (1955). A proposal for the Dartmouth summer research project on artificial intelligence: August 31, 1955. AI Mag.

[12] Ali O, Abdelbaki W, Shrestha A, Elbasi E, Alryalat MAA, Dwivedi YK (2023). A systematic literature review of artificial intelligence in the healthcare sector: benefits, challenges, methodologies, and functionalities. J Innov Knowl.

[13] (2022). Introducing ChatGPT. OpenAI.

[14] Mukherjee M, Le J, Chow YW (2025). Generative AI-enhanced intelligent tutoring system for graduate cybersecurity programs. Future Internet.

[15] Caudell TP, Summers KL, Holten J (2003). Virtual patient simulator for distributed collaborative medical education. Anat Rec B New Anat.

[16] Mirchi N, Bissonnette V, Yilmaz R, Ledwos N, Winkler-Schwartz A, Del Maestro RF (2020). The Virtual Operative Assistant: an explainable artificial intelligence tool for simulation-based training in surgery and medicine. PLoS ONE.

[17] Mastour H, Dehghani T, Moradi E, Eslami S (2025). Explainable artificial intelligence for predicting medical students’ performance in comprehensive assessments. Sci Rep.

[18] Vrdoljak J, Boban Z, Vilović M, Kumrić M, Božić J (2025). A review of large language models in medical education, clinical decision support, and healthcare administration. Healthcare (Basel).

[19] Waldock WJ, Zhang J, Guni A, Nabeel A, Darzi A, Ashrafian H (2024). The accuracy and capability of artificial intelligence solutions in health care examinations and certificates: systematic review and meta-analysis. J Med Internet Res.

[20] Shan G, Chen X, Wang C (2025). Comparing diagnostic accuracy of clinical professionals and large language models: systematic review and meta-analysis. JMIR Med Inform.

[21] Lucas HC, Upperman JS, Robinson JR (2024). A systematic review of large language models and their implications in medical education. Med Educ.

[22] Li J, Yin K, Wang Y, Jiang X, Chen D (2025). Effectiveness of generative artificial intelligence-based teaching versus traditional teaching methods in medical education: a meta-analysis of randomized controlled trials. BMC Med Educ.

[23] Samarakoon L, Fernando T, Rodrigo C, Rajapakse S (2013). Learning styles and approaches to learning among medical undergraduates and postgraduates. BMC Med Educ.

[24] Sharma AD, Goyal LS, Rehman A (2021). Artificial Intelligence and Internet of Things.

[25] Alliger GM, Janak EA (1989). Kirkpatrick’s levels of training criteria: thirty years later. Pers Psychol.

[26] Rethlefsen ML, Kirtley S, Waffenschmidt S (2021). PRISMA-S: an extension to the PRISMA Statement for Reporting Literature Searches in Systematic Reviews. Syst Rev.

[27] (2025). Homepage. Claude.

[28] Johnston S, Coyer FM, Nash R (2018). Kirkpatrick’s evaluation of simulation and debriefing in health care education: a systematic review. J Nurs Educ.

[29] Wang Y, Keitz S, Briel M (2025). Development of ROBUST-RCT: risk of bias instrument for use in systematic reviews-for randomised controlled trials. BMJ.

[30] Cohen J (1988). Statistical Power Analysis for the Behavioral Sciences.

[31] Kraft MA (2020). Interpreting effect sizes of education interventions. Educ Res.

[32] Norman GR, Sloan JA, Wyrwich KW (2003). Interpretation of changes in health-related quality of life: the remarkable universality of half a standard deviation. Med Care.

[33] Hozo SP, Djulbegovic B, Hozo I (2005). Estimating the mean and variance from the median, range, and the size of a sample. BMC Med Res Methodol.

[34] Higgins JP, Higgins JP, Chandler J, Cumpston M (2024). Cochrane Handbook for Systematic Reviews of Interventions Version 65.

[35] Altman DG, McKenzie JE, Veroniki AA, Higgins JP, Chandler J, Cumpston M Cochrane Handbook for Systematic Reviews of Interventions.

[36] Langan D, Higgins JPT, Jackson D (2019). A comparison of heterogeneity variance estimators in simulated random-effects meta-analyses. Res Synth Methods.

[37] IntHout J, Ioannidis JPA, Borm GF (2014). The Hartung-Knapp-Sidik-Jonkman method for random effects meta-analysis is straightforward and considerably outperforms the standard DerSimonian-Laird method. BMC Med Res Methodol.

[38] Higgins JPT, Thomas J, Chandler J, Cumpston M, Li T, Page MJ (2022). Cochrane Handbook for Systematic Reviews of Interventions.

[39] IntHout J, Ioannidis JPA, Rovers MM, Goeman JJ (2016). Plea for routinely presenting prediction intervals in meta-analysis. BMJ Open.

[40] Sterne JA, Egger M, Moher D, Boutron I, Higgins JP, Chandler J, Cumpston MS (2017). Cochrane Handbook for Systematic Reviews of Interventions.

[41] Sterne JAC, Sutton AJ, Ioannidis JPA (2011). Recommendations for examining and interpreting funnel plot asymmetry in meta-analyses of randomised controlled trials. BMJ.

[42] Page MJ, Sterne JAC, Boutron I (2023). ROB-ME: a tool for assessing risk of bias due to missing evidence in systematic reviews with meta-analysis. BMJ.

[43] Schünemann H, Brożek J, Guyatt G, Oxman A (2013). Handbook for Grading the Quality of Evidence and the Strength of Recommendations Using the GRADE Approach.

[44] Page MJ, McKenzie JE, Bossuyt PM (2021). The PRISMA 2020 statement: an updated guideline for reporting systematic reviews. Syst Rev.

[45] Page MJ, McKenzie JE, Bossuyt PM (2021). The PRISMA 2020 statement: an updated guideline for reporting systematic reviews. BMJ.

[46] Liaw SY, Tan JZ, Bin Rusli KD (2023). Artificial intelligence versus human-controlled doctor in virtual reality simulation for sepsis team training: randomized controlled study. J Med Internet Res.

[47] Ting PW, Wolffsohn JS (2026). Artificial intelligence-driven patient history and symptoms combined with slit-lamp eye simulation for enhancing the clinical training of students. Clin Exp Optom.

[48] Veras M, Dyer JO, Shannon H (2024). A mixed methods crossover randomized controlled trial exploring the experiences, perceptions, and usability of artificial intelligence (ChatGPT) in health sciences education. Digit Health.

[49] Yilmaz R, Bakhaidar M, Alsayegh A (2024). Real-time multifaceted artificial intelligence vs in-person instruction in teaching surgical technical skills: a randomized controlled trial. Sci Rep.

[50] Fazlollahi AM, Bakhaidar M, Alsayegh A (2022). Effect of artificial intelligence tutoring vs expert instruction on learning simulated surgical skills among medical students. JAMA Netw Open.

[51] Akutay S, Yüceler Kaçmaz H, Kahraman H (2024). The effect of artificial intelligence supported case analysis on nursing students’ case management performance and satisfaction: a randomized controlled trial. Nurse Educ Pract.

[52] Ali M, Rehman S, Cheema E (2025). Impact of artificial intelligence on the academic performance and test anxiety of pharmacy students in objective structured clinical examination: a randomized controlled trial. Int J Clin Pharm.

[53] Aneesh KV, Mohanan S, Jose S, Sajla K, Indulekha C, Sukumaran S (2025). Effectiveness of generative AI versus traditional resources for self-directed learning in physiology among MBBS students: a comparative interventional study. Int J Med Public Health.

[54] Arkan B, Dallı ÖE, Varol B (2025). The impact of ChatGPT training in the nursing process on nursing students’ problem-solving skills, attitudes towards artificial intelligence, competency, and satisfaction levels: single-blind randomized controlled study. Nurse Educ Today.

[55] Aronovitz N, Hazan I, Jedwab R (2024). The effect of real-time EF automatic tool on cardiac ultrasound performance among medical students. PLoS ONE.

[56] Ayan E, Bayraktar Y, Çelik Ç, Ayhan B (2024). Dental student application of artificial intelligence technology in detecting proximal caries lesions. J Dent Educ.

[57] Brügge E, Ricchizzi S, Arenbeck M (2024). Large language models improve clinical decision making of medical students through patient simulation and structured feedback: a randomized controlled trial. BMC Med Educ.

[58] Chang J, Bliss L, Angelov N, Glick A (2024). Artificial intelligence-assisted full-mouth radiograph mounting in dental education. J Dent Educ.

[59] Chen Y (2025). Evaluation of the impact of AI-driven personalized learning platform on medical students’ learning performance. Front Med.

[60] Cheng CT, Chen CC, Fu CY (2020). Artificial intelligence-based education assists medical students’ interpretation of hip fracture. Insights Imaging.

[61] Çiçek FE, Ülker M, Özer M, Kıyak YS (2025). ChatGPT versus expert feedback on clinical reasoning questions and their effect on learning: a randomized controlled trial. Postgrad Med J.

[62] Coşkun Ö, Kıyak YS, Budakoğlu Iİ (2025). ChatGPT to generate clinical vignettes for teaching and multiple-choice questions for assessment: a randomized controlled experiment. Med Teach.

[63] Digiacomo A, Orsini A, Cicchetti R (2025). Chatgpt vs traditional pedagogy: a comparative study in urological learning. World J Urol.

[64] Fazlollahi A, Bakhaidar M, Alsayegh A (2022). 510 artificial intelligence tutoring compared with expert instruction in neurosurgical simulation training: a randomized controlled trial. Clin Neurosurg.

[65] Ferrer-Peña R, Di-Bonaventura S, Pérez-González A, Lerín-Calvo A (2025). Feasibility of a randomized controlled trial of large AI-based linguistic models for clinical reasoning training of physical therapy students: pilot randomized parallel-group study. JMIR Form Res.

[66] Fung TCJ, Chan SL, Lam CFM (2025). Effects of generative artificial intelligence (GenAI) patient simulation on perceived clinical competency among global nursing undergraduates: a cross-over randomised controlled trial. BMC Nurs.

[67] Gan W, Ouyang J, Li H (2024). Integrating ChatGPT in orthopedic education for medical undergraduates: randomized controlled trial. J Med Internet Res.

[68] Han JW, Park J, Lee H (2025). Development and effects of a chatbot education program for self-directed learning in nursing students. BMC Med Educ.

[69] Höhne E, Bauer E, Bauer C (2025). A comparative bicentric study on ultrasound education for students: app- and AI-supported learning versus traditional hands-on instruction (AI-teach study). Acad Radiol.

[70] Huang S, Wen C, Bai X (2025). Exploring the application capability of ChatGPT as an instructor in skills education for dental medical students: randomized controlled trial. J Med Internet Res.

[71] Hui Z, Zewu Z, Jiao H, Yu C (2025). Application of ChatGPT-assisted problem-based learning teaching method in clinical medical education. BMC Med Educ.

[72] Kalam KA, Masoud FD, Muntaser A, Ranga R, Geng X, Goyal M (2025). ChatGPT as a learning tool for medical students: results from a randomized controlled trial. Cureus.

[73] Kestel S, Çalık A, Kuş M (2025). The effect of chatbot-supported instruction on nursing students’ history-taking questioning skills and stress level: a randomized controlled study. J Prof Nurs.

[74] Li J, Ouyang J, Liu J (2023). Artificial intelligence-based online platform assists blood cell morphology learning: a mixed-methods sequential explanatory designed research. Med Teach.

[75] Lyu X, Dong L, Fan Z (2024). Artificial intelligence-based graded training of pulmonary nodules for junior radiology residents and medical imaging students. BMC Med Educ.

[76] Mahrous A, Botsko DL, Elgreatly A, Tsujimoto A, Qian F, Schneider GB (2023). The use of artificial intelligence and game-based learning in removable partial denture design: a comparative study. J Dent Educ.

[77] McCarrick CA, McEntee PD, Boland PA (2025). A randomized controlled trial of a deep language learning model-based simulation tool for undergraduate medical students in surgery. J Surg Educ.

[78] Molu B (2025). Improving nursing students’ learning outcomes in neonatal resuscitation: a quasi-experimental study comparing ai-assisted care plan learning with traditional instruction. J Eval Clin Pract.

[79] Montagna M, Chiabrando F, De Lorenzo R, Rovere Querini P, Medical Students (2025). Impact of clinical decision support systems on medical students’ case-solving performance: comparison study with a focus group. JMIR Med Educ.

[80] Ng ISH, Siu A, Han CSJ (2025). Evaluating a custom chatbot in undergraduate medical education: randomised crossover mixed-methods evaluation of performance, utility, and perceptions. Behav Sci (Basel).

[81] Nissen L, Rother JF, Heinemann M, Reimer LM, Jonas S, Raupach T (2025). A randomised cross-over trial assessing the impact of AI-generated individual feedback on written online assignments for medical students. Med Teach.

[82] Ramsamooj A, Ibrahim SM, Gerriets VA, Cusick JK, Ramsamooj R (2025). Artificial intelligence versus traditional learning in a medical school setting. Cureus.

[83] Saatçi G, Korkut S, Ünsal A (2024). The effect of the use of artificial intelligence in the preparation of patient education materials by nursing students on the understandability, actionability and quality of the material: a randomized controlled trial. Nurse Educ Pract.

[84] Sahin GE, Bayram GA, Sierra AS (2025). Effects of artificial intelligence based physiotherapy educational approach in developing clinical reasoning skills: a randomized controlled trial. BMC Med Educ.

[85] Schropp L, Sørensen APS, Devlin H, Matzen LH (2024). Use of artificial intelligence software in dental education: a study on assisted proximal caries assessment in bitewing radiographs. Eur J Dent Educ.

[86] Kejingyun S, Mingjun R (2025). Randomized controlled study on the impact of problem-based learning combined with large language models on critical thinking skills in nursing students. Nurse Educ.

[87] Shin H, De Gagne JC, Kim SS, Hong M (2024). The impact of artificial intelligence-assisted learning on nursing students’ ethical decision-making and clinical reasoning in pediatric care. Comput Inform Nurs.

[88] Song D, Zhang P, Zhu Y (2025). Effects of generative artificial intelligence on higher-order thinking skills and artificial intelligence literacy in nursing undergraduates: a quasi-experimental study. Nurse Educ Pract.

[89] Staples G, Webster C, Baraza W (2025). SP3.03 artificially intelligent simulated patients in undergraduate surgical teaching: a single blinded randomized controlled study. Br J Surg.

[90] Svendsen K, Askar M, Umer D, Halvorsen KH (2024). Short-term learning effect of ChatGPT on pharmacy students’ learning. Explor Res Clin Soc Pharm.

[91] Shalong W, Yi Z, Bin Z (2025). Enhancing self-directed learning with custom GPT AI facilitation among medical students: a randomized controlled trial. Med Teach.

[92] Wang YF, Hsu MH, Yue-Feng Wang M (2025). Gamified mobile learning: an escape room chatbot to enhance medical terminology acquisition. Health Educ J.

[93] Wang Z, Fan TT, Li ML, Zhu NJ, Wang XC (2025). Feasibility study of using GPT for history-taking training in medical education: a randomized clinical trial. BMC Med Educ.

[94] Yeo CT, Ungi T, U-Thainual P, Lasso A, McGraw RC, Fichtinger G (2011). The effect of augmented reality training on percutaneous needle placement in spinal facet joint injections. IEEE Trans Biomed Eng.

[95] Gokkurt Yilmaz BN, Ozbey F, Yilmaz BE (2025). Effect of artificial intelligence-assisted personalized feedback on radiographic diagnostic performance of dental students: a controlled study. BMC Med Educ.

[96] Zeng J, Sun K, Qin P, Liu S (2025). Enhancing ophthalmology students’ awareness of retinitis pigmentosa: assessing the efficacy of ChatGPT in AI-assisted teaching of rare diseases-a quasi-experimental study. Front Med (Lausanne).

[97] Al Kahf S, Roux B, Clerc S (2023). Chatbot-based serious games: a useful tool for training medical students? A randomized controlled trial. PLoS ONE.

[98] Castano-Villegas N, Llano I, Villa MC, Zea J, Velásquez L (2025). Clinical med students’ validation of arkangel AI are their responses any better when supported by the AI?. SSRN.

[99] Dao L, Harmouch SS, Chin A (2022). Effect of an artificial intelligence chest x-ray disease prediction system on the radiological education of medical students: a pilot study. medRxiv.

[100] Döner A, Ceyhan Ö, Taşci S (2026). Effects of artificial intelligence-supported case analysis method on nursing students’ clinical competencies: mixed design research. Nurse Educ.

[101] Fang Q, Reynaldi R, Araminta AS (2025). Artificial intelligence (AI)-driven dental education: exploring the role of chatbots in a clinical learning environment. J Prosthet Dent.

[102] Huang Y, Xu B bei, Wang X yan, Luo Y cheng, Teng M miao, Weng X (2024). Implementation and evaluation of an optimized surgical clerkship teaching model utilizing ChatGPT. BMC Med Educ.

[103] Jiang Y, Fu X, Wang J (2024). Enhancing medical education with chatbots: a randomized controlled trial on standardized patients for colorectal cancer. BMC Med Educ.

[104] Kobayashi M, Iwamoto M, Une S, Kurazume R, Nakazawa A, Honda M (2022). Simulated communication skills training program effects using augmented reality with real‐time feedback: a randomized control study. Alzheimer’s & Dementia.

[105] Lau YH, Acharyya S, Wee CWL (2025). Effectiveness of traditional, artificial intelligence-assisted, and virtual reality training modalities for focused cardiac ultrasound skill acquisition: a randomised controlled study. Ultrasound J.

[106] Lee HY, Kim J, Choi H (2025). Comparing AI chatbot simulation and peer role-play for OSCE preparation: a pilot randomized controlled trial. BMC Med Educ.

[107] Louie YLJ, Lin CTL, Fong BYR, Yung KK, See C, Poon LCY (2025). AI vs conventional learning of clinical skills in O&G - A comparison. BJOG.

[108] Luo MJ, Bi S, Pang J (2025). A large language model digital patient system enhances ophthalmology history taking skills. NPJ Digit Med.

[109] Meng J, Chen J, Lai Y, Wang N, Ho CF, Tartari E (2025). AI-based augmented reality for hand hygiene: preliminary CRCT results in health sciences students. Antimicrob Resist Infect Control.

[110] Simsek-Cetinkaya S, Cakir SK (2023). Evaluation of the effectiveness of artificial intelligence assisted interactive screen-based simulation in breast self-examination: an innovative approach in nursing students. Nurse Educ Today.

[111] Usta SN, Silva E, Tekkanat H, Keskin C (2026). Virtual reality haptic simulators and mobile applications-potential AI-enhanced tools for improving clinical endodontic training: a randomized controlled trial. J Endod.

[112] Vannaprathip N, Haddawy P, Schultheis H, Suebnukarn S (2025). SDMentor: a virtual reality-based intelligent tutoring system for surgical decision making in dentistry. Artif Intell Med.

[113] Wu C, Chen L, Han M, Li Z, Yang N, Yu C (2025). Application of ChatGPT-based blended medical teaching in clinical education of hepatobiliary surgery. Med Teach.

[114] Wu D, Xiang Y, Wu X (2020). Artificial intelligence-tutoring problem-based learning in ophthalmology clerkship. Ann Transl Med.

[115] Benfatah M, Elazizi I, Lamiri A (2026). AI-assisted prebriefing to enhance simulation readiness in nursing education. Teach Learn Nurs.

[116] Benfatah M, Marfak A, Saad E, Hilali A, Nejjari C, Youlyouz-Marfak I (2024). Assessing the efficacy of ChatGPT as a virtual patient in nursing simulation training: a study on nursing students’ experience. Teach Learn Nurs.

[117] Jin HK, Lee HE, Kim E (2024). Performance of ChatGPT-3.5 and GPT-4 in national licensing examinations for medicine, pharmacy, dentistry, and nursing: a systematic review and meta-analysis. BMC Med Educ.

[118] Liu M, Okuhara T, Chang X (2024). Performance of ChatGPT across different versions in medical licensing examinations worldwide: systematic review and meta-analysis. J Med Internet Res.

[119] Murray E (2002). Challenges in educational research. Med Educ.

[120] Riding R (2005). Individual differences and educational performance. Educ Psychol (Lond).

[121] (2025). The AI research assistant. Elicit.

