# Supplementary material for: The Effectiveness of Artificial Intelligence in Undergraduate Health Professions Education: Systematic Review and Meta-Analysis of Randomized Controlled Trials
Source: JMIR Med Educ. 2026 May 5;12:e88933. doi: 10.2196/88933 (PMC13151782; doi:10.2196/88933)
Supplement: Multimedia Appendix 1 [file mededu-v12-e88933-s001.docx]

**Supplementary Material**

**The Effectiveness of Artificial Intelligence in Undergraduate Health Professions Education: a Systematic Review and Meta-analysis of Randomized Controlled Trials**

Nai Ming Lai, MRCPCH, Yin Sear Lim, MRCPCH, Min Thein Win, MMedSc, Prabal Bhargava, DNB (Ophthal), Paraidathathu Thomas, PhD, Qi Chwen Ong, MPH

Table of Contents

[Appendix 1. Detailed description of the review methods 3](#_Toc228786293)

[Criteria for selection of studies 3](#_Toc228786294)

[Inclusion criteria 3](#_Toc228786295)

[**Study design** 3](#_Toc228786296)

[**Setting** 3](#_Toc228786297)

[**Population** 3](#_Toc228786298)

[**Intervention** 3](#_Toc228786299)

[**Comparison** 3](#_Toc228786300)

[**Main outcomes** 3](#_Toc228786301)

[Differences between review and protocol 3](#_Toc228786302)

[Search strategies 4](#_Toc228786303)

[Data extraction, selection and coding 4](#_Toc228786304)

[Risk-of-bias assessment 4](#_Toc228786305)

[Differences between review and protocol 4](#_Toc228786306)

[Dealing with missing data 5](#_Toc228786307)

[Strategy for dealing with heterogeneity 5](#_Toc228786308)

[Assessment of publication and reporting bias 5](#_Toc228786309)

[Strategy for data synthesis 6](#_Toc228786310)

[AI intervention sub-categorisation 6](#_Toc228786311)

[Outcome selection for meta-analysis 7](#_Toc228786312)

[Statistical methods 7](#_Toc228786313)

[Differences between review and protocol 9](#_Toc228786314)

[Sensitivity analysis 10](#_Toc228786315)

[Subgroup analyses 10](#_Toc228786316)

[Differences between review and protocol 10](#_Toc228786317)

[Certainty-of-evidence rating 10](#_Toc228786318)

[References 12](#_Toc228786319)

[Appendix 2. Complete search strategies 13](#_Toc228786320)

[Appendix 3: Summary of findings table 20](#_Toc228786321)

[Appendix 4. List of shortlisted studies that were excluded 33](#_Toc228786322)

[Appendix 5. Characteristic of included RCTs 38](#_Toc228786323)

[Appendix 6. Risk of bias assessment summary graphs 52](#_Toc228786324)

[Appendix 7. Exploration of heterogeneity 53](#_Toc228786325)

[Appendix 8. Additional results of meta-analyses 54](#_Toc228786326)

[Funnel plot for theoretical knowledge : LLM personalised learning aid vs control 54](#_Toc228786327)

**Sensitivity analyses*……………………………………………………………………....55***

[Appendix 9. Published reviews that evaluated AI interventions for undergraduate Health Education 56](#_Toc228786328)

[Appendix 10. Prompts for LLMs (Claude AI and Elicit AI) and human verification process 59](#_Toc228786329)

[Claude (Sonnet 4.5 and 4.6) 59](#_Toc228786330)

[Elicit AI 60](#_Toc228786331)

# **Appendix 1. Detailed description of the review methods**

# Criteria for selection of studies

## **Inclusion criteria**

### **Study design**

We included randomised controlled trials (RCTs), randomised cross-over trials and cluster RCTs that fulfilled the following criteria:

### **Setting**

Clinical teaching (involving patient engagement and discussion), clinical and practical skills training, classroom learning in the form of problem-based, task-based or case-based learning, written assignment, as well as any form of clinical and written assessment.

### **Population**

Students of health care, including Medicine, Pharmacy, Nursing, Allied Health and other fields in Health Sciences, at the undergraduate level.

### **Intervention**

Artificial intelligence, which includes machine learning, deep learning and/or robotics applications, as well as Generative AI in any form, used for teaching and assessment purpose, either as the main or an adjunctive tool. For studies that assessed robotics and virtual or augmented reality, there must be an explicit statement on the use of AI in the paper to be accepted.

### **Comparison**

Conventional methods of teaching and assessment, which includes direct human face to face input, as well as the use of various technologies except AI.

### **Main outcomes**

Learning gain evaluated using various scales in accordance with the levels as depicted in the Kirkpatrick Model of Training Evaluation (including reaction (level 1), knowledge gain (level 2), behavioural change (level 3) and improvement in outcomes at the level of target-client, namely, patient health outcomes (level 4)).

## **Differences between review and protocol**

1. We excluded non-randomised trials as well as quasi-experimental studies because of the sufficient number of RCTs gathered in our latest search.
2. Population: we focused on studies at the undergraduate level and excluded studies on postgraduate level to limit our review to a manageable workload and because relevant reviews on postgraduate levels have been published.
3. Intervention: we added generative AI since the introduction of large language models (LLMs) during the development of this review. We also elaborated on the studies that assessed robotics or virtual/augmented reality, requiring studies to explicitly state the use of AI to be accepted, since robotics and virtual/augmented reality platforms may or may not involve AI during their operation.

# **Search strategies**

We searched MEDLINE (PubMed) and Cochrane Central Register of Controlled Trials (CENTRAL) (which covered EMBASE, CINAHL and trial registers including WHO International Trial Registry Platform and ClinicalTrials.gov), Embase, ERIC (Educational Resources Information Centre) and Web of Science (WoS) for published studies till 26 January 2026 without language and publication type restriction (see Appendix 2 for search strategies). We searched the reference lists of relevant reviews for additional studies but did not identify any.

# **Data extraction, selection and coding**

Two authors (NML, YSL) independently screened titles and abstracts for shortlisting and evaluated shortlisted articles in full texts to determine eligibility using EndNote. One author (NML) employed Claude Sonnet, a LLM application, to perform preliminary data extraction, including population, intervention, comparison and outcomes, checked accuracy of the extracted characteristics against the study full texts (examples of prompts available in Appendix 12), and manually transcribed the verified data into an Excel spreadsheet, while a second author (QCO) cross-checked the extracted data by NML against the full texts. We resolved disagreements by discussion leading to a consensus, with referral to the third author (MTW) as required.

# **Risk-of-bias assessment**

Two authors (NML, QCO) independently assessed risk-of-bias using the ROBUST-RCT tool, with minor adaptation in the domain of blinding from health providers to education providers. The tool consisted of six core domains (random sequence generation, allocation concealment, blinding of participants, blinding of educational providers, blinding of outcome assessors and outcome data not included in the analysis), with eight optional domains that we did not assess. We assessed risk-of-bias based on two broad groups of outcomes: subjective and objective outcomes. For subjective outcomes, we further separated our assessment into two sub-domains: perception or satisfaction and self-efficacy or confidence, as we considered the studies to have distinct risks in each sub-domain particularly with regards to the blinding of education providers. We discussed any disagreement leading to a consensus and if necessary, the involvement of the third review author (PT). As the ROBUST-RCT tool does not include an overall risk of bias domain because the determination of the overall risk of bias depends on the context of the outcome assessed, we ascertained the degree of concern that arose from the risk of bias of the studies only in the context of the certainty of evidence, using the GRADE approach. For this purpose, we examined the number of high-risk domains in a single study and the number of studies affected in the body of evidence (as considered under the GRADE domain of “study limitations”). For example, if the majority of the included studies for the outcome had a single high-risk domain, we would consider the body of evidence to have serious concerns in terms of risk of bias, and downgraded the certainty of the evidence by one level due to study limitations. However, if the majority of the included studies for the outcome assessed had two or more high-risk domains, we would consider the body of evidence to have very serious concerns in terms of risk of bias, and downgraded the certainty of the evidence by two levels due to study limitations.

## Differences between review and protocol

1. Data extraction, screening and selection: we screened all references manually via EndNote, instead of using ASReview as stated in our protocol, because we found ASReview in this particular instance suboptimal in identifying relevant papers when the preliminary yield is large.
2. Risk of bias assessment: we used the newly published ROBUST-RCT tool instead of the Cochrane RoB 2 tool because of its user-friendliness. We did not use other tools mentioned in the protocol (ROBINS-I tool and Joanna Briggs Institute Checklist) following our decision to include only RCTs.

# **Dealing with missing data**

We determined the dropout rates from each study and assessed the number of participants that were initially randomised against the total number analysed. We considered a dropout rate greater than 20% as significant [1]. We determined that if we found a significant dropout rate with no reasonable explanation, or markedly different dropout rates between the assigned groups, we would judge the study at high risk in the domain of data not included in the analysis in our risk-of-bias assessment. If we considered the extent of missing data to be critical to the final estimates in our meta-analysis, we would have contacted the authors of the individual studies to request further information. In this review, we did not consider the proportion of missing data to be critical, and so we did not contact any study author for further information.

# **Strategy for dealing with heterogeneity**

We visually inspected the forest plots for any evidence of heterogeneity. We used the I² statistic to measure heterogeneity among the trials in each analysis. We identified and measured clinical, methodological and statistical heterogeneity as recommended in the Cochrane Handbook for Systematic Reviews of Interventions [2]. To evaluate clinical heterogeneity, we assessed variability in the participants’ educational setting, country and programme enrolled, as well as the major category and specific sub-type of AI intervention delivered, and outcome measurements, and evaluated whether these study level factors moderated the direction and magnitude of the pooled estimates, in addition to the resultant changes in the degree of heterogeneity as measured by the I². We also assessed variability in the risk-of-bias of included studies. For statistical heterogeneity, we classified heterogeneity with I² as follows:

- 0% to 40%: might not be important;
- 30% to 60%: may represent moderate heterogeneity;
- 50% and above: may represent substantial heterogeneity.

# *Assessment of publication and reporting bias*

We created funnel plots to screen for small study effects, which is one reason for publication bias for outcomes in which there were 10 or more included studies, using RevMan. If small study effects were suggested by significant asymmetry in the funnel plot, we downgraded the certainty-of-evidence for concerns on strong suspicion of publication bias due possibly to small study effects [3].

In this review, we did not specifically assess the risk-of-bias arising from expected but missing results in a synthesis (i.e. reporting biases), using recently established risk-of-bias tools such as the RoB-ME tool [4].

# **Strategy for data synthesis**

Studies with suitable numerical outcome data, either reported in text, table or derivable from graphs were eligible for synthesis. We tabulated the major components of the intervention and comparison in each study in the characteristics of included studies table. We sub-categorised AI interventions based on technology type (LLM vs non-LLM) and predominant educational functions as detailed in the next segment “AI intervention sub-categorisation”. We divided the outcomes according to the levels of Kirkpatrick’s Model of Training Evaluation, with further division for different sub-domains based on the characteristics of the outcomes assessed: perception or satisfaction and self-efficacy or confidence (Kirkpatrick level 1), and theoretical knowledge, clinical skills and practical skills (Kirkpatrick level 2), based on consensus among the review authors. No studies reporting level 3 (behavioural change in clinical practice) or level 4 (patient health outcomes) outcomes were identified.

If studies reported multiple outcomes in the same domain, we exercised our judgement in selecting the most suitable outcomes for our meta-analysis following a discussion among the review authors. For example, we used combined score in preference to scores for individual components, and if no combined scores were reported, we selected one component that was most relevant to the domain; and if there were multiple components of equal relevance, we would manually derive the combined mean and SD following the formulae as recommended in chapter 10 of the Cochrane Handbook [5].

For randomized cross-over studies, we planned to include outcome data from the first period only, before the cross-over, following one of the approaches as suggested in the Cochrane Handbook [5]. If first-period data was not available, we would conduct sensitivity analysis to assess the impact of these cross-over studies on the pooled results.

## **AI intervention sub-categorisation**

Within each Kirkpatrick sub-domain, we further sub-categorised studies by the type and functional role of the AI intervention. This granular approach was adopted to more usefully inform educational practice. We distinguished two broad technology categories: large language model (LLM)-based applications and non-LLM applications (encompassing earlier machine learning, natural language processing rule-based systems, AI-moderated adaptive platforms, AI-guided procedure assistants, AI imaging diagnostic aids, and AI-integrated virtual reality environments). Within each technology category, we further classified interventions by their primary educational function, resulting in the following sub-categories (a detailed working definition of each application is available in Table 1 within the manuscript):

**LLM-based applications**

- LLM content generator
- LLM personalised learning aid
- LLM virtual patient
- LLM gamification tool
- LLM-integrated curriculum
- LLM combination

**Non-LLM applications**

- NLP rule-based chatbot
- NLP rule-based virtual patient
- Non-LLM AI-moderated adaptive learning platform
- Non-LLM AI procedure assistant
- Non-LLM AI imaging diagnostic aid
- Non-LLM AI-VR virtual doctor
- Non-LLM AI gamification tool

Where a study involved a combination of non-LLM AI functions, this was noted accordingly.

## **Outcome selection for meta-analysis**

If studies reported multiple outcomes in the same domain, we exercised our judgement in selecting the most suitable outcomes for our meta-analysis following discussion among the review authors. We used combined scores in preference to scores for individual components; if no combined scores were reported, we selected the component most relevant to the domain; and if there were multiple components of equal relevance, we derived combined mean and SD using formulae as recommended in the Cochrane Handbook.

## **Statistical methods**

We performed random-effects meta-analysis. For continuous outcomes, we used the standardised mean difference (SMD) to pool study results, as included studies used different measurement scales. We used Cohen's benchmarks as a descriptive reference (SMD <0.2 small, 0.2–0.5 small to medium, >0.5–0.8 medium to large, >0.8 large) [7], while recognising that these thresholds were not developed for educational intervention contexts and should not be interpreted as definitive indices of educational importance [8]. Accordingly, we contextualised effect sizes using a default minimally important difference (MID) of SMD 0.5, consistent with the empirically-derived half-standard-deviation rule [9], applying this threshold in our interpretation of synthesis results. For dichotomous outcomes we reported results using relative risk (RR)[8]. All point estimates are presented with their 95% confidence intervals (CI).

For multi-arm studies, we selected the arms that are relevant to our review, AI and control group. In studies with multiple control groups, we only selected the control group that represented current standard educational intervention for the study. We ensured that each study was only represented once in each analysis, thereby avoiding multiple counting of the participants.

Heterogeneity variance (τ²) was estimated using the Restricted Maximum Likelihood (REML) method, which provides more reliable estimates of between-study variance than the conventional DerSimonian-Laird method, particularly when the number of studies is small [10]. Confidence intervals for pooled estimates were calculated using the Hartung-Knapp-Sidik-Jonkman (HKSJ) method, which adjusts the standard error of the pooled estimate based on the estimated heterogeneity and tends to produce more conservative (wider) confidence intervals than conventional methods, thereby reducing the risk of false-positive conclusions when heterogeneity is substantial or the number of studies is small [11].

In addition to pooled point estimates and 95% CIs, we calculated 95% prediction intervals for each comparison, and reported the intervals for analyses involving two or more studies. The prediction interval estimates the range within which the true effect of AI is likely to fall in a new, similar study, thereby communicating the uncertainty arising from between-study heterogeneity more intuitively than the 95% CI alone. A prediction interval crossing the null (SMD = 0 or RR = 1) indicates that, despite a statistically significant pooled estimate, the intervention effect may be negligible or even reversed in some settings [12].

We derived missing standard deviations (SDs) by multiplying the standard error (SE) by the square root of the sample size in the corresponding group. For studies reporting results as median and interquartile range (IQR), we approximated the median as the mean and estimated the SD by dividing the IQR by 1.35, as recommended by Hozo et al.[14]

Analyses were performed using RevMan (The Cochrane Collaboration).

Following are examples of studies from which outcome data extraction for synthesis was non-straightforward. The issues in the studies are followed by our approach in selecting or deriving the outcome data (please refer to Appendix 13 for the analysis numbers).

1. Analysis 1.1 (Kirkpatrick level 1: perception or satisfaction (LLM content generator vs control)):
   1. Akutay 2024: Mean scores for LLM group were reported by the authors as 9.08 (SD, 1.45) in the main text, but the other way around in their Table results. We followed the figures in the main text as they were accompanied by interpretation- one group higher than the other, which was more likely to be what the authors intended.
2. Analysis 1.2 (Kirkpatrick level 1: perception or satisfaction (LLM personalised learning aid vs control)):
   1. Huang 2025: Exact mean scores and SD values were not reported in texts. We extrapolated the means from the graphs. p values were given, and SD of difference derived from p value, means and participant number. Since no of participants were almost identical in both groups, SD change was taken as SD of both groups.
3. Analysis 1.15 (Kirkpatrick level 1: self-efficacy and confidence (LLM personalised learning aid vs control)):
   1. Sahin 2025: Change scores instead of end scores were used, because of a clear difference in the pre-test scores between groups (intervention: 70.7±21.4 and control 109.6±19.0), as suggested in the Cochrane handbook.
   2. Huang 2025: Exact mean scores and SD values were not reported in texts. We extrapolated the means from the graphs. p values were given, and SD of difference derived from p value, means and participant number. Since no of participants were almost identical in both groups, SD change was taken as SD of both groups.
4. Analysis 1.31 (Kirkpatrick level 2: theoretical knowledge (LLM personalised learning aid vs control)):
   1. Digiacomo 2025: the outcome data were derived from graphs – median taken as means and SD from extrapolated IQR by dividing the IQR by 1.35, as proposed by Hozo et al.
5. Analysis 1.43 (Kirkpatrick level 2: clinical skills (LLM personalised learning aid vs control)):
   1. Montagna 2025: The authors reported the completeness and accuracy ratings. In view of their equal importance in our consideration, we derived an overall rating (mean and SD) by combining the completeness and accuracy ratings. In doing this, two non-AI groups (CPG and OR) combined to form a combined control groups, with their combined completeness and accuracy ratings (mean and SD) combined again between the two groups.
6. Analysis 1.47 (Kirkpatrick level 2: clinical skills (non-LLM AI imaging diagnostic aid vs control)):
   1. Cheng 2020: there are two possible scores to be used for the intervention group: AI-assisted test score (With-AI score) (88.87 , SD=5.51), or postlearning score (84.93 ± 14.53 ). We opted to use the WithAI score, because it was measured immediately at the end of the intervention, recognising that the score represented a form of a combined effort between AI and human participants. The postlearning score, although entirely from human participants, were taken 1 week after the intervention. We accept that the choice of the WithAI score in meta-analysis represented our team’s preference, and a good case could also have been made for the use of postlearning.
   2. Dao 2022: The SD were derived manually from the means, 95% confidence intervals and the number of participants in each group.
7. Analysis 1.59 (Kirkpatrick level 2: practical skills (non-LLM AI procedure assistant vs control)):
   1. Chang 2024: the SD for control group is 0.00 in the manuscript. We opted to use 0.01 as a plausible proxy to retain the study in the meta-analysis, because RevMan cannot compute a weighted mean difference or SMD from a group with SD = 0.
   2. Fazlollahi 2022: The SD were derived manually from the means, 95% confidence intervals and the number of participants in each group.
   3. Yilmaz 2024: The manuscript did not provide CIs or SDs. The SD of 0.11 was derived from an estimated SE of 0.2, which in turn was derived from the graph (figure 4c)).

## **Differences between review and protocol**

1. Outcome sub-domains: in our review, we subdivided the outcomes within each Kirkpatrick level according to the nature of the outcomes, namely, perception or satisfaction and self-efficacy or confidence (Kirkpatrick level 1), and theoretical knowledge, clinical skills and practical skills (Kirkpatrick level 2), based on consensus among the review authors.
2. AI intervention sub-categorisation: we introduced a granular classification of AI interventions by technology type (LLM vs non-LLM) and primary educational function within each Kirkpatrick sub-domain. This was not specified in the original protocol but was adopted in response to the substantial technological heterogeneity among included studies and to improve educational interpretability of the evidence.
3. Statistical methods: we used REML for τ² estimation and the HKSJ method for confidence interval calculation, rather than the DerSimonian-Laird method used in the original review version. We additionally calculated 95% prediction intervals for all multi-study comparisons. These updates were adopted to improve statistical robustness and to more transparently communicate uncertainty arising from heterogeneity.
4. We added an updated section on the strategy to select or manage outcome if multiple outcomes were reported by individual studies.
5. We added a statement on how we proposed to handle data presented by randomised cross over trials in the synthesis.

# **Sensitivity analysis**

We performed sensitivity analyses for the primary outcomes, and any secondary outcomes with at risk 5 included studies in each category evaluated, to assess the impact of excluding studies with high overall risk of bias. In the review, the vast majority of studies was judged to have at least some major domains with high risk-of-bias, including allocation concealment and blinding either of participants or education providers. In this review, we only performed sensitivity analysis for a single comparison of LLM personalised learning aid versus control for the outcome of theoretical knowledge, as this was the only analysis with 5 or more studies in each risk of bias stratum.

# **Subgroup analyses**

We performed the following pre-specified and post-hoc subgroup analyses for the overall comparison of AI intervention (any) versus control, where data permitted, in addition to the separate comparisons created by sub-dividing the AI interventions into multiple sub-categories:

- Field of study: Medicine, Nursing, Dentistry, Pharmacy, Physiotherapy, Health Sciences and others.
- Region of study: Asia, North America, South America, Central America, Africa, Europe, Oceania, and multi-continental.
- Technology type: LLM-based versus non-LLM AI applications.
- Primary function of the AI application: teaching and learning versus assessment versus combined teaching-learning and assessment.
- Session structure: single session versus multiple sessions.

These subgroup analyses were performed for outcomes with a sufficient number of included studies to allow meaningful comparison (generally ≥ 5 studies per subgroup). We did not perform formal tests for subgroup interaction but used subgroup estimates and their 95% CIs to inform interpretation. All subgroup analyses should be considered exploratory.

## **Differences between review and protocol**

1. Contrary to the original protocol, which specified that subgroup analyses would be limited to healthcare discipline given the constraint of undergraduate studies only, we performed additional subgroup analyses by region, technology type (LLM vs non-LLM), primary function, and session structure. These additional analyses were conducted post-hoc to explore sources of clinical and methodological heterogeneity, particularly given the substantial I² values observed across most outcomes. Conversely, we did not perform subgroup analyses by healthcare discipline under each individual AI sub-category comparison, as the number of studies per cell was insufficient.

# **Certainty-of-evidence rating**

Two authors (NML, QCO) independently assessed the certainty-of-evidence for seven major outcomes — self-efficacy or confidence, perception or satisfaction, theoretical knowledge, clinical skills, practical skills, task efficiency and generic or personal skills — using the GRADE approach.[15] We considered evidence from RCTs as high-certainty to begin with, downgrading one level for serious (or two levels for very serious) limitations based on five considerations: risk-of-bias, inconsistency across studies, indirectness of the evidence, imprecision of estimates, and publication bias. We used GRADEpro GDT (https://www.gradepro.org/) to create the summary of findings table.

In assessing study limitations (risk-of-bias), two authors (NML, QCO) independently evaluated the level of downgrading based on the degree of concern on the risk-of-bias status of the body of evidence included in the outcome. As the risk-of-bias assessment tool we used, ROBUST-RCT, does not include an overall risk-of-bias domain, we exercised judgment in downgrading based on the proportion and characteristics of high-risk domains. For example, if the majority of the included studies had high risk of bias in a single major domain (for example, blinding), we assigned the body of evidence as having serious concerns on study limitations, hence downgraded the evidence certainty by one level in this domain. On the other hand, if we found high risk-of-bias in multiple major domains (for example, random sequence generation as well as blinding), we assigned the body of evidence as having very serious concerns on study limitations and downgraded the certainty by two levels in this domain.

We assessed imprecision in accordance with the current GRADE guidance, which recommends a contextualised approach based on the minimally important difference (MID) or, when the MID is unavailable, on the magnitude of the effect and the width of the 95% CI in relation to the null effect. For continuous outcomes measured using SMD, we applied the following considerations:

- We downgraded by one level when the 95% CI was wide and crossed a default MID threshold of SMD 0.5 (corresponding to a medium effect size) [9], or when the 95% CI included both appreciable benefit and negligible or no benefit, indicating substantial uncertainty about the true direction or magnitude of effect.
- We downgraded by two levels when the 95% CI was very wide and crossed both directions of appreciable effect (i.e., spanned from potential harm to potential benefit beyond the MID threshold), reflecting very serious imprecision.
- In addition to the 95% CI, we considered the 95% prediction interval when judging imprecision: a prediction interval that widely crossed the null was treated as additional evidence of very serious imprecision, supporting further downgrading, particularly when few studies contributed to the estimate.
- The total sample size contributed to the assessment of imprecision, with comparisons involving fewer than 400 total participants generally considered underpowered for detecting small to medium effects and thus potentially subject to imprecision downgrading.

We interpreted the GRADE ratings as follows:

- **High certainty:** we are very confident that the true effect lies close to that of the estimate of the effect.
- **Moderate certainty:** we are moderately confident in the effect estimate; the true effect is likely to be close to the estimate of the effect, but there is a possibility that it is substantially different.
- **Low certainty:** our confidence in the effect estimate is limited; the true effect may be substantially different from the estimate of the effect.
- **Very low certainty:** we have very little confidence in the effect estimate; the true effect is likely to be substantially different from the estimate of effect.

We justified all decisions to downgrade the certainty of the evidence using footnotes and made comments to aid the reader’s understanding of the review where necessary.

# **References**

1. Guyatt GH, Sackett DL, Cook DJ. Users’ guides to the medical literature. II. How to use an article about therapy or prevention. A. Are the results of the study valid? Evidence-Based Medicine Working Group. JAMA. 1993;270(21):2598-2601.

2. Higgins JPT, Thomas J, Chandler J, et al. Cochrane Handbook for Systematic Reviews of Interventions version 6.3 (updated February 2022). The Cochrane Collaboration; 2022.

3. Sterne JA, Egger M, Moher D, Boutron I. Chapter 10: Addressing reporting biases. In: Higgins JP, et al., eds. Cochrane Handbook for Systematic Reviews of Interventions Version 5.2.0. The Cochrane Collaboration; 2017.

4. Page MJ, Sterne JAC, Boutron I, et al. ROB-ME: a tool for assessing risk of bias due to missing evidence in systematic reviews with meta-analysis. BMJ. 2023;383:e076754.

5. Higgins JP ES, Li T. Chapter 23: Including variants on randomized trials [last updated October 2019]. In: Higgins JP TJ, Chandler J, Cumpston M, Li T, Page MJ, et al, editor(s). editor. Cochrane Handbook for Systematic Reviews of Interventions version 65. Available from cochrane.org/handbook: Cochrane 2024.

6. Deeks JJ HJ, Altman DG, McKenzie JE, Veroniki AA (editors). Chapter 10: Analysing data and undertaking meta-analyses In: Higgins JP TJ, Chandler J, Cumpston M, Li T, Page MJ, et al, editor(s). , editor. Cochrane Handbook for Systematic Reviews of Interventions version 65 Cochrane 2024.

7. Cohen J. Statistical Power Analysis for the Behavioral Sciences. 2nd ed. Lawrence Erlbaum Associates; 1988.

8. Kraft, M. A. (2020). Interpreting Effect Sizes of Education Interventions. Educational Researcher, 49(4), 241-253. <https://doi.org/10.3102/0013189X20912798>.

9. Norman GR, Sloan JA, Wyrwich KW. Interpretation of changes in health-related quality of life: the remarkable universality of half a standard deviation. Med Care. 2003 May;41(5):582-92. doi: 10.1097/01.MLR.0000062554.74615.4C. PMID: 12719681.

10. Higgins JPT, Thomas J, Chandler J, et al. Cochrane Handbook for Systematic Reviews of Interventions version 6.3 (updated February 2022). The Cochrane Collaboration; 2022.

11. Hartung J, Knapp G. A refined method for the meta-analysis of controlled clinical trials with binary outcome. Stat Med. 2001;20(24):3875-3889. Sidik K, Jonkman JN. A simple confidence interval for meta-analysis. Stat Med. 2002;21(21):3153-3159.

12. Veroniki AA, Jackson D, Viechtbauer W, et al. Methods to estimate the between-study variance and its uncertainty in meta-analysis. Res Synth Methods. 2016;7(1):55-79.

13. Higgins JPT, Thompson SG, Spiegelhalter DJ. A re-evaluation of random-effects meta-analysis. J R Stat Soc Ser A. 2009;172(1):137-159.

14. Hozo SP, Djulbegovic B, Hozo I. Estimating the mean and variance from the median, range, and the size of a sample. BMC Med Res Methodol. 2005;5(1):13.

15. Guyatt GH, Oxman AD, Vist GE, et al. GRADE: an emerging consensus on rating quality of evidence and strength of recommendations. BMJ. 2008;336(7650):924-926.

# **Appendix 2. Complete search strategies**

**MEDLINE (PubMed)**

#1 "artificial intelligence"[Title/Abstract]

#2 "artificial intelligence"[MeSH Terms]

#3 "AI"[Title/Abstract]

#4 "machine learning"[Title/Abstract]

#5 "machine learning"[MeSH Terms]

#6 "deep learning"[Title/Abstract]

#7 robot*[Title/Abstract]

#8 "robotics"[MeSH Terms]

#9 neural network*[Title/Abstract]

#10 neural network model[MeSH Terms]

#11 "learning management system" [Title/Abstract]

#12 "intelligent tutoring"[Title/Abstract]

#13 Computational Intelligence [MeSH Terms]

#14 Computer Reasoning [MeSH Terms]

#15 Computer Vision Systems [MeSH Terms]

#16 Knowledge Acquisition (Computer) [MeSH Terms]

#17 Knowledge Representation (Computer) [MeSH Terms]

#18 Machine Intelligence [MeSH Terms]

#19 Transfer Learning [MeSH Terms]

#20 Hierarchical Learning [MeSH Terms]

#21 Expert System [MeSH Terms]

#22 large language models [MeSH Terms]

#23 "large language model*" [Title/Abstract]

#24 "LLM*" [Title/Abstract]

#25 artificial intelligence, generative [MeSH Terms]

#26 "generative artificial intelligence"[Title/Abstract]

#27 "generative AI"[Title/Abstract]

#28 chatbots [MeSH Terms]

#29 chatbot* [Title/Abstract]

#30 "conversational agent*" [Title/Abstract]

#31 "natural language processing" [MeSH Terms]

#32 "natural language processing"[Title/Abstract]

#33 "NLP"[Title/Abstract]

#34 "computer assisted instruction"[MeSH Terms]

#35 "computer assisted instruction"[Title/Abstract]

#36 "virtual patient*"[Title/Abstract]

#37 "virtual tutor*"[Title/Abstract]

#38 #1 OR #2 OR #3 OR #4 OR #5 OR #6 OR #7 OR #8 OR #9 OR #10 OR #11 OR #12 OR #13 OR #14 OR #15 OR #16 OR #17 OR #18 OR #19 OR #20 OR #21 OR #22 OR #23 OR #24 OR #25 OR #26 OR #27 OR #28 OR #29 OR #30 OR #31 OR #32 OR #33 OR #34 OR #35 OR #36 OR #37

#39 health education[MeSH Terms]

#40 medical education[MeSH Terms]

#41 Educat*[Title/Abstract]

#42 train*[Title/Abstract]

#43 teach*[Title/Abstract]

#44 learn*[Title/Abstract]

#45 #39 OR #40 OR #41 OR #42 OR #43 OR #44

#46 #38 AND #45

#47 "comparative study"[Publication Type]

#48 #46 AND #47

**Cochrane CENTRAL**

#1 ("artificial intelligence"):ti,ab,kw

#2 MeSH descriptor: [Artificial Intelligence] explode all trees

#3 (AI):ti,ab,kw

#4 ("machine learning"):ti,ab,kw

#5 MeSH descriptor: [Machine Learning] explode all trees

#6 ("deep learning"):ti,ab,kw

#7 (robot*):ti,ab,kw

#8 MeSH descriptor: [Robotics] explode all trees

#9 (neural network*):ti,ab,kw

#10 MeSH descriptor: [Neural Networks, Computer] explode all trees

#11 ("learning management system"):ti,ab,kw

#12 ("intelligent tutoring"):ti,ab,kw

#13 MeSH descriptor: [Artificial Intelligence] explode all trees

#14 MeSH descriptor: [Artificial Intelligence] explode all trees

#15 MeSH descriptor: [Artificial Intelligence] explode all trees

#16 MeSH descriptor: [Artificial Intelligence] explode all trees

#17 MeSH descriptor: [Artificial Intelligence] explode all trees

#18 MeSH descriptor: [Artificial Intelligence] explode all trees

#19 MeSH descriptor: [Machine Learning] explode all trees

#20 MeSH descriptor: [Deep Learning] explode all trees

#21 MeSH descriptor: [Expert Systems] explode all trees

#22 MeSH descriptor: [Large Language Models] explode all trees

#23 ("large language" NEXT model*):ti,ab,kw

#24 (LLM):ti,ab,kw

#25 MeSH descriptor: [Generative Artificial Intelligence] explode all trees

#26 ("generative artificial intelligence"):ti,ab,kw

#27 ("generative AI"):ti,ab,kw

#28 MeSH descriptor: [Generative Artificial Intelligence] explode all trees

#29 (chatbot*):ti,ab,kw

#30 (conversational NEXT agent*):ti,ab,kw

#31 MeSH descriptor: [Natural Language Processing] explode all trees

#32 ("natural language processing"):ti,ab,kw

#33 (NLP):ti,ab,kw

#34 MeSH descriptor: [Computer-Assisted Instruction] explode all trees

#35 ("computer assisted instruction"):ti,ab,kw

#36 (virtual NEXT patient*):ti,ab,kw

#37 (virtual NEXT tutor*):ti,ab,kw

#38 #1 OR #2 OR #3 OR #4 OR #5 OR #6 OR #7 OR #8 OR #9 OR #10 OR #11 OR #12 OR #13 OR #14 OR #15 OR #16 OR #17 OR #18 OR #19 OR #20 OR #21 OR #22 OR #23 OR #24 OR #25 OR #26 OR #27 OR #28 OR #29 OR #30 OR #31 OR #32 OR #33 OR #34 OR #35 OR #36 OR #37

#39 MeSH descriptor: [Health Education] explode all trees

#40 MeSH descriptor: [Education, Medical] explode all trees

#41 (Educat*):ti,ab,kw

#42 (train*):ti,ab,kw

#43 (teach*):ti,ab,kw

#44 (learn*):ti,ab,kw

#45 #39 OR #40 OR #41 OR #42 OR #43 OR #44

#46 #38 AND #45 (limited to “Trials”)

**EMBASE (OVID)**

1 artificial intelligence.ti,ab.

2 exp artificial intelligence/

3 AI.ti,ab.

4 machine learning.ti,ab.

5 exp machine learning/

6 deep learning.ti,ab.

7 robot*.ti,ab.

8 exp robotics/

9 neural network.ti,ab.

10 exp convolutional neural network/ or exp artificial neural network/

11 learning management system.ti,ab.

12 intelligent tutoring.ti,ab.

13 computational intelligence.ti,ab.

14 exp automated reasoning/

15 exp computer vision/

16 transfer learning.ti,ab.

17 Hierarchical learning.ti,ab.

18 exp expert system/

19 exp large language model/

20 large language model*.ti,ab.

21 LLM*.ti,ab.

22 exp generative artificial intelligence/

23 generative artificial intelligence.ti,ab.

24 generative AI.ti,ab.

25 exp chatbot/

26 chatbot*.ti,ab.

27 conversational agent*.ti,ab. 661

28 exp natural language processing/

29 natural language processing.ti,ab.

30 NLP.ti,ab.

31 exp computer assisted education/

32 computer assisted education.ti,ab.

33 virtual patient*.ti,ab.

34 virtual tutor*.ti,ab.

35 1 or 2 or 3 or 4 or 5 or 6 or 7 or 8 or 9 or 10 or 11 or 12 or 13 or 14 or 15 or 16 or 17 or 18 or 19 or 20 or 21 or 22 or 23 or 24 or 25 or 26 or 27 or 28 or 29 or 30 or 31 or 32 or 33 or 34

36 exp medical education/

37 medical educat*.ti,ab.

38 health profession* education*.ti,ab.

39 medical train*.ti,ab.

40 Nursing train*.ti,ab.

41 nursing educat*.ti,ab.

42 pharmacist train*.ti,ab.

43 (pharmacist educat* or pharmacy educat*).ti,ab.

44 (dental educat* or dentistry educat*).ti,ab.

45 dental train*.ti,ab.

46 36 or 37 or 38 or 39 or 40 or 41 or 42 or 43 or 44 or 45

47 35 and 46

**Web of Science collection**

TI=((“artificial intelligence” or AI or “machine learning” or “deep learning” or “robot*” or “neural network” or “learning management system*” or “intelligent tutor*” or “computational intelligence” or “automated reasoning” or “computer vision” or “transfer learning” or “hierarchical learning” or “expert system*” or “large language model*” or “LLM*” or “generative artificial intelligence” or “generative AI” or “chatbot*” or “conversational agent*” or “natural language processing” or “NLP” or “computer assisted education” or “virtual patient*” or “virtual tutor*”) and (“medical educat*” or “health profession educat*” or “medical train*” or “nursing train*” or “nursing educat*” or “pharmacist train*” or “pharmacist educat*” or “pharmacy educat*” or “dental educat*” or “dentistry educat*” or “dental train*”)) OR AB=((“artificial intelligence” or AI or “machine learning” or “deep learning” or “robot*” or “neural network” or “learning management system*” or “intelligent tutor*” or “computational intelligence” or “automated reasoning” or “computer vision” or “transfer learning” or “hierarchical learning” or “expert system*” or “large language model*” or “LLM*” or “generative artificial intelligence” or “generative AI” or “chatbot*” or “conversational agent*” or “natural language processing” or “NLP” or “computer assisted education” or “virtual patient*” or “virtual tutor*”) AND (“medical educat*” or “health profession educat*” or “medical train*” or “nursing train*” or “nursing educat*” or “pharmacist train*” or “pharmacist educat*” or “pharmacy educat*” or “dental educat*” or “dentistry educat*” or “dental train*”))

**Education Resource Information Centre (ERIC)**

*Search terms have been modified for maximum yield in ERIC (straight adaptation of search strategies from conventional databases did not work as 0 hit was returned)*

(abstract: "artificial intelligence" OR abstract: AI OR abstract: "machine learning" OR abstract: "deep learning" OR abstract: "robotics" OR abstract: “neural network” OR abstract: "learning management" OR abstract: "intelligent tutoring" OR abstract: "generative" OR abstract: "large language" OR abstract: LLM OR abstract: chatbot OR abstract: "conversational agent" OR abstract: "natural language" OR abstract: "virtual patient" OR abstract: "virtual tutor")(abstract: health OR abstract: medicine OR abstract: dentistry OR abstract: nursing)

Search yields with sequential filter

- Overall: 26813
- Limited to journal articles: 12481
- Limited to “higher education”: 1025
- Shortlisted after manual inspection for AI and Health Education: 5

|  |
| --- |

| **Appendix 2:** **PRISMA-S Checklist**   \| **Section/topic** \| **#** \| **Checklist item** \| **Location(s) Reported** \| \| --- \| --- \| --- \| --- \| \| **INFORMATION SOURCES AND METHODS** \| \| \| \| \| Database name \| 1 \| Name each individual database searched, stating the platform for each. \| Methods, Search strategies \| \| Multi-database searching \| 2 \| If databases were searched simultaneously on a single platform, state the name of the platform, listing all of the databases searched. \|  \| \| Study registries \| 3 \| List any study registries searched. \| Methods, Search strategies (listed under CENTRAL search) \| \| Online resources and browsing \| 4 \| Describe any online or print source purposefully searched or browsed (e.g., tables of contents, print conference proceedings, web sites), and how this was done. \| We did not search these resources, mentioned in Methods, Search strategies \| \| Citation searching \| 5 \| Indicate whether cited references or citing references were examined, and describe any methods used for locating cited/citing references (e.g., browsing reference lists, using a citation index, setting up email alerts for references citing included studies). \| Methods, Search strategies \| \| Contacts \| 6 \| Indicate whether additional studies or data were sought by contacting authors, experts, manufacturers, or others. \| We did not search these resources, mentioned in Methods, Search strategies \| \| Other methods \| 7 \| Describe any additional information sources or search methods used. \| No other methods used \| \| **SEARCH STRATEGIES** \| \| \| \| \| Full search strategies \| 8 \| Include the search strategies for each database and information source, copied and pasted exactly as run. \| Supplementary materials, Appendix 3 \| \| Limits and restrictions \| 9 \| Specify that no limits were used, or describe any limits or restrictions applied to a search (e.g., date or time period, language, study design) and provide justification for their use. \| Methods, Search strategies \| \| Search filters \| 10 \| Indicate whether published search filters were used (as originally designed or modified), and if so, cite the filter(s) used. \| No published search filters were used, mentioned in Methods, Search strategies. \| \| Prior work \| 11 \| Indicate when search strategies from other literature reviews were adapted or reused for a substantive part or all of the search, citing the previous review(s). \| Not used \| \| Updates \| 12 \| Report the methods used to update the search(es) (e.g., rerunning searches, email alerts). \| Not applicable for current review (not an update) \| \| Dates of searches \| 13 \| For each search strategy, provide the date when the last search occurred. \| Methods, Search strategies \| \| **PEER REVIEW** \| \| \| \| \| Peer review \| 14 \| Describe any search peer review process. \| Methods, Search strategies \| \| **MANAGING RECORDS** \| \| \| \| \| Total Records \| 15 \| Document the total number of records identified from each database and other information sources. \| PRISMA flowchart (Figure 1), Results, first line. \| \| Deduplication \| 16 \| Describe the processes and any software used to deduplicate records from multiple database searches and other information sources. \| Methods, study selection and Data extraction, Results, first line. \| \|  \|  \|  \|  \| \| PRISMA-S: An Extension to the PRISMA Statement for Reporting Literature Searches in Systematic Reviews \| \| \|  \| \| Rethlefsen ML, Kirtley S, Waffenschmidt S, Ayala AP, Moher D, Page MJ, Koffel JB, PRISMA-S Group. \| \| \|  \| \| Last updated February 27, 2020. \| \|  \|  \|  \|  \| \| **Appendix 3: Summary of findings table** \| \| \| \| \| \| \| \| --- \| --- \| --- \| --- \| --- \| --- \| --- \| --- \| --- \| \|  \| \| **The Effectiveness of Artificial Intelligence in Undergraduate Health Professions Education: a Systematic Review and Meta-analysis of Randomized Controlled Trials** \| \| \| \| \| \| \| \|  \| \| **Patient or population:** Undergraduate students in health sciences  **Setting:** Undergraduate health professions education  **Intervention:** AI  **Comparison:** control \| \| \| \| \| \| \| \| Outcomes \|  \| \| **Anticipated absolute effects^*^** (95% CI) \| \| Relative effect (95% CI) \| № of participants (studies) \| Certainty of the evidence (GRADE) \| Comments \| \| **Specific comparison (AI sub-categories)** \| \| **Risk with control** \| **Risk with AI** \| \| Kirkpatrick level 1: satisfaction or motivation \| LLM content generator vs control \| \| The median satisfaction or perception score was 82% \| SMD **0.65 higher** (0.73 lower to 2.04 higher) \| - \| 509 (5 RCTs) \| ⨁◯◯◯ Very low^a,b,c^ \| The evidence is very uncertain about the effect of LLM content generator on satisfaction or perception \| \| LLM personalised learning aid vs control \| \| The median satisfaction or perception score was 76% \| SMD **0.93 higher** (0.4 higher to 1.46 higher) \| - \| 430 (7 RCTs) \| ⨁◯◯◯ Very low^a,d,e^ \| The evidence is very uncertain about the effect of LLM personalised learning aid on satisfaction or perception \| \| LLM virtual patient vs control \| \| The median satisfaction or perception score was 54% \| SMD **0.69 higher** (0.83 lower to 2.21 higher) \| - \| 127 (3 RCTs) \| ⨁◯◯◯ Very low ^c,f^ \| The evidence is very uncertain about the effect of LLM virtual patient on satisfaction or perception \| \| LLM content generator + LLM virtual patient + LLM personalised learning aid vs control \| \| The median satisfaction or perception score was 56.5% \| SMD **0.02 lower** (0.44 lower to 0.40 higher) \| - \| 88 (1 RCT) \| ⨁◯◯◯ Very low ^g,h^ \| The evidence is very uncertain about the effect of a combination of LLM applications (content generator, virtual patient and personalised learning aid) on satisfaction or perception \| \| LLM-integrated curriculum vs control \| \| The median satisfaction or perception score was 55.2% \| SMD **1.31 higher** (0.87 higher to 1.76 higher) \| - \| 96 (1 RCT) \| ⨁◯◯◯ Very low ^I,j^ \| The evidence is very uncertain about the effect of LLM-integrated curriculum on satisfaction or perception \| \| non-LLM AI-moderated adaptive learning platform vs control \| \| The median satisfaction or perception score was 79% \| SMD **0.55 higher** (1.00 lower to 2.11 higher) \| - \| 143 (2 RCTs) \| ⨁◯◯◯ Very low ^k^ \| The evidence is very uncertain about the effect of non-LLM AI-moderated adaptive learning platform on satisfaction or perception \| \| NLP rule-based chatbot versus control \| \| The median satisfaction or perception score was 63.9% \| SMD **0.74 higher** (3.65 lower to 5.73 higher) \| - \| 146 (2 RCTs) \| ⨁◯◯◯ Very low^k^ \| The evidence is very uncertain about the effect of NLP rule-based chatbot on satisfaction or perception \| \| NLP rule-based chatbot + rule based virtual patient vs control \| \| The median satisfaction or perception score was 80% \| SMD **0.17 higher** (0.33 lower to 0.67 higher) \| - \| 61 (1 RCT) \| ⨁◯◯◯ Very low ^l,m^ \| The evidence is very uncertain about the effect of NLP rule-based chatbot + rule based virtual patient on satisfaction or perception \| \| NLP rule-based virtual patient vs control \| \| The median satisfaction or perception score was 82.8% \| SMD **0.75 higher** (0.29 higher to 1.20 higher) \| - \| 79 (1 RCT) \| ⨁◯◯◯ Low^n^ \| NLP rule-based virtual patient may improve student satisfaction or perception \| \| Kirkpatrick level 1: self-efficacy or confidence \| LLM personalised learning aid vs control \| \| The median self-efficacy or confidence score was 78% \| SMD **0.91 higher** (0.54 higher to 1.29 higher) \| - \| 609 (7 RCTs) \| ⨁◯◯◯ Very low^0,p,q^ \| The evidence is very uncertain about the effect of LLM personalised learning aid on self-efficacy or confidence \| \| LLM virtual patient vs control \| \| The median self-efficacy or confidence score was 54% \| SMD **1.36 higher** (8.09 lower to 10.81 higher) \| - \| 100 (2 RCTs) \| ⨁◯◯◯ Very low ^r,s,t^ \| The evidence is very uncertain about the effect of LLM virtual patient on self-efficacy or confidence \| \| LLM-integrated curriculum vs control \| \| The median self-efficacy or confidence score was 56.3% \| SMD 1.23 **higher** (0.79 higher to 1.67 higher) \| - \| 96 (1 RCT) \| ⨁◯◯◯ Very low ^I,j^ \| The evidence is very uncertain about the effect of LLM-integrated curriculum on self-efficacy or confidence \| \| non-LLM AI procedure assistant vs control \| \| The median self-efficacy or confidence score was 89% \| SMD **0.0 -no difference** (0.62 lower to 0.62 higher) \| - \| 40 (1 RCT) \| ⨁◯◯◯ Very low ^u,v^ \| The evidence is very uncertain about the effect of non-LLM AI procedure assistant on self-efficacy or confidence \| \| non-LLM AI moderated adaptive learning platform vs control \| \| The median self-efficacy or confidence indicator (no of questions asked) was 7.4 questions asked per session \| SMD **2.45 higher** (1.61 higher to 3.28 higher) \| - \| 40 (1 RCT) \| ⨁◯◯◯ Very low ^w,x^ \| The evidence is very uncertain about the effect of non-LLM AI moderated adaptive learning platform on self-efficacy or confidence \| \| NLP rule-based chatbot vs control \| \| The median self-efficacy or confidence score was 60% \| SMD **0.87 higher** (2.11 lower to 3.86 higher) \| - \| 146 (2 RCTs) \| ⨁◯◯◯ Very low^k^ \| The evidence is very uncertain about the effect of NLP rule-based chatbot on self-efficacy or confidence \| \| non-LLM AI-VR virtual doctor vs control \| \| The median self-efficacy or confidence score was 69.6% \| SMD **0.68 lower** (1.18 lower to 0.17 lower) \| - \| 64 (1 RCT) \| ⨁◯◯◯ Very low ^y,z^ \| The evidence is very uncertain about the effect of non-LLM AI-VR virtual doctor on self-efficacy or confidence \| \| AL procedure assistant + adaptive learning platform vs control \| \| The median self-efficacy or confidence score was 81.7% \| SMD **0.55 higher** (0.34 lower to 1.45 higher) \| - \| 20 (1 RCTs) \| ⨁◯◯◯ Very low ^y,aa^ \| The evidence is very uncertain about the effect of AL procedure assistant + adaptive learning platform on self-efficacy or confidence \| \|  \| AI procedure assistant (non-LLM) vs control (proportion of participants confident in echocardiography view) \| \| The proportion in the control group was 22.7% \|  \| **RR 1.26**  (0.45 – 3.50) \| 43 (1 RCTs) \| ⨁◯◯◯ Very low ^ab,ac^ \| The evidence is very uncertain about the effect of AL procedure assistant on the proportion of participants who were confident on echocardiography view. \| \| Kirkpatrick level 2: theoretical knowledge score \| LLM content generator vs control \| \| The median theoretical knowledge score was 60.0% \| SMD **0.99 higher** (1.04 lower to 3.01 higher) \| - \| 357 (3 RCTs) \| ⨁◯◯◯ Very low ^ad,ae,af^ \| The evidence is very uncertain about the effect of LLM content generator on theoretical knowledge \| \| LLM gamification tool vs control \| \| The median theoretical knowledge score was 91.2% \| SMD **0.79 higher** (0.20 higher to 1.38 higher) \| - \| 48 (1 RCT) \| ⨁◯◯◯ Very low ^y, ag^ \| The evidence is very uncertain about the effect of LLM gamification tool on theoretical knowledge \| \| LLM personalised learning aid vs control \| \| The median theoretical knowledge score was 65.7% \| SMD **0.53 higher** (0.13 higher to 0.94 higher) \| - \| 955 (12 RCTs) \| ⨁◯◯◯ Very low ^ah,ai,aj^ \| The evidence is very uncertain about the effect of LLM personalised learning aid on theoretical knowledge \| \| non-LLM AI moderated adaptive learning platform vs control \| \| The median theoretical knowledge score was 81.7% \| SMD **0.68 higher** (0.04 higher to 1.32 higher) \| - \| 40 (1 RCT) \| ⨁◯◯◯ Very low ^w,aj^ \| The evidence is very uncertain about the effect of non-LLM AI moderated adaptive learning platform on theoretical knowledge \| \| NLP rule-based chatbot vs control \| \| The median theoretical knowledge score was 61.5% \| SMD **1.06 higher** (2.19 lower to 4.32 higher) \| - \| 530 (3 RCTs) \| ⨁◯◯◯ Very low ^ak,al,am^ \| The evidence is very uncertain about the effect of NLP rule-based chatbot on theoretical knowledge \| \| non-LLM AI imaging diagnostic aid vs control \| \| The median theoretical knowledge score was 40.6% \| SMD **1.26 higher** (6.23 lower to 8.74 higher) \| - \| 69 (2 RCTs) \| ⨁◯◯◯ Very low ^an,ao,ap^ \| The evidence is very uncertain about the effect of non-LLM AI imaging diagnostic aid on theoretical knowledge \| \| non-LLM AI-VR virtual doctor vs control \| \| The median theoretical knowledge score was unknown (total scale not stated in the single included study) \| SMD **0.67 higher** (0.16 higher to 1.17 higher) \| - \| 64 (1 RCT) \| ⨁◯◯◯ Very low ^y,aj^ \| The evidence is very uncertain about the effect of non-LLM AI-VR virtual doctor on theoretical knowledge \| \| Proportion with grade A or B: non-LLM AI gamification tool vs control \| \| The proportion in the control group was 64.9% \|  \| **RR 1.33**  (1.01 – 1.74) \| 73 (1 RCT) \| ⨁◯◯◯ Very low ^aj,aq^ \| The evidence is very uncertain about the effect of non-LLM AI gamification tool on the proportion of participants who achieved the desired grade. \| \| Kirkpatrick level 2: clinical skills \| LLM content generator vs control \| \| The median clinical skills score was 64.2% \| SMD **0.52 higher** (8.66 lower to 9.69 higher) \| - \| 295 (2 RCTs) \| ⨁◯◯◯ Very low ^ar,as,at^ \| The evidence is very uncertain about the effect of LLM content generator on clinical skills. \| \| LLM personalised learning aid vs control \| \| The median clinical skills score was 57.0% \| SMD **0.49 higher** (0.00 to 0.97 higher) \| - \| 609 (9 RCTs) \| ⨁◯◯◯ Very low ^au,av, aw^ \| The evidence is very uncertain about the effect of LLM personalised learning aid on clinical skills. \| \| LLM virtual patient vs control \| \| The median clinical skills score was 73.6% \| SMD **2.53 higher** (1.82 higher to 3.25 higher) \| - \| 56 (1 RCT) \| ⨁⨁◯◯ Low ^ax,ay^ \| LLM virtual patient may improve clinical skills. \| \| LLM content generator + LLM virtual patient + LLM personalised learning aid vs control \| \| The median clinical skills score was 45.1% \| SMD **0.11 lower** (0.53 lower to 0.31 higher) \| - \| 88 (1 RCT) \| ⨁◯◯◯ Very low ^az,ba^ \| The evidence is very uncertain about the effect of LLM content generator + LLM virtual patient + LLM personalised learning aid on clinical skills. \| \| LLM virtual patient + LLM personalised learning aid vs control \| \| The median clinical skills score was 55.4% \| SMD 1**.82 higher** (3.63 lower to 7.27 higher) \| - \| 124 (3 RCTs) \| ⨁◯◯◯ Very low ^bb,bc,bd^ \| The evidence is very uncertain about the effect of LLM virtual patient + LLM personalised learning aid vs control on clinical skills. \| \| non-LLM AI imaging diagnostic aid vs control \| \| The mean clinical skills score was 78.5% \| SMD 0.43 **higher** (0.41 lower to 1.27 higher) \| - \| 176 (4 RCTs) \| ⨁◯◯◯ Very low ^bb,be,bf^ \| The evidence is very uncertain about the effect of non-LLM AI imaging diagnostic aid on clinical skills. \| \| non-LLM AI moderated adaptive learning platform vs control \| \| The mean clinical skills score was 64.4% \| SMD **0.59 higher** (19.98 lower to 21.17 higher) \| - \| 139 (2 RCTs) \| ⨁◯◯◯ Very low ^bg,bh,bi^ \| The evidence is very uncertain about the effect of non-LLM AI moderated adaptive learning platform on clinical skills. \| \| NLP rule-based virtual patient vs control \| \| The mean clinical skills score was 74.1% \| SMD **2.03 higher** (1.49 higher to 2.58 higher) \| - \| 79 (1 RCT) \| ⨁⨁⨁◯ Moderate^ay^ \| NLP rule-based virtual patient probably improves clinical skills. \| \| NLP rule-based chatbot+ virtual patient vs control \| \| The mean clinical skills score was 68.1% \| SMD **0.24 higher** (0.26 lower to 0.74 higher) \| - \| 61 (1 RCT) \| ⨁◯◯◯ Very low ^bj,bk^ \| The evidence is very uncertain about the effect of NLP rule-based chatbot+ virtual patient on clinical skills. \| \| AI-VR virtual doctor vs control \| \| The mean clinical skills score was 70.8% \| SMD **0.21 higher** (0.28 lower to 0.71 higher) \| - \| 64 (1 RCT) \| ⨁◯◯◯ Very low ^y,bk^ \| The evidence is very uncertain about the effect of AI-VR virtual doctor on clinical skills. \| \| AI procedure assistant + AI-moderated adaptive learning platform vs control \| \| The mean clinical skills score was 70.6% \| SMD **0.80 higher** (0.12 lower to 1.72 higher) \| - \| 20 (1 RCT) \| ⨁◯◯◯ Very low ^bl,bm^ \| The evidence is very uncertain about the effect of AI procedure assistant + AI-moderated adaptive learning platform on clinical skills. \| \| Kirkpatrick level 2: practical skills \| LLM personalised learning aid vs control \| \| The median practical skills score was 65.5% \| SMD **0.67 higher** (0.37 higher to 0.96 higher) \| - \| 187 (1 RCT) \| ⨁⨁⨁◯ Moderate ^bn^ \| LLM personalised learning aid probably improves practical skills. \| \| non-LLM AI procedure assistant vs control \| \| The median practical skills score was 67% \| SMD **0.18 higher** (0.97 lower to 1.34 higher) \| - \| 309 (6 RCTs) \| ⨁◯◯◯ Very low ^bo,bp,bq^ \| The evidence is very uncertain about the effect of non-LLM AI procedure assistant on practical skills. \| \| Kirkpatrick level 2: Task efficiency (time taken to perform tasks) \| LLM personalised learning aid vs control \| \| The range of time taken to perform tasks in the studies included ranged from 2.1 to 8.1 minutes \| SMD **0.15 lower** (4.24 lower to 3.95 higher) \| - \| 100 (2 RCTs) \| ⨁◯◯◯ Very low ^br,bs^ \| The evidence is very uncertain about the effect of LLM personalised learning aid on task efficiency (time taken to perform tasks). \| \| non-LLM AI imaging diagnostic aid vs control \| \| The time taken to perform tasks in the study included was 7.3 minutes \| SMD **2.70 higher** (1.82 higher to 3.58 higher) \| - \| 40 (1 RCT) \| ⨁◯◯◯ Very low ^bt,bu^ \| The evidence is very uncertain about the effect of non-LLM AI imaging diagnostic aid on task efficiency (time taken to perform tasks). \| \| non-LLM AI procedure assistant vs control \| \| The range of time taken to perform tasks in the studies included ranged from 2.25 to 2.63 minutes \| SMD **1.26 lower** (14.56 lower to 12.12 higher) \| - \| 52 (2 RCTs) \| ⨁◯◯◯ Very low^bv,bw,bx^ \| The evidence is very uncertain about the effect of non-LLM AI procedure assistant on task efficiency (time taken to perform tasks). \| \| non-LLM AI procedure assistant + AI-moderated adaptive learning platform vs control \| \| The range of time taken to perform tasks in the study included was 0.36 minutes \| SMD **0.87 lower** (1.80 lower to 0.05 higher) \| - \| 20 (1 RCT) \| ⨁◯◯◯ Very low^by,bz^ \| The evidence is very uncertain about the effect of non-LLM AI procedure assistant + AI-moderated adaptive learning platform on task efficiency (time taken to perform tasks). \| \| Kirkpatrick level 2: Generic or personal skills \| LLM personalised learning aid vs control \| \| The median generic or personal skills score was 58% \| SMD **0.45 higher** (0.05 higher to 0.84 higher) \| - \| 101 (1 RCT) \| ⨁◯◯◯ Very low ^ca,,cb^ \| The evidence is very uncertain about the effect of LLM personalised learning aid on generic or personal skills. \| \| LLM virtual patient vs control \| \| The median generic or personal skills score was 73% \| SMD **0.00 no difference** (1.06 lower to 1.06 higher) \| - \| 27 (1 RCT) \| ⨁◯◯◯ Very low ^cc,,cd^ \| The evidence is very uncertain about the effect of LLM virtual patient on generic or personal skills. \| \| LLM-integrated curriculum vs control \| \| The median generic or personal skills score was 47.2% \| SMD **0.60 higher** (0.19 higher to 1.01 higher) \| - \| 96 (1 RCT) \| ⨁⨁⨁◯ Moderate ^ce^ \| LLM-integrated curriculum probably improve generic or personal skills. \| \| non-LLM AI communication analysis vs control \| \| The median generic or personal skills score was not available (total scale range not reported) \| SMD **1.85 higher** (0.88 higher to 2.81 higher) \| - \| 25 (1 RCT) \| ⨁◯◯◯ Very low ^cf,,cg^ \| The evidence is very uncertain about the effect of non-LLM AI communication analysis on generic or personal skills. \| \| AI-VR virtual doctor vs control \| \| The median generic or personal skills score was not available (total scale range not reported) \| SMD **0.31 higher** (0.18 lower to 0.81 higher) \| - \| 64 (1 RCT) \| ⨁◯◯◯ Very low ^y,,ch^ \| The evidence is very uncertain about the effect of AI-VR virtual doctor on generic or personal skills. \| \|  \| \| ***The risk in the intervention group** (and its 95% confidence interval) is based on the assumed risk in the comparison group and the **relative effect** of the intervention (and its 95% CI).  **CI:** confidence interval; **RR:** risk ratio; **SMD:** standardised mean difference \| \| \| \| \| \| \| \|  \| \| **GRADE Working Group grades of evidence** **High certainty:** we are very confident that the true effect lies close to that of the estimate of the effect. **Moderate certainty:** we are moderately confident in the effect estimate: the true effect is likely to be close to the estimate of the effect, but there is a possibility that it is substantially different. **Low certainty:** our confidence in the effect estimate is limited: the true effect may be substantially different from the estimate of the effect. **Very low certainty:** we have very little confidence in the effect estimate: the true effect is likely to be substantially different from the estimate of effect. \| \| \| \| \| \| \| |
| --- | --- | --- | --- | --- | --- | --- | --- | --- | --- | --- | --- | --- | --- | --- | --- | --- | --- | --- | --- | --- | --- | --- | --- | --- | --- | --- | --- | --- | --- | --- | --- | --- | --- | --- | --- | --- | --- | --- | --- | --- | --- | --- | --- | --- | --- | --- | --- | --- | --- | --- | --- | --- | --- | --- | --- | --- | --- | --- | --- | --- | --- | --- | --- | --- | --- | --- | --- | --- | --- | --- | --- | --- | --- | --- | --- | --- | --- | --- | --- | --- | --- | --- | --- | --- | --- | --- | --- | --- | --- | --- | --- | --- | --- | --- | --- | --- | --- | --- | --- | --- | --- | --- | --- | --- | --- | --- | --- | --- | --- | --- | --- | --- | --- | --- | --- | --- | --- | --- | --- | --- | --- | --- | --- | --- | --- | --- | --- | --- | --- | --- | --- | --- | --- | --- | --- | --- | --- | --- | --- | --- | --- | --- | --- | --- | --- | --- | --- | --- | --- | --- | --- | --- | --- | --- | --- | --- | --- | --- | --- | --- | --- | --- | --- | --- | --- | --- | --- | --- | --- | --- | --- | --- | --- | --- | --- | --- | --- | --- | --- | --- | --- | --- | --- | --- | --- | --- | --- | --- | --- | --- | --- | --- | --- | --- | --- | --- | --- | --- | --- | --- | --- | --- | --- | --- | --- | --- | --- | --- | --- | --- | --- | --- | --- | --- | --- | --- | --- | --- | --- | --- | --- | --- | --- | --- | --- | --- | --- | --- | --- | --- | --- | --- | --- | --- | --- | --- | --- | --- | --- | --- | --- | --- | --- | --- | --- | --- | --- | --- | --- | --- | --- | --- | --- | --- | --- | --- | --- | --- | --- | --- | --- | --- | --- | --- | --- | --- | --- | --- | --- | --- | --- | --- | --- | --- | --- | --- | --- | --- | --- | --- | --- | --- | --- | --- | --- | --- | --- | --- | --- | --- | --- | --- | --- | --- | --- | --- | --- | --- | --- | --- | --- | --- | --- | --- | --- | --- | --- | --- | --- | --- | --- | --- | --- | --- | --- | --- | --- | --- | --- | --- | --- | --- | --- | --- | --- | --- | --- | --- | --- | --- | --- | --- | --- | --- | --- | --- | --- | --- | --- | --- | --- | --- | --- | --- | --- | --- | --- | --- | --- | --- | --- | --- | --- | --- | --- | --- | --- | --- | --- | --- | --- | --- | --- | --- | --- | --- | --- | --- | --- | --- | --- | --- | --- | --- | --- | --- | --- | --- | --- | --- | --- | --- | --- | --- | --- | --- | --- | --- | --- | --- | --- | --- | --- | --- | --- | --- | --- | --- | --- | --- | --- | --- | --- | --- | --- | --- | --- | --- | --- | --- | --- | --- | --- | --- | --- | --- | --- | --- | --- | --- | --- | --- | --- | --- | --- | --- | --- | --- | --- | --- | --- | --- | --- | --- | --- | --- | --- | --- | --- | --- | --- | --- | --- | --- | --- | --- | --- | --- | --- | --- | --- | --- | --- | --- | --- | --- | --- | --- | --- | --- | --- | --- | --- | --- | --- | --- | --- | --- | --- | --- | --- | --- | --- | --- | --- | --- | --- | --- | --- | --- | --- | --- | --- | --- | --- | --- | --- | --- | --- | --- | --- | --- | --- | --- | --- | --- | --- | --- | --- | --- | --- | --- | --- | --- | --- | --- | --- | --- | --- | --- | --- | --- | --- | --- | --- | --- | --- | --- | --- | --- | --- | --- | --- | --- | --- | --- | --- | --- | --- | --- | --- | --- | --- | --- | --- | --- | --- | --- | --- | --- | --- | --- | --- | --- | --- | --- | --- | --- | --- | --- |

#### **Explanations**

a. The majority of the included studies had at least probable high risk of bias in randomisation and allocation, blinding of participants and outcome assessors for the outcomes evaluated. Certainty downgraded by one level.

b. There is a great degree of heterogeneity as indicated by the very high I^2^ value (96%), which is contributed equally by all included studies (i.e. no single chief contributing study), and remains unexplained after exploration of population, category of intervention and comparison. Certainty downgraded by two levels for very serious inconsistency between studies.

c. For the target effect of an important increase in self-perceived ratings as suggested by the point estimate (which indicates a moderate effect size), the 95% CI ranges from a moderate decrease to a large increase in effect size. Certainty downgraded by two levels for very serious imprecision.

d. There is a substantial degree of heterogeneity as indicated by the high I^2^ value (74%), which is contributed equally by all included studies (i.e. no single chief contributing study), and remains unexplained after exploration of population, category of intervention and comparison. Certainty downgraded by one level for serious inconsistency between studies.

e. For the target effect of an important increase in self-perceived ratings as suggested by the point estimate (which indicates a large effect size), the 95% CI ranges from a moderate to a large increase in effect size. Certainty downgraded by one level for serious imprecision.

f. There is a substantial degree of heterogeneity as indicated by the high I^2^ value (64%), which is contributed chiefly by one study (Wang 2025a), but no convincing reasons for the difference between this and the other two studies were identified, either in terms of field (all differed), comparison and single versus multiple sessions. Certainty downgraded by one level for serious unexplained inconsistency between studies.

g. The single included study has high or probable high risks of bias in allocation concealment, blinding of participants and outcome assessors. Certainty downgraded by one level for serious risk of bias concerns.

h. For the target effect of little to no effect as suggested by the point estimate, the 95% CI ranges from a moderate reduction to a moderate increase in effect size. Certainty downgraded by two levels for very serious imprecision

i. The single included study has high or probable high risks of bias in allocation concealment, blinding of participants, outcome assessors and missing outcome data. Certainty downgraded by two levels for very serious risk of bias concerns.

j. For the target effect of a large effect size as suggested by the point estimate, the 95% CI ranges from a moderate to a large increase in effect size. Certainty downgraded by one level for serious imprecision

k. For the target effect of a moderate increase in self-perceived ratings as suggested by the point estimate (which indicates a moderate effect size), the 95% CI ranges from a large decrease to a large increase in effect size. Certainty downgraded by three levels for extremely serious imprecision.

l. The single included study has high/probable high risk of bias in allocation concealment and blinding of participants. Certainty downgraded by one level for serious risk of bias concerns.

m. For the target effect of a small increase in self-perceived ratings as suggested by the point estimate (which indicates a small effect size), the 95% CI ranges from a moderate decrease to a moderate increase in effect size. Certainty downgraded by two levels for very serious imprecision.

n. For the target effect of a moderate increase in self-perceived ratings as suggested by the point estimate (which indicates a moderate effect size), the 95% CI ranges from a small increase to a large increase in effect size. Certainty downgraded by two levels for very serious imprecision.

o. Most included studies had high/probably high risks of bias in random sequence generation and allocation concealment, blinding of educational providers and outcome assessors, and all studies had high risks of bias in blinding of participants. Certainty downgraded by two levels for very serious risk of bias concerns.

p. There is a substantial degree of heterogeneity as indicated by the high I^2^ value (64%), which is contributed equally by all included studies (i.e. no single chief contributing study), and remains unexplained after exploration of population, category of intervention and comparison. Certainty downgraded by one level for serious inconsistency between studies.

q. For the target effect of a moderate increase in self-efficacy ratings as suggested by the point estimate (which indicates a large effect size), the 95% CI ranges from a moderate increase to a large increase in effect size. Certainty downgraded by one level for serious imprecision.

r. Both studies had high risks of bias in blinding of participants and educational providers, and one at high risk in blinding of outcome assessors. Certainty downgraded one level for serious risk of bias concerns.

s. There is a very high degree of heterogeneity as indicated by the high I^2^ value (91%), which is contributed equally by both included studies, and remains unexplained after exploration of population, category of intervention and comparison. Certainty downgraded by two levels for very serious inconsistency between studies.

t. For the target effect of a large increase in self-efficacy ratings as suggested by the point estimate (which indicates a large effect size), the 95% CI ranges from a very large decrease to a very large increase in effect size. Certainty downgraded by three levels for extremely serious imprecision.

u. The single included study had high risks of bias in most domains including random sequence generation and allocation concealment. Certainty downgraded two levels for very serious risk of bias concerns.

v. For the target effect of little to no difference self-efficacy ratings as suggested by the point estimate (which indicates no effect), the 95% CI ranges from a moderate decrease to a moderate increase in effect size. Certainty downgraded by two levels for very serious imprecision.

w. The single included study had high risks of bias in blinding of participants and educational providers. Certainty downgraded by one level for serious risk of bias concerns.

x. For the target effect of a very large increase in self-efficacy ratings as suggested by the point estimate (which indicates a very large effect size), the 95% CI ranges from a large to a very large increase in effect size. Certainty downgraded by two levels for very serious imprecision.

y. The single included study had high risks of bias in allocation concealment, blinding of participants, educational providers and outcome assessors. Certainty downgraded by two levels for very serious risk of bias concerns.

z. For the target effect of a moderate decrease in self-efficacy ratings as suggested by the point estimate (which indicates a moderate effect size), the 95% CI ranges from a large to a small decrease in effect size. Certainty downgraded by two levels for very serious imprecision.

aa. For the target effect of a moderate increase in self-efficacy ratings as suggested by the point estimate (which indicates a moderate effect size), the 95% CI ranges from a moderate decrease to a large increase in effect size. Certainty downgraded by two levels for very serious imprecision.

ab. The single included study had high risks of bias in blinding of participants, educational providers and outcome assessors. Certainty downgraded by one level for serious risk of bias concerns.

ac. For the target effect of a small increase in the proportion who were confident, as suggested by the point estimate (which indicates a small effect size), the 95% CI ranges from a large decrease to a large increase in effect size. Certainty downgraded by two levels for very serious imprecision.

ad. All included studies had high risks of bias in random sequence generation and/or allocation concealment, blinding of participant and educational providers. Certainty downgraded for two levels for very serious imprecision.

ae. There is a very high degree of heterogeneity as indicated by the high I^2^ value (93%), which is contributed equally by all included studies (i.e. no single chief contributor), and remains unexplained after exploration of population, category of intervention and comparison. Certainty downgraded by two levels for very serious inconsistency between studies.

af. For the target effect of a large increase in knowledge score as suggested by the point estimate (which indicates a large effect size), the 95% CI ranges from a large decrease to a very large increase in effect size. Certainty downgraded by two levels for very serious imprecision.

ag. For the target effect of a moderate increase in knowledge score as suggested by the point estimate (which indicates a moderate effect size), the 95% CI ranges from a small increase to a large increase in effect size. Certainty downgraded by two levels for very serious imprecision.

ah. Most studies had high risks of bias in allocation concealment and blinding of participants. Certainty downgraded by one level for serious risk of bias concerns.

ai. There is a very high degree of heterogeneity as indicated by the high I^2^ value (86%), which is contributed equally by all included studies (i.e. no single chief contributor), and remains unexplained after exploration of population, category of intervention and comparison. Certainty downgraded by two levels for very serious inconsistency between studies.

aj. For the target effect of a moderate increase in knowledge score as suggested by the point estimate (which indicates a moderate effect size), the 95% CI ranges from a small increase to a large increase in effect size. Certainty downgraded by two levels for very serious imprecision.

ak. One study had high risk of bias in sequence generation and allocation concealment, and two out of three studies had high risks in blinding of participants and educational providers. Certainty downgraded one level for serious risk of bias concerns.

al. There is a very high degree of heterogeneity as indicated by the high I^2^ value (97%), which is chiefly contributed by one study (Hsu 2024). However, no distinctive characteristics including field, purpose (teaching-learning or assessment), control group characteristics or frequency of intervention that separated Hsu and the other two studies. The heterogeneity thus remains unexplained after exploration of population, category of intervention and comparison. Certainty downgraded by two levels for very serious inconsistency between studies.

am. For the target effect of a large increase in knowledge score as suggested by the point estimate (which indicates a large effect size), the 95% CI ranges from a large decrease to a very large increase in effect size. Certainty downgraded by two levels for very serious imprecision.

an. One study had high risk of bias in sequence generation and allocation concealment, and both studies had high risks in blinding of participants and educational providers. Certainty downgraded one level for serious risk of bias concerns.

ao. There is a substantial degree of heterogeneity as indicated by the high I^2^ value (78%), which remained unexplained after exploration of population, category of intervention and comparison. Certainty downgraded by one level for serious inconsistency between studies.

ap. For the target effect of a large increase in knowledge score as suggested by the point estimate (which indicates a large effect size), the 95% CI ranges from a very large decrease to a very large increase in effect size. Certainty downgraded by three levels for extremely serious imprecision.

aq. The single included study had high risks of bias in random sequence generation, allocation concealment and blinding of the participants. Certainty downgraded by two levels for very serious risk of bias concerns.

ar. Both studies had high risks of bias in allocation concealment, and one study had high risks of bias in blinding of participants, educational providers and outcome assessors. Certainty downgraded one level for serious risk of bias concerns.

as. There is a very high degree of heterogeneity as indicated by the high I^2^ value (97%), which remained unexplained after exploration of population, category of intervention and comparison. Certainty downgraded by two levels for very serious inconsistency between studies.

at. For the target effect of a moderate increase in clinical skill score as suggested by the point estimate (which indicates a moderate effect size), the 95% CI ranges from a very large decrease to a very large increase in effect size. Certainty downgraded by three levels for extremely serious imprecision.

au. The majority of the included studies had high risks of bias in allocation concealment, blinding of the participants, educational providers and outcome assessors. Certainty downgraded by two levels for very serious risk of bias concerns.

av. There is a very high degree of heterogeneity as indicated by the high I^2^ value (83%), which remained unexplained after exploration of population, category of intervention and comparison. Certainty downgraded by two levels for very serious inconsistency between studies.

aw. For the target effect of a moderate increase in clinical skill score as suggested by the point estimate (which indicates a moderate effect size), the 95% CI ranges from no difference to a large increase in effect size. Certainty downgraded by two levels for very serious imprecision.

ax. The single included study had high risks of bias in blinding of the participants and educational providers. Certainty downgraded by one level for serious risk of bias concerns.

ay. For the target effect of a large increase in clinical skill score as suggested by the point estimate (which indicates a large effect size), the 95% CI ranges from a large increase to a very large increase in effect size. Certainty downgraded by one level for serious imprecision.

az. The single included study had high risks of bias in allocation concealment and blinding of the participants. Certainty downgraded by one level for serious risk of bias concerns.

ba. For the target effect of a small increase in clinical skill score as suggested by the point estimate (which indicates a small effect size), the 95% CI ranges from a moderate decrease to a small-moderate increase in effect size. Certainty downgraded by two levels for very serious imprecision.

bb. Majority of the included studies had high risks of bias in allocation concealment, and all had high risks of bias in blinding of the participants and educational providers. Certainty downgraded by two levels for very serious risk of bias concerns.

bc. There is a very high degree of heterogeneity as indicated by the high I^2^ value (95%), which remained unexplained after exploration of population, category of intervention and comparison. Certainty downgraded by two levels for very serious inconsistency between studies.

bd. For the target effect of a large increase in clinical skill score as suggested by the point estimate (which indicates a large effect size), the 95% CI ranges from a very large decrease to a very large increase in effect size. Certainty downgraded by three levels for extremely serious imprecision.

be. There is a substantial degree of heterogeneity as indicated by the high I^2^ value (62%), which remained unexplained after exploration of population, category of intervention and comparison. Certainty downgraded by one level for serious inconsistency between studies.

bf. For the target effect of a moderate increase in clinical skill score as suggested by the point estimate (which indicates a moderate effect size), the 95% CI ranges from a moderate decrease to a large increase in effect size. Certainty downgraded by two levels for very serious imprecision.

bg. Both included studies had high risks of bias in allocation concealment, blinding of the participants and educational providers. Certainty downgraded by two levels for very serious risk of bias concerns.

bh. There is a very high degree of heterogeneity as indicated by the high I^2^ value (98%), which remained unexplained after exploration of population, category of intervention and comparison. Certainty downgraded by two levels for very serious inconsistency between studies.

bi. For the target effect of a moderate increase in clinical skill score as suggested by the point estimate (which indicates a moderate effect size), the 95% CI ranges from a very large decrease to a very large increase in effect size. Certainty downgraded by three levels for extremely serious imprecision.

bj. The single included study had high risks of bias in allocation concealment, blinding of the participants, educational providers and outcome assessors. Certainty downgraded by two levels for very serious risk of bias concerns.

bk. For the target effect of a small increase in clinical skill score as suggested by the point estimate (which indicates a small effect size), the 95% CI ranges from a small decrease to a moderate-large increase in effect size. Certainty downgraded by two levels for very serious imprecision.

bl. The single included study had high risks of bias in allocation concealment, blinding of the participants and educational providers. Certainty downgraded by two levels for very serious risk of bias concerns.

bm. For the target effect of a large increase in clinical skill score as suggested by the point estimate (which indicates a large effect size), the 95% CI ranges from a small decrease to a large increase in effect size. Certainty downgraded by two levels for very serious imprecision.

bn. For the target effect of a moderate increase in practical skill score as suggested by the point estimate (which indicates a moderate effect size), the 95% CI ranges from a small increase to a large increase in effect size. Certainty downgraded by one level for serious imprecision.

bo. Most of the included studies had high risks of bias in allocation concealment, blinding of the participants and educational providers. Certainty downgraded by two levels for very serious risk of bias concerns.

bp. There is a very high degree of heterogeneity as indicated by the high I^2^ value (92%), which remained unexplained after exploration of population, category of intervention and comparison. Certainty downgraded by two levels for very serious inconsistency between studies.

bq. For the target effect of a small increase in practical skill score as suggested by the point estimate (which indicates a small effect size), the 95% CI ranges from a large decrease to a large increase in effect size. Certainty downgraded by three levels for extremely serious imprecision.

br. One of the two included studies had high risks of bias in allocation concealment, blinding of the participants and educational providers. Certainty downgraded by one level for serious risk of bias concerns.

bs. For the target effect of a small decrease in time needed to complete tasks as suggested by the point estimate (which indicates a small effect size), the 95% CI ranges from a large decrease to a large increase in effect size. Certainty downgraded by three levels for extremely serious imprecision.

bt. The single included study had high risks of bias in random sequence generation, allocation concealment, blinding of the participants and educational providers. Certainty downgraded by two levels for very serious risk of bias concerns.

bu. For the target effect of a large increase in time needed to complete tasks as suggested by the point estimate (which indicates a large effect size), the 95% CI ranges from a large increase to a very large increase in effect size. Certainty downgraded by one level for serious imprecision.

bv. One of the two included studies had high risks of bias in random sequence generation and allocation concealment, and both studies had high risks of bias in blinding of the participants and educational providers. Certainty downgraded by two levels for very serious risk of bias concerns.

bw. There is a very high degree of heterogeneity as indicated by the high I^2^ value (88%), which remained unexplained after exploration of population, category of intervention and comparison. Certainty downgraded by two levels for very serious inconsistency between studies.

bx. For the target effect of a large decrease in time needed to complete tasks as suggested by the point estimate (which indicates a large effect size), the 95% CI ranges from a very large decrease to a very large increase in effect size. Certainty downgraded by three levels for extremely serious imprecision.

by. The single included study had high risks of bias in allocation concealment, blinding of the participants and educational providers. Certainty downgraded by two levels for very serious risk of bias concerns.

bz. For the target effect of a large decrease in time needed to complete tasks as suggested by the point estimate (which indicates a large effect size), the 95% CI ranges from a very large decrease to a small increase in effect size. Certainty downgraded by two levels for very serious imprecision.

ca. The single included study had high risks of bias in allocation concealment, blinding of the participants and educational providers. Certainty downgraded by two levels for very serious risk of bias concerns.

cb. For the target effect of a moderate increase in generic/personal skills score as suggested by the point estimate (which indicates a moderate effect size), the 95% CI ranges from a small increase to a large increase in effect size. Certainty downgraded by one for serious imprecision.

cc. The single included study had high risks of bias in most domains including random sequence generation, allocation concealment, blinding of the participants and educational providers. Certainty downgraded by two levels for very serious risk of bias concerns.

cd. For the target effect of little to no effect in generic/personal skills score as suggested by the point estimate (which indicates a null effect size), the 95% CI ranges from a large decrease to a large increase in effect size. Certainty downgraded by three levels for extremely serious imprecision.

ce. For the target effect of a moderate increase in generic/personal skills score as suggested by the point estimate (which indicates a moderate effect size), the 95% CI ranges from a small increase to a large increase in effect size. Certainty downgraded by one level for serious imprecision.

cf. The single included study had high risks of bias in random sequence generation, allocation concealment and blinding of the participants. Certainty downgraded by two levels for very serious risk of bias concerns.

cg. For the target effect of a large increase in generic/personal skills score as suggested by the point estimate (which indicates a large effect size), the 95% CI ranges from a large increase to a very large increase in effect size. Certainty downgraded by one level for serious imprecision.

ch. For the target effect of a moderate increase in generic/personal skills score as suggested by the point estimate (which indicates a moderate effect size), the 95% CI ranges from a small decrease to a large increase in effect size. Certainty downgraded by two levels for very serious imprecision.

# **Appendix 4. List of shortlisted studies that were excluded**

1. Abdel Fatah NNA, Mohamed Bakr AS, Mahmoud Shaaban HA, Ashry SK, Abdel-Hamid Elzahry MAA. Evaluation of Artificial Intelligence as a Tool for Assessing Presentation Skills Among First-Year Medical Students at Ain Shams University. QJM: An International Journal of Medicine. 2025 01 Nov;118(Supplement 1):i65. PMID: 649439373.
2. Abuadas M, Albikawi Z, Rayani A. The impact of an AI-focused ethics education program on nursing students' ethical awareness, moral sensitivity, attitudes, and generative AI adoption intention: a quasi-experimental study. BMC nursing. 2025;24(1):720. PMID: CN-02881094. doi: 10.1186/s12912-025-03458-2.
3. Afranie-Sakyi JA, Restrepo V, Van Doren M, Martin K, Guru A, Van Doren LN. Leveraging a Large Language Learning Model to Improve Health Equity Content in the First-Year Medical School Classical Hematology Curriculum. Blood. 2024 05 Nov;144(Supplement 1):7953. PMID: 2039406443.
4. Aldukhail S. Mapping the Landscape of Generative Language Models in Dental Education: A Comparison Between ChatGPT and Google Bard. European journal of dental education : official journal of the Association for Dental Education in Europe. 2025 Feb;29(1):136-48. PMID: 39563479. doi: 10.1111/eje.13056.
5. Ali R, Tang OY, Connolly ID, Zadnik Sullivan PL, Shin JH, Fridley JS, et al. Performance of ChatGPT and GPT-4 on Neurosurgery Written Board Examinations. medRxiv. 2023;29. PMID: 2023737793.
6. Al-Karad D, Bitar E, Kattan A, Nasr M, Sahilieh H, AlKrad L, et al. Charting the Future of Otolaryngology Education: from Text to Vision with Intelligent Tutoring Systems - a Pilot RCT Study in Damascus Hospitals. British journal of surgery. 2025;112:x150. PMID: CN-02884552. doi: 10.1093/bjs/znaf128.601.
7. Allan AY, Chasapi M, Kapila A, Iyer H, Chowdhury R, Erotocritou M, et al. Three-dimensional microscope skill acquisition: A randomised controlled study comparing two-dimensional laboratory microscope training, video gaming and virtual reality gaming. Journal of Plastic, Reconstructive and Aesthetic Surgery. 2024 01 Nov;98:373-80. PMID: 2034774321.
8. Almaghaslah D. Challenging the curve: can ChatGPT-generated MCQs reduce grade inflation in pharmacy education. Frontiers in Pharmacology. 2025;16(no pagination)(1516381). PMID: 2033203986.
9. Alnabelsi T, Al-Hussaini A, Owens D. Comparison of traditional face-to-face teaching with synchronous e-learning in otolaryngology emergencies teaching to medical undergraduates: a randomised controlled trial. European archives of oto-rhino-laryngology. 2015;272(3):759‐63. PMID: CN-01043475. doi: 10.1007/s00405-014-3326-6.
10. Alsolami AS. The effectiveness of using artificial intelligence in improving academic skills of school-aged students with mild intellectual disabilities in Saudi Arabia. Research in developmental disabilities. 2025;156:104884. PMID: CN-02795607. doi: 10.1016/j.ridd.2024.104884.
11. Al-Thani SN, Anjum S, Bhutta ZA, Bashir S, Majeed MA, Khan AS, et al. Comparative performance of ChatGPT, Gemini, and final-year emergency medicine clerkship students in answering multiple-choice questions: implications for the use of AI in medical education. International Journal of Emergency Medicine. 2025 01 Dec;18(1) (no pagination)(146). PMID: 2035640499.
12. Anonymous. Evaluation of Nursing Students' Crisis Management Skills in Medication Safety Simulation: A Randomized Controlled Study. clinicaltrialsgov. 2024;02. PMID: NCT06359405.
13. Arain SA, Akhund SA, Barakzai MA, Meo SA. Transforming medical education: leveraging large language models to enhance PBL-a proof-of-concept study. Advances in Physiology Education. 2025 01 Jun;49(2):398-404. PMID: 646542029.
14. Araji T, Brooks AD. Evaluating The Role of ChatGPT as a Study Aid in Medical Education in Surgery. Journal of surgical education. 2024 01 May;81(5):753-7. PMID: 2031377655.
15. Arun G, Perumal V, Urias F, Ler YE, Tan BWT, Vallabhajosyula R, et al. ChatGPT versus a customized AI chatbot (Anatbuddy) for anatomy education: A comparative pilot study. Anatomical sciences education. 2024 Oct;17(7):1396-405. PMID: 39169464. doi: 10.1002/ase.2502.
16. Aydogan S, Yacan Kok A, Dursun Ergezen F, Bozkurt SA. The effect of role-playing and AI-based learning on nursing students' knowledge of the nursing process and diagnostic accuracy: A quasi-experimental study. Nurse education in practice. 2026 01 Feb;91(no pagination)(104680). PMID: 2042559192.
17. Aziz S, Khan JA, Babar TSN, Sheikh A, Kazi S, Butt RZ. Incorporating Digital Tools to Facilitate Self-Directed Learning in Medical students and family medical residents. International journal of pharmacy research and technology. 2025;15(2):806‐13. PMID: CN-02895602. doi: 10.48047/ijprt/15.02.115.
18. Battista S, Furri L, de Biagi G, Depedri L, Broggi V, Testa M. Clinical records after asynchronous and synchronous e-learning courses: a multi-method randomised controlled trial on students' performance and experience. BMC medical education. 2023;23(1):584. PMID: CN-02590557. doi: 10.1186/s12909-023-04528-2.
19. Bongers PJ, Diederick van Hove P, Stassen LP, Dankelman J, Schreuder HW. A new virtual-reality training module for laparoscopic surgical skills and equipment handling: can multitasking be trained? A randomized controlled trial. Journal of surgical education. 2015 Mar-Apr;72(2):184-91. PMID: 25439179. doi: 10.1016/j.jsurg.2014.09.004.
20. Bonnin C, Pejoan D, Ranvial E, Marchat M, Andrieux N, Fourcade L, et al. Immersive virtual patient simulation compared with traditional education for clinical reasoning: a pilot randomised controlled study. Journal of visual communication in medicine. 2023;46(2):66‐74. PMID: CN-02573847. doi: 10.1080/17453054.2023.2216243.
21. Burisch C, Bellary A, Breuckmann F, Ehlers J, Thal SC, Sellmann T, et al. ChatGPT-4 Performance on German Continuing Medical Education-Friend or Foe (Trick or Treat)? Protocol for a Randomized Controlled Trial. JMIR research protocols. 2025;14:e63887. PMID: CN-02806510. doi: 10.2196/63887.
22. Chauhan S, Bhavsar A, Dutt V. Instructional modality influences neurocognitive engagement during moral learning. Scientific reports. 2025. PMID: CN-02944825. doi: 10.1038/s41598-025-31136-5.
23. Chen Y, Xiang P, Zhou Q, Li C, Zhang X, Wang J, et al. Using large language model to aid in teaching medical imaging report writing. Medical teacher. 2025 26 Dec:1-10. PMID: 649733879.
24. Dhanvijay AD, Kumari A, Pinjar MJ, Kumari A, Ganguly A, Priya A, et al. Faculty versus artificial intelligence chatbot: a comparative analysis of multiple-choice question quality in physiology. Adv Physiol Educ. 2025 Dec 1;49(4):1045-51. PMID: 40981738. doi: 10.1152/advan.00197.2025.
25. Fazlollahi AM, Yilmaz R, Winkler-Schwartz A, Mirchi N, Ledwos N, Bakhaidar M, et al. AI in Surgical Curriculum Design and Unintended Outcomes for Technical Competencies in Simulation Training. JAMA network open. 2023;6(9):e2334658. PMID: CN-02598675. doi: 10.1001/jamanetworkopen.2023.34658.
26. Feifer A, Al-Ammari A, Kovac E, Delisle J, Carrier S, Anidjar M. Randomized controlled trial of virtual reality and hybrid simulation for robotic surgical training. BJU international. 2011;108(10):1652‐6; discussion 7. PMID: CN-00812137. doi: 10.1111/j.1464-410X.2010.10060.x.
27. Fonseca G, Tipoe GL, Ganotice FA. Facilitating active learning of sectional anatomy with technology-enhanced small-group tasks: assessment of knowledge gains, technology usability, and students' perceptions. Clinical anatomy (New York, NY). 2024;37(7):769‐77. PMID: CN-02784701. doi: 10.1002/ca.24190.
28. Foster A, Chaudhary N, Kim T, Waller JL, Wong J, Borish M, et al. Using Virtual Patients to Teach Empathy: a Randomized Controlled Study to Enhance Medical Students' Empathic Communication. Simulation in healthcare. 2016;11(3):181‐9. PMID: CN-01342963. doi: 10.1097/SIH.0000000000000142.
29. Gagnon MP, Gagnon J, Desmartis M, Njoya M. The impact of blended teaching on knowledge, satisfaction, and self-directed learning in nursing undergraduates: a randomized, controlled trial. Nursing education perspectives. 2013;34(6):377‐82. PMID: CN-00982604. doi: 10.5480/10-459.
30. García-Robles P, Obrero-Gaitán E, Cortés-Pérez I, Ibancos-Losada MDR, Díaz-Fernández Á, Osuna-Pérez MC. The effectiveness of immersive virtual reality as a student-centered tool for learning neuroanatomy: a single-blind randomized controlled trial with physiotherapy students. Anatomical sciences education. 2025;18(10):1083‐94. PMID: CN-02873546. doi: 10.1002/ase.70068.
31. Giglio B, Albeloushi A, Alhaj AK, Alhantoobi M, Saeedi R, Davidovic V, et al. Artificial Intelligence-Augmented Human Instruction and Surgical Simulation Performance: a Randomized Clinical Trial. JAMA surgery. 2025;160(9):993‐1003. PMID: CN-02892993. doi: 10.1001/jamasurg.2025.2564.
32. Goh E, Gallo R, Strong E, Weng Y, Kerman H, Freed J, et al. Large Language Model Influence on Management Reasoning: a Randomized Controlled Trial. medRxiv: the preprint server for health sciences. 2024. PMID: CN-02737819. doi: 10.1101/2024.08.05.24311485.
33. Gross S, Wunderlich K, Arpagaus A, Becker C, Gössi F, Bissmann B, et al. Effectiveness of blended learning to improve medical students' communication skills: a randomized, controlled trial. BMC medical education. 2025;25(1):383. PMID: CN-02819231. doi: 10.1186/s12909-025-06938-w.
34. Hamid H, Zulkifli K, Naimat F, Che Yaacob NL, Ng KW. Exploratory study on student perception on the use of chat AI in process-driven problem-based learning. Currents in Pharmacy Teaching and Learning. 2023 01 Dec;15(12):1017-25. PMID: 2028137440.
35. Hasan MR, Khan B. An AI-based intervention for improving undergraduate STEM learning. PloS one. 2023;18(7):e0288844. PMID: CN-02585089. doi: 10.1371/journal.pone.0288844.
36. Hassoulas A, Crawford O, Hemrom S, de Almeida A, Coffey MJ, Hodgson M, et al. A pilot study investigating the efficacy of technology enhanced case based learning (CBL) in small group teaching. Scientific reports. 2025 04 May;15(1):15604. PMID: 647283538.
37. Haut KG, Epstein R, Carroll TM, Kane B, Schubert L, Hoque E. SOPHIE: testing a Virtual, Interactive, AI-Augmented End-of-Life Communication Training Tool (RP122). Journal of pain and symptom management. 2024;67(5):e794‐e5. PMID: CN-02691642. doi: 10.1016/j.jpainsymman.2024.02.469.
38. Higashitsuji A, Otsuka T, Watanabe K. Impact of ChatGPT on case creation efficiency and learning quality in case-based learning for undergraduate nursing students. TEACHING AND LEARNING IN NURSING. 2025 JAN;20(1):e159-e66. PMID: WOS:001407436200001. doi: 10.1016/j.teln.2024.10.002.
39. Hirosawa T, Yokose M, Sakamoto T, Harada Y, Tokumasu K, Mizuta K, et al. Utility of Generative Artificial Intelligence for Japanese Medical Interview Training: randomized Crossover Pilot Study. JMIR medical education. 2025;11:e77332. PMID: CN-02890375. doi: 10.2196/77332.
40. Hsu MH. Mastering medical terminology with ChatGPT and Termbot. Health education journal. 2024;83(4):352‐8. PMID: CN-02622938. doi: 10.1177/00178969231197371.
41. Kiyak YS, Coskun Ö, Budakoglu, II. 'ChatGPT can make mistakes' warnings fail: a randomized controlled trial. Medical education. 2025. PMID: CN-02913554. doi: 10.1111/medu.70056.
42. Kiyak YS, Emekli E, Is Kara T, Coskun O, Budakoglu II. AI Teaches Surgical Diagnostic Reasoning to Medical Students: Evidence from an Experiment Using a Fully Automated, Low-Cost Feedback System. Journal of surgical education. 2025 01 Oct;82(10) (no pagination)(103639). PMID: 2039881539.
43. Koch R, Gassner L, Gerlach N, Festl-Wietek T, Hirt B, Joos S, et al. Integrated e-Learning for Shoulder Anatomy and Clinical Examination Skills in First-Year Medical Students: randomized Controlled Trial. JMIR medical education. 2025;11:e62666. PMID: CN-02907197. doi: 10.2196/62666.
44. Le NN, Riscinti M. Using Real-Time Deep Learning Algorithms to Assist Novice Learners Acquire Cardiothoracic Ultrasound Images. Academic emergency medicine. 2023;30:394. PMID: CN-02621730. doi: 10.1111/acem.14718.
45. Li TP, Slocum S, Sahoo A, Ochuba A, Kolakowski L, Henn Iii RF, et al. Socratic Artificial Intelligence Learning (SAIL): the Role of a Virtual Voice Assistant in Learning Orthopedic Knowledge. Journal of surgical education. 2024;81(11):1655‐66. PMID: CN-02760434. doi: 10.1016/j.jsurg.2024.08.006.
46. Mansour L, Sushereba C, San Miguel CE, Militello LG, Allen TT, Patterson ES. Incorporating Augmented Reality Patients Into Online Trauma Training to Support Mental Model Development: an Experimental Study. Simulation in healthcare. 2025;20(4):267‐76. PMID: CN-02803414. doi: 10.1097/SIH.0000000000000839.
47. Mastour H, Dehghani T, Moradi E, Eslami S. Early prediction of medical students' performance in high-stakes examinations using machine learning approaches. HELIYON. 2023 JUL;9(7). PMID: WOS:001043024400001. doi: 10.1016/j.heliyon.2023.e18248.
48. Mastour H, Dehghani T, Moradi E, Eslami S. Explainable artificial intelligence for predicting medical students' performance in comprehensive assessments. Scientific reports. 2025 03 Jul;15(1):23752. PMID: 647866984.
49. Meinlschmidt G, Koc S, Boerner E, Tegethoff M, Simacek T, Schirmer L, et al. Enhancing professional communication training in higher education through artificial intelligence(AI)-integrated exercises: study protocol for a randomised controlled trial. BMC medical education. 2025;25(1):804. PMID: CN-02866212. doi: 10.1186/s12909-025-07307-3.
50. Molu B. Improving Nursing Students' Learning Outcomes in Neonatal Resuscitation: a Quasi‐Experimental Study Comparing AI‐Assisted Care Plan Learning With Traditional Instruction. Journal of evaluation in clinical practice. 2025;31(1):1‐9. PMID: CN-02872782. doi: 10.1111/jep.14286.
51. Morgan DJ, Scherer L, Pineles L, Baghdadi J, Magder L, Thom K, et al. Game-based learning to improve diagnostic accuracy: a pilot randomized-controlled trial. Diagnosis (Berlin, Germany). 2024;11(2):136‐41. PMID: CN-02696404. doi: 10.1515/dx-2023-0133.
52. Nakao E, Igeta M, Kobayashi N, Kumazu Y, Otani Y, Murakami M, et al. Effectiveness of artificial intelligence-based visualization for surgical anatomy education: a cluster quasirandomized controlled trial. Surgery. 2025;188:109723. PMID: CN-02913647. doi: 10.1016/j.surg.2025.109723.
53. Nazari N, Shabbir MS, Setiawan R. Application of Artificial Intelligence powered digital writing assistant in higher education: randomized controlled trial. Heliyon. 2021;7(5):e07014. PMID: CN-02648264. doi: 10.1016/j.heliyon.2021.e07014.
54. Orlando MS, Thomaier L, Abernethy MG, Chen CCG. Retention of laparoscopic and robotic skills among medical students: a randomized controlled trial. Surgical endoscopy. 2017;31(8):3306‐12. PMID: CN-01454946. doi: 10.1007/s00464-016-5363-2.
55. Padilha JM, Machado PP, Ribeiro A, Ramos J, Costa P. Clinical Virtual Simulation in Nursing Education: randomized Controlled Trial. Journal of medical Internet research. 2019;21(3):e11529. PMID: CN-01917324. doi: 10.2196/11529.
56. Qutieshat A, Al Rusheidi A, Al Ghammari S, Alarabi A, Salem A, Zelihic M. Comparative analysis of diagnostic accuracy in endodontic assessments: dental students vs. artificial intelligence. Diagnosis (Berlin, Germany). 2024 Aug 1;11(3):259-65. PMID: 38696271. doi: 10.1515/dx-2024-0034.
57. Ramezanzade S, Dascalu TL, Bakhshandeh A, Uribe SE, Ibragimov B, Bjørndal L. The Impact of Training Dental Students to Use an Artificial Intelligence-Based Platform for Pulp Exposure Prediction Prior to Deep Caries Excavation: a Proof-of-Concept Randomised Controlled Trial. International endodontic journal. 2025. PMID: CN-02921130. doi: 10.1111/iej.70046.
58. Ribeiro RVP, Maximiliano J, Barreiro G, de Souza Gastal OH, Machado PS, Marcelino LP, et al. Acquisition of robotic surgical skills does not require laparoscopic training: a randomized controlled trial. Surgical endoscopy. 2022;36(10):7325‐33. PMID: CN-02465967. doi: 10.1007/s00464-022-09118-9.
59. Rosenfeldt Nielsen M, Kristensen EQ, Jensen RO, Mollerup AM, Pfeiffer T, Graumann O. Clinical Ultrasound Education for Medical Students: virtual Reality Versus e-Learning, a Randomized Controlled Pilot Trial. Ultrasound quarterly. 2021;37(3):292‐6. PMID: CN-02322861. doi: 10.1097/RUQ.0000000000000558.
60. Sahin Karaduman G, Basak T, Duman S. Using virtual patient simulation with partial task trainer: a quasi-experimental study. Nurse education in practice. 2024;81:104177. PMID: CN-02783427. doi: 10.1016/j.nepr.2024.104177.
61. Shang Y, Cao KF, Yue JY, Zhao SZ, Hao SH, Sun YZ, et al. Comparative effectiveness of various teaching modes, including PBL, CBL, and CTTM in paediatric medical education with combined online and offline approaches. BMC medical education. 2025 Jan 2;25(1):8. PMID: 39748413. doi: 10.1186/s12909-024-06267-4.
62. Shin H, De Gagne JC, Kim SS, Hong M. The Impact of Artificial Intelligence-Assisted Learning on Nursing Students' Ethical Decision-making and Clinical Reasoning in Pediatric Care: a Quasi-Experimental Study. Computers, informatics, nursing. 2024;42(10):704‐11. PMID: CN-02742642. doi: 10.1097/CIN.0000000000001177.
63. Song D, Zhang P, Zhu Y, Qi S, Yang Y, Gong L, et al. Effects of generative artificial intelligence on higher-order thinking skills and artificial intelligence literacy in nursing undergraduates: a quasi-experimental study. Nurse education in practice. 2025;88:104549. PMID: CN-02906379. doi: 10.1016/j.nepr.2025.104549.
64. Srikasem S, Seephom S, Viriyopase A, Phutrakool P, Khowinthaseth S, Narajeenron K. Comparing the Effectiveness of Multimodal Learning Using Computer-Based and Immersive Virtual Reality Simulation-Based Interprofessional Education With Co-Debriefing, Medical Movies, and Massive Online Open Courses for Mitigating Stress and Long-Term Burnout in Medical Training: Quasi-Experimental Study. JMIR medical education. 2025 Sep 24;11:e70726. PMID: 40991944. doi: 10.2196/70726.
65. Tekin A, Karamus NF, Colak T. Anatomy exam model for the circulatory and respiratory systems using GPT-4: a medical school study. Surgical and Radiologic Anatomy. 2025 01 Dec;47(1) (no pagination)(158). PMID: 2034862319.
66. Wang H, Cho KK, Foo YM, Dalziel B, Hu W. A CHATBOT FOR PRACTICING CARDIAC AUSCULTATION - A CROSSOVER STUDY ON THE INFLUENCE OF GAMIFICATION. Journal of the American College of Cardiology. 2024;83(13):2480. PMID: CN-02685789. doi: 10.1016/S0735-1097(24)04470-X.
67. Warlick A, Clifton C, Trinh T, Kaur R, Weinberg A, Collins J. Integrating a chatbot into simulation-based perfusion training: a pilot randomized controlled trial. Perfusion. 2025:2676591251394841. PMID: CN-02929362. doi: 10.1177/02676591251394841.
68. Wolfensberger A, Desiron JC, Domenech-Jakob B, Petko D, Zingg W. To see, or not to see… pathogens in virtual reality hand hygiene training. Infection control and hospital epidemiology. 2024;45(10):1‐6. PMID: CN-02765780. doi: 10.1017/ice.2024.135.
69. Xie W, Yuan Z, Si Y, Huang Z, Li Y, Wu F, et al. Enhancing medical students' diagnostic accuracy of infectious keratitis with AI-generated images. BMC medical education. 2025 09 Jul;25(1):1027. PMID: 647928593.
70. Xuto P, Prasitwattanaseree P, Chaiboonruang T, Chaiwuth S, Khwanngern P, Nuntakwang C, et al. Development and Evaluation of an AI-Assisted Answer Assessment (4A) for Cognitive Assessments in Nursing Education. NURSING REPORTS. 2025 FEB 26;15(3). PMID: WOS:001452363000001. doi: 10.3390/nursrep15030080.
71. Zeng J, Sun K, Qin P, Liu S. Enhancing ophthalmology students' awareness of retinitis pigmentosa: assessing the efficacy of ChatGPT in AI-assisted teaching of rare diseases-a quasi-experimental study. Frontiers in medicine. 2025;12:1534294. PMID: CN-02841954. doi: 10.3389/fmed.2025.1534294.
72. Zhang K. Enhancing Critical Writing Through AI Feedback: a Randomized Control Study. Behavioral sciences (Basel, Switzerland). 2025;15(5). PMID: CN-02855040. doi: 10.3390/bs15050600.
73. Zhu P, He Y, Jiang B, Tan C, Wang B, Xia L, et al. Enhancing clinical skills education through scenario-based simulation with debriefing: a randomized controlled study on bone marrow aspiration training. Medicine. 2025;104(34):e44031. PMID: CN-02900739. doi: 10.1097/MD.0000000000044031.

# **Appendix 5. Characteristic of included RCTs**

| **Study ID** | **Study design** | **Country** | **Participants** | **Programme** | **Discipline/subfield** | **No randomised** | **Intervention description (AI application)** | **Duration of intervention** | **AI intervention category** | **Main domain of intervention (teaching-learning, assessment or both)** | **Comparison description** | **Outcomes** |
| --- | --- | --- | --- | --- | --- | --- | --- | --- | --- | --- | --- | --- |
| Akutay 2024 | RCT | Turkey | Third-year nursing students | Nursing | Surgical Nursing | 188 | DALL-E3 Image-Gen with D-ID program for creating audible and animated case presentations | 1.5 months | LLM content generator | Teaching-learning | Conventional Power Point case presentation | Case management knowledge, nursing diagnoses performance*, student satisfaction |
| Ali 2025 | RCT | Pakistan | Pharmacy students of 6th semester | Pharmacy | Pharmacy Practice | 88 | Training session on how to use AI tools (ChatGPT, Gemini and Perplexity) for generating study materials and simulating OSCE stations with personalized feedback | 4 weeks | LLM content generator + LLM virtual patient + LLM personalised feedback | Teaching-learning | Usual OSCE preparation material and instructions | Academic performance, test anxiety levels |
| Al-Kahf 2023 | RCT | France | Fourth-year medical students | Medicine | Pulmonology | 426 | Chatprogress - chatbot-based, clinical MCQ generating platform with feedback | 6 weeks | NLP rule-based chatbot | Teaching-learning | Standard university lectures (3 hours/week per specialty in traditional classroom setting) | Pulmonology sub-test score**, overall PCC (pulmonology, cardiology, critical care) exam scores, student satisfaction* |
| Aneesh 2025 | RCT | India | First-year medical students | Medicine | Physiology | 103 | Perplexity AI - a generative AI tool using large language models (LLMs) that provides real-time, natural language responses to questions with interactive conversational support. | 3 days | LLM personalised learning aid | Teaching-learning | 1. Google Search engine as SDL resource; 2. Conventional textbooks as SDL resource. | Engagement level and perceived time efficiency, theoretical knowledge (20 MCQ) |
| Arkan 2025 | RCT | Turkey | Third-year nursing students | Nursing | Nursing practice | 96 | "Artificial Intelligence Integrated Nursing Process" (AINT-P) training program using ChatGPT version 3.5, covering AI fundamentals in healthcare, nursing diagnoses and goal setting, nursing interventions, and ethical principles in AI use | 4 weeks | LLM-integrated curriculum | Teaching-learning | Standard nursing process education included in curriculum | Problem-solving skills, attitudes towards artificial intelligence, nursing process competency**, satisfaction levels |
| Aronovitz 2024 | RCT | Israel | 4th-year medical students | Medicine | Cardiac Ultrasound | 60 | Real-time EF automatic tool for cardiac ultrasound | Not specified | AI procedure assistant | Teaching-learning | Standard training without AI assistance | Performance on cardiac ultrasound views |
| Ayan 2024 | RCT | Turkey | Dental students | Dentistry | Dental Radiology | 120 | AI-powered CNN (modified YOLOv5 model) for detecting enamel and dentin caries lesions in bitewing radiographs | Single session (duration not reported) | AI imaging diagnostic aid | Teaching-learning and assessment | Standard training without AI assistance | Dental caries detection performance and labeling time |
| Benfatah 2024 | RCT | Morocco | Undergraduate nursing students | Nursing | Clinical skills/simulation | 40 | ChatGPT-assisted debriefing in simulation training | 2 weeks | LLM personalised learning aid | Teaching-learning | Standard debriefing without AI | Confidence, satisfaction, knowledge and skills |
| Benfatah 2026 | RCT | Morrocco | Undergraduate nursing students | Nursing | Clinical skills/simulation | 60 | ChatGPT-assisted pre-simulation briefing and personalised instructions | Single session (duration not reported) | LLM personalised learning aid | Teaching-learning | Standard briefing without AI | Perceived preparation, self-confidence, simulation performance, AI tool acceptability* |
| Brügge 2024 | RCT | Germany | Medical students | Medicine | Clinical decision-making (medical history taking) | 21 | ChatGPT 3.5 used to simulate patients with neurological/neurosurgical conditions (herniated disc, stroke, meningitis, concussion) for medical history conversations, with structured feedback based on Clinical Reasoning Indicator-History Taking Inventory (CRI-HTI) criteria | 4 training sessions (total 30-45 minutes) | LLM virtual patient + LLM personalised feedback | Teaching-learning | Control group conducted AI-simulated patient conversations only without receiving AI-generated feedback | Clinical decision-making performance |
| Castano-Villegas 2025 | RCT | Colombia | Clinical medical students (4th year and above) | Medicine | Clinical medicine (orthopedics, psychiatry, pediatrics, gynecology) | 83 | ArkangelAI - LLM conversational assistant that uses GPT-4o, GPT-4o mini, and GPT-o3 mini. Students answered 4 clinical questions from each of 4 clinical cases using the tool. | Single session (time not fixed) | LLM personalised learning aid | Teaching-learning | Traditional search strategies including Google search, PubMed, medical libraries, textbooks, clinical guidelines, and consultation with colleagues | Answer validity (scored by expert physicians)*; Answer efficiency (time per case); perception* |
| Chang 2024 | RCT | USA | Third-year dental students | Dentistry | Dental Radiology | 40 | AI-based interface custom-coded in React for radiograph mounting | Single session (duration not reported) | AI procedure assistant | Teaching-learning | Manual radiograph mounting | Self-confidence and time efficiency for radiograph mounting |
| Chen 2025 | RCT | China | Full-time medical undergraduates aged 17-19 | Medicine | Clinical Medicine | 40 | AI-driven personalized learning platform using Deep Q-Networks (DQN) algorithms, Immersive clinical simulation with VR-based virtual case system | 12 weeks | AI-moderated adaptive learning platform | Teaching-learning | Traditional lecture-based teaching model | Academic performance learning satisfaction and participation, self-directed learning behaviours** |
| Cheng 2020 | RCT | Taiwan | 5th year medical students | Medicine | Radiology | 30 | AI-based medical image learning system to highlight hip fracture on plain pelvic film | 6 months | AI imaging diagnostic aid | Assessment | Conventional learning without AI assistance | Diagnostic accuracy scores |
| Çiçek 2024 | RCT | Turkey | First-year medical students | Medicine | Clinical reasoning | 129 | ChatGPT-3.5 generated explanations for case-based multiple-response multiple-choice questions on urinary tract infections | 5 days | LLM content generator | Teaching-learning | Human feedback on identical questions formative tests | Clinical reasoning, openness to using artificial intelligence |
| Coşkun 2024 | RCT | Turkey | Fourth-year medical students | Medicine | Evidence-based medicine | 74 | ChatGPT (version 3.5) generated clinical vignettes and multiple-choice questions on evidence-based medicine | One month | LLM content generator | Teaching-learning and assessment | Traditional human-written cases | Students' evaluation of case suitability and quality |
| Dao 2022 | RCT | Canada | Third-year medical students | Medicine | Radiology | 32 | Chester - a web-based, locally run AI system for diagnosing frontal chest X-rays. Students used Chester during practice test | Two exam sessions (timing between exams not specified) | AI imaging diagnostic aid | Teaching-learning | Standard exam preparation without access to Chester | Diagnostic performance (exam scores), confidence*, perceived usefulness* |
| Digiacomo 2025 | RCT | Italy | Medical students in 3rd and 4th year | Medicine | Urology | 121 | ChatGPT 3.5 for self-directed learning | Single session (90 minutes)) | LLM personalised learning aid | Teaching-learning | Traditional 90-minute lecture using pre-prepared material by human teachers | Knowledge test |
| Doner 2025 | RCT | Turkey | Fourth-year nursing students | Nursing | Internal Medicine Nursing | 94 | Case presentation using: ChatGPT-4.0 mini, ChatGPT-4.0 mini high, and ChatGPT-4.1 for image generation; Sora for short video generation, InVideo platform for multimedia editing. | 5 weeks | LLM content generator | Teaching-learning | 1. Traditional teaching group (TTG): instructor-led power point presentations; 2. Case analysis group (CAG): student-prepared presentations from nephrology clinic cases | Knowledge (10 MCQ), nursing diagnosis knowledge**, case management perception (satisfaction, focus, interest, motivation - 10-point VAS scale) |
| Fang 2025 | RCT | USA | Third-and-fourth-year dental students | Dentistry | Restorative Dentistry | 86 | Custom-developed rule-based chatbot using NLP with knowledge base of 1300 clinical entries | 10-15 minutes (single session) | NLP rule-based chatbot | Teaching-learning | Traditional Blackboard (BB) online platform containing standard learning materials | AI perception/satisfaction (averaged), confidence (anxiety reduction) |
| Ferrer-Pena 2025 | RCT | Spain | Third-year physical therapy students | Physiotherapy | Clinical reasoning | 46 | Personal ChatGPT account (version 3.5) for solving clinical cases over 4 weeks, with LLM serving as a virtual patient. | 4 weeks | LLM virtual patient | Teaching-learning | Standard teaching-learning activities | Satisfaction (visual analog scale 0-100mm), Digital competencies including overall digital health literacy (26-item questionnaire) |
| Fazlollahi 2022 | RCT | Canada | Medical students | Medicine | Neurosurgery, Surgical skills | 70 | Virtual Operative Assistant (VOA) AI tutor using linear support vector machine classifier providing automated audiovisual feedback | Single session (60 minutes) | AI procedure assistant | Teaching-learning and assessment | Remote expert instruction and no-feedback control | OSATS ratings, Expertise Score (ICEMS)**, emotions and cognitive load* |
| Fung 2025 | Randomized cross over trial | Multiple (Hong Kong, Taiwan, Thailand, Spain, South Korea, Australia) | Nursing students from years 1-3 | Nursing | General nursing/Clinical competence/Cultural awareness | 44 | GenAI patient simulation system using specialized LLM with interactive clinical scenarios. | Three-day simulation intervention per phase, with one-week washout period between crossover | LLM virtual patient | Teaching-learning | VR simulation with immersive video scenarios | Self-perceived clinical competence, Cultural awareness*, AI readiness (MAIRS-MS total and subscales)*, Simulation effectiveness (SET-M)* |
| Gan 2024 | RCT | China | Third-year medical students | Medicine | Orthopedics | 129 | ChatGPT-4.0 used as learning aid | 2 weeks | LLM personalised learning aid | Teaching-learning | Conventional learning without AI assistance | MCQ test performance; Final examination scores |
| Han 2025 | RCT | Republic of Korea | Fourth-year nursing students | Nursing | Critical care nursing, Mechanical ventilation nursing | 60 | Mechanical ventilation nursing chatbot educational program using LandBot online tool with natural language processing (NLP), natural language understanding (NLU), and decision-making engine (DMG) | Not specified | NLP rule-based chatbot | Teaching-learning | Control group provided with relevant learning materials and URLs intended for independent study | Self-perceived clinical reasoning competency, self-confidence in mechanical ventilation nursing**, satisfaction with education, mechanical ventilation nursing knowledge, |
| Höhne 2025 | RCT | Germany | Clinical-phase medical students | Medicine | Radiology, Ultrasound imaging | 50 | AI-assisted ScanLab learning application with step-by-step instructional guidance, high-quality reference images, real-time anatomical structure marking, and quality assessment bar with color-coded feedback for self-directed ultrasound learning | 1 week | AI procedure assistant | Teaching-learning | Traditional instructor-led hands-on ultrasound workshop | Objective Structured Clinical Examination (OSCE) scores for ultrasound skills in lung ultrasound and FAST examination modules, ultrasound image quality assessment using SonoScore** |
| Hsu 2024 | RCT | Taiwan | Nursing students (not further specified) | Nursing | Medical Terminology | 48 | Wordbot - a LINE-based chatbot with gamified flashcard games for medical terminology learning | 4 months (minimum 1 hour/week) | NLP rule-based chatbot | Teaching-learning | Self-study without Wordbot | Medical terminology knowledge (test scores %), user satisfaction* |
| Huang 2024 | RCT | China | Medical students (not further specified) | Medicine | General Surgery | 64 | ChatGPT-assisted teaching model for "Intestinal Obstruction" course. | Not explicitly stated (appears to be single teaching session) | LLM personalised learning aid | Teaching-learning | Traditional multimedia teaching model with clinical cases using 2D text, images, videos | Satisfaction (100 points: 25 points per domain); learning compliance, theoretical knowledge assessment scores |
| Huang 2025 | RCT | China | Dental students in fourth or fifth years | Dentistry | Clinical operative skills, Dental restoration | 187 | ChatGPT-3.5 used as supplementary learning tool in addition to videos for dental operative skill acquisition | 1 week | LLM personalised learning aid | Teaching-learning | Videos alone for skill acquisition | Self-efficacy, learning motivation, desktop virtual reality operational test performance, cognitive load measured by pupil diameter changes via eye-tracking***, spatial ability effects*** |
| Hui 2025 | RCT | China | 5th-year medical students | Medicine | Clinical medical education, Urology | 42 | ChatGPT 4.0-assisted problem-based learning (PBL) teaching method | 2 weeks | LLM PBL learning aid | Teaching-learning | Traditional PBL learning | Theoretical knowledge exam scores, Mini-Clinical Evaluation Exercise (Mini-CEX) clinical skills assessment, student satisfaction survey* |
| Jiang 2024 | RCT | China | Fourth-year medical students | Medicine | Gastroenterology | 61 | Rule-based NLP dialogue system on WeChat platform that simulates patient interactions for medical history taking, diagnostic hypothesis formation, and treatment planning, with MCQ and feedback incorporated. | NLP rule-based chatbot + NLP rule-based vertual patient | 2 hours (1 hour teaching phase + 1 hour synthesis/reporting phase) | Teaching-learning | Traditional standardized patient (TSP) combined with CBL. SP instructor trained with identical case script. | Basic knowledge test, clinical thinking ability assessment, course satisfaction |
| Kalam 2025 | RCT | USA | First-year medical students | Medicine | General first-year medical curriculum (pathology, pharmacology, physiology, anatomy) | 33 | ChatGPT-4.0 (OpenAI GPT-4.0 model, April 2025 release) accessed through standard web interface for ad hoc queries during quiz | 2 weeks | LLM personalised learning aid | Teaching-learning and assessment | 2 compared arms: Group B: external resources including Google, PubMed, third-party educational websites, excluding AI-assisted tools; Group C: institutional resources such as lecture materials, electronic textbooks, course-provided slides. | Quiz score (10 MCQ), quiz completion time (task efficiency), student perceptions* |
| Kestel 2025 | RCT | Turkey | First-year nursing students | Nursing | Fundamentals of Nursing (history-taking skills training) | 82 | NLP (non LLM) rule-based chatbot simulated a virtual patient | 2 weeks | Rule-based virtual patient | Teaching-learning | Traditional teaching methods | History-taking questioning skills (0-100, higher better); Clinical stress levels (0-80, higher scores indicate higher stress levels)*, Clinical Stress- Benefit subscale (0-4, higher more positive) |
| Kobayashi 2022 | RCT | Japan | Nursing students (not further specified) | Nursing | Communication skills | 25 | Augmented reality (AR) training system with real-time graphic of patient expression. Communication skills of gaze and voice were evaluated by artificial intelligence (AI) with feedback. | 1 hour | AI communication analysis (application not specified) | Assessment | Conventional nursing communication training using mannequin | Proportion of time spent in eye contact during communication*, Empathy score JSPE-HSP change score |
| Lau 2025 | RCT | Singapore | Medical students (not further specified) | Medicine | Emergency/Critical Care Medicine | 66 | AI-enabled handheld ultrasound system (Kosmos, EchoNous) with real-time feedback on image quality, probe positioning optimization, and anatomical labelling | 1 hour | AI procedure assistant | Teaching-learning | Traditional training by human trainers | Confidence in acquiring A4C view, RACE (Rapid Assessment of Competency in Echocardiography) score, time to acquire optimal A4C view (seconds) |
| Lee 2025 | RCT | South Korea | Final-year medicine students | Medicine | Clinical skills | 19 | GPT-4o/Claude 3.5-based chatbot providing scenario-driven patient simulation with automated scoring and individualized text feedback | 1.5 hours | LLM virtual patient + LLM personalised learning aid | Teaching-learning | Peer role-play (PRP) in pairs under tutor supervision with peer feedback | OSCE performance; learning experience satisfaction* |
| Li 2023 | Randomized cross over trial | China | Third-year medical students | Medicine | Laboratory Science/Haematology | 31 | DeepCyto morphology AI system (CNN-based object detection and identification system) with morphology guide platform for blood cell identification | 4 weeks | AI imaging diagnostic aid | Teaching-learning and assessment | Conventional microscopy learning | Blood Cell Morphology Test Scores, student perspectives through interviews* |
| Liaw 2023 | RCT | Singapore | Year 3 nursing students | Nursing | Sepsis care | 64 | AI-powered virtual doctor in virtual reality simulation | Two weeks | AI-VR virtual doctor | Teaching-learning and assessment | Human-controlled virtual doctor (medical students) | Sepsis care performance, interprofessional communication performance, sepsis care knowledge, self-efficacy in interprofessional communication |
| Louie 2025 | RCT | Hong Kong | Year 5 medical students | Medicine | Obstetrics and Gynaecology | 66 | AI-avatar module using Face2Face (generative adversarial network) with voice synthesis technology | Not specified | AI-moderated adaptive learning platform | Teaching-learning | Traditional teaching with demonstration of examination on mannequin | Knowledge (MCQs)*, self-efficacy*, satisfaction* |
| Luo 2025 | RCT | China | Fourth-year medical students | Medicine | Ophthalmology | 84 | LLM-based digital patient and knowledge bank with voice-enabled virtual patient with free-text dialog, automatic scoring system based on OSCE checklists, real-time adaptive feedback. | 1 hour | LLM virtual patient + LLM personalised learning aid | Teaching-learning | Traditional real patient-based training | Medical history-taking assessment (MHTA) scores, student satisfaction and attitudes* |
| Lyu 2024 | RCT | China | Medical imaging students | Medicine | Radiology | 18 | AI-assisted diagnosis system | Not specified | AI imaging diagnostic aid | Teaching-learning and assessment | Traditional case-based teaching | Pulmonary nodule detection rate* |
| Mahrous 2023 | RCT | USA | Pre-clinical second-year dental students | Dentistry | Prosthodontics | 73 | AiDental software - AI-powered removable partial denture (RPD) design system with game component | Two months | AI gamification tool | Teaching-learning | Conventional RPD instruction without software access | RPD design academic performance, student perceptions* |
| McCarrick 2025 | RCT | Ireland | Final-year medical students | Medicine | Surgery | 90 | ChatGPT (Open AI) as simulated patient for surgical history-taking training. | 3 days | LLM virtual patient | Teaching-learning | Standard teaching-learning activities | OSCE performance*; Self-efficacy/communication confidence (5-point Likert scale)* |
| Meng 2025 | RCT | Hong Kong | Health sciences undergraduates (not further specified) | Health Sciences | Infection control | 126 | HAND-HEART: an AI-based augmented reality application providing individualized assessment and personalized feedback on hand washing technique using computer vision to analyze hand hygiene steps | Single session (duration not specified) | AI procedure assistant | Teaching-learning | Standard 7-step handwashing video and hand scanner providing visual feedback without AI-based individualized assessment | Hand washing quality (percentage correctly performing all 7 steps)*, decontamination effectiveness (percentage of residual fluorescent lotion)*, knowledge* |
| Montagna 2025 | RCT | Italy | Senior medical students | Medicine | Internal medicine, Clinical reasoning, Clinical decision-making | 16 | ChatGPT (version 3.5) used as clinical decision support system for clinical case management | Single session (duration not reported) | LLM personalised learning aid | Teaching-learning and assessment | Two control groups: 1. Clinical Practice Guidelines group using internet search for guidelines, 2. Online Repositories group using UpToDate | Case-solving performance |
| Ng 2025 | Randomized cross over trial | UK | First-year medical students | Medicine | Anatomy | 20 | Lenny AI - a custom-designed educational chatbot built on ChatGPT-4o LLM. | Single session (total 40 minutes) | LLM personalised learning aid | Teaching-learning | Conventional learning resources | Performance (SBA percentage correct); confidence and perceptions (5-point Likert scales, most relevant subscales of confidence and satisfaction selected). |
| Nissen 2025 | Randomized cross over trial | Germany | Fourth-year medical students | Medicine | Clinical reasoning, Cardiorespiratory medicine | 154 | GPT-4 generating personalized feedback on key-feature questions delivered through smartphone application "AppER" with daily push notifications | 23 days | LLM personalised learning aid | Teaching-learning and assessment | Human teachers’ comments provided after student elaborations | Clinical Exit exam percent scores (intervention vs control items), student perception* |
| Ramsamooj 2025 | RCT | USA | First-year medical students | Medicine | Pre-clinical medical education (Cell Biology, Immunology, Biochemistry, Pharmacology, Microbiology, Histology, Pathology, Medical Terminology) | 62 | LLM-based personalised AI tutoring system | Single session (duration not reported) | LLM personalised learning aid | Teaching-learning | Power-point voice-over videos | Quiz score (22 items) |
| Saatçi 2024 | RCT | Turkey | First-year nursing students | Nursing | Patient education materials development | 180 | Multiple AI tools (ChatGPT 62%, Copilot 20%, Gemini 13%, Bing 1%, CANVA 4%) used alongside traditional resources for creating patient education materials | Five days | LLM content generator | Teaching-learning | Traditional preparation methods without AI tools | PEMAT understandability and actionability scores, Global Quality Scale (GQS) scores* |
| Sahin 2025 | RCT | Turkey, Spain | Second-year physiotherapy students | Physiotherapy | Chronic Low Back Pain (CLBP) rehabilitation | 40 | AI-supported problem-based learning (AI-PBL) using ChatGPT-4.0. | 2 weeks | LLM PBL learning aid | Teaching-learning | Traditional instructor-led PBL. | Theoretical Knowledge Test (20-item MCQ, 0-100 scale), Mini Clinical Evaluation Exercise (Mini-CEX); Internet Addiction Test (IAT) - 20-item scale***; Adult Reading Motivation Scale (ARMS) - 21-item; AI Self-Efficacy Scale (AI-SES) - 22-item |
| Schropp 2023 | RCT | Denmark | Third-year dental students | Dentistry | Dental Radiology | 74 | AssistDent® AI software for detecting proximal enamel-only caries in bitewing radiographs | 1 month | AI imaging diagnostic aid | Teaching-learning and assessment | Conventional assessment without AI assistance | Proportion of dental caries diagnosed |
| Shi 2025 | RCT | China | First-year nursing students | Nursing | Critical thinking skills | 100 | LLMs (primarily ChatGPT) integrated with Problem-Based Learning to provide patient scenarios, critical feedback, literature assistance, and formative assessment support | 16 hours | LLM PBL learning aid | Teaching-learning | Traditional Problem-Based Learning (PBL) without LLM integration | Critical thinking, exit test score |
| Simsek-Cetinkaya 2023 | RCT | Turkey | First-year nursing students | Nursing | Clinical skills | 103 | AI-AISBS (Artificial Intelligence Assisted Interactive Screen-Based Simulation): AI platform with interactive communication capabilities. | Single session (~20 minutes per student) | AI-moderated adaptive learning platform | Teaching-learning | Traditional training: students performed breast examination on a trained standardized patient | BSE performance skills score (0-100), Student satisfaction (13-65), State anxiety (20-80)** |
| Staples 2025 | RCT | New Zealand | First clinical year medical students | Medicine | Surgery | Unclear (abstract) | AI simulated patient (AISP) for self-directed learning | Not reported | LLM virtual patient | Teaching-learning | Standard educational resources | Proportion who achieved distinction grades*, overall performance (standardised virtual Clinical Skills Assessment)*, satisfaction and enthusiasm* |
| Svendsen 2024 | RCT | Norway | Pharmacy students | Pharmacy | General pharmacy | 31 | ChatGPT 4.0 (privacy enhanced version) as a learning assistant | Single session (3 hours) | LLM personalised learning aid | Teaching-learning | All study tools except ChatGPT | Pharmacy knowledge scores |
| Ting 2025 | Randomized cross over trial | UK | Final-year optometry students | Optometry | Clinical optometry | 16 | AI-driven patient history and symptoms simulation using two platforms: (1) SimConverse AI chatbot trained with case-specific patient history/symptoms and feedback rubrics, and (2) ChatGPT-4o with role-play prompts | Single, 2-hour session | LLM virtual patient | Teaching-learning | Human simulated patients | Self-assessed competencies in clinical diagnosis and communication (pre-post questionnaire, 11 domains)* |
| Usta 2025 | RCT | Turkey | 4th-year dental students | Dentistry | Endodontics | 30 | Group 1 (n=10): VR haptic simulator (VirTeaSy Dental) with AI-driven modules providing real-time feedback; Group 2 (n=10): Mobile application (Dental EndoMaster) with AI-powered analytics providing procedural guidance and feedback | 1 week | AI procedure assistant + AI-moderated adaptive learning platform | Teaching-learning | Group 3 (n=10): Control group - no prior VRHS or mobile application training | Clinical performance, self-perceived clinical performance (confidence), task efficiency (drilling time), stress levels***, preparedness*, satisfaction* |
| Vannaprathip 2025 | RCT | Thailand | 5th year Dentistry students | Dentistry | Endodontics | 36 | SDMentor: VR-based intelligent tutoring system with conversational intelligent tutor that evaluates actions and generates feedback | 3 days | AI-moderated adaptive learning platform | Teaching-learning | Traditional phantom head training with supervision from human tutor | Clinical skills (modified essay questions assessing surgical decision-making skills). |
| Veras 2024 | Randomized cross over trial | Canada | Third-year health sciences students | Health Sciences | Chronic Disease and Disability | 27 | ChatGPT-3.5 with structured guidance including interaction tips and ethical/equitable use guidelines for completing academic assignments | 6 days | LLM personalised learning aid | Teaching-learning | Conventional web-based tools (any web-based tool excluding AI) | System Usability Scale (SUS) scores, students' perceptions and experiences questionnaires*, focus group discussions on experiences and perceptions* |
| Wang 2024 | RCT | China | Medical students in 3rd year of study | Medicine | Self-directed learning | 103 | LearnGuide - specialized ChatGPT tool in PBL training | 12 weeks | LLM PBL learning aid | Teaching-learning | Traditional PBL training without AI assistance | Self-directed learning skill, critical thinking, Global Flow** |
| Wang 2025 | RCT | Taiwan | First-year nursing students | Nursing | Medical terminology | 48 | Medical Terminology Escape Room Game (MTEG) - a chatbot-based gamified tool | 4 months | AI gamification tool | Teaching-learning | Traditional learning methods including flashcards, lectures, and rote memorization techniques | Medical terminology test performance, ARCS Learning Motivation Questionnaire* |
| Wang 2025 | RCT | China | Fifth-year medical students | Medicine | Clinical skills (history-taking) | 56 | GPT-based simulated patient system using ChatGPT GPTs platform (GPT-4 family model). | 4 weeks | LLM virtual patient | Teaching-learning | Traditional role-playing | Structured clinical examination (100 points); Student self-perception and satisfaction (5-point Likert scale) |
| Wu 2020 | RCT | China | Grade-two medical students | Medicine | Ophthalmology | 38 | CC-Cruiser - an AI consultation platform that uses slit lamp photos and convolutional neural networks to identify characteristics of cataract lesions and provides treatment recommendations | Single session (45 minutes) | AI imaging diagnostic aid | Teaching-learning | Traditional teaching-learning activities | Theoretical knowledge, student satisfaction* |
| Wu 2025 | RCT | China | Medical students in clinical years | Medicine | Surgery | 61 | ChatGPT-based blended teaching approach | 4 weeks | LLM personalised learning aid | Teaching-learning | Traditional teaching-learning activities | Theoretical knowledge (final exam theoretical scores), clinical skills (physical examination and practical operation scores), confidence an satisfaction |
| Yilmaz 2024 | RCT | Canada | Medical students of years 1-4 | Medicine | Neurosurgery (simulated brain tumour resection) | 97 | ICEMS (Intelligent Continuous Expertise Monitoring System) providing real-time assessment and feedback during virtual tumor resection | 90 minutes | AI procedure assistant | Teaching-learning and assessment | 1) Post-hoc feedback only 2) Expert in-person instruction | OSATS performance rating, composite performance score**, cognitive load*** |
| Yilmaz 2025 | RCT | Turkey | Fifth-year dental students | Dentistry | Dentomaxillofacial Radiology | 110 | ChatGPT-4o generated personalized learning guides | 1 month | LLM content generator | Teaching-learning | Classical feedback limited to correct/incorrect answer analysis with no content-specific feedback or guidance | Radiographic diagnostic performance (test scores), student satisfaction |

***data unsuitable for meta-analysis ** data not selected for meta-analysis despite suitability, as data from another outcome that evaluate the same domain were chosen in preference by the review authors due to greater relevance or representativeness ***not a pre-specified outcome for our review**

# **Appendix 6. Risk of bias assessment summary graphs**


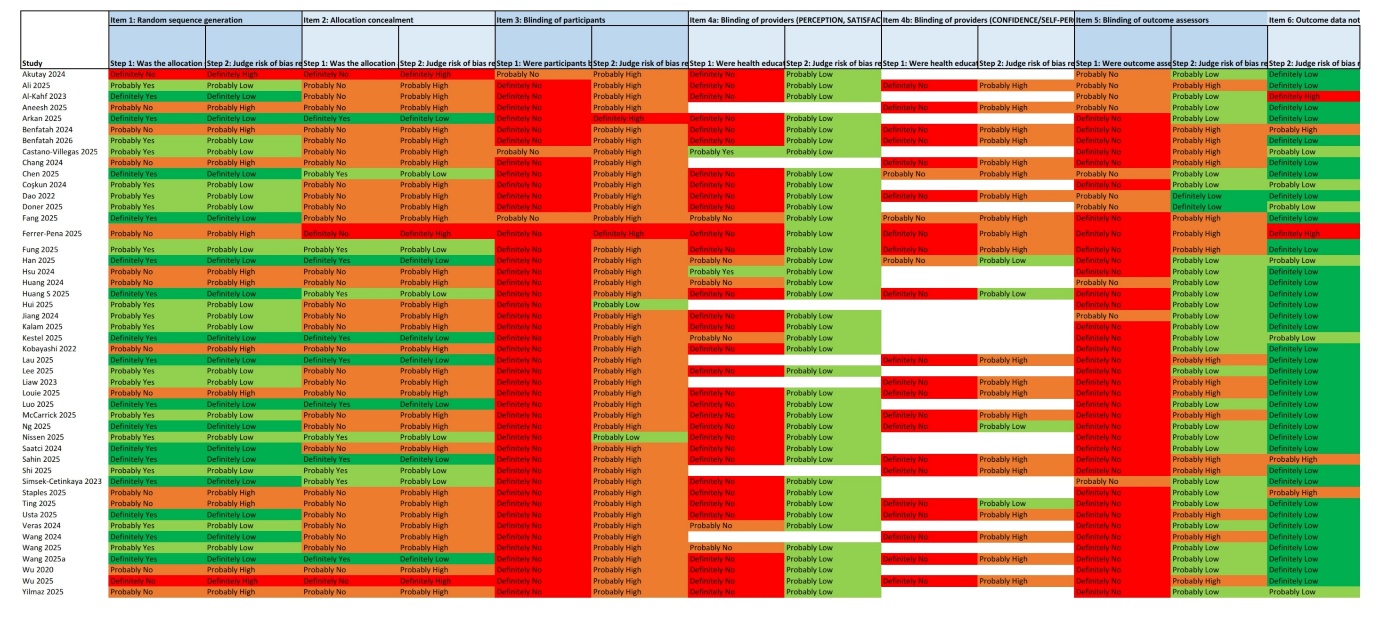


**Subjective outcomes**


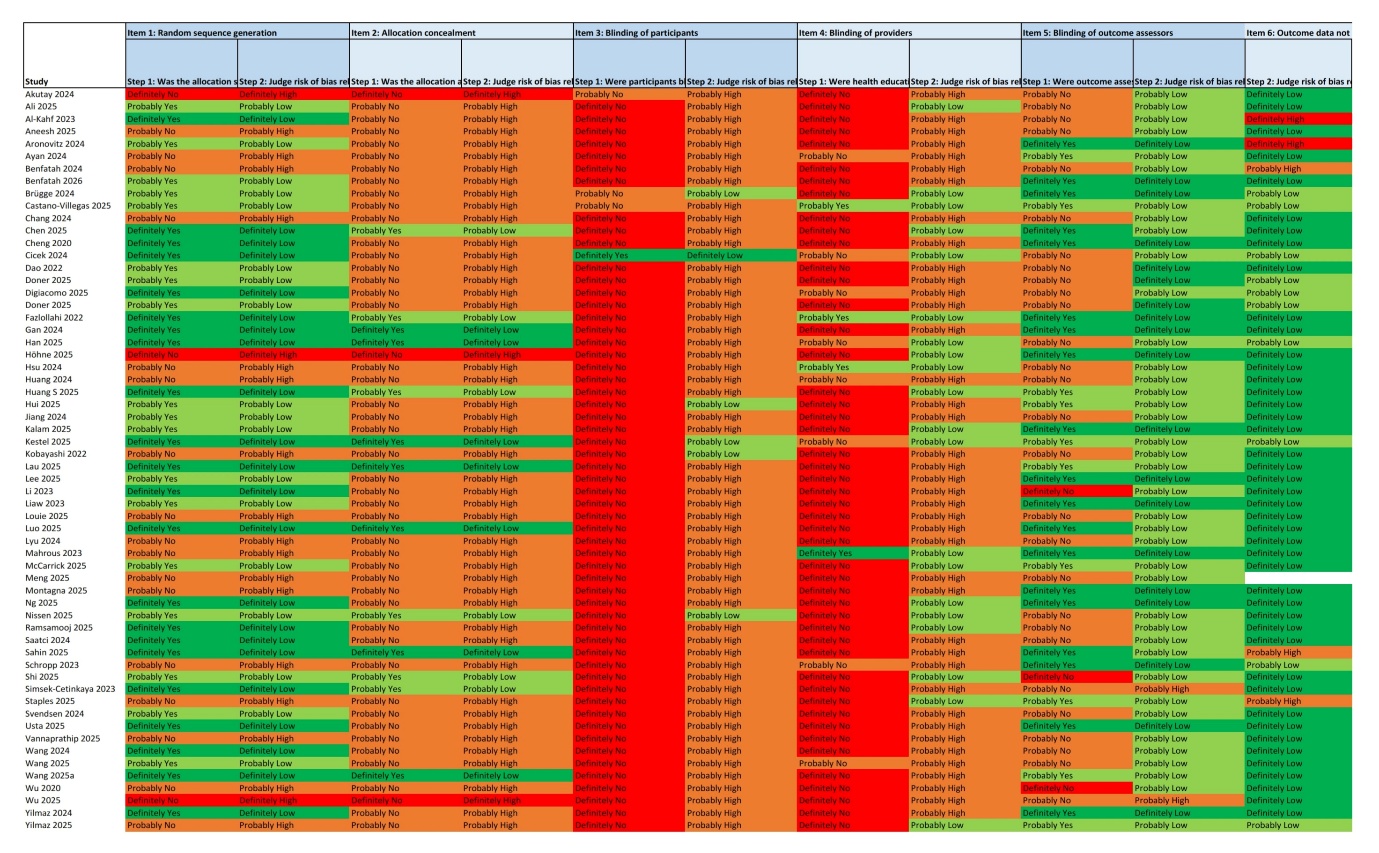


**Objective outcomes**

# **Appendix 7. Exploration of heterogeneity**

There was substantial heterogeneity across all outcomes (I² of 86–94%), with the exception of Kirkpatrick level 2: generic or personal skills (I² = 51%). To explore potential sources of heterogeneity, we conducted subgroup analyses for each outcome category. We could only perform subgroup analyses for the overall comparison of AI versus control, rather than for each specific comparison of AI subcategories, because in most specific comparisons, the number of studies are too small to enable a meaningful subgroup analysis.

The five study level variables examined in our subgroup analyses were: field of study (medicine, nursing, dentistry, pharmacy, physiotherapy, and others); region of study; AI technology type (LLM-based versus non-LLM AI applications); predominant function of the application (teaching and learning versus assessment); and number of intervention sessions (single versus multiple). Forest plots for all subgroup analyses are displayed in the supplementary analyses file (Analyses 1.10–1.14 for perception or satisfaction; 1.23–1.27 for self-efficacy and confidence; 1.37–1.41 for theoretical knowledge; 1.53–1.57 for clinical skills; 1.60–1.64 for practical skills; 1.69–1.73 for task efficiency; and 1.79–1.83 for generic or personal skills).

The same pattern was observed consistently across all outcomes and all subgroup variables: within-subgroup I² values remained high (ranging from 64% to 98% in the larger subgroups), and no subgroup variable meaningfully reduced heterogeneity. For the LLM versus non-LLM subgroup analysis in particular, the test for between-subgroup differences was non-significant for all outcomes and both subgroups independently exhibited substantial residual heterogeneity. Similarly, subgrouping by field of study, region of study, application function, and number of sessions did not identify any group of studies as the principal driver of heterogeneity; rather, each study contributed roughly equally. No single study or cluster of studies was found to account for a disproportionate share of the observed heterogeneity in any analysis. Importantly, none of these study level factors consistently moderated the direction and magnitude of the pooled estimate across outcomes.

We therefore found no basis for separating studies into distinct analytical subgroups beyond the AI sub-categories that we have already classified. We therefore accepted the pooled estimates for all outcomes and downgraded the certainty of evidence for inconsistency in all analyses where heterogeneity remained unexplained.

# **Appendix 8. Additional results of meta-analyses**

## **Funnel plot for theoretical knowledge : LLM personalised learning aid vs control**


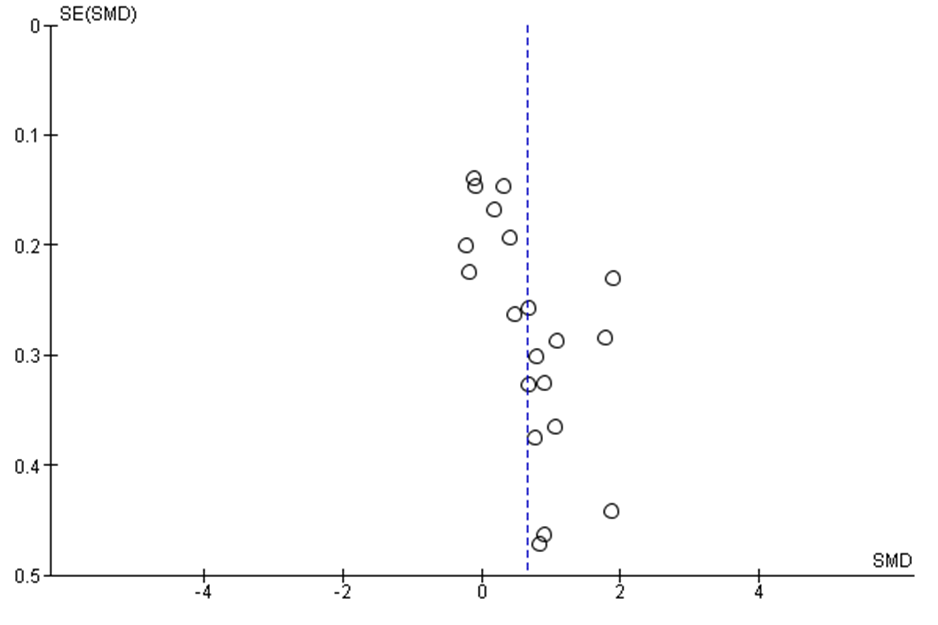


**Sensitivity analysis**

**Kirpatrick level 2: theoretical knowledge (LLM personalised learning aid vs control)**

- Overall pooled estimate: 0.53 (0.13, 0.94) (12 studies, 955 participants)
- After excluding high-risk in random sequence generation (Aneesh 2025, Wu 2024): 0.47 (0.08, 0.86) (10 studies, 688 participants)
  - Interpretation: no meaningful difference
- After excluding high-risk in allocation concealment (Aneesh 2025, Hui 2025, Kalam 2025, Ng 2025, Ramsamooj 2025, Svendsen 2024, Wu 2024): 0.13 (-0.48, 0.74) (5 studies, 512 participants)
  - Interpretation: clear difference between results – significantly higher theoretical knowledge overall vs no significant difference after excluding high-risk studies. This might indicate that low-risk studies tended to show more conversative results, or simply the result of a reduced power from reduced number of studies and participants.

# **Appendix 9. Published reviews that evaluated AI interventions for undergraduate Health Education**

**Citations (retrieved from PubMed on 20 December 2025):**

1. Ammenwerth, E., & Zwan, L. (2025). Integration of Digital Tools into Clinical Reasoning Education: A Rapid Review. *Stud Health Technol Inform, 324*, 49-50. doi:10.3233/shti250159
2. Benítez, T. M., Xu, Y., Boudreau, J. D., Kow, A. W. C., Bello, F., Van Phuoc, L., . . . Chung, K. C. (2024). Harnessing the potential of large language models in medical education: promise and pitfalls. *J Am Med Inform Assoc, 31*(3), 776-783. doi:10.1093/jamia/ocad252
3. Cecchini, M. J., Borowitz, M. J., Glassy, E. F., Gullapalli, R. R., Hart, S. N., Hassell, L. A., . . . Anderson, S. R. (2025). Harnessing the Power of Generative Artificial Intelligence in Pathology Education: Opportunities, Challenges, and Future Directions. *Arch Pathol Lab Med, 149*(2), 142-151. doi:10.5858/arpa.2024-0187-RA
4. Chary, M., Parikh, S., Manini, A. F., Boyer, E. W., & Radeos, M. (2019). A Review of Natural Language Processing in Medical Education. *West J Emerg Med, 20*(1), 78-86. doi:10.5811/westjem.2018.11.39725
5. Cheng, Y., & Zhu, L. (2025). A review of ChatGPT in medical education: exploring advantages and limitations. *Int J Surg, 111*(7), 4586-4602. doi:10.1097/js9.0000000000002505
6. Crotty, E., Singh, A., Neligan, N., Chamunyonga, C., & Edwards, C. (2024). Artificial intelligence in medical imaging education: Recommendations for undergraduate curriculum development. *Radiography (Lond), 30 Suppl 2*, 67-73. doi:10.1016/j.radi.2024.10.008
7. Currie, G. M. (2025). Generative Artificial Intelligence in Nuclear Medicine Education. *J Nucl Med Technol, 53*(1), 72-79. doi:10.2967/jnmt.124.268323
8. Dearani, J. A., & Mavroudis, C. (2025). The Emerging Influence of Artificial Intelligence on Traditional Medical Textbooks. *Ann Thorac Surg, 119*(6), 1340-1345. doi:10.1016/j.athoracsur.2025.01.018
9. Duong, M. T., Rauschecker, A. M., Rudie, J. D., Chen, P. H., Cook, T. S., Bryan, R. N., & Mohan, S. (2019). Artificial intelligence for precision education in radiology. *Br J Radiol, 92*(1103), 20190389. doi:10.1259/bjr.20190389
10. Forney, M. C., & McBride, A. F. (2020). Artificial Intelligence in Radiology Residency Training. *Semin Musculoskelet Radiol, 24*(1), 74-80. doi:10.1055/s-0039-3400270
11. Gordon, M., Daniel, M., Ajiboye, A., Uraiby, H., Xu, N. Y., Bartlett, R., . . . Thammasitboon, S. (2024). A scoping review of artificial intelligence in medical education: BEME Guide No. 84. *Med Teach, 46*(4), 446-470. doi:10.1080/0142159x.2024.2314198
12. Han, E. R., Yeo, S., Kim, M. J., Lee, Y. H., Park, K. H., & Roh, H. (2019). Medical education trends for future physicians in the era of advanced technology and artificial intelligence: an integrative review. *BMC Med Educ, 19*(1), 460. doi:10.1186/s12909-019-1891-5
13. Hanycz, S. A., & Antiperovitch, P. (2025). A practical review of generative AI in cardiac electrophysiology medical education. *J Electrocardiol, 90*, 153903. doi:10.1016/j.jelectrocard.2025.153903
14. Heinke, A., Radgoudarzi, N., Huang, B. B., & Baxter, S. L. (2024). A review of ophthalmology education in the era of generative artificial intelligence. *Asia Pac J Ophthalmol (Phila), 13*(4), 100089. doi:10.1016/j.apjo.2024.100089
15. Hersh, W. (2025). Generative Artificial Intelligence: Implications for Biomedical and Health Professions Education. *Annu Rev Biomed Data Sci, 8*(1), 355-380. doi:10.1146/annurev-biodatasci-103123-094756
16. Isaac, S., Phillips, M. R., Chen, K. A., Carlson, R., Greenberg, C. C., & Khairat, S. (2024). Usability, Acceptability, and Implementation of Artificial Intelligence (AI) and Machine Learning (ML) Techniques in Surgical Coaching and Training: A Scoping Review. *J Surg Educ, 81*(7), 994-1003. doi:10.1016/j.jsurg.2024.03.018
17. Jin, H. K., Lee, H. E., & Kim, E. (2024). Performance of ChatGPT-3.5 and GPT-4 in national licensing examinations for medicine, pharmacy, dentistry, and nursing: a systematic review and meta-analysis. *BMC Med Educ, 24*(1), 1013. doi:10.1186/s12909-024-05944-8
18. Khan, S. A., Taiyara, J., Zary, N., & Otaki, F. (2025). Artificial Intelligence in Narrative Feedback Analysis for Competency-Based Medical Education: A Review. *Stud Health Technol Inform, 327*, 1423-1427. doi:10.3233/shti250637
19. Kıyak, Y. S., & Emekli, E. (2024). ChatGPT prompts for generating multiple-choice questions in medical education and evidence on their validity: a literature review. *Postgrad Med J, 100*(1189), 858-865. doi:10.1093/postmj/qgae065
20. Lee, J., Kim, H., & Kron, F. (2024). Virtual education strategies in the context of sustainable health care and medical education: A topic modelling analysis of four decades of research. *Med Educ, 58*(1), 47-62. doi:10.1111/medu.15202
21. Lee, J., Wu, A. S., Li, D., & Kulasegaram, K. M. (2021). Artificial Intelligence in Undergraduate Medical Education: A Scoping Review. *Acad Med, 96*(11s), S62-s70. doi:10.1097/acm.0000000000004291
22. Lee, Q. Y., Chen, M., Ong, C. W., & Ho, C. S. H. (2025). The role of generative artificial intelligence in psychiatric education- a scoping review. *BMC Med Educ, 25*(1), 438. doi:10.1186/s12909-025-07026-9
23. Leon, S., Lee, S., Perez, J. E., & Hashimoto, D. A. (2025). Artificial intelligence and the education of future surgeons. *Am J Surg, 246*, 116257. doi:10.1016/j.amjsurg.2025.116257
24. Lillehaug, S. I., & Lajoie, S. P. (1998). AI in medical education--another grand challenge for medical informatics. *Artif Intell Med, 12*(3), 197-225. doi:10.1016/s0933-3657(97)00054-7
25. Liu, M., Okuhara, T., Chang, X., Shirabe, R., Nishiie, Y., Okada, H., & Kiuchi, T. (2024). Performance of ChatGPT Across Different Versions in Medical Licensing Examinations Worldwide: Systematic Review and Meta-Analysis. *J Med Internet Res, 26*, e60807. doi:10.2196/60807
26. Loi, S. J., Ng, W., Lai, C., & Chua, E. C. (2025). Artificial intelligence education in medical imaging: A scoping review. *J Med Imaging Radiat Sci, 56*(2), 101798. doi:10.1016/j.jmir.2024.101798
27. Meşe, İ., Altıntaş Taşlıçay, C., Kuzan, B. N., Kuzan, T. Y., & Sivrioğlu, A. K. (2024). Educating the next generation of radiologists: a comparative report of ChatGPT and e-learning resources. *Diagn Interv Radiol, 30*(3), 163-174. doi:10.4274/dir.2023.232496
28. Mohammad, B., Supti, T., Alzubaidi, M., Shah, H., Alam, T., Shah, Z., & Househ, M. (2023). The Pros and Cons of Using ChatGPT in Medical Education: A Scoping Review. *Stud Health Technol Inform, 305*, 644-647. doi:10.3233/shti230580
29. Morosky, C. M., Baecher-Lind, L., Chen, K. T., Fleming, A., Sims, S. M., Morgan, H. K., . . . Bhargava, R. (2025). Practical applications of artificial intelligence chatbots in obstetrics and gynecology medical education. *Am J Obstet Gynecol, 233*(1), 4-11. doi:10.1016/j.ajog.2025.04.021
30. Nagi, F., Salih, R., Alzubaidi, M., Shah, H., Alam, T., Shah, Z., & Househ, M. (2023). Applications of Artificial Intelligence (AI) in Medical Education: A Scoping Review. *Stud Health Technol Inform, 305*, 648-651. doi:10.3233/shti230581
31. Perrin, J., & Petronic-Rosic, V. (2024). The potential role and restrictions of artificial intelligence in medical school dermatology education. *Clin Dermatol, 42*(5), 477-479. doi:10.1016/j.clindermatol.2024.06.017
32. Pillai, J., & Pillai, K. (2024). ChatGPT as a medical education resource in cardiology: Mitigating replicability challenges and optimizing model performance. *Curr Probl Cardiol, 49*(12), 102879. doi:10.1016/j.cpcardiol.2024.102879
33. Proumen, L. A., Uribe-Marquez, S., Booth, L. G. J., & Mitchell, J. D. (2025). Artificial Intelligence in Medical Education. *Anesthesiol Clin, 43*(3), 563-576. doi:10.1016/j.anclin.2025.05.008
34. Qu, X., Yang, J., Chen, T., & Zhang, W. (2023). [Reflections on the Implications of the Developments in ChatGPT for Changes in Medical Education Models]. *Sichuan Da Xue Xue Bao Yi Xue Ban, 54*(5), 937-940. doi:10.12182/20231360302
35. Randhawa, G. K., & Jackson, M. (2020). The role of artificial intelligence in learning and professional development for healthcare professionals. *Healthc Manage Forum, 33*(1), 19-24. doi:10.1177/0840470419869032
36. Rincón, E. H. H., Jimenez, D., Aguilar, L. A. C., Flórez, J. M. P., Tapia Á, E. R., & Peñuela, C. L. J. (2025). Mapping the use of artificial intelligence in medical education: a scoping review. *BMC Med Educ, 25*(1), 526. doi:10.1186/s12909-025-07089-8
37. Sevgi, M., Antaki, F., & Keane, P. A. (2024). Medical education with large language models in ophthalmology: custom instructions and enhanced retrieval capabilities. *Br J Ophthalmol, 108*(10), 1354-1361. doi:10.1136/bjo-2023-325046
38. Shishehgar, S., Murray-Parahi, P., Alsharaydeh, E., Mills, S., & Liu, X. (2025). Artificial Intelligence in Health Education and Practice: A Systematic Review of Health Students' and Academics' Knowledge, Perceptions and Experiences. *Int Nurs Rev, 72*(2), e70045. doi:10.1111/inr.70045
39. Thompson, R. A. M., Shah, Y. B., Aguirre, F., Stewart, C., Lallas, C. D., & Shah, M. S. (2025). Artificial Intelligence Use in Medical Education: Best Practices and Future Directions. *Curr Urol Rep, 26*(1), 45. doi:10.1007/s11934-025-01277-1
40. Tozsin, A., Ucmak, H., Soyturk, S., Aydin, A., Gozen, A. S., Fahim, M. A., . . . Ahmed, K. (2024). The Role of Artificial Intelligence in Medical Education: A Systematic Review. *Surg Innov, 31*(4), 415-423. doi:10.1177/15533506241248239
41. Turner, L., Knopp, M. I., Mendonca, E. A., & Desai, S. (2025). Bridging Artificial Intelligence and Medical Education: Navigating the Alignment Paradox. *ATS Sch, 6*(2), 135-148. doi:10.34197/ats-scholar.2024-0086PS
42. Verghese, B. G., Iyer, C., Borse, T., Cooper, S., White, J., & Sheehy, R. (2025). Modern artificial intelligence and large language models in graduate medical education: a scoping review of attitudes, applications & practice. *BMC Med Educ, 25*(1), 730. doi:10.1186/s12909-025-07321-5
43. Waldman, C. E., Hermel, M., Hermel, J. A., Allinson, F., Pintea, M. N., Bransky, N., . . . Bhavnani, S. P. (2022). Artificial intelligence in healthcare: a primer for medical education in radiomics. *Per Med, 19*(5), 445-456. doi:10.2217/pme-2022-0014
44. Wang, S., Geng, R., & Xu, R. (2025). An Overview of Generative Artificial Intelligence in Medical Education. *J Coll Physicians Surg Pak, 35*(6), 793-796. doi:10.29271/jcpsp.2025.06.793
45. Wang, X., He, X., Wei, J., Liu, J., Li, Y., & Liu, X. (2022). Application of artificial intelligence to the public health education. *Front Public Health, 10*, 1087174. doi:10.3389/fpubh.2022.1087174
46. Ward, T. M., Mascagni, P., Madani, A., Padoy, N., Perretta, S., & Hashimoto, D. A. (2021). Surgical data science and artificial intelligence for surgical education. *J Surg Oncol, 124*(2), 221-230. doi:10.1002/jso.26496
47. Weidener, L., & Fischer, M. (2023). Teaching AI Ethics in Medical Education: A Scoping Review of Current Literature and Practices. *Perspect Med Educ, 12*(1), 399-410. doi:10.5334/pme.954
48. Xu, X., Chen, Y., & Miao, J. (2024). Opportunities, challenges, and future directions of large language models, including ChatGPT in medical education: a systematic scoping review. *J Educ Eval Health Prof, 21*, 6. doi:10.3352/jeehp.2024.21.6

# **Appendix 10. Prompts for LLMs (Claude AI and Elicit AI) and human verification process**

# Claude (Sonnet 4.5 and 4.6)

**Task: preliminary data extraction (study characteristics in terms of population, intervention, comparison, outcomes and methodologies)**

*Prompts*

# Context: To perform data extraction on study characteristics for a systematic review#

#Information: Attached are: 1. A draft background and methods for a systematic review titled 'The use of AI in undergraduate health education", 2. A relevant article that is eligible for inclusion in the systematic review, and 3. A spreadsheet that contains the major headings of study characteristics for the aforementioned article. Note the headings in the spreadsheet, which include Study ID, title, country/countries, study design: RCT, quasi-RCT, randomised cross-over trial or cluster-RCT, education field, educational subfield or discipline evaluated, population inclusion criteria, population exclusion criteria, intervention description, intervention classification (LLM or non-LLM), comparison description, outcome evaluated and funding statement #

#Role: world-leading expert in systematic review and meta-analysis, health professions education and AI#

#Tasks: Let’s take it step by step. 1. Read thoroughly the latest draft manuscript attached to understand what this paper will feed into. 2. Read thoroughly the attached full text of an included study, 3. Read thoroughly and understand all headings in the excel spreadsheet attached also understand the extent and manner that the currently included studies characteristics are compiled in this excel sheet. 3. From the attached paper, extract study characteristics and populate all relevant characteristics. Point out the location where each study characteristics is extracted (page no, paragraph no, headings etc) to facilitate human verification#

#Format: present in the form of a table as shown in the spreadsheet, 2. Be concise but included major details as expected to fit into a Characteristics of included studies table in a systematic review. #

#Follow-up task: Double check your accuracy independently before finalising. Post queries if there is anything unclear#

*Human verification process and decision*

We examined accuracy of the information extracted, aided by the location of the information as extracted by Claude. In most cases, we selected part of the key information, or summarised the information extracted to transcribe into the final spreadsheet.

Note: As we did not systematically engage these LLM platform formally for every instance of data extraction or evaluate these platforms in the context of a study, we did not have a formal record of error rates during cross-checking. However, we can report that in every single instance, there were differences in the preliminary extracted data by Claude AI that what we eventually transcribed in terms of in population and intervention characteristics, because invariably, the information extracted by Claude were far too elaborated to fit into the typical characteristics of included studies table. We have had to exercise judgment in selecting or summarising information extracted after verification with the full text. To us, this reflected either an issue with LLM in exercising judgment to be selective and restrained in offering information compared to a human, or an issue with our prompting technique, or a combination of both. Less commonly, there was a transcription error by Claude (either data in the intervention group transcribed onto the control group and vice versa, or a true error in transcribing the numerical figures). Also, Claude tended to provide data for all groups compared in multi-arm studies despite our instructions to limit the data to the control group.

**Task: Proof-reading of manuscript draft**

*Prompts*

# Context: Proof-reading a manuscript#

#Information: Attached is a draft manuscript of a systematic review titled 'The use of AI in undergraduate health education", aiming to submit to the journal xyz

#Role: experience journal editor with extensive experience in systematic review and meta-analysis#

#Tasks: Let’s take it step by step. 1. Read thoroughly the draft manuscript, from abstract to conclusions as well as the subsequent segments like acknowledgment and references 2. Assess the writing, flow, coherence of information presented in different parts, including accuracy of information including description and numerical presentations, grammar problems or typos, or inconsistency with the journal requirements, 3. Check the presentation of Tables and figures with their respective captions to ensure that they are in line with journal requirements 3. Flag out major issues observed, suggest improvement or edits without altering the writing style.

#Format: Be concise and professional. Use American spelling throughout. #

#Follow-up task: Double check your accuracy independently before finalising. Post queries if there is anything unclear#

*Human verification process and decision*

We accepted all corrections of grammar and typos, as well as suggestions of spelling to American spelling. We incorporated most of the suggested edits to make the writing to improve clarity and conciseness. We did not accept suggestion to report in more detail comparisons that involved single studies in the abstract as well as the Results proper, as we considered highlighting them risks misleading the readers on the importance we place in these analyses.

# Elicit AI

**Suggesting relevant articles for background and discussion**

**Note:** we used Elicit AI casually on a trial basis to test out this new generative AI platform.

*Prompts*

#"Context: identify key paper/s that can support the writing of the background in a systematic review titled "AI in undergraduate health education: a systematic review and meta-analysis"#

#Role: expert information specialist and medical educator#

#Tasks: 1. Please identify key paper/s that are relevant in shaping the background of this review. The background is divided into the following broad segment: a. The importance of health professions education in general, and undergraduate health professions education specifically, 2. AI, definition, and importance, 3. Use of AI in health professions education (undergraduate and post-graduate), and 4. Recent reviews on the use of AI in undergraduate health professions education#

#Format: summarise the key findings of each paper in a table, and provide the citation in a format that enables manual verification#

#Follow-up task: Verify independently that the retrieved references are authentic, and all citation details and accurate before finalising#

*Human verification process and decision*

Elicit retrieved 4 “top references” in response to this prompt, listed as follows:

1. Burney IA, Ahmad N. Artificial Intelligence in Medical Education: A citation-based systematic literature review. JSTMU [Internet]. 3Sep.2022 [cited 2Mar.2026];5(1):43-. Available from: <https://j.stmu.edu.pk/ojs/index.php/jstmu/article/view/183>
2. Gordon M, Daniel M, Ajiboye A, Uraiby H, Xu NY, Bartlett R, Hanson J, Haas M, Spadafore M, Grafton-Clarke C, Gasiea RY, Michie C, Corral J, Kwan B, Dolmans D, Thammasitboon S. A scoping review of artificial intelligence in medical education: BEME Guide No. 84. Med Teach. 2024 Apr;46(4):446-470. doi: 10.1080/0142159X.2024.2314198. Epub 2024 Feb 29. PMID: 38423127.
3. Varma JR, Fernando S, Ting BY, Aamir S, Sivaprakasam R. The Global Use of Artificial Intelligence in the Undergraduate Medical Curriculum: A Systematic Review. Cureus. 2023 May 30;15(5):e39701. doi: 10.7759/cureus.39701. PMID: 37398823; PMCID: PMC10309075.
4. L. Chen, P. Chen and Z. Lin, "Artificial Intelligence in Education: A Review," in IEEE Access, vol. 8, pp. 75264-75278, 2020, doi: 10.1109/ACCESS.2020.2988510.

Among these 4 citations, we only selected citation no 2 (BEME guide) to be included in our background. Citations 1 and 3 were not used either because there are better sources of similar information; and citation 4 was considered as not directly relevant as it covered education in general and not health professions education.

Note: We only engaged Elicit AI casually once to test out the platform when it was relatively newly launched. However, because we cited one of the four articles retrieved, we included Elicit AI in our acknowledgment list.
