# Supplementary material for: The Effectiveness of Artificial Intelligence in Undergraduate Health Professions Education: Systematic Review and Meta-Analysis of Randomized Controlled Trials
Source: JMIR Med Educ. 2026 May 5;12:e88933. doi: 10.2196/88933 (PMC13151782; doi:10.2196/88933)
Supplement: Multimedia Appendix 2 [file mededu-v12-e88933-s002.pdf]

Appendix 13: full analysis results with forest plots

Lai NM

Analyses

Analysis group 1: AI versus control

Analysis or subgroup title	No. of studies	No. of participants	Statistical method	Effect size
1.1 Kirkpatrick level 1: perception or satisfaction (LLM content generator vs control)	5	509	Std. Mean Difference (IV, Random, 95% CI)	0.65 [−0.73, 2.04]
1.2 Kirkpatrick level 1: perception or satisfaction (LLM personalised learning aid vs control)	7	430	Std. Mean Difference (IV, Random, 95% CI)	0.93 [0.40, 1.46]
1.3 Kirkpatrick level 1: perception or satisfaction (LLM virtual patient vs control)	3	127	Std. Mean Difference (IV, Random, 95% CI)	0.69 [−0.83, 2.21]
1.4 Kirkpatrick level 1: perception or satisfaction (LLM combination of content generator virtual patient personalised learning aid vs control)	1	88	Std. Mean Difference (IV, Random, 95% CI)	−0.02 [−0.44, 0.40]
1.5 Kirkpatrick level 1: perception or satisfaction (LLM-integrated curriculum vs control)	1	96	Std. Mean Difference (IV, Random, 95% CI)	1.31 [0.87, 1.76]
1.6 Kirkpatrick level 1: perception or satisfaction (non-LLM AI-moderated adaptive learning platform vs control)	2	143	Std. Mean Difference (IV, Random, 95% CI)	0.55 [−1.00, 2.11]
1.7 Kirkpatrick level 1: perception or satisfaction (NLP rule-based chatbot versus control)	2	146	Std. Mean Difference (IV, Random, 95% CI)	0.74 [−3.65, 5.13]
1.8 Kirkpatrick level 1: perception or satisfaction (NLP rule-based chatbot + rule based virtual patient vs control)	1	61	Std. Mean Difference (IV, Random, 95% CI)	0.17 [−0.33, 0.67]
1.9 Kirkpatrick level 1: perception or satisfaction (NLP rule-based virtual patient vs control)	1	79	Std. Mean Difference (IV, Random, 95% CI)	0.75 [0.29, 1.20]
1.10 Kirkpatrick level 1: perception or satisfaction (subgroup: field of study)	23	1679	Std. Mean Difference (IV, Random, 95% CI)	0.71 [0.42, 1.00]
1.10.1 Medicine	7	388	Std. Mean Difference (IV, Random, 95% CI)	0.55 [−0.14, 1.24]
1.10.2 Nursing	9	731	Std. Mean Difference (IV, Random, 95% CI)	0.92 [0.56, 1.28]
1.10.3 Dentistry	3	383	Std. Mean Difference (IV, Random, 95% CI)	1.01 [−1.74, 3.76]
1.10.4 Pharmacy	1	88	Std. Mean Difference (IV, Random, 95% CI)	−0.02 [−0.44, 0.40]
1.10.5 Physiotherapy	2	62	Std. Mean Difference (IV, Random, 95% CI)	0.13 [−0.90, 1.16]
1.10.6 Health Sciences	1	27	Std. Mean Difference (IV, Random, 95% CI)	0.64 [−0.14, 1.41]
1.10.7 Optometry	0	0	Std. Mean Difference (IV, Random, 95% CI)	Not estimable
1.10.8 Others	0	0	Std. Mean Difference (IV, Random, 95% CI)	Not estimable
1.11 Kirkpatrick level 1: perception or satisfaction (subgroup: region of study)	23	1679	Std. Mean Difference (IV, Random, 95% CI)	0.71 [0.42, 1.00]
1.11.1 Asia	7	553	Std. Mean Difference (IV, Random, 95% CI)	0.62 [0.11, 1.13]
1.11.2 North America	2	113	Std. Mean Difference (IV, Random, 95% CI)	0.47 [−0.71, 1.65]
1.11.3 South America	0	0	Std. Mean Difference (IV, Random, 95% CI)	Not estimable
1.11.4 Central America	0	0	Std. Mean Difference (IV, Random, 95% CI)	Not estimable
1.11.5 Africa	2	100	Std. Mean Difference (IV, Random, 95% CI)	1.52 [−0.41, 3.44]
1.11.6 Europe	11	869	Std. Mean Difference (IV, Random, 95% CI)	0.68 [0.14, 1.22]
1.11.7 Oceania	0	0	Std. Mean Difference (IV, Random, 95% CI)	Not estimable

Analysis or subgroup title	No. of studies	No. of participants	Statistical method	Effect size
1.11.8 Multi-continents	1	44	Std. Mean Difference (IV, Random, 95% CI)	0.53 [−0.07, 1.13]
1.12 Kirkpatrick level 1: perception or satisfaction (subgroup: LLM vs non-LLM applications)	23	1679	Std. Mean Difference (IV, Random, 95% CI)	0.71 [0.42, 1.00]
1.12.1 LLM	16	1154	Std. Mean Difference (IV, Random, 95% CI)	0.73 [0.32, 1.14]
1.12.2 Non-LLM	7	525	Std. Mean Difference (IV, Random, 95% CI)	0.69 [0.31, 1.06]
1.13 Kirkpatrick level 1: perception or satisfaction (subgroup: main function of application - teaching leaning vs assessment)	23	1679	Std. Mean Difference (IV, Random, 95% CI)	0.71 [0.42, 1.00]
1.13.1 Teaching and learning	22	1615	Std. Mean Difference (IV, Random, 95% CI)	0.72 [0.42, 1.03]
1.13.2 Assessment	0	0	Std. Mean Difference (IV, Random, 95% CI)	Not estimable
1.13.3 Teaching-learning and assessment	1	64	Std. Mean Difference (IV, Random, 95% CI)	0.45 [−0.05, 0.95]
1.14 Kirkpatrick level 1: perception or satisfaction (subgroup: single vs multiple sessions)	22	1619	Std. Mean Difference (IV, Random, 95% CI)	0.69 [0.39, 0.99]
1.14.1 Single session	5	330	Std. Mean Difference (IV, Random, 95% CI)	0.77 [0.02, 1.53]
1.14.2 Multiple sessions	17	1289	Std. Mean Difference (IV, Random, 95% CI)	0.67 [0.30, 1.03]
1.15 Kirkpatrick level 1: self-efficacy and confidence (LLM personalised learning aid vs control)	7	609	Std. Mean Difference (IV, Random, 95% CI)	0.91 [0.54, 1.29]
1.16 Kirkpatrick level 1: self-efficacy and confidence (LLM virtual patient vs control)	2	100	Std. Mean Difference (IV, Random, 95% CI)	1.36 [−8.09, 10.81]
1.17 Kirkpatrick level 1: self-efficacy and confidence (LLM-integrated curriculum vs control)	1	96	Std. Mean Difference (IV, Random, 95% CI)	1.23 [0.79, 1.67]
1.18 Kirkpatrick level 1: self-efficacy and confidence (non-LLM AI procedure assistant vs control)	1	40	Std. Mean Difference (IV, Random, 95% CI)	0.00 [−0.62, 0.62]
1.19 Kirkpatrick level 1: self-efficacy and confidence (non-LLM AI moderated adaptive learning platform vs control)	1	40	Std. Mean Difference (IV, Random, 95% CI)	2.45 [1.61, 3.28]
1.20 Kirkpatrick level 1: self-efficacy and confidence (NLP rule-based chatbot vs control)	2	146	Std. Mean Difference (IV, Random, 95% CI)	0.87 [−2.11, 3.86]
1.21 Kirkpatrick level 1: self-efficacy and confidence (non-LLM AI-VR virtual doctor vs control)	1	64	Std. Mean Difference (IV, Random, 95% CI)	−0.68 [−1.18, −0.17]
1.22 Kirkpatrick level 1: self-efficacy and confidence (AL procedure assistant + adaptive learning platform vs control)	1	20	Std. Mean Difference (IV, Random, 95% CI)	0.55 [−0.34, 1.45]
1.23 Kirkpatrick level 1: self-efficacy and confidence (subgroup: field of study)	16	1115	Std. Mean Difference (IV, Random, 95% CI)	0.91 [0.51, 1.31]
1.23.1 Medicine	5	383	Std. Mean Difference (IV, Random, 95% CI)	1.48 [0.62, 2.33]
1.23.2 Nursing	6	364	Std. Mean Difference (IV, Random, 95% CI)	0.68 [−0.06, 1.43]
1.23.3 Dentistry	4	333	Std. Mean Difference (IV, Random, 95% CI)	0.40 [0.01, 0.78]
1.23.4 Pharmacy	0	0	Std. Mean Difference (IV, Random, 95% CI)	Not estimable
1.23.5 Physiotherapy	1	35	Std. Mean Difference (IV, Random, 95% CI)	1.74 [0.95, 2.53]
1.23.6 Health Sciences	0	0	Std. Mean Difference (IV, Random, 95% CI)	Not estimable
1.23.7 Optometry	0	0	Std. Mean Difference (IV, Random, 95% CI)	Not estimable
1.23.8 Others	0	0	Std. Mean Difference (IV, Random, 95% CI)	Not estimable
1.24 Kirkpatrick level 1: self-efficacy and confidence (subgroup: region of study)	16	1115	Std. Mean Difference (IV, Random, 95% CI)	0.91 [0.51, 1.31]
1.24.1 Asia	7	674	Std. Mean Difference (IV, Random, 95% CI)	1.03 [0.07, 1.98]
1.24.2 North America	2	126	Std. Mean Difference (IV, Random, 95% CI)	0.37 [−3.85, 4.59]
1.24.3 South America	0	0	Std. Mean Difference (IV, Random, 95% CI)	Not estimable
1.24.4 Central America	0	0	Std. Mean Difference (IV, Random, 95% CI)	Not estimable
1.24.5 Africa	2	100	Std. Mean Difference (IV, Random, 95% CI)	0.89 [0.07, 1.71]
1.24.6 Europe	4	171	Std. Mean Difference (IV, Random, 95% CI)	1.19 [0.54, 1.83]

Analysis or subgroup title	No. of studies	No. of participants	Statistical method	Effect size
1.24.7 Oceania	0	0	Std. Mean Difference (IV, Random, 95% CI)	Not estimable
1.24.8 Multi-continents	1	44	Std. Mean Difference (IV, Random, 95% CI)	0.62 [0.01, 1.23]
1.25 Kirkpatrick level 1: self-efficacy and confidence (subgroup: LLM vs non-LLM)	16	1115	Std. Mean Difference (IV, Random, 95% CI)	0.91 [0.51, 1.31]
1.25.1 LLM	9	709	Std. Mean Difference (IV, Random, 95% CI)	1.02 [0.61, 1.44]
1.25.2 Non-LLM	7	406	Std. Mean Difference (IV, Random, 95% CI)	0.75 [−0.16, 1.65]
1.26 Kirkpatrick level 1: self-efficacy and confidence (subgroup: main function of application- teaching learning vs assessment)	16	1115	Std. Mean Difference (IV, Random, 95% CI)	0.91 [0.51, 1.31]
1.26.1 Teaching and learning	15	1051	Std. Mean Difference (IV, Random, 95% CI)	1.01 [0.67, 1.35]
1.26.2 Assessment	0	0	Std. Mean Difference (IV, Random, 95% CI)	Not estimable
1.26.3 Teaching-learning and assessment	1	64	Std. Mean Difference (IV, Random, 95% CI)	−0.68 [−1.18, −0.17]
1.27 Kirkpatrick level 1: self-efficacy and confidence (subgroup: single vs multiple sessions)	15	1055	Std. Mean Difference (IV, Random, 95% CI)	0.90 [0.47, 1.32]
1.27.1 Single session	4	206	Std. Mean Difference (IV, Random, 95% CI)	0.60 [−0.04, 1.23]
1.27.2 Multiple sessions	11	849	Std. Mean Difference (IV, Random, 95% CI)	1.01 [0.43, 1.58]
1.28 Kirkpatrick level 1: self-efficacy and confidence (proportion confident in echocardiography) (AI procedure assistant (non-LLM) vs control)	1	43	Risk Ratio (IV, Random, 95% CI)	1.26 [0.45, 3.50]
1.29 Kirkpatrick level 2: theoretical knowledge (LLM content generator vs control)	3	359	Std. Mean Difference (IV, Random, 95% CI)	0.99 [−1.04, 3.01]
1.30 Kirkpatrick level 2: theoretical knowledge (LLM gamification tool vs control)	1	48	Std. Mean Difference (IV, Random, 95% CI)	0.79 [0.20, 1.38]
1.31 Kirkpatrick level 2: theoretical knowledge (LLM personalised learning aid vs control)	12	955	Std. Mean Difference (IV, Random, 95% CI)	0.53 [0.13, 0.94]
1.32 Kirkpatrick level 2: theoretical knowledge (non-LLM AI moderated adaptive learning platform vs control)	1	40	Std. Mean Difference (IV, Random, 95% CI)	0.68 [0.04, 1.32]
1.33 Kirkpatrick level 2: theoretical knowledge (NLP rule-based chatbot vs control)	3	530	Std. Mean Difference (IV, Random, 95% CI)	1.06 [−2.19, 4.32]
1.34 Kirkpatrick level 2: theoretical knowledge (non-LLM AI imaging diagnostic aid vs control)	2	69	Std. Mean Difference (IV, Random, 95% CI)	1.26 [−6.23, 8.74]
1.35 Kirkpatrick level 2: theoretical knowledge (non-LLM AI-VR virtual doctor vs control)	1	64	Std. Mean Difference (IV, Random, 95% CI)	0.67 [0.16, 1.17]
1.36 Kirkpatrick level 2: theoretical knowledge (proportion with grade A or B: non-LLM AI gamification tool vs control)	1	73	Risk Ratio (IV, Random, 95% CI)	1.33 [1.01, 1.74]
1.37 Kirkpatrick level 2: theoretical knowledge (subgroup: field of study)	24	2126	Std. Mean Difference (IV, Random, 95% CI)	0.74 [0.44, 1.03]
1.37.1 Medicine	13	1194	Std. Mean Difference (IV, Random, 95% CI)	0.69 [0.32, 1.06]
1.37.2 Nursing	7	569	Std. Mean Difference (IV, Random, 95% CI)	0.73 [−0.06, 1.52]
1.37.3 Dentistry	2	297	Std. Mean Difference (IV, Random, 95% CI)	0.89 [−11.74, 13.52]
1.37.4 Pharmacy	1	31	Std. Mean Difference (IV, Random, 95% CI)	0.77 [0.04, 1.51]
1.37.5 Physiotherapy	1	35	Std. Mean Difference (IV, Random, 95% CI)	1.05 [0.34, 1.77]
1.37.6 Health Sciences	0	0	Std. Mean Difference (IV, Random, 95% CI)	Not estimable
1.37.7 Optometry	0	0	Std. Mean Difference (IV, Random, 95% CI)	Not estimable
1.37.8 Others	0	0	Std. Mean Difference (IV, Random, 95% CI)	Not estimable
1.38 Kirkpatrick level 2: theoretical knowledge (subgroup: region of study)	24	2126	Std. Mean Difference (IV, Random, 95% CI)	0.74 [0.44, 1.03]
1.38.1 Asia	14	1096	Std. Mean Difference (IV, Random, 95% CI)	0.75 [0.29, 1.20]
1.38.2 North America	2	83	Std. Mean Difference (IV, Random, 95% CI)	1.03 [−0.01, 2.07]
1.38.3 South America	0	0	Std. Mean Difference (IV, Random, 95% CI)	Not estimable
1.38.4 Central America	0	0	Std. Mean Difference (IV, Random, 95% CI)	Not estimable

Analysis or subgroup title	No. of studies	No. of participants	Statistical method	Effect size
1.38.5 Africa	0	0	Std. Mean Difference (IV, Random, 95% CI)	Not estimable
1.38.6 Europe	8	947	Std. Mean Difference (IV, Random, 95% CI)	0.68 [0.14, 1.22]
1.38.7 Oceania	0	0	Std. Mean Difference (IV, Random, 95% CI)	Not estimable
1.38.8 Multi-continents	0	0	Std. Mean Difference (IV, Random, 95% CI)	Not estimable
1.39 Kirkpatrick level 2: theoretical knowledge (subgroup: LLM vs non-LLM)	24	2126	Std. Mean Difference (IV, Random, 95% CI)	0.74 [0.44, 1.03]
1.39.1 LLM	16	1362	Std. Mean Difference (IV, Random, 95% CI)	0.64 [0.29, 0.99]
1.39.2 Non-LLM	8	764	Std. Mean Difference (IV, Random, 95% CI)	0.94 [0.28, 1.60]
1.40 Kirkpatrick level 2: theoretical knowledge (subgroup: predominant function of application - teaching learning vs assessment)	24	2126	Std. Mean Difference (IV, Random, 95% CI)	0.74 [0.44, 1.03]
1.40.1 Teaching and learning	21	2010	Std. Mean Difference (IV, Random, 95% CI)	0.69 [0.37, 1.01]
1.40.2 Assessment	0	0	Std. Mean Difference (IV, Random, 95% CI)	Not estimable
1.40.3 Teaching-learning and assessment	3	116	Std. Mean Difference (IV, Random, 95% CI)	1.10 [−0.49, 2.68]
1.41 Kirkpatrick level 2: theoretical knowledge (subgroup: single vs multiple sessions)	23	2066	Std. Mean Difference (IV, Random, 95% CI)	0.75 [0.45, 1.06]
1.41.1 Single session	6	292	Std. Mean Difference (IV, Random, 95% CI)	0.62 [0.13, 1.12]
1.41.2 Multiple sessions	17	1774	Std. Mean Difference (IV, Random, 95% CI)	0.80 [0.40, 1.20]
1.42 Kirkpatrick level 2: clinical skills (LLM content generator vs control)	2	295	Std. Mean Difference (IV, Random, 95% CI)	0.52 [−8.66, 9.69]
1.43 Kirkpatrick level 2: clinical skills (LLM personalised learning aid vs control)	9	609	Std. Mean Difference (IV, Random, 95% CI)	0.49 [0.00, 0.97]
1.44 Kirkpatrick level 2: clinical skills (LLM virtual patient vs control)	1	56	Std. Mean Difference (IV, Random, 95% CI)	2.53 [1.82, 3.25]
1.45 Kirkpatrick level 2: clinical skills (LLM content generator + LLM virtual patient + LLM personalised learning aid vs control)	1	88	Std. Mean Difference (IV, Random, 95% CI)	−0.11 [−0.53, 0.31]
1.46 Kirkpatrick level 2: clinical skills (LLM virtual patient + LLM personalised learning aid vs control)	3	124	Std. Mean Difference (IV, Random, 95% CI)	1.82 [−3.63, 7.27]
1.47 Kirkpatrick level 2: clinical skills (non-LLM AI imaging diagnostic aid vs control)	4	176	Std. Mean Difference (IV, Random, 95% CI)	0.43 [−0.41, 1.27]
1.48 Kirkpatrick level 2: clinical skills (non-LLM AI moderated adaptive learning platform vs control)	2	139	Std. Mean Difference (IV, Random, 95% CI)	0.59 [−19.98, 21.17]
1.49 Kirkpatrick level 2: clinical skills (NLP rule-based virtual patient vs control)	1	79	Std. Mean Difference (IV, Random, 95% CI)	2.03 [1.49, 2.58]
1.50 Kirkpatrick level 2: clinical skills (NLP rule-based chatbot+ virtual patient vs control)	1	61	Std. Mean Difference (IV, Random, 95% CI)	0.24 [−0.26, 0.74]
1.51 Kirkpatrick level 2: clinical skills (AI-VR virtual doctor vs control)	1	64	Std. Mean Difference (IV, Random, 95% CI)	0.21 [−0.28, 0.71]
1.52 Kirkpatrick level 2: clinical skills (AI procedure assistant + AI-moderated adaptive learning platform vs control)	1	20	Std. Mean Difference (IV, Random, 95% CI)	0.80 [−0.12, 1.72]
1.53 Kirkpatrick level 2: clinical skills (subgroup: field of study)	26	1711	Std. Mean Difference (IV, Random, 95% CI)	0.69 [0.28, 1.11]
1.53.1 Medicine	13	792	Std. Mean Difference (IV, Random, 95% CI)	0.79 [0.09, 1.49]
1.53.2 Nursing	7	626	Std. Mean Difference (IV, Random, 95% CI)	0.56 [−0.44, 1.55]
1.53.3 Dentistry	4	170	Std. Mean Difference (IV, Random, 95% CI)	0.98 [−0.43, 2.39]
1.53.4 Pharmacy	1	88	Std. Mean Difference (IV, Random, 95% CI)	−0.11 [−0.53, 0.31]
1.53.5 Physiotherapy	1	35	Std. Mean Difference (IV, Random, 95% CI)	0.41 [−0.26, 1.09]
1.53.6 Health Sciences	0	0	Std. Mean Difference (IV, Random, 95% CI)	Not estimable
1.53.7 Optometry	0	0	Std. Mean Difference (IV, Random, 95% CI)	Not estimable
1.53.8 Others	0	0	Std. Mean Difference (IV, Random, 95% CI)	Not estimable
1.54 Kirkpatrick level 2: clinical skills (subgroup: region of study)	26	1711	Std. Mean Difference (IV, Random, 95% CI)	0.69 [0.28, 1.11]

Analysis or subgroup title	No. of studies	No. of participants	Statistical method	Effect size
1.54.1 Asia	12	742	Std. Mean Difference (IV, Random, 95% CI)	0.77 [0.20, 1.33]
1.54.2 North America	1	32	Std. Mean Difference (IV, Random, 95% CI)	−0.12 [−0.82, 0.57]
1.54.3 South America	0	0	Std. Mean Difference (IV, Random, 95% CI)	Not estimable
1.54.4 Central America	0	0	Std. Mean Difference (IV, Random, 95% CI)	Not estimable
1.54.5 Africa	2	100	Std. Mean Difference (IV, Random, 95% CI)	1.01 [0.47, 1.56]
1.54.6 Europe	11	837	Std. Mean Difference (IV, Random, 95% CI)	0.66 [−0.24, 1.57]
1.54.7 Oceania	0	0	Std. Mean Difference (IV, Random, 95% CI)	Not estimable
1.54.8 Multi-continents	0	0	Std. Mean Difference (IV, Random, 95% CI)	Not estimable
1.55 Kirkpatrick level 2: clinical skills (subgroup: LLM vs non-LLM)	26	1711	Std. Mean Difference (IV, Random, 95% CI)	0.69 [0.28, 1.11]
1.55.1 LLM	16	1172	Std. Mean Difference (IV, Random, 95% CI)	0.75 [0.17, 1.33]
1.55.2 Non-LLM	10	539	Std. Mean Difference (IV, Random, 95% CI)	0.61 [−0.09, 1.31]
1.56 Kirkpatrick level 2: clinical skills (subgroup: main function - teaching learning vs assessment)	26	1711	Std. Mean Difference (IV, Random, 95% CI)	0.69 [0.28, 1.11]
1.56.1 Teaching and learning	22	1379	Std. Mean Difference (IV, Random, 95% CI)	0.78 [0.30, 1.27]
1.56.2 Assessment	0	0	Std. Mean Difference (IV, Random, 95% CI)	Not estimable
1.56.3 Teaching-learning and assessment	4	332	Std. Mean Difference (IV, Random, 95% CI)	0.21 [−0.50, 0.92]
1.57 Kirkpatrick level 2: clinical skills (subgroup: single versus multiple sessions)	26	1711	Std. Mean Difference (IV, Random, 95% CI)	0.69 [0.28, 1.11]
1.57.1 Single session	7	383	Std. Mean Difference (IV, Random, 95% CI)	0.35 [−0.38, 1.07]
1.57.2 Multiple sessions	19	1328	Std. Mean Difference (IV, Random, 95% CI)	0.83 [0.30, 1.36]
1.58 Kirkpatrick level 2: practical skills (LLM personalised learning aid vs control)	1	187	Std. Mean Difference (IV, Random, 95% CI)	0.67 [0.37, 0.96]
1.59 Kirkpatrick level 2: practical skills (non-LLM AI procedure assistant vs control)	6	305	Std. Mean Difference (IV, Random, 95% CI)	0.18 [−0.97, 1.34]
1.60 Kirkpatrick level 2: practical skills (subgroup: field of study)	7	492	Std. Mean Difference (IV, Random, 95% CI)	0.26 [−0.68, 1.20]
1.60.1 Medicine	5	265	Std. Mean Difference (IV, Random, 95% CI)	0.48 [−0.64, 1.61]
1.60.2 Nursing	0	0	Std. Mean Difference (IV, Random, 95% CI)	Not estimable
1.60.3 Dentistry	2	227	Std. Mean Difference (IV, Random, 95% CI)	−0.33 [−13.24, 12.59]
1.60.4 Pharmacy	0	0	Std. Mean Difference (IV, Random, 95% CI)	Not estimable
1.60.5 Physiotherapy	0	0	Std. Mean Difference (IV, Random, 95% CI)	Not estimable
1.60.6 Health Sciences	0	0	Std. Mean Difference (IV, Random, 95% CI)	Not estimable
1.60.7 Optometry	0	0	Std. Mean Difference (IV, Random, 95% CI)	Not estimable
1.60.8 Others	0	0	Std. Mean Difference (IV, Random, 95% CI)	Not estimable
1.61 Kirkpatrick level 2: practical skills (subgroup: region of study)	7	492	Std. Mean Difference (IV, Random, 95% CI)	0.26 [−0.68, 1.20]
1.61.1 Asia	3	290	Std. Mean Difference (IV, Random, 95% CI)	0.44 [−1.59, 2.47]
1.61.2 North America	3	152	Std. Mean Difference (IV, Random, 95% CI)	0.18 [−3.63, 3.99]
1.61.3 South America	0	0	Std. Mean Difference (IV, Random, 95% CI)	Not estimable
1.61.4 Central America	0	0	Std. Mean Difference (IV, Random, 95% CI)	Not estimable
1.61.5 Africa	0	0	Std. Mean Difference (IV, Random, 95% CI)	Not estimable

Analysis or subgroup title	No. of studies	No. of participants	Statistical method	Effect size
1.61.6 Europe	1	50	Std. Mean Difference (IV, Random, 95% CI)	−0.08 [−0.63, 0.47]
1.61.7 Oceania	0	0	Std. Mean Difference (IV, Random, 95% CI)	Not estimable
1.61.8 Multi-continents	0	0	Std. Mean Difference (IV, Random, 95% CI)	Not estimable
1.62 Kirkpatrick level 2: practical skills (subgroup: LLM vs non-LLM)	7	492	Std. Mean Difference (IV, Random, 95% CI)	0.26 [−0.68, 1.20]
1.62.1 LLM	1	187	Std. Mean Difference (IV, Random, 95% CI)	0.67 [0.37, 0.96]
1.62.2 Non-LLM	6	305	Std. Mean Difference (IV, Random, 95% CI)	0.18 [−0.97, 1.34]
1.63 Kirkpatrick level 2: practical skills (subgroup: main function of application - teaching learning vs assessment)	7	492	Std. Mean Difference (IV, Random, 95% CI)	0.26 [−0.68, 1.20]
1.63.1 Teaching and learning	5	380	Std. Mean Difference (IV, Random, 95% CI)	−0.01 [−1.21, 1.19]
1.63.2 Assessment	0	0	Std. Mean Difference (IV, Random, 95% CI)	Not estimable
1.63.3 Teaching-learning and assessment	2	112	Std. Mean Difference (IV, Random, 95% CI)	0.94 [−8.83, 10.71]
1.64 Kirkpatrick level 2: practical skills (subgroup: single vs multiple sessions)	6	432	Std. Mean Difference (IV, Random, 95% CI)	0.12 [−0.97, 1.20]
1.64.1 Single session	4	195	Std. Mean Difference (IV, Random, 95% CI)	0.01 [−2.05, 2.08]
1.64.2 Multiple sessions	2	237	Std. Mean Difference (IV, Random, 95% CI)	0.33 [−4.39, 5.05]
1.65 Kirkpatrick level 2: task efficiency (LLM personalised learning aid vs control)	2	100	Std. Mean Difference (IV, Random, 95% CI)	−0.15 [−4.24, 3.95]
1.66 Kirkpatrick level 2: task efficiency (non-LLM AI imaging diagnostic aid vs control)	1	40	Std. Mean Difference (IV, Random, 95% CI)	2.70 [1.82, 3.58]
1.67 Kirkpatrick level 2: task efficiency (non-LLM AI procedure assistant vs control)	2	52	Std. Mean Difference (IV, Random, 95% CI)	−1.26 [−14.65, 12.12]
1.68 Kirkpatrick level 2: task efficiency (non-LLM AI procedure assistant + AI-moderated adaptive learning platform vs control)	1	20	Std. Mean Difference (IV, Random, 95% CI)	−0.87 [−1.80, 0.05]
1.69 Kirkpatrick level 2: task efficiency (subgroup: field of study)	6	212	Std. Mean Difference (IV, Random, 95% CI)	−0.12 [−1.84, 1.60]
1.69.1 Medicine	3	112	Std. Mean Difference (IV, Random, 95% CI)	−0.20 [−1.05, 0.66]
1.69.2 Nursing	0	0	Std. Mean Difference (IV, Random, 95% CI)	Not estimable
1.69.3 Dentistry	3	100	Std. Mean Difference (IV, Random, 95% CI)	−0.15 [−6.52, 6.22]
1.69.4 Pharmacy	0	0	Std. Mean Difference (IV, Random, 95% CI)	Not estimable
1.69.5 Physiotherapy	0	0	Std. Mean Difference (IV, Random, 95% CI)	Not estimable
1.69.6 Health Sciences	0	0	Std. Mean Difference (IV, Random, 95% CI)	Not estimable
1.69.7 Optometry	0	0	Std. Mean Difference (IV, Random, 95% CI)	Not estimable
1.69.8 Others	0	0	Std. Mean Difference (IV, Random, 95% CI)	Not estimable
1.70 Kirkpatrick level 2: task efficiency (subgroup: region of study)	6	212	Std. Mean Difference (IV, Random, 95% CI)	−0.12 [−1.84, 1.60]
1.70.1 Asia	1	12	Std. Mean Difference (IV, Random, 95% CI)	−0.16 [−1.36, 1.04]
1.70.2 North America	2	61	Std. Mean Difference (IV, Random, 95% CI)	−0.99 [−17.29, 15.30]
1.70.3 South America	1	79	Std. Mean Difference (IV, Random, 95% CI)	−0.38 [−0.83, 0.06]
1.70.4 Central America	0	0	Std. Mean Difference (IV, Random, 95% CI)	Not estimable
1.70.5 Africa	0	0	Std. Mean Difference (IV, Random, 95% CI)	Not estimable
1.70.6 Europe	2	60	Std. Mean Difference (IV, Random, 95% CI)	0.92 [−21.77, 23.61]
1.70.7 Oceania	0	0	Std. Mean Difference (IV, Random, 95% CI)	Not estimable
1.70.8 Multi-continents	0	0	Std. Mean Difference (IV, Random, 95% CI)	Not estimable

Analysis or subgroup title	No. of studies	No. of participants	Statistical method	Effect size
1.71 Kirkpatrick level 2: task efficiency (subgroup: LLM vs non-LLM)	6	212	Std. Mean Difference (IV, Random, 95% CI)	−0.12 [−1.84, 1.60]
1.71.1 LLM	2	100	Std. Mean Difference (IV, Random, 95% CI)	−0.15 [−4.24, 3.95]
1.71.2 Non-LLM	4	112	Std. Mean Difference (IV, Random, 95% CI)	−0.15 [−3.50, 3.19]
1.72 Kirkpatrick level 2: task efficiency (subgroup: main function of application - teaching learning vs assessment)	6	212	Std. Mean Difference (IV, Random, 95% CI)	−0.12 [−1.84, 1.60]
1.72.1 Teaching and learning	4	151	Std. Mean Difference (IV, Random, 95% CI)	−0.94 [−2.44, 0.57]
1.72.2 Assessment	0	0	Std. Mean Difference (IV, Random, 95% CI)	Not estimable
1.72.3 Teaching-learning and assessment	2	61	Std. Mean Difference (IV, Random, 95% CI)	1.50 [−13.78, 16.77]
1.73 Kirkpatrick level 2: task efficiency (subgroup: single vs multiple sessions)	6	212	Std. Mean Difference (IV, Random, 95% CI)	−0.12 [−1.84, 1.60]
1.73.1 Single session	4	171	Std. Mean Difference (IV, Random, 95% CI)	−0.04 [−3.30, 3.23]
1.73.2 Multiple sessions	2	41	Std. Mean Difference (IV, Random, 95% CI)	−0.28 [−7.69, 7.14]
1.74 Kirkpatrick level 2: generic or personal skills (LLM personalised learning aid vs control)	1	101	Std. Mean Difference (IV, Random, 95% CI)	0.45 [0.05, 0.84]
1.75 Kirkpatrick level 2: generic or personal skills (LLM virtual patient vs control)	1	27	Std. Mean Difference (IV, Random, 95% CI)	0.00 [−1.06, 1.06]
1.76 Kirkpatrick level 2: generic or personal skills (LLM-integrated curriculum vs control)	1	96	Std. Mean Difference (IV, Random, 95% CI)	0.60 [0.19, 1.01]
1.77 Kirkpatrick level 2: generic or personal skills (non-LLM AI communication analysis vs control)	1	25	Std. Mean Difference (IV, Random, 95% CI)	1.85 [0.88, 2.81]
1.78 Kirkpatrick level 2: generic or personal skills (AI-VR virtual doctor vs control)	1	64	Std. Mean Difference (IV, Random, 95% CI)	0.31 [−0.18, 0.81]
1.79 Kirkpatrick level 2: generic or personal skills (subgroup: field of study)	5	313	Std. Mean Difference (IV, Random, 95% CI)	0.57 [−0.09, 1.23]
1.79.1 Medicine	1	101	Std. Mean Difference (IV, Random, 95% CI)	0.45 [0.05, 0.84]
1.79.2 Nursing	3	185	Std. Mean Difference (IV, Random, 95% CI)	0.82 [−1.07, 2.72]
1.79.3 Dentistry	0	0	Std. Mean Difference (IV, Random, 95% CI)	Not estimable
1.79.4 Pharmacy	0	0	Std. Mean Difference (IV, Random, 95% CI)	Not estimable
1.79.5 Physiotherapy	1	27	Std. Mean Difference (IV, Random, 95% CI)	0.00 [−1.06, 1.06]
1.79.6 Health Sciences	0	0	Std. Mean Difference (IV, Random, 95% CI)	Not estimable
1.79.7 Optometry	0	0	Std. Mean Difference (IV, Random, 95% CI)	Not estimable
1.79.8 Others	0	0	Std. Mean Difference (IV, Random, 95% CI)	Not estimable
1.80 Kirkpatrick level 2: generic or personal skills (subgroup: region of study)	5	313	Std. Mean Difference (IV, Random, 95% CI)	0.57 [−0.09, 1.23]
1.80.1 Asia	3	190	Std. Mean Difference (IV, Random, 95% CI)	0.77 [−1.21, 2.76]
1.80.2 North America	0	0	Std. Mean Difference (IV, Random, 95% CI)	Not estimable
1.80.3 South America	0	0	Std. Mean Difference (IV, Random, 95% CI)	Not estimable
1.80.4 Central America	0	0	Std. Mean Difference (IV, Random, 95% CI)	Not estimable
1.80.5 Africa	0	0	Std. Mean Difference (IV, Random, 95% CI)	Not estimable
1.80.6 Europe	2	123	Std. Mean Difference (IV, Random, 95% CI)	0.51 [−2.23, 3.24]
1.80.7 Oceania	0	0	Std. Mean Difference (IV, Random, 95% CI)	Not estimable
1.80.8 Multi-continents	0	0	Std. Mean Difference (IV, Random, 95% CI)	Not estimable
1.81 Kirkpatrick level 2: generic or personal skills (subgroup: LLM vs non-LLM)	5	313	Std. Mean Difference (IV, Random, 95% CI)	0.57 [−0.09, 1.23]
1.81.1 LLM	2	128	Std. Mean Difference (IV, Random, 95% CI)	0.39 [−1.46, 2.24]

Analysis or subgroup title	No. of studies	No. of participants	Statistical method	Effect size
1.81.2 Non-LLM	3	185	Std. Mean Difference (IV, Random, 95% CI)	0.82 [−1.07, 2.72]
1.82 Kirkpatrick level 2: generic or personal skills (subgroup: main function of application - teaching learning vs assessment)	5	313	Std. Mean Difference (IV, Random, 95% CI)	0.57 [−0.09, 1.23]
1.82.1 Teaching and learning	3	224	Std. Mean Difference (IV, Random, 95% CI)	0.48 [0.03, 0.94]
1.82.2 Assessment	1	25	Std. Mean Difference (IV, Random, 95% CI)	1.85 [0.88, 2.81]
1.82.3 Teaching-learning and assessment	1	64	Std. Mean Difference (IV, Random, 95% CI)	0.31 [−0.18, 0.81]
1.83 Kirkpatrick level 2: generic or personal skills (subgroup: single vs multiple sessions)	5	313	Std. Mean Difference (IV, Random, 95% CI)	0.57 [−0.09, 1.23]
1.83.1 Single session	1	25	Std. Mean Difference (IV, Random, 95% CI)	1.85 [0.88, 2.81]
1.83.2 Multiple sessions	4	288	Std. Mean Difference (IV, Random, 95% CI)	0.44 [0.17, 0.72]

Figures and tables

Analysis 1.1: Kirkpatrick level 1: perception or satisfaction (LLM content generator vs control)

	LLM content generator			Control group				Std. Mean Difference	Std. Mean Difference
**Study or Subgroup**	**Mean [SMD]**	**SD [SMD]**	**Total**	**Mean [SMD]**	**SD [SMD]**	**Total**	**Weight**	**IV, Random, 95% CI [SMD]**	**IV, Random, 95% CI [SMD]**
Akutay 2024	9.08	1.45	94	8.8	1.41	94	20.5%	0.19 [-0.09 , 0.48]	
Çiçek 2024	4.62	1.44	39	5.53	1.14	47	20.1%	-0.70 [-1.14 , -0.26]	
Coşkun 2024	4.44	0.61	34	4.1	0.88	30	19.8%	0.45 [-0.05 , 0.95]	
Doner 2025	9.86	0.36	33	8.78	1.43	28	19.7%	1.06 [0.52 , 1.60]	
Yilmaz 2025	4.7	0.3	55	3.3	0.8	55	19.9%	2.30 [1.82 , 2.79]	
**Total (HKSJ [a])**			**255**			**254**	**100.0%**	**0.65 [-0.73 , 2.04]**	
95% prediction interval								[-2.67 , 3.98]	

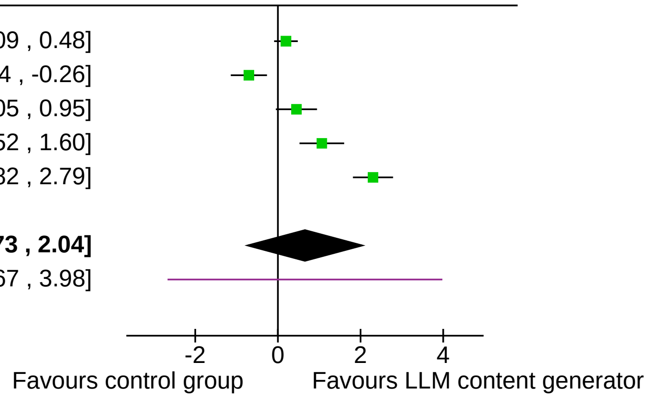

Test for overall effect: T = 1.31, df = 4 (P = 0.26)

Heterogeneity: Tau² (REML [b]) = 1.19; Chi² = 90.37, df = 4 (P < 0.00001); I² = 96%

Footnotes

[a] CI calculated by Hartung-Knapp-Sidik-Jonkman (HKSJ) method.

[b] Tau² calculated by Restricted Maximum-Likelihood method.

Analysis 1.2: Kirkpatrick level 1: perception or satisfaction (LLM personalised learning aid vs control)

Study or Subgroup	LLM personalised learning aid Mean [SMD]	SD [SMD]	Total	Control group Mean [SMD]	SD [SMD]	Total	Weight	Std. Mean Difference IV, Random, 95% CI [SMD]
Benfatah 2024	8.9	0.7	20	7.8	0.9	20	13.7%	1.34 [0.64 , 2.03]
Benfatah 2026	44.8	3.2	30	38.1	4.7	30	15.0%	1.64 [1.05 , 2.24]
Huang 2025	4.5	2.04	94	3.8	2.04	93	18.9%	0.34 [0.05 , 0.63]
Ng 2025	4	0.94	10	2.6	1.16	10	10.2%	1.27 [0.29 , 2.25]
Sahin 2025	74.2	3.9	19	73.2	6.8	16	14.0%	0.18 [-0.49 , 0.85]
Veras 2024	67.29	9.67	14	60.1	12.19	13	12.6%	0.64 [-0.14 , 1.41]
Wu 2024	17.23	1.33	31	15.38	1.54	30	15.6%	1.27 [0.72 , 1.82]
**Total (HKSJ [a])**			**218**			**212**	**100.0%**	**0.93 [0.40 , 1.46]**
95% prediction interval								[-0.40 , 2.26]

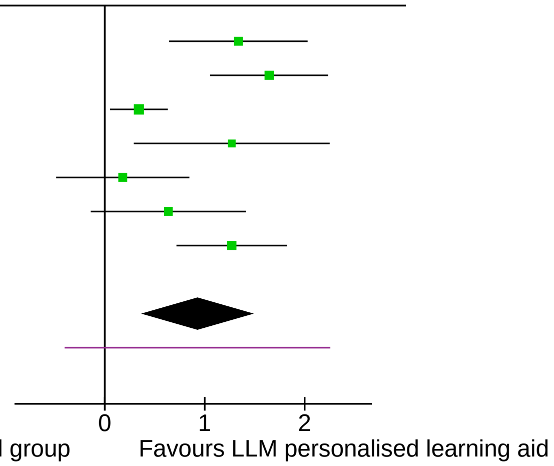

Test for overall effect: T = 4.26, df = 6 (P = 0.005)

Heterogeneity: Tau² (REML [b]) = 0.25; Chi² = 26.61, df = 6 (P = 0.0002); I² = 74%

Footnotes

[a] CI calculated by Hartung-Knapp-Sidik-Jonkman (HKSJ) method.

[b] Tau² calculated by Restricted Maximum-Likelihood method.

Analysis 1.3: Kirkpatrick level 1: perception or satisfaction (LLM virtual patient vs control)

Study or Subgroup	LLM virtual patient Mean [SMD]	SD [SMD]	Total	Control group Mean [SMD]	SD [SMD]	Total	Weight	Std. Mean Difference IV, Random, 95% CI [SMD]
Ferrer-Peña 2025	77	14.07	4	77	17.04	23	23.4%	0.00 [-1.06 , 1.06]
Fung 2025	8.45	8.25	22	4	8.25	22	37.9%	0.53 [-0.07 , 1.13]
Wang 2025a	3	1.48	28	1.5	0.74	28	38.8%	1.26 [0.69 , 1.84]
**Total (HKSJ [a])**			**54**			**73**	**100.0%**	**0.69 [-0.83 , 2.21]**
95% prediction interval								[-1.86 , 3.24]

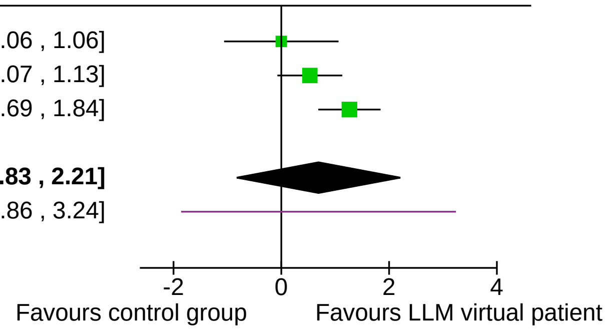

Test for overall effect: T = 1.96, df = 2 (P = 0.19)

Heterogeneity: Tau² (REML [b]) = 0.23; Chi² = 5.43, df = 2 (P = 0.07); I² = 64%

Footnotes

[a] CI calculated by Hartung-Knapp-Sidik-Jonkman (HKSJ) method.

[b] Tau² calculated by Restricted Maximum-Likelihood method.

Analysis 1.4: Kirkpatrick level 1: perception or satisfaction (LLM combination of content generator virtual patient personalised learning aid vs control)

Study or Subgroup	LLM content generator + LLM virtual patient + LLM personalised learning aid Mean [SMD]	SD [SMD]	Total	Control group Mean [SMD]	SD [SMD]	Total	Weight	Std. Mean Difference IV, Random, 95% CI [SMD]
Ali 2025	44.93	10.97	44	45.18	11.3	44	100.0%	-0.02 [-0.44 , 0.40]
**Total**			**44**			**44**	**100.0%**	**-0.02 [-0.44 , 0.40]**
95% prediction interval								[-0.44 , 0.40]

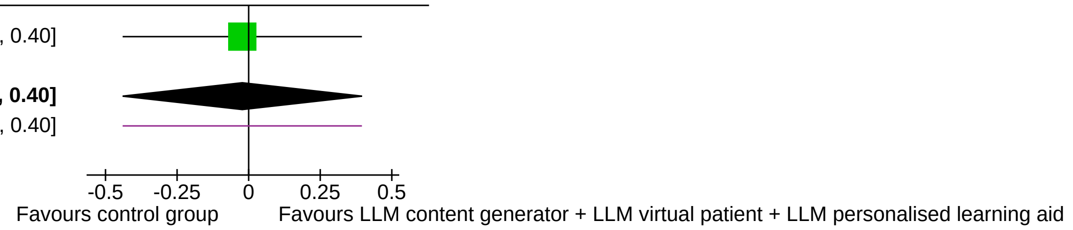

Test for overall effect: Z = 0.10 (P = 0.92)

Heterogeneity: Not applicable

Analysis 1.5: Kirkpatrick level 1: perception or satisfaction (LLM-integrated curriculum vs control)

Study or Subgroup	LLM-integrated curriculum Mean [SMD]	SD [SMD]	Total	Control group Mean [SMD]	SD [SMD]	Total	Weight	Std. Mean Difference IV, Random, 95% CI [SMD]
Arkan 2025	7.25	1.08	48	5.52	1.5	48	100.0%	1.31 [0.87 , 1.76]
**Total**			**48**			**48**	**100.0%**	**1.31 [0.87 , 1.76]**
95% prediction interval								[0.87 , 1.76]

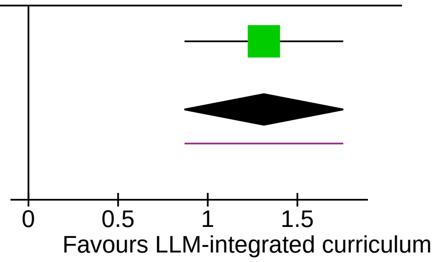

Test for overall effect: Z = 5.81 (P < 0.00001)

Heterogeneity: Not applicable

Analysis 1.6: Kirkpatrick level 1: perception or satisfaction (non-LLM AI-moderated adaptive learning platform vs control)

Study or Subgroup	AI-moderated adaptive learning platform (non-LLM) Mean [SMD]	SD [SMD]	Total	Control group Mean [SMD]	SD [SMD]	Total	Weight	Std. Mean Difference IV, Random, 95% CI [SMD]
Chen 2025	17.45	3.94	20	16.05	3.69	20	28.6%	0.36 [-0.27 , 0.98]
Simsek-Cetinkaya 2023	56.59	6.27	52	50.45	12.19	51	71.4%	0.63 [0.23 , 1.03]
**Total (HKSJ [a])**			**72**			**71**	**100.0%**	**0.55 [-1.00 , 2.11]**
95% prediction interval								[-1.00 , 2.11]

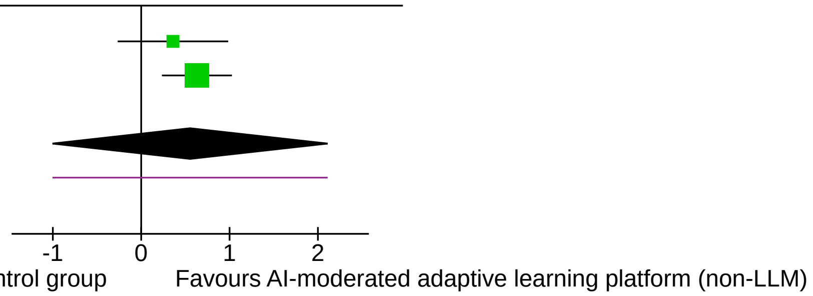

Test for overall effect: T = 4.51, df = 1 (P = 0.14)

Heterogeneity: Tau^2 (REML [b]) = 0.00; Chi^2 = 0.52, df = 1 (P = 0.47); I^2 = 0%

Footnotes

[a] CI calculated by Hartung-Knapp-Sidik-Jonkman (HKSJ) method.

[b] Tau^2 calculated by Restricted Maximum-Likelihood method.

Analysis 1.7: Kirkpatrick level 1: perception or satisfaction (NLP rule-based chatbot versus control)

Study or Subgroup	NLP rule-based chatbot (non-LLM) Mean [SMD]	SD [SMD]	Total	Control group Mean [SMD]	SD [SMD]	Total	Weight	Std. Mean Difference IV, Random, 95% CI [SMD]
Fang 2025	4.05	0.84	43	3.61	1.22	43	53.1%	0.42 [-0.01 , 0.84]
Han 2025	7.42	1.59	31	5.55	1.74	29	46.9%	1.11 [0.56 , 1.66]
**Total (HKSJ [a])**			**74**			**72**	**100.0%**	**0.74 [-3.65 , 5.13]**
95% prediction interval								[-6.18 , 7.66]

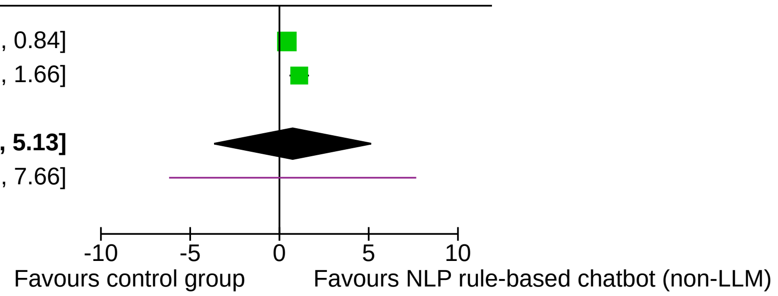

Test for overall effect: T = 2.14, df = 1 (P = 0.28)

Heterogeneity: Tau^2 (REML [b]) = 0.18; Chi^2 = 3.83, df = 1 (P = 0.05); I^2 = 74%

Footnotes

[a] CI calculated by Hartung-Knapp-Sidik-Jonkman (HKSJ) method.

[b] Tau^2 calculated by Restricted Maximum-Likelihood method.

Analysis 1.8: Kirkpatrick level 1: perception or satisfaction (NLP rule-based chatbot + rule based virtual patient vs control)

	NLP rule-based chatbot + NLP rule-based virtual patient			Control group				Std. Mean Difference
Study or Subgroup	Mean [SMD]	SD [SMD]	Total	Mean [SMD]	SD [SMD]	Total	Weight	IV, Random, 95% CI [SMD]
Jiang 2024	20.6	3.3	31	20	3.6	30	100.0%	0.17 [-0.33 , 0.67]
**Total**			**31**			**30**	**100.0%**	**0.17 [-0.33 , 0.67]**
95% prediction interval								[-0.33 , 0.67]

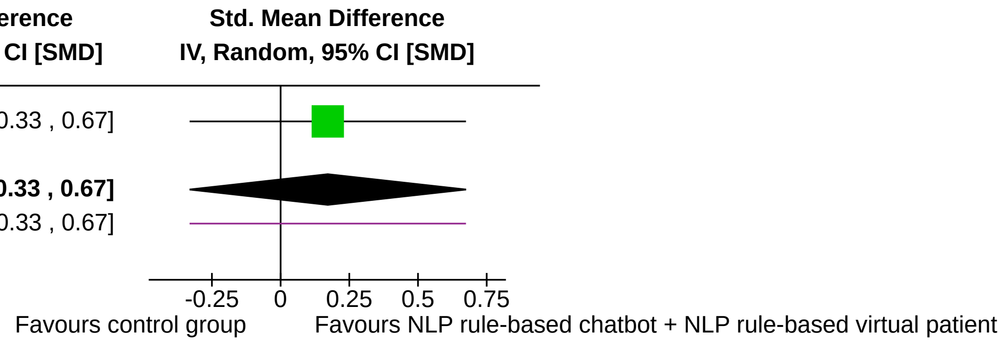

Test for overall effect: Z = 0.67 (P = 0.50)

Heterogeneity: Not applicable

Analysis 1.9: Kirkpatrick level 1: perception or satisfaction (NLP rule-based virtual patient vs control)

	Rule-based virtual patient (non-LLM)			Control group				Std. Mean Difference
Study or Subgroup	Mean [SMD]	SD [SMD]	Total	Mean [SMD]	SD [SMD]	Total	Weight	IV, Random, 95% CI [SMD]
Kestel 2025	4.68	1.85	38	3.31	1.79	41	100.0%	0.75 [0.29 , 1.20]
**Total**			**38**			**41**	**100.0%**	**0.75 [0.29 , 1.20]**
95% prediction interval								[0.29 , 1.20]

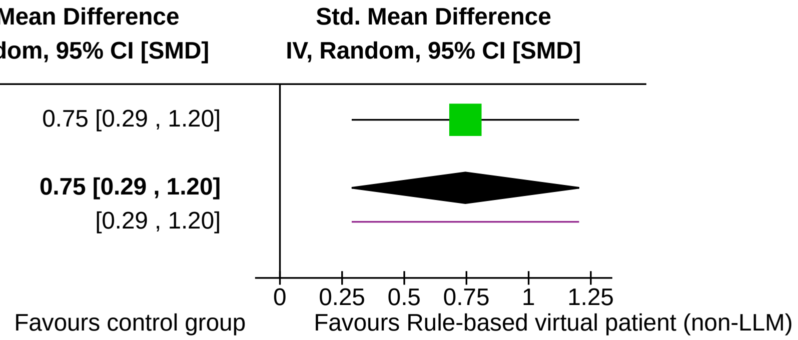

Test for overall effect: Z = 3.20 (P = 0.001)

Heterogeneity: Not applicable

Analysis 1.10: Kirkpatrick level 1: perception or satisfaction (subgroup: field of study)

Study or Subgroup	AI Mean [SMD]	AI SD [SMD]	AI Total	Control group Mean [SMD]	Control group SD [SMD]	Control group Total	Weight	Std. Mean Difference IV, Random, 95% CI [SMD]
**1.10.1 Medicine**								
Chen 2025	17.45	3.94	20	16.05	3.69	20	4.2%	0.36 [-0.27 , 0.98]
Çiçek 2024	4.62	1.44	39	5.53	1.14	47	4.7%	-0.70 [-1.14 , -0.26]
Coşkun 2024	4.44	0.61	34	4.1	0.88	30	4.5%	0.45 [-0.05 , 0.95]
Jiang 2024	20.6	3.3	31	20	3.6	30	4.5%	0.17 [-0.33 , 0.67]
Ng 2025	4	0.94	10	2.6	1.16	10	3.2%	1.27 [0.29 , 2.25]
Wang 2025a	3	1.48	28	1.5	0.74	28	4.3%	1.26 [0.69 , 1.84]
Wu 2024	17.23	1.33	31	15.38	1.54	30	4.4%	1.27 [0.72 , 1.82]
**Subtotal (HKSJ [a])**			**193**			**195**	**29.8%**	**0.55 [-0.14 , 1.24]**
Test for overall effect: T = 1.94, df = 6 (P = 0.10)								
Heterogeneity: Tau² (REML [b]) = 0.48; Chi² = 46.31, df = 6 (P < 0.00001); I² = 86%								
**1.10.2 Nursing**								
Akutay 2024	9.08	1.45	94	8.8	1.41	94	5.0%	0.19 [-0.09 , 0.48]
Arkan 2025	7.25	1.08	48	5.52	1.5	48	4.7%	1.31 [0.87 , 1.76]
Benfatah 2024	8.9	0.7	20	7.8	0.9	20	4.0%	1.34 [0.64 , 2.03]
Benfatah 2026	44.8	3.2	30	38.1	4.7	30	4.3%	1.64 [1.05 , 2.24]
Doner 2025	9.86	0.36	33	8.78	1.43	28	4.4%	1.06 [0.52 , 1.60]
Fung 2025	8.45	8.25	22	4	8.25	22	4.2%	0.53 [-0.07 , 1.13]
Han 2025	7.42	1.59	31	5.55	1.74	29	4.4%	1.11 [0.56 , 1.66]
Kestel 2025	4.68	1.85	38	3.31	1.79	41	4.6%	0.75 [0.29 , 1.20]
Simsek-Cetinkaya 2023	56.59	6.27	52	50.45	12.19	51	4.8%	0.63 [0.23 , 1.03]
**Subtotal (HKSJ [a])**			**368**			**363**	**40.4%**	**0.92 [0.56 , 1.28]**
Test for overall effect: T = 5.88, df = 8 (P = 0.0004)								
Heterogeneity: Tau² (REML [b]) = 0.16; Chi² = 35.96, df = 8 (P < 0.0001); I² = 74%								
**1.10.3 Dentistry**								
Fang 2025	4.05	0.84	43	3.61	1.22	43	4.7%	0.42 [-0.01 , 0.84]
Huang 2025	4.5	2.04	94	3.8	2.04	93	5.0%	0.34 [0.05 , 0.63]
Yilmaz 2025	4.7	0.3	55	3.3	0.8	55	4.6%	2.30 [1.82 , 2.79]
**Subtotal (HKSJ [a])**			**192**			**191**	**14.3%**	**1.01 [-1.74 , 3.76]**
Test for overall effect: T = 1.58, df = 2 (P = 0.25)								
Heterogeneity: Tau² (REML [b]) = 1.17; Chi² = 49.25, df = 2 (P < 0.00001); I² = 97%								
**1.10.4 Pharmacy**								
Ali 2025	44.93	10.97	44	45.18	11.3	44	4.7%	-0.02 [-0.44 , 0.40]
**Subtotal**			**44**			**44**	**4.7%**	**-0.02 [-0.44 , 0.40]**
Test for overall effect: Z = 0.10 (P = 0.92)								
Heterogeneity: Not applicable								
**1.10.5 Physiotherapy**								
Ferrer-Peña 2025	77	14.07	4	77	17.04	23	3.0%	0.00 [-1.06 , 1.06]
Sahin 2025	74.2	3.9	19	73.2	6.8	16	4.1%	0.18 [-0.49 , 0.85]
**Subtotal (HKSJ [a])**			**23**			**39**	**7.0%**	**0.13 [-0.90 , 1.16]**
Test for overall effect: T = 1.59, df = 1 (P = 0.36)								
Heterogeneity: Tau² (REML [b]) = 0.00; Chi² = 0.08, df = 1 (P = 0.78); I² = 0%								
**1.10.6 Health Sciences**								
Veras 2024	67.29	9.67	14	60.1	12.19	13	3.7%	0.64 [-0.14 , 1.41]
**Subtotal**			**14**			**13**	**3.7%**	**0.64 [-0.14 , 1.41]**
Test for overall effect: Z = 1.61 (P = 0.11)								
Heterogeneity: Not applicable								
**1.10.7 Optometry**								
**Subtotal**			**0**			**0**		**Not estimable**
Test for overall effect: Not applicable								

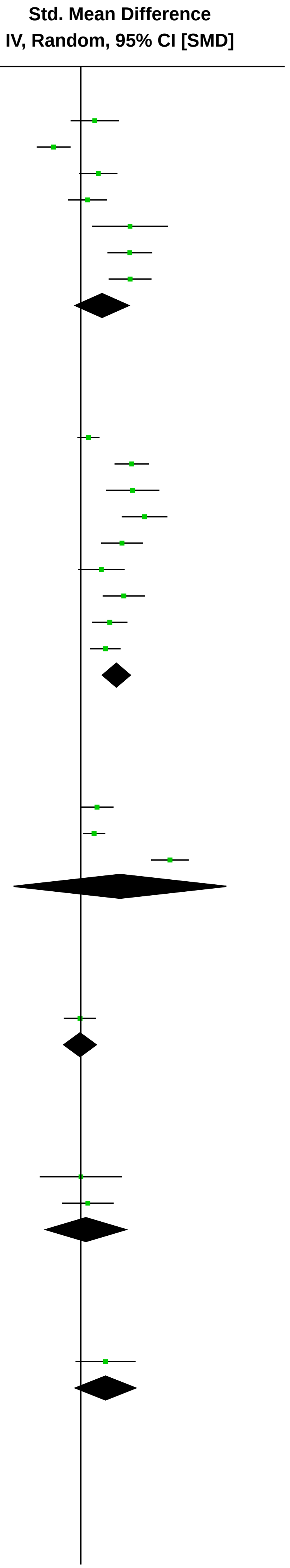

Heterogeneity: Not applicable				
**1.10.8 Others**				
**Subtotal**	**0**	**0**		**Not estimable**
Test for overall effect: Not applicable				
Heterogeneity: Not applicable				
**Total (HKSJ [a])**	**834**	**845**	**100.0%**	**0.71 [0.42 , 1.00]**
95% prediction interval				[-0.59 , 2.01]

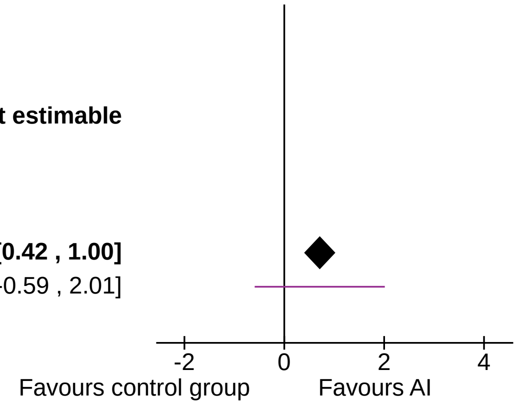

Test for overall effect: T = 5.09, df = 22 (P < 0.0001)
Test for subgroup differences: Chi² = 25.06, df = 5 (P = 0.0001), I² = 80.0%
Heterogeneity: Tau² (REML [b]) = 0.38; Chi² = 153.07, df = 22 (P < 0.00001); I² = 86%

Footnotes

[a] CI calculated by Hartung-Knapp-Sidik-Jonkman (HKSJ) method.

[b] Tau² calculated by Restricted Maximum-Likelihood method.

Analysis 1.11: Kirkpatrick level 1: perception or satisfaction (subgroup: region of study)

	AI			Control group				Std. Mean Difference
**Study or Subgroup**	**Mean [SMD]**	**SD [SMD]**	**Total**	**Mean [SMD]**	**SD [SMD]**	**Total**	**Weight**	**IV, Random, 95% CI [SMD]**
**1.11.1 Asia**								
Ali 2025	44.93	10.97	44	45.18	11.3	44	4.7%	-0.02 [-0.44 , 0.40]
Chen 2025	17.45	3.94	20	16.05	3.69	20	4.2%	0.36 [-0.27 , 0.98]
Han 2025	7.42	1.59	31	5.55	1.74	29	4.4%	1.11 [0.56 , 1.66]
Huang 2025	4.5	2.04	94	3.8	2.04	93	5.0%	0.34 [0.05 , 0.63]
Jiang 2024	20.6	3.3	31	20	3.6	30	4.5%	0.17 [-0.33 , 0.67]
Wang 2025a	3	1.48	28	1.5	0.74	28	4.3%	1.26 [0.69 , 1.84]
Wu 2024	17.23	1.33	31	15.38	1.54	30	4.4%	1.27 [0.72 , 1.82]
**Subtotal (HKSJ [a])**			**279**			**274**	**31.5%**	**0.62 [0.11 , 1.13]**
Test for overall effect: T = 2.98, df = 6 (P = 0.02)								
Heterogeneity: Tau² (REML [b]) = 0.24; Chi² = 27.93, df = 6 (P < 0.0001); I² = 80%								
**1.11.2 North America**								
Fang 2025	4.05	0.84	43	3.61	1.22	43	4.7%	0.42 [-0.01 , 0.84]
Veras 2024	67.29	9.67	14	60.1	12.19	13	3.7%	0.64 [-0.14 , 1.41]
**Subtotal (HKSJ [a])**			**57**			**56**	**8.5%**	**0.47 [-0.71 , 1.65]**
Test for overall effect: T = 5.03, df = 1 (P = 0.13)								
Heterogeneity: Tau² (REML [b]) = 0.00; Chi² = 0.24, df = 1 (P = 0.63); I² = 0%								
**1.11.3 South America**								
**Subtotal**			**0**			**0**		**Not estimable**
Test for overall effect: Not applicable								
Heterogeneity: Not applicable								
**1.11.4 Central America**								
**Subtotal**			**0**			**0**		**Not estimable**
Test for overall effect: Not applicable								
Heterogeneity: Not applicable								
**1.11.5 Africa**								
Benfatah 2024	8.9	0.7	20	7.8	0.9	20	4.0%	1.34 [0.64 , 2.03]
Benfatah 2026	44.8	3.2	30	38.1	4.7	30	4.3%	1.64 [1.05 , 2.24]
**Subtotal (HKSJ [a])**			**50**			**50**	**8.3%**	**1.52 [-0.41 , 3.44]**
Test for overall effect: T = 9.98, df = 1 (P = 0.06)								
Heterogeneity: Tau² (REML [b]) = 0.00; Chi² = 0.44, df = 1 (P = 0.51); I² = 0%								
**1.11.6 Europe**								
Akutay 2024	9.08	1.45	94	8.8	1.41	94	5.0%	0.19 [-0.09 , 0.48]
Arkan 2025	7.25	1.08	48	5.52	1.5	48	4.7%	1.31 [0.87 , 1.76]
Çiçek 2024	4.62	1.44	39	5.53	1.14	47	4.7%	-0.70 [-1.14 , -0.26]
Coşkun 2024	4.44	0.61	34	4.1	0.88	30	4.5%	0.45 [-0.05 , 0.95]
Doner 2025	9.86	0.36	33	8.78	1.43	28	4.4%	1.06 [0.52 , 1.60]
Ferrer-Peña 2025	77	14.07	4	77	17.04	23	3.0%	0.00 [-1.06 , 1.06]
Kestel 2025	4.68	1.85	38	3.31	1.79	41	4.6%	0.75 [0.29 , 1.20]
Ng 2025	4	0.94	10	2.6	1.16	10	3.2%	1.27 [0.29 , 2.25]
Sahin 2025	74.2	3.9	19	73.2	6.8	16	4.1%	0.18 [-0.49 , 0.85]
Simsek-Cetinkaya 2023	56.59	6.27	52	50.45	12.19	51	4.8%	0.63 [0.23 , 1.03]
Yilmaz 2025	4.7	0.3	55	3.3	0.8	55	4.6%	2.30 [1.82 , 2.79]
**Subtotal (HKSJ [a])**			**426**			**443**	**47.5%**	**0.68 [0.14 , 1.22]**
Test for overall effect: T = 2.79, df = 10 (P = 0.02)								
Heterogeneity: Tau² (REML [b]) = 0.58; Chi² = 106.92, df = 10 (P < 0.00001); I² = 91%								
**1.11.7 Oceania**								
**Subtotal**			**0**			**0**		**Not estimable**
Test for overall effect: Not applicable								
Heterogeneity: Not applicable								

Std. Mean Difference
IV, Random, 95% CI [SMD]

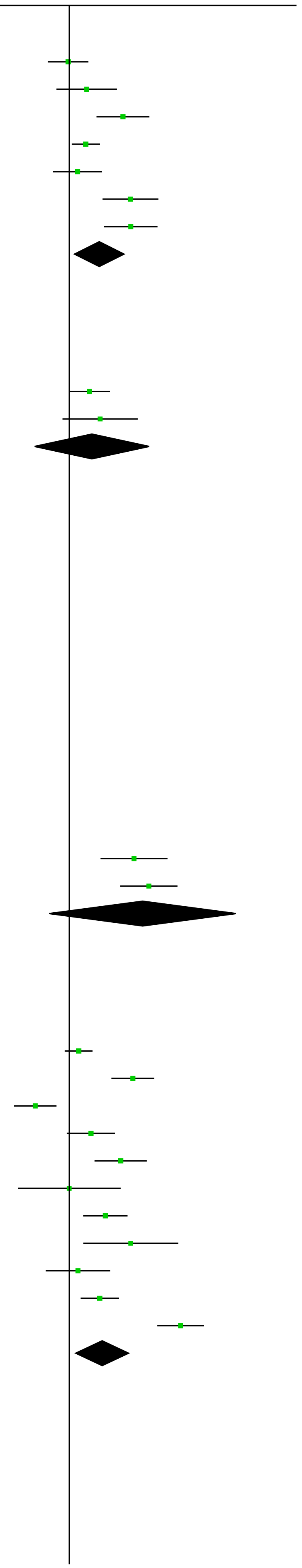

**1.11.8 Multi-continents**								
Fung 2025	8.45	8.25	22	4	8.25	22	4.2%	0.53 [-0.07 , 1.13]
**Subtotal**			**22**			**22**	**4.2%**	**0.53 [-0.07 , 1.13]**
Test for overall effect: Z = 1.72 (P = 0.08)								
Heterogeneity: Not applicable								
**Total (HKSJ [a])**			**834**			**845**	**100.0%**	**0.71 [0.42 , 1.00]**
95% prediction interval								[-0.59 , 2.01]

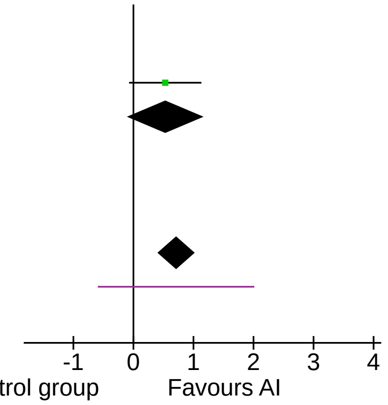

Test for overall effect: T = 5.09, df = 22 (P < 0.0001)
Test for subgroup differences: Chi² = 35.44, df = 4 (P < 0.00001), I² = 88.7%
Heterogeneity: Tau² (REML [b]) = 0.38; Chi² = 153.07, df = 22 (P < 0.00001); I² = 86%

Footnotes
[a] CI calculated by Hartung-Knapp-Sidik-Jonkman (HKSJ) method.
[b] Tau² calculated by Restricted Maximum-Likelihood method.

Analysis 1.12: Kirkpatrick level 1: perception or satisfaction (subgroup: LLM vs non-LLM applications)

Study or Subgroup	AI Mean [SMD]	AI SD [SMD]	AI Total	Control group Mean [SMD]	Control group SD [SMD]	Control group Total	Weight	Std. Mean Difference IV, Random, 95% CI [SMD]
**1.12.1 LLM**								
Akutay 2024	9.08	1.45	94	8.8	1.41	94	5.0%	0.19 [-0.09 , 0.48]
Ali 2025	44.93	10.97	44	45.18	11.3	44	4.7%	-0.02 [-0.44 , 0.40]
Benfatah 2024	8.9	0.7	20	7.8	0.9	20	4.0%	1.34 [0.64 , 2.03]
Benfatah 2026	44.8	3.2	30	38.1	4.7	30	4.3%	1.64 [1.05 , 2.24]
Çiçek 2024	4.62	1.44	39	5.53	1.14	47	4.7%	-0.70 [-1.14 , -0.26]
Coşkun 2024	4.44	0.61	34	4.1	0.88	30	4.5%	0.45 [-0.05 , 0.95]
Doner 2025	9.86	0.36	33	8.78	1.43	28	4.4%	1.06 [0.52 , 1.60]
Ferrer-Peña 2025	77	14.07	4	77	17.04	23	3.0%	0.00 [-1.06 , 1.06]
Fung 2025	8.45	8.25	22	4	8.25	22	4.2%	0.53 [-0.07 , 1.13]
Huang 2025	4.5	2.04	94	3.8	2.04	93	5.0%	0.34 [0.05 , 0.63]
Ng 2025	4	0.94	10	2.6	1.16	10	3.2%	1.27 [0.29 , 2.25]
Sahin 2025	74.2	3.9	19	73.2	6.8	16	4.1%	0.18 [-0.49 , 0.85]
Veras 2024	67.29	9.67	14	60.1	12.19	13	3.7%	0.64 [-0.14 , 1.41]
Wang 2025a	3	1.48	28	1.5	0.74	28	4.3%	1.26 [0.69 , 1.84]
Wu 2024	17.23	1.33	31	15.38	1.54	30	4.4%	1.27 [0.72 , 1.82]
Yilmaz 2025	4.7	0.3	55	3.3	0.8	55	4.6%	2.30 [1.82 , 2.79]
**Subtotal (HKSJ [a])**			**571**			**583**	**68.1%**	**0.73 [0.32 , 1.14]**
Test for overall effect: T = 3.78, df = 15 (P = 0.002)								
Heterogeneity: Tau² (REML [b]) = 0.52; Chi² = 134.98, df = 15 (P < 0.00001); I² = 89%								
**1.12.2 Non-LLM**								
Arkan 2025	7.25	1.08	48	5.52	1.5	48	4.7%	1.31 [0.87 , 1.76]
Chen 2025	17.45	3.94	20	16.05	3.69	20	4.2%	0.36 [-0.27 , 0.98]
Fang 2025	4.05	0.84	43	3.61	1.22	43	4.7%	0.42 [-0.01 , 0.84]
Han 2025	7.42	1.59	31	5.55	1.74	29	4.4%	1.11 [0.56 , 1.66]
Jiang 2024	20.6	3.3	31	20	3.6	30	4.5%	0.17 [-0.33 , 0.67]
Kestel 2025	4.68	1.85	38	3.31	1.79	41	4.6%	0.75 [0.29 , 1.20]
Simsek-Cetinkaya 2023	56.59	6.27	52	50.45	12.19	51	4.8%	0.63 [0.23 , 1.03]
**Subtotal (HKSJ [a])**			**263**			**262**	**31.9%**	**0.69 [0.31 , 1.06]**
Test for overall effect: T = 4.42, df = 6 (P = 0.004)								
Heterogeneity: Tau² (REML [b]) = 0.11; Chi² = 16.74, df = 6 (P = 0.01); I² = 65%								
**Total (HKSJ [a])**			**834**			**845**	**100.0%**	**0.71 [0.42 , 1.00]**
95% prediction interval								[-0.59 , 2.01]

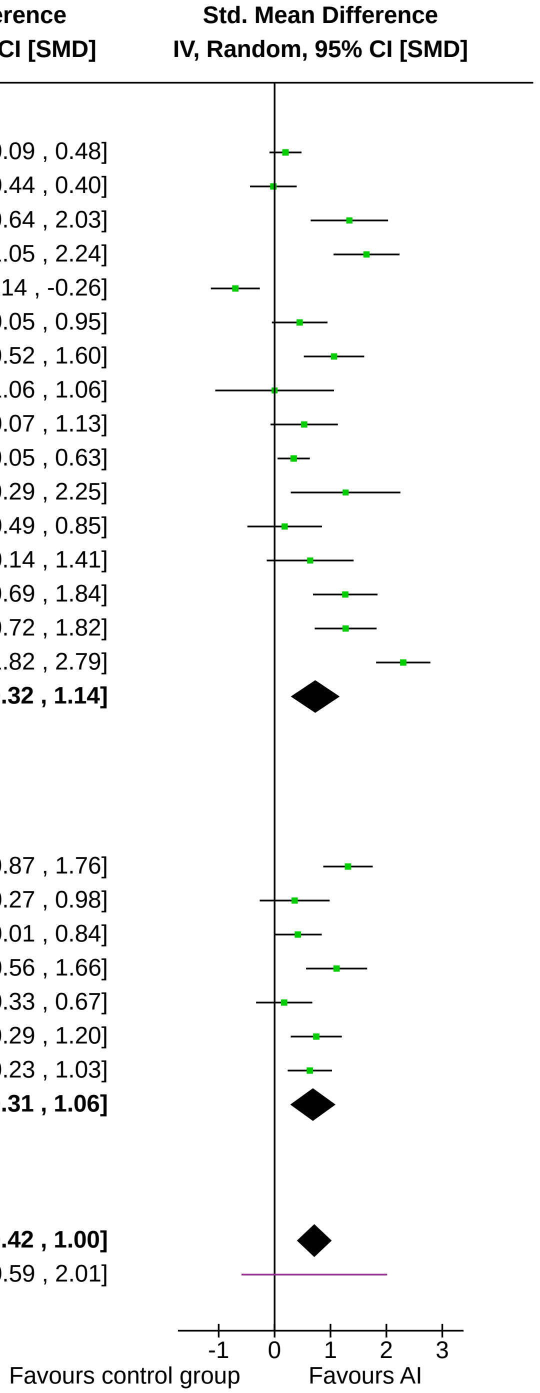

Test for overall effect: T = 5.09, df = 22 (P < 0.0001)
Test for subgroup differences: Chi² = 0.03, df = 1 (P = 0.87), I² = 0%
Heterogeneity: Tau² (REML [b]) = 0.38; Chi² = 153.07, df = 22 (P < 0.00001); I² = 86%

Footnotes

[a] CI calculated by Hartung-Knapp-Sidik-Jonkman (HKSJ) method.

[b] Tau² calculated by Restricted Maximum-Likelihood method.

Analysis 1.13: Kirkpatrick level 1: perception or satisfaction (subgroup: main function of application - teaching leaning vs assessment)

Study or Subgroup	AI Mean [SMD]	AI SD [SMD]	AI Total	Control group Mean [SMD]	Control group SD [SMD]	Control group Total	Weight	Std. Mean Difference IV, Random, 95% CI [SMD]
**1.13.1 Teaching and learning**								
Akutay 2024	9.08	1.45	94	8.8	1.41	94	5.0%	0.19 [-0.09 , 0.48]
Ali 2025	44.93	10.97	44	45.18	11.3	44	4.7%	-0.02 [-0.44 , 0.40]
Arkan 2025	7.25	1.08	48	5.52	1.5	48	4.7%	1.31 [0.87 , 1.76]
Benfatah 2024	8.9	0.7	20	7.8	0.9	20	4.0%	1.34 [0.64 , 2.03]
Benfatah 2026	44.8	3.2	30	38.1	4.7	30	4.3%	1.64 [1.05 , 2.24]
Chen 2025	17.45	3.94	20	16.05	3.69	20	4.2%	0.36 [-0.27 , 0.98]
Çiçek 2024	4.62	1.44	39	5.53	1.14	47	4.7%	-0.70 [-1.14 , -0.26]
Doner 2025	9.86	0.36	33	8.78	1.43	28	4.4%	1.06 [0.52 , 1.60]
Fang 2025	4.05	0.84	43	3.61	1.22	43	4.7%	0.42 [-0.01 , 0.84]
Ferrer-Peña 2025	77	14.07	4	77	17.04	23	3.0%	0.00 [-1.06 , 1.06]
Fung 2025	8.45	8.25	22	4	8.25	22	4.2%	0.53 [-0.07 , 1.13]
Han 2025	7.42	1.59	31	5.55	1.74	29	4.4%	1.11 [0.56 , 1.66]
Huang 2025	4.5	2.04	94	3.8	2.04	93	5.0%	0.34 [0.05 , 0.63]
Jiang 2024	20.6	3.3	31	20	3.6	30	4.5%	0.17 [-0.33 , 0.67]
Kestel 2025	4.68	1.85	38	3.31	1.79	41	4.6%	0.75 [0.29 , 1.20]
Ng 2025	4	0.94	10	2.6	1.16	10	3.2%	1.27 [0.29 , 2.25]
Sahin 2025	74.2	3.9	19	73.2	6.8	16	4.1%	0.18 [-0.49 , 0.85]
Simsek-Cetinkaya 2023	56.59	6.27	52	50.45	12.19	51	4.8%	0.63 [0.23 , 1.03]
Veras 2024	67.29	9.67	14	60.1	12.19	13	3.7%	0.64 [-0.14 , 1.41]
Wang 2025a	3	1.48	28	1.5	0.74	28	4.3%	1.26 [0.69 , 1.84]
Wu 2024	17.23	1.33	31	15.38	1.54	30	4.4%	1.27 [0.72 , 1.82]
Yilmaz 2025	4.7	0.3	55	3.3	0.8	55	4.6%	2.30 [1.82 , 2.79]
**Subtotal (HKSJ [a])**			**800**			**815**	**95.5%**	**0.72 [0.42 , 1.03]**
Test for overall effect: T = 4.97, df = 21 (P < 0.0001)								
Heterogeneity: Tau² (REML [b]) = 0.39; Chi² = 152.67, df = 21 (P < 0.00001); I² = 86%								
**1.13.2 Assessment**								
**Subtotal**			**0**			**0**		**Not estimable**
Test for overall effect: Not applicable								
Heterogeneity: Not applicable								
**1.13.3 Teaching-learning and assessment**								
Coşkun 2024	4.44	0.61	34	4.1	0.88	30	4.5%	0.45 [-0.05 , 0.95]
**Subtotal**			**34**			**30**	**4.5%**	**0.45 [-0.05 , 0.95]**
Test for overall effect: Z = 1.77 (P = 0.08)								
Heterogeneity: Not applicable								
**Total (HKSJ [a])**			**834**			**845**	**100.0%**	**0.71 [0.42 , 1.00]**
95% prediction interval								[-0.59 , 2.01]

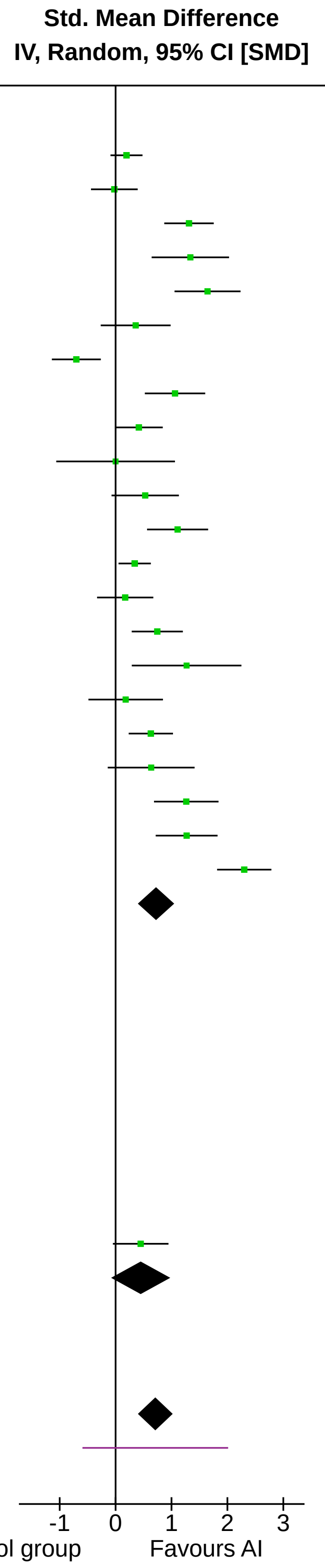

Test for overall effect: T = 5.09, df = 22 (P < 0.0001)
Test for subgroup differences: Chi² = 0.88, df = 1 (P = 0.35), I² = 0%
Heterogeneity: Tau² (REML [b]) = 0.38; Chi² = 153.07, df = 22 (P < 0.00001); I² = 86%

Footnotes
[a] CI calculated by Hartung-Knapp-Sidik-Jonkman (HKSJ) method.
[b] Tau² calculated by Restricted Maximum-Likelihood method.

Analysis 1.14: Kirkpatrick level 1: perception or satisfaction (subgroup: single vs multiple sessions)

Study or Subgroup	AI Mean [SMD]	AI SD [SMD]	AI Total	Control group Mean [SMD]	Control group SD [SMD]	Control group Total	Weight	Std. Mean Difference IV, Random, 95% CI [SMD]
**1.14.1 Single session**								
Benfatah 2026	44.8	3.2	30	38.1	4.7	30	4.5%	1.64 [1.05 , 2.24]
Fang 2025	4.05	0.84	43	3.61	1.22	43	4.9%	0.42 [-0.01 , 0.84]
Jiang 2024	20.6	3.3	31	20	3.6	30	4.7%	0.17 [-0.33 , 0.67]
Ng 2025	4	0.94	10	2.6	1.16	10	3.4%	1.27 [0.29 , 2.25]
Simsek-Cetinkaya 2023	56.59	6.27	52	50.45	12.19	51	5.0%	0.63 [0.23 , 1.03]
**Subtotal (HKSJ [a])**			**166**			**164**	**22.5%**	**0.77 [0.02 , 1.53]**
Test for overall effect: T = 2.86, df = 4 (P = 0.05)								
Heterogeneity: Tau² (REML [b]) = 0.28; Chi² = 17.06, df = 4 (P = 0.002); I² = 80%								
**1.14.2 Multiple sessions**								
Akutay 2024	9.08	1.45	94	8.8	1.41	94	5.2%	0.19 [-0.09 , 0.48]
Ali 2025	44.93	10.97	44	45.18	11.3	44	4.9%	-0.02 [-0.44 , 0.40]
Arkan 2025	7.25	1.08	48	5.52	1.5	48	4.9%	1.31 [0.87 , 1.76]
Benfatah 2024	8.9	0.7	20	7.8	0.9	20	4.2%	1.34 [0.64 , 2.03]
Chen 2025	17.45	3.94	20	16.05	3.69	20	4.4%	0.36 [-0.27 , 0.98]
Çiçek 2024	4.62	1.44	39	5.53	1.14	47	4.9%	-0.70 [-1.14 , -0.26]
Coşkun 2024	4.44	0.61	34	4.1	0.88	30	4.7%	0.45 [-0.05 , 0.95]
Doner 2025	9.86	0.36	33	8.78	1.43	28	4.6%	1.06 [0.52 , 1.60]
Ferrer-Peña 2025	77	14.07	4	77	17.04	23	3.1%	0.00 [-1.06 , 1.06]
Fung 2025	8.45	8.25	22	4	8.25	22	4.4%	0.53 [-0.07 , 1.13]
Huang 2025	4.5	2.04	94	3.8	2.04	93	5.2%	0.34 [0.05 , 0.63]
Kestel 2025	4.68	1.85	38	3.31	1.79	41	4.8%	0.75 [0.29 , 1.20]
Sahin 2025	74.2	3.9	19	73.2	6.8	16	4.3%	0.18 [-0.49 , 0.85]
Veras 2024	67.29	9.67	14	60.1	12.19	13	3.9%	0.64 [-0.14 , 1.41]
Wang 2025a	3	1.48	28	1.5	0.74	28	4.5%	1.26 [0.69 , 1.84]
Wu 2024	17.23	1.33	31	15.38	1.54	30	4.6%	1.27 [0.72 , 1.82]
Yilmaz 2025	4.7	0.3	55	3.3	0.8	55	4.8%	2.30 [1.82 , 2.79]
**Subtotal (HKSJ [a])**			**637**			**652**	**77.5%**	**0.67 [0.30 , 1.03]**
Test for overall effect: T = 3.85, df = 16 (P = 0.001)								
Heterogeneity: Tau² (REML [b]) = 0.44; Chi² = 132.13, df = 16 (P < 0.00001); I² = 88%								
**Total (HKSJ [a])**			**803**			**816**	**100.0%**	**0.69 [0.39 , 0.99]**
95% prediction interval								[-0.64 , 2.02]

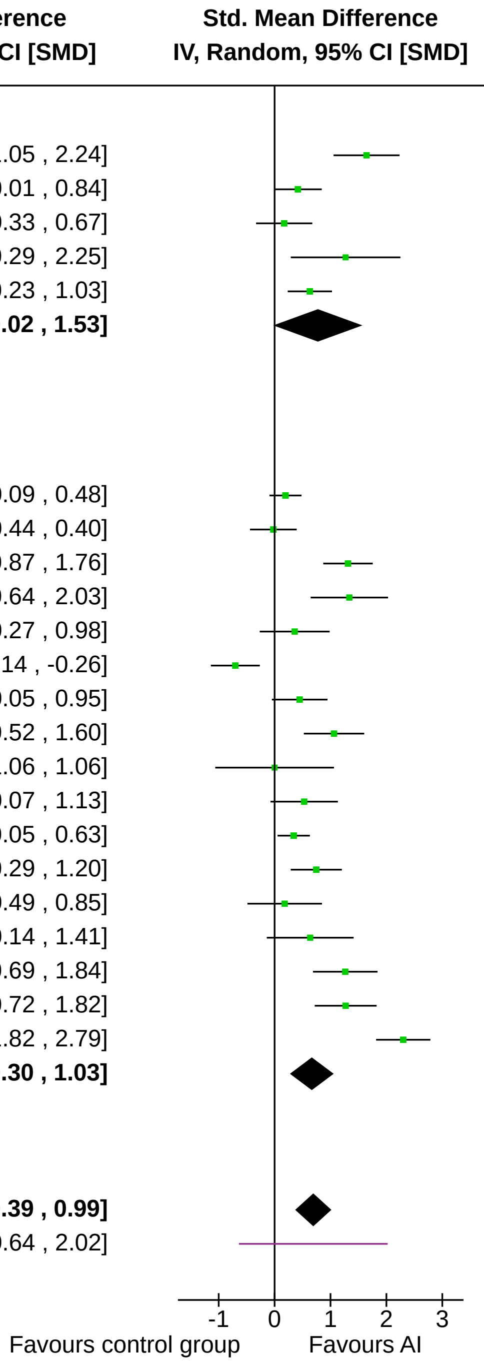

Test for overall effect: T = 4.78, df = 21 (P < 0.0001)
Test for subgroup differences: Chi² = 0.11, df = 1 (P = 0.74), I² = 0%
Heterogeneity: Tau² (REML [b]) = 0.39; Chi² = 149.69, df = 21 (P < 0.00001); I² = 86%

Footnotes

[a] CI calculated by Hartung-Knapp-Sidik-Jonkman (HKSJ) method.
[b] Tau² calculated by Restricted Maximum-Likelihood method.

Analysis 1.15: Kirkpatrick level 1: self-efficacy and confidence (LLM personalised learning aid vs control)

	LLM personalised learning aid			Control group				Std. Mean Difference
Study or Subgroup	Mean [SMD]	SD [SMD]	Total	Mean [SMD]	SD [SMD]	Total	Weight	IV, Random, 95% CI [SMD]
Aneesh 2025	4.6	0.6	103	3.9	0.8	103	20.8%	0.99 [0.70 , 1.28]
Benfatah 2024	4.6	0.6	20	3.9	0.8	20	11.9%	0.97 [0.31 , 1.63]
Benfatah 2026	8.1	0.9	30	7.1	1.4	30	14.7%	0.84 [0.31 , 1.37]
Huang 2025	4.5	2.04	94	3.8	2.04	93	20.8%	0.34 [0.05 , 0.63]
Ng 2025	3.4	0.84	10	2.5	0.97	10	7.7%	0.95 [0.01 , 1.89]
Sahin 2025	19.8	11.7	19	-3.9	15	16	9.6%	1.74 [0.95 , 2.53]
Wu 2024	9.14	0.54	31	8.46	0.7	30	14.4%	1.08 [0.54 , 1.62]
**Total (HKSJ [a])**			**307**			**302**	**100.0%**	**0.91 [0.54 , 1.29]**
95% prediction interval								[0.05 , 1.77]

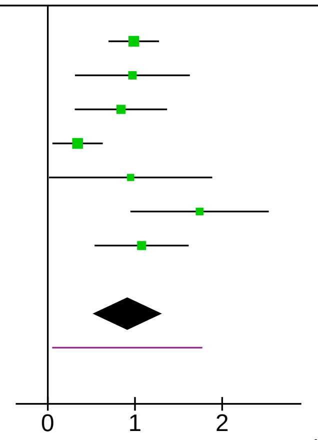

Test for overall effect: T = 5.96, df = 6 (P = 0.0010)

Heterogeneity: Tau² (REML [b]) = 0.10; Chi² = 18.05, df = 6 (P = 0.006); I² = 64%

Footnotes

[a] CI calculated by Hartung-Knapp-Sidik-Jonkman (HKSJ) method.

[b] Tau² calculated by Restricted Maximum-Likelihood method.

Analysis 1.16: Kirkpatrick level 1: self-efficacy and confidence (LLM virtual patient vs control)

	LLM virtual patient			Control group				Std. Mean Difference
Study or Subgroup	Mean [SMD]	SD [SMD]	Total	Mean [SMD]	SD [SMD]	Total	Weight	IV, Random, 95% CI [SMD]
Fung 2025	198.77	26.31	22	182.18	26.31	22	50.4%	0.62 [0.01 , 1.23]
Wang 2025a	4	1.48	28	1.5	0.74	28	49.6%	2.11 [1.44 , 2.77]
**Total (HKSJ [a])**			**50**			**50**	**100.0%**	**1.36 [-8.09 , 10.81]**
95% prediction interval								[-14.49 , 17.20]

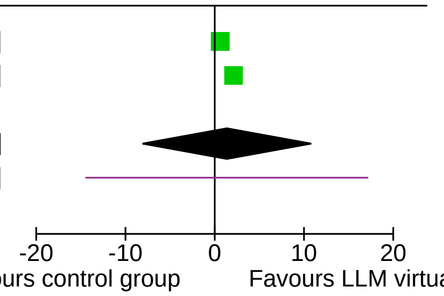

Test for overall effect: T = 1.82, df = 1 (P = 0.32)

Heterogeneity: Tau² (REML [b]) = 1.00; Chi² = 10.55, df = 1 (P = 0.001); I² = 91%

Footnotes

[a] CI calculated by Hartung-Knapp-Sidik-Jonkman (HKSJ) method.

[b] Tau² calculated by Restricted Maximum-Likelihood method.

Analysis 1.17: Kirkpatrick level 1: self-efficacy and confidence (LLM-integrated curriculum vs control)

	LLM-integrated curriculum			Control group				Std. Mean Difference
Study or Subgroup	Mean [SMD]	SD [SMD]	Total	Mean [SMD]	SD [SMD]	Total	Weight	IV, Random, 95% CI [SMD]
Arkan 2025	7.25	1.08	48	5.63	1.5	48	100.0%	1.23 [0.79 , 1.67]
**Total**			**48**			**48**	**100.0%**	**1.23 [0.79 , 1.67]**
95% prediction interval								[0.79 , 1.67]

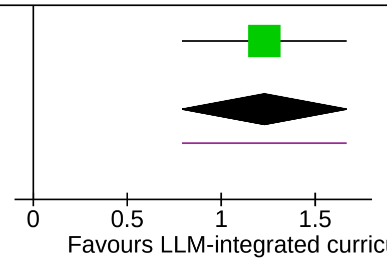

Test for overall effect: Z = 5.51 (P < 0.00001)

Heterogeneity: Not applicable

Analysis 1.18: Kirkpatrick level 1: self-efficacy and confidence (non-LLM AI procedure assistant vs control)

Study or Subgroup	AI procedure assistant (non-LLM) Mean [SMD]	SD [SMD]	Total	Control group Mean [SMD]	SD [SMD]	Total	Weight	Std. Mean Difference IV, Random, 95% CI [SMD]
Chang 2024	4.45	0.69	20	4.45	0.52	20	100.0%	0.00 [-0.62 , 0.62]
**Total**			**20**			**20**	**100.0%**	**0.00 [-0.62 , 0.62]**
95% prediction interval								[-0.62 , 0.62]

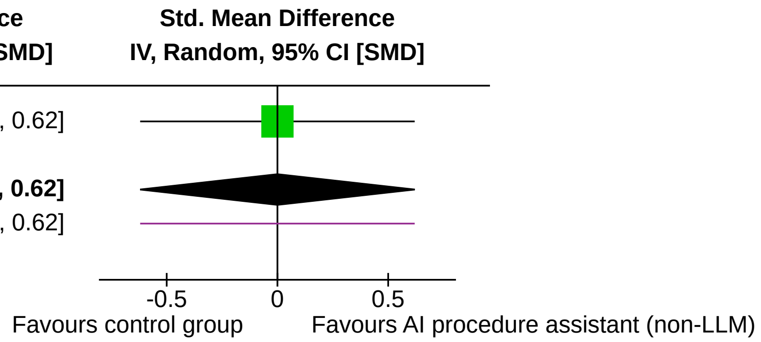

Test for overall effect: Z = 0.00 (P = 1.00)

Heterogeneity: Not applicable

Analysis 1.19: Kirkpatrick level 1: self-efficacy and confidence (non-LLM AI moderated adaptive learning platform vs control)

Study or Subgroup	AI-moderated adaptive learning platform (non-LLM) Mean [SMD]	SD [SMD]	Total	Control group Mean [SMD]	SD [SMD]	Total	Weight	Std. Mean Difference IV, Random, 95% CI [SMD]
Chen 2025	16.05	3.36	20	7.4	3.57	20	100.0%	2.45 [1.61 , 3.28]
**Total**			**20**			**20**	**100.0%**	**2.45 [1.61 , 3.28]**
95% prediction interval								[1.61 , 3.28]

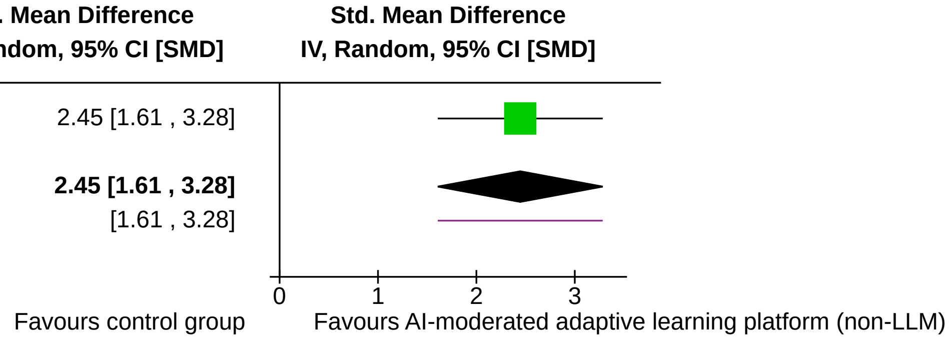

Test for overall effect: Z = 5.72 (P < 0.00001)

Heterogeneity: Not applicable

Analysis 1.20: Kirkpatrick level 1: self-efficacy and confidence (NLP rule-based chatbot vs control)

Study or Subgroup	NLP rule-based chatbot (non-LLM) Mean [SMD]	SD [SMD]	Total	Control group Mean [SMD]	SD [SMD]	Total	Weight	Std. Mean Difference IV, Random, 95% CI [SMD]
Fang 2025	3.8	1	43	2.9	1.6	43	56.5%	0.67 [0.23 , 1.10]
Han 2025	56.1	9.26	31	47.21	5.51	29	43.5%	1.14 [0.59 , 1.69]
**Total (HKSJ [a])**			**74**			**72**	**100.0%**	**0.87 [-2.11 , 3.86]**
95% prediction interval								[-3.21 , 4.96]

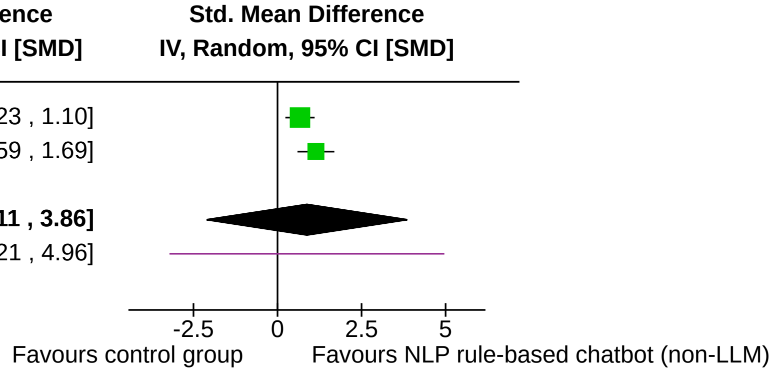

Test for overall effect: T = 3.72, df = 1 (P = 0.17)

Heterogeneity: Tau² (REML [b]) = 0.05; Chi² = 1.76, df = 1 (P = 0.18); I² = 43%

Footnotes

[a] CI calculated by Hartung-Knapp-Sidik-Jonkman (HKSJ) method.

[b] Tau² calculated by Restricted Maximum-Likelihood method.

Analysis 1.21: Kirkpatrick level 1: self-efficacy and confidence (non-LLM AI-VR virtual doctor vs control)

Study or Subgroup	AI-VR virtual doctor (non-LLM) Mean [SMD]	SD [SMD]	Total	Control group Mean [SMD]	SD [SMD]	Total	Weight	Std. Mean Difference IV, Random, 95% CI [SMD]
Liaw 2023	60.1	13.3	32	69.6	14.4	32	100.0%	-0.68 [-1.18 , -0.17]
**Total**			**32**			**32**	**100.0%**	**-0.68 [-1.18 , -0.17]**
95% prediction interval								[-1.18 , -0.17]

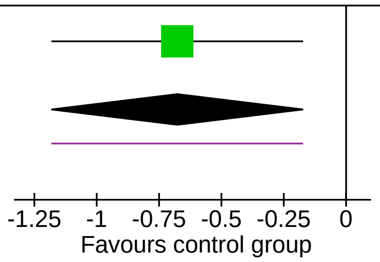

Test for overall effect: Z = 2.63 (P = 0.009)

Heterogeneity: Not applicable

Analysis 1.22: Kirkpatrick level 1: self-efficacy and confidence (AL procedure assistant + adaptive learning platform vs control)

Study or Subgroup	AI procedure assistant + AI-moderated adaptive learning platform Mean [SMD]	SD [SMD]	Total	Control group Mean [SMD]	SD [SMD]	Total	Weight	Std. Mean Difference IV, Random, 95% CI [SMD]
Usta 2025	86.6	7.85	10	81.7	9.06	10	100.0%	0.55 [-0.34 , 1.45]
**Total**			**10**			**10**	**100.0%**	**0.55 [-0.34 , 1.45]**
95% prediction interval								[-0.34 , 1.45]

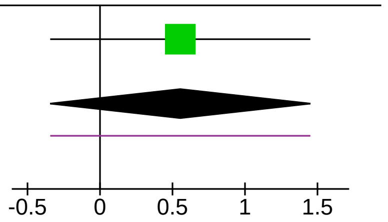

Test for overall effect: Z = 1.21 (P = 0.23)

Heterogeneity: Not applicable

Analysis 1.23: Kirkpatrick level 1: self-efficacy and confidence (subgroup: field of study)

Study or Subgroup	AI Mean [SMD]	AI SD [SMD]	AI Total	Control group Mean [SMD]	Control group SD [SMD]	Control group Total	Weight	Std. Mean Difference IV, Random, 95% CI [SMD]
**1.23.1 Medicine**								
Aneesh 2025	4.6	0.6	103	3.9	0.8	103	7.2%	0.99 [0.70 , 1.28]
Chen 2025	16.05	3.36	20	7.4	3.57	20	5.4%	2.45 [1.61 , 3.28]
Ng 2025	3.4	0.84	10	2.5	0.97	10	5.0%	0.95 [0.01 , 1.89]
Wang 2025a	4	1.48	28	1.5	0.74	28	6.1%	2.11 [1.44 , 2.77]
Wu 2024	9.14	0.54	31	8.46	0.7	30	6.5%	1.08 [0.54 , 1.62]
**Subtotal (HKSJ [a])**			**192**			**191**	**30.2%**	**1.48 [0.62 , 2.33]**
Test for overall effect: T = 4.78, df = 4 (P = 0.009)								
Heterogeneity: Tau² (REML [b]) = 0.35; Chi² = 18.20, df = 4 (P = 0.001); I² = 80%								
**1.23.2 Nursing**								
Arkan 2025	7.25	1.08	48	5.63	1.5	48	6.8%	1.23 [0.79 , 1.67]
Benfatah 2024	4.6	0.6	20	3.9	0.8	20	6.1%	0.97 [0.31 , 1.63]
Benfatah 2026	8.1	0.9	30	7.1	1.4	30	6.5%	0.84 [0.31 , 1.37]
Fung 2025	198.77	26.31	22	182.18	26.31	22	6.3%	0.62 [0.01 , 1.23]
Han 2025	56.1	9.26	31	47.21	5.51	29	6.5%	1.14 [0.59 , 1.69]
Liaw 2023	60.1	13.3	32	69.6	14.4	32	6.6%	-0.68 [-1.18 , -0.17]
**Subtotal (HKSJ [a])**			**183**			**181**	**38.8%**	**0.68 [-0.06 , 1.43]**
Test for overall effect: T = 2.36, df = 5 (P = 0.06)								
Heterogeneity: Tau² (REML [b]) = 0.44; Chi² = 37.68, df = 5 (P < 0.00001); I² = 85%								
**1.23.3 Dentistry**								
Chang 2024	4.45	0.69	20	4.45	0.52	20	6.2%	0.00 [-0.62 , 0.62]
Fang 2025	3.8	1	43	2.9	1.6	43	6.8%	0.67 [0.23 , 1.10]
Huang 2025	4.5	2.04	94	3.8	2.04	93	7.2%	0.34 [0.05 , 0.63]
Usta 2025	86.6	7.85	10	81.7	9.06	10	5.2%	0.55 [-0.34 , 1.45]
**Subtotal (HKSJ [a])**			**167**			**166**	**25.5%**	**0.40 [0.01 , 0.78]**
Test for overall effect: T = 3.25, df = 3 (P = 0.05)								
Heterogeneity: Tau² (REML [b]) = 0.01; Chi² = 3.33, df = 3 (P = 0.34); I² = 9%								
**1.23.4 Pharmacy**								
**Subtotal**			**0**			**0**		**Not estimable**
Test for overall effect: Not applicable								
Heterogeneity: Not applicable								
**1.23.5 Physiotherapy**								
Sahin 2025	19.8	11.7	19	-3.9	15	16	5.6%	1.74 [0.95 , 2.53]
**Subtotal**			**19**			**16**	**5.6%**	**1.74 [0.95 , 2.53]**
Test for overall effect: Z = 4.30 (P < 0.0001)								
Heterogeneity: Not applicable								
**1.23.6 Health Sciences**								
**Subtotal**			**0**			**0**		**Not estimable**
Test for overall effect: Not applicable								
Heterogeneity: Not applicable								
**1.23.7 Optometry**								
**Subtotal**			**0**			**0**		**Not estimable**
Test for overall effect: Not applicable								
Heterogeneity: Not applicable								
**1.23.8 Others**								
**Subtotal**			**0**			**0**		**Not estimable**
Test for overall effect: Not applicable								
Heterogeneity: Not applicable								

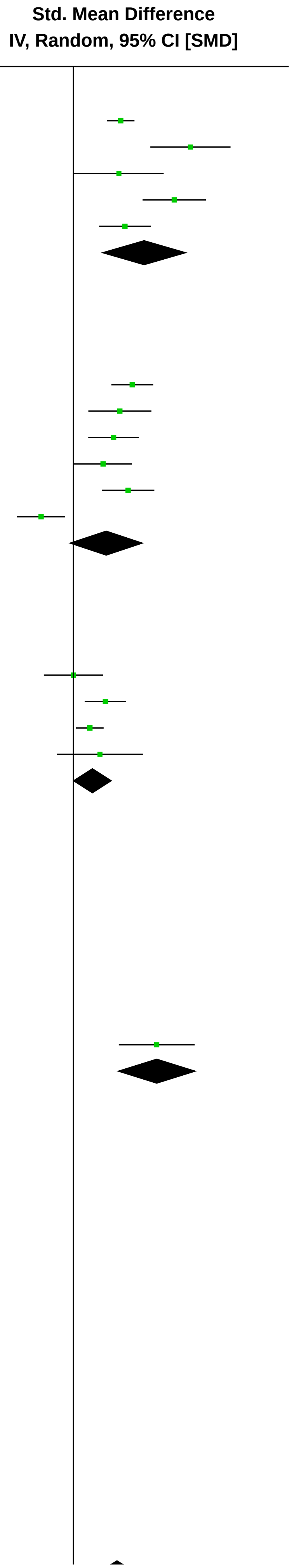

**Total (HKSJ [a])**	**561**	**554**	**100.0%**	**0.91 [0.51 , 1.31]**
95% prediction interval				[-0.57 , 2.39]

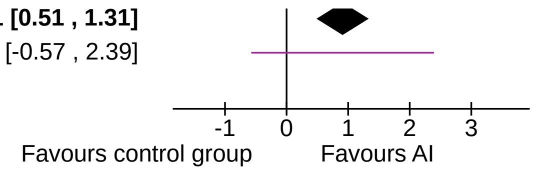

Test for overall effect: T = 4.87, df = 15 (P = 0.0002)
Test for subgroup differences: Chi² = 18.79, df = 3 (P = 0.0003), I² = 84.0%
Heterogeneity: Tau² (REML [b]) = 0.45; Chi² = 93.26, df = 15 (P < 0.00001); I² = 87%

Footnotes
[a] CI calculated by Hartung-Knapp-Sidik-Jonkman (HKSJ) method.
[b] Tau² calculated by Restricted Maximum-Likelihood method.

Analysis 1.24: Kirkpatrick level 1: self-efficacy and confidence (subgroup: region of study)

Study or Subgroup	AI Mean [SMD]	AI SD [SMD]	AI Total	Control group Mean [SMD]	Control group SD [SMD]	Control group Total	Weight	Std. Mean Difference IV, Random, 95% CI [SMD]
**1.24.1 Asia**								
Aneesh 2025	4.6	0.6	103	3.9	0.8	103	7.2%	0.99 [0.70 , 1.28]
Chen 2025	16.05	3.36	20	7.4	3.57	20	5.4%	2.45 [1.61 , 3.28]
Han 2025	56.1	9.26	31	47.21	5.51	29	6.5%	1.14 [0.59 , 1.69]
Huang 2025	4.5	2.04	94	3.8	2.04	93	7.2%	0.34 [0.05 , 0.63]
Liaw 2023	60.1	13.3	32	69.6	14.4	32	6.6%	-0.68 [-1.18 , -0.17]
Wang 2025a	4	1.48	28	1.5	0.74	28	6.1%	2.11 [1.44 , 2.77]
Wu 2024	9.14	0.54	31	8.46	0.7	30	6.5%	1.08 [0.54 , 1.62]
**Subtotal (HKSJ [a])**			**339**			**335**	**45.5%**	**1.03 [0.07 , 1.98]**
Test for overall effect: T = 2.63, df = 6 (P = 0.04)								
Heterogeneity: Tau^2 (REML [b]) = 0.97; Chi^2 = 76.16, df = 6 (P < 0.00001); I^2 = 95%								
**1.24.2 North America**								
Chang 2024	4.45	0.69	20	4.45	0.52	20	6.2%	0.00 [-0.62 , 0.62]
Fang 2025	3.8	1	43	2.9	1.6	43	6.8%	0.67 [0.23 , 1.10]
**Subtotal (HKSJ [a])**			**63**			**63**	**13.0%**	**0.37 [-3.85 , 4.59]**
Test for overall effect: T = 1.12, df = 1 (P = 0.46)								
Heterogeneity: Tau^2 (REML [b]) = 0.15; Chi^2 = 2.99, df = 1 (P = 0.08); I^2 = 67%								
**1.24.3 South America**								
**Subtotal**			**0**			**0**		**Not estimable**
Test for overall effect: Not applicable								
Heterogeneity: Not applicable								
**1.24.4 Central America**								
**Subtotal**			**0**			**0**		**Not estimable**
Test for overall effect: Not applicable								
Heterogeneity: Not applicable								
**1.24.5 Africa**								
Benfatah 2024	4.6	0.6	20	3.9	0.8	20	6.1%	0.97 [0.31 , 1.63]
Benfatah 2026	8.1	0.9	30	7.1	1.4	30	6.5%	0.84 [0.31 , 1.37]
**Subtotal (HKSJ [a])**			**50**			**50**	**12.6%**	**0.89 [0.07 , 1.71]**
Test for overall effect: T = 13.86, df = 1 (P = 0.05)								
Heterogeneity: Tau^2 (REML [b]) = 0.00; Chi^2 = 0.09, df = 1 (P = 0.76); I^2 = 0%								
**1.24.6 Europe**								
Arkan 2025	7.25	1.08	48	5.63	1.5	48	6.8%	1.23 [0.79 , 1.67]
Ng 2025	3.4	0.84	10	2.5	0.97	10	5.0%	0.95 [0.01 , 1.89]
Sahin 2025	19.8	11.7	19	-3.9	15	16	5.6%	1.74 [0.95 , 2.53]
Usta 2025	86.6	7.85	10	81.7	9.06	10	5.2%	0.55 [-0.34 , 1.45]
**Subtotal (HKSJ [a])**			**87**			**84**	**22.6%**	**1.19 [0.54 , 1.83]**
Test for overall effect: T = 5.86, df = 3 (P = 0.010)								
Heterogeneity: Tau^2 (REML [b]) = 0.01; Chi^2 = 4.07, df = 3 (P = 0.25); I^2 = 6%								
**1.24.7 Oceania**								
**Subtotal**			**0**			**0**		**Not estimable**
Test for overall effect: Not applicable								
Heterogeneity: Not applicable								
**1.24.8 Multi-continents**								
Fung 2025	198.77	26.31	22	182.18	26.31	22	6.3%	0.62 [0.01 , 1.23]
**Subtotal**			**22**			**22**	**6.3%**	**0.62 [0.01 , 1.23]**
Test for overall effect: Z = 2.00 (P = 0.05)								
Heterogeneity: Not applicable								

Std. Mean Difference
IV, Random, 95% CI [SMD]

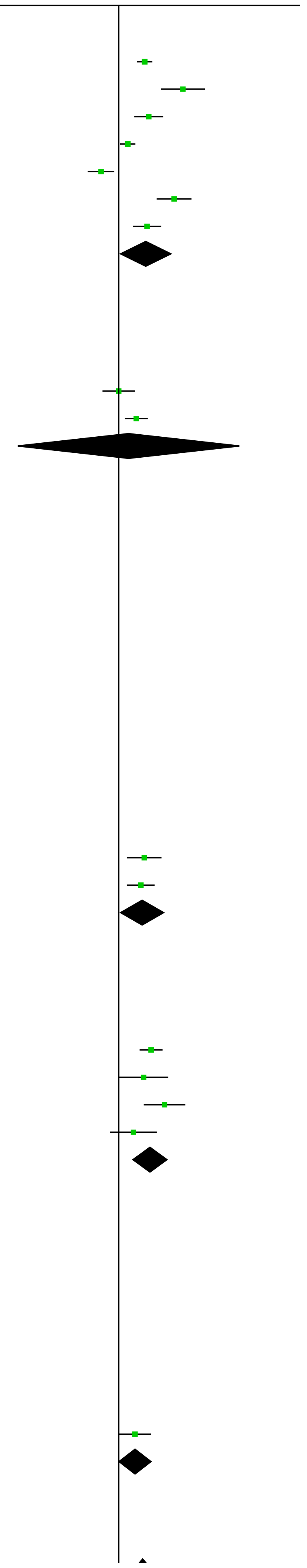

**Total (HKSJ [a])**			**561**			**554**	**100.0%**	**0.91 [0.51 , 1.31]**
95% prediction interval								[-0.57 , 2.39]

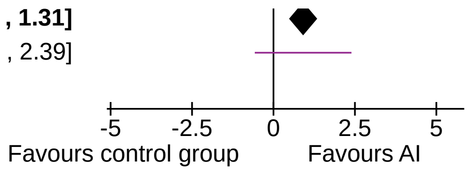

Test for overall effect: T = 4.87, df = 15 (P = 0.0002)
Test for subgroup differences: Chi² = 5.48, df = 4 (P = 0.24), I^2 = 27.0%
Heterogeneity: Tau² (REML [b]) = 0.45; Chi² = 93.26, df = 15 (P < 0.00001); I^2 = 87%

Footnotes
[a] CI calculated by Hartung-Knapp-Sidik-Jonkman (HKSJ) method.
[b] Tau² calculated by Restricted Maximum-Likelihood method.

Analysis 1.25: Kirkpatrick level 1: self-efficacy and confidence (subgroup: LLM vs non-LLM)

	AI			Control group				Std. Mean Difference
**Study or Subgroup**	**Mean [SMD]**	**SD [SMD]**	**Total**	**Mean [SMD]**	**SD [SMD]**	**Total**	**Weight**	**IV, Random, 95% CI [SMD]**
**1.25.1 LLM**								
Aneesh 2025	4.6	0.6	103	3.9	0.8	103	7.2%	0.99 [0.70 , 1.28]
Benfatah 2024	4.6	0.6	20	3.9	0.8	20	6.1%	0.97 [0.31 , 1.63]
Benfatah 2026	8.1	0.9	30	7.1	1.4	30	6.5%	0.84 [0.31 , 1.37]
Fung 2025	198.77	26.31	22	182.18	26.31	22	6.3%	0.62 [0.01 , 1.23]
Huang 2025	4.5	2.04	94	3.8	2.04	93	7.2%	0.34 [0.05 , 0.63]
Ng 2025	3.4	0.84	10	2.5	0.97	10	5.0%	0.95 [0.01 , 1.89]
Sahin 2025	19.8	11.7	19	-3.9	15	16	5.6%	1.74 [0.95 , 2.53]
Wang 2025a	4	1.48	28	1.5	0.74	28	6.1%	2.11 [1.44 , 2.77]
Wu 2024	9.14	0.54	31	8.46	0.7	30	6.5%	1.08 [0.54 , 1.62]
**Subtotal (HKSJ [a])**			**357**			**352**	**56.5%**	**1.02 [0.61 , 1.44]**
Test for overall effect: T = 5.72, df = 8 (P = 0.0004)								
Heterogeneity: Tau² (REML [b]) = 0.20; Chi² = 32.84, df = 8 (P < 0.0001); I^2 = 76%								
**1.25.2 Non-LLM**								
Arkan 2025	7.25	1.08	48	5.63	1.5	48	6.8%	1.23 [0.79 , 1.67]
Chang 2024	4.45	0.69	20	4.45	0.52	20	6.2%	0.00 [-0.62 , 0.62]
Chen 2025	16.05	3.36	20	7.4	3.57	20	5.4%	2.45 [1.61 , 3.28]
Fang 2025	3.8	1	43	2.9	1.6	43	6.8%	0.67 [0.23 , 1.10]
Han 2025	56.1	9.26	31	47.21	5.51	29	6.5%	1.14 [0.59 , 1.69]
Liaw 2023	60.1	13.3	32	69.6	14.4	32	6.6%	-0.68 [-1.18 , -0.17]
Usta 2025	86.6	7.85	10	81.7	9.06	10	5.2%	0.55 [-0.34 , 1.45]
**Subtotal (HKSJ [a])**			**204**			**202**	**43.5%**	**0.75 [-0.16 , 1.65]**
Test for overall effect: T = 2.01, df = 6 (P = 0.09)								
Heterogeneity: Tau² (REML [b]) = 0.84; Chi² = 58.27, df = 6 (P < 0.00001); I^2 = 91%								
**Total (HKSJ [a])**			**561**			**554**	**100.0%**	**0.91 [0.51 , 1.31]**
95% prediction interval								[-0.57 , 2.39]

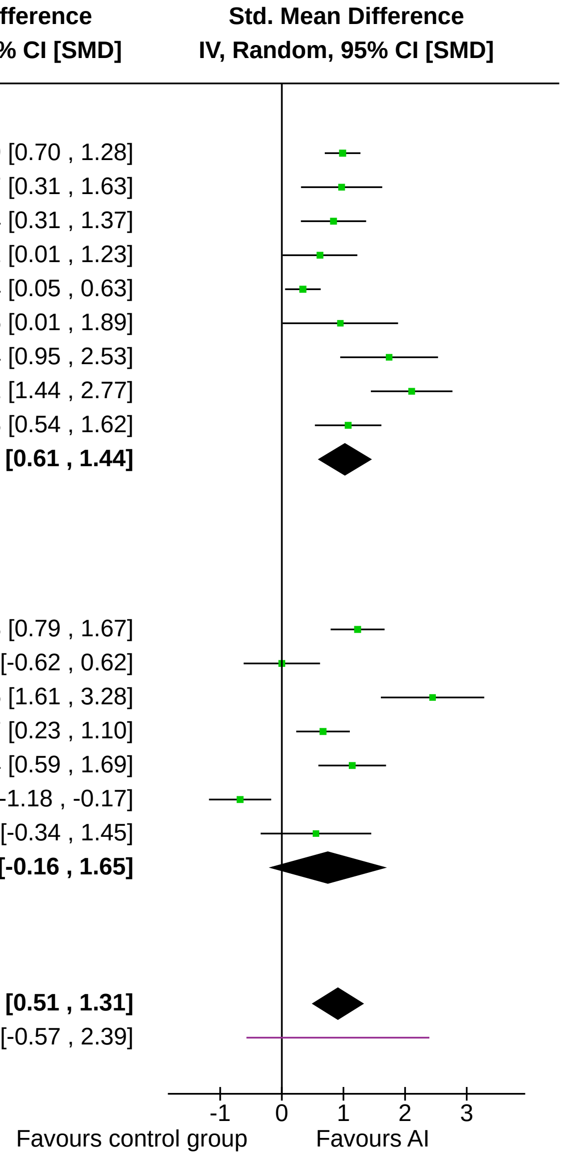

Test for overall effect: T = 4.87, df = 15 (P = 0.0002)
Test for subgroup differences: Chi² = 0.45, df = 1 (P = 0.50), I^2 = 0%
Heterogeneity: Tau² (REML [b]) = 0.45; Chi² = 93.26, df = 15 (P < 0.00001); I^2 = 87%

Footnotes
[a] CI calculated by Hartung-Knapp-Sidik-Jonkman (HKSJ) method.
[b] Tau² calculated by Restricted Maximum-Likelihood method.

Analysis 1.26: Kirkpatrick level 1: self-efficacy and confidence (subgroup: main function of application- teaching learning vs assessment)

Study or Subgroup	AI Mean [SMD]	AI SD [SMD]	AI Total	Control group Mean [SMD]	Control group SD [SMD]	Control group Total	Weight	Std. Mean Difference IV, Random, 95% CI [SMD]
**1.26.1 Teaching and learning**								
Aneesh 2025	4.6	0.6	103	3.9	0.8	103	7.2%	0.99 [0.70 , 1.28]
Arkan 2025	7.25	1.08	48	5.63	1.5	48	6.8%	1.23 [0.79 , 1.67]
Benfatah 2024	4.6	0.6	20	3.9	0.8	20	6.1%	0.97 [0.31 , 1.63]
Benfatah 2026	8.1	0.9	30	7.1	1.4	30	6.5%	0.84 [0.31 , 1.37]
Chang 2024	4.45	0.69	20	4.45	0.52	20	6.2%	0.00 [-0.62 , 0.62]
Chen 2025	16.05	3.36	20	7.4	3.57	20	5.4%	2.45 [1.61 , 3.28]
Fang 2025	3.8	1	43	2.9	1.6	43	6.8%	0.67 [0.23 , 1.10]
Fung 2025	198.77	26.31	22	182.18	26.31	22	6.3%	0.62 [0.01 , 1.23]
Han 2025	56.1	9.26	31	47.21	5.51	29	6.5%	1.14 [0.59 , 1.69]
Huang 2025	4.5	2.04	94	3.8	2.04	93	7.2%	0.34 [0.05 , 0.63]
Ng 2025	3.4	0.84	10	2.5	0.97	10	5.0%	0.95 [0.01 , 1.89]
Sahin 2025	19.8	11.7	19	-3.9	15	16	5.6%	1.74 [0.95 , 2.53]
Usta 2025	86.6	7.85	10	81.7	9.06	10	5.2%	0.55 [-0.34 , 1.45]
Wang 2025a	4	1.48	28	1.5	0.74	28	6.1%	2.11 [1.44 , 2.77]
Wu 2024	9.14	0.54	31	8.46	0.7	30	6.5%	1.08 [0.54 , 1.62]
**Subtotal (HKSJ [a])**			**529**			**522**	**93.4%**	**1.01 [0.67 , 1.35]**
Test for overall effect: T = 6.32, df = 14 (P < 0.0001)								
Heterogeneity: Tau² (REML [b]) = 0.27; Chi² = 58.80, df = 14 (P < 0.00001); I² = 80%								
**1.26.2 Assessment**								
**Subtotal**			**0**			**0**		**Not estimable**
Test for overall effect: Not applicable								
Heterogeneity: Not applicable								
**1.26.3 Teaching-learning and assessment**								
Liaw 2023	60.1	13.3	32	69.6	14.4	32	6.6%	-0.68 [-1.18 , -0.17]
**Subtotal**			**32**			**32**	**6.6%**	**-0.68 [-1.18 , -0.17]**
Test for overall effect: Z = 2.63 (P = 0.009)								
Heterogeneity: Not applicable								
**Total (HKSJ [a])**			**561**			**554**	**100.0%**	**0.91 [0.51 , 1.31]**
95% prediction interval								[-0.57 , 2.39]

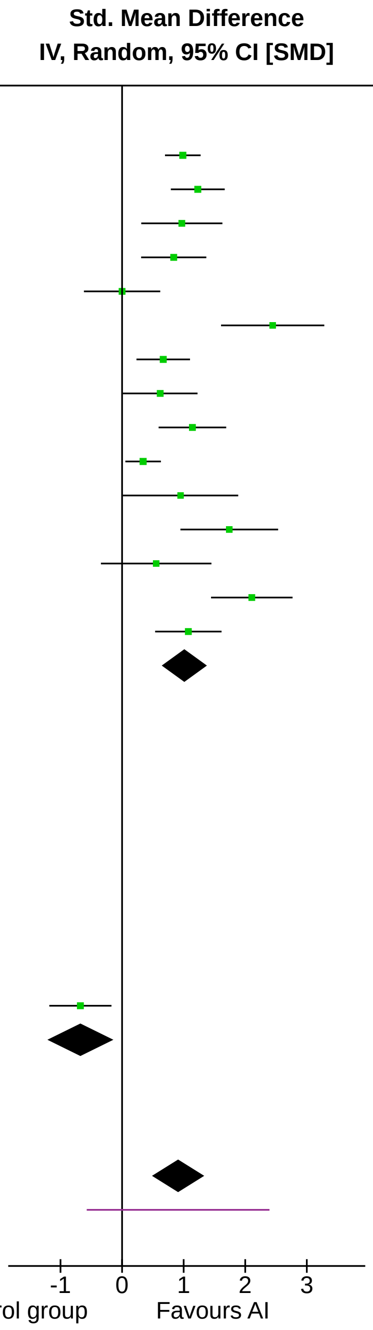

Test for overall effect: T = 4.87, df = 15 (P = 0.0002)
Test for subgroup differences: Chi² = 30.99, df = 1 (P < 0.00001), I² = 96.8%
Heterogeneity: Tau² (REML [b]) = 0.45; Chi² = 93.26, df = 15 (P < 0.00001); I² = 87%

Footnotes
[a] CI calculated by Hartung-Knapp-Sidik-Jonkman (HKSJ) method.
[b] Tau² calculated by Restricted Maximum-Likelihood method.

Analysis 1.27: Kirkpatrick level 1: self-efficacy and confidence (subgroup: single vs multiple sessions)

Study or Subgroup	AI Mean [SMD]	AI SD [SMD]	AI Total	Control group Mean [SMD]	Control group SD [SMD]	Control group Total	Weight	Std. Mean Difference IV, Random, 95% CI [SMD]
**1.27.1 Single session**								
Benfatah 2026	8.1	0.9	30	7.1	1.4	30	7.0%	0.84 [0.31 , 1.37]
Chang 2024	4.45	0.69	20	4.45	0.52	20	6.6%	0.00 [-0.62 , 0.62]
Fang 2025	3.8	1	43	2.9	1.6	43	7.3%	0.67 [0.23 , 1.10]
Ng 2025	3.4	0.84	10	2.5	0.97	10	5.5%	0.95 [0.01 , 1.89]
**Subtotal (HKSJ [a])**			**103**			**103**	**26.3%**	**0.60 [-0.04 , 1.23]**
Test for overall effect: T = 2.99, df = 3 (P = 0.06)								
Heterogeneity: Tau² (REML [b]) = 0.06; Chi² = 5.01, df = 3 (P = 0.17); I² = 40%								
**1.27.2 Multiple sessions**								
Aneesh 2025	4.6	0.6	103	3.9	0.8	103	7.7%	0.99 [0.70 , 1.28]
Arkan 2025	7.25	1.08	48	5.63	1.5	48	7.3%	1.23 [0.79 , 1.67]
Benfatah 2024	4.6	0.6	20	3.9	0.8	20	6.5%	0.97 [0.31 , 1.63]
Chen 2025	16.05	3.36	20	7.4	3.57	20	5.8%	2.45 [1.61 , 3.28]
Fung 2025	198.77	26.31	22	182.18	26.31	22	6.7%	0.62 [0.01 , 1.23]
Huang 2025	4.5	2.04	94	3.8	2.04	93	7.7%	0.34 [0.05 , 0.63]
Liaw 2023	60.1	13.3	32	69.6	14.4	32	7.0%	-0.68 [-1.18 , -0.17]
Sahin 2025	19.8	11.7	19	-3.9	15	16	6.0%	1.74 [0.95 , 2.53]
Usta 2025	86.6	7.85	10	81.7	9.06	10	5.6%	0.55 [-0.34 , 1.45]
Wang 2025a	4	1.48	28	1.5	0.74	28	6.5%	2.11 [1.44 , 2.77]
Wu 2024	9.14	0.54	31	8.46	0.7	30	6.9%	1.08 [0.54 , 1.62]
**Subtotal (HKSJ [a])**			**427**			**422**	**73.7%**	**1.01 [0.43 , 1.58]**
Test for overall effect: T = 3.89, df = 10 (P = 0.003)								
Heterogeneity: Tau² (REML [b]) = 0.63; Chi² = 84.91, df = 10 (P < 0.00001); I² = 91%								
**Total (HKSJ [a])**			**530**			**525**	**100.0%**	**0.90 [0.47 , 1.32]**
95% prediction interval								[-0.66 , 2.45]

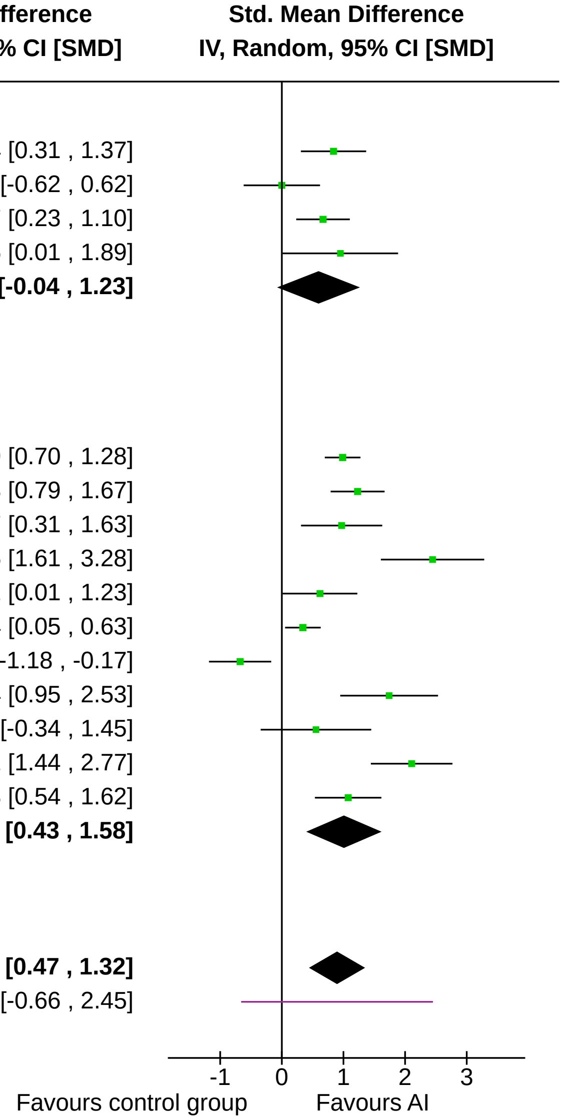

Test for overall effect: T = 4.49, df = 14 (P = 0.0005)
Test for subgroup differences: Chi² = 1.59, df = 1 (P = 0.21), I² = 37.0%
Heterogeneity: Tau² (REML [b]) = 0.49; Chi² = 91.57, df = 14 (P < 0.00001); I² = 88%

Footnotes
[a] CI calculated by Hartung-Knapp-Sidik-Jonkman (HKSJ) method.
[b] Tau² calculated by Restricted Maximum-Likelihood method.

Analysis 1.28: Kirkpatrick level 1: self-efficacy and confidence (proportion confident in echocardiography) (AI procedure assistant (non-LLM) vs control)

Study or Subgroup	AI procedure assistant (non-LLM) Events	AI procedure assistant (non-LLM) Total	Control group Events	Control group Total	Weight	Risk Ratio IV, Random, 95% CI
Lau 2025	6	21	5	22	100.0%	1.26 [0.45 , 3.50]
**Total**		**21**		**22**	**100.0%**	**1.26 [0.45 , 3.50]**
95% prediction interval						[0.45 , 3.50]
Total events:	6		5			
Test for overall effect: Z = 0.44 (P = 0.66)						
Heterogeneity: Not applicable						

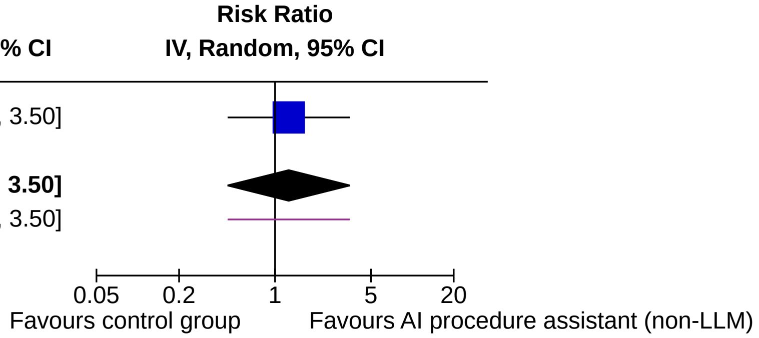

Analysis 1.29: Kirkpatrick level 2: theoretical knowledge (LLM content generator vs control)

Study or Subgroup	LLM content generator Mean [SMD]	SD [SMD]	Total	Control group Mean [SMD]	SD [SMD]	Total	Weight	Std. Mean Difference IV, Random, 95% CI [SMD]
Akutay 2024	7.43	1.58	94	6.9	1.74	94	34.7%	0.32 [0.03 , 0.61]
Doner 2025	67.87	12.68	33	57.85	12.85	28	32.2%	0.78 [0.25 , 1.30]
Yilmaz 2025	11.4	1.1	55	9	1.4	55	33.1%	1.89 [1.44 , 2.35]
**Total (HKSJ [a])**			**182**			**177**	**100.0%**	**0.99 [-1.04 , 3.01]**
95% prediction interval								[-2.96 , 4.93]

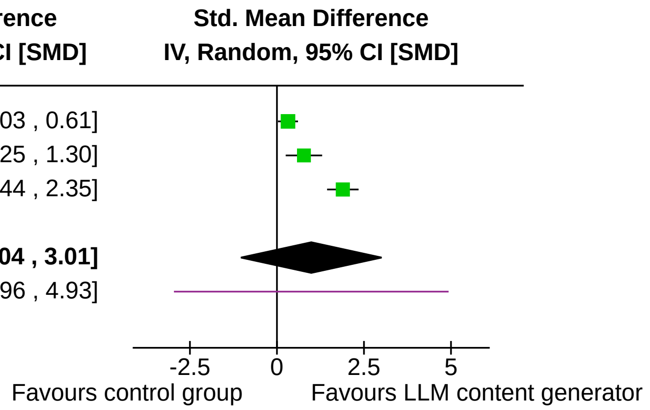

Test for overall effect: T = 2.10, df = 2 (P = 0.17)

Heterogeneity: Tau² (REML [b]) = 0.62; Chi² = 33.18, df = 2 (P < 0.00001); I² = 93%

Footnotes

[a] CI calculated by Hartung-Knapp-Sidik-Jonkman (HKSJ) method.

[b] Tau² calculated by Restricted Maximum-Likelihood method.

Analysis 1.30: Kirkpatrick level 2: theoretical knowledge (LLM gamification tool vs control)

Study or Subgroup	LLM gamification tool Mean [SMD]	SD [SMD]	Total	Control group Mean [SMD]	SD [SMD]	Total	Weight	Std. Mean Difference IV, Random, 95% CI [SMD]
Wang 2025	95.83	5.22	24	91.22	6.21	24	100.0%	0.79 [0.20 , 1.38]
**Total**			**24**			**24**	**100.0%**	**0.79 [0.20 , 1.38]**
95% prediction interval								[0.20 , 1.38]

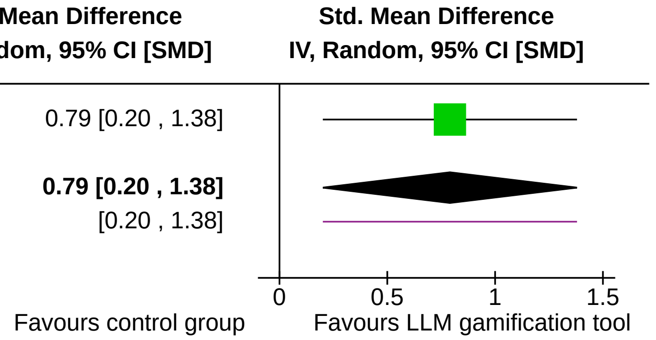

Test for overall effect: Z = 2.63 (P = 0.009)

Heterogeneity: Not applicable

Analysis 1.31: Kirkpatrick level 2: theoretical knowledge (LLM personalised learning aid vs control)

Study or Subgroup	LLM personalised learning aid Mean [SMD]	SD [SMD]	Total	Control group Mean [SMD]	SD [SMD]	Total	Weight	Std. Mean Difference IV, Random, 95% CI [SMD]
Aneesh 2025	12.83	3.47	103	13.18	3.36	103	9.9%	-0.10 [-0.38 , 0.17]
Digiacomo 2025	10	11.11	41	12	10.37	39	9.1%	-0.18 [-0.62 , 0.26]
Gan 2024	141.2	26.68	54	130.8	25.56	56	9.4%	0.40 [0.02 , 0.77]
Huang 2025	41.24	8.19	94	42.03	8.34	93	9.9%	-0.10 [-0.38 , 0.19]
Hui 2025	93.9	3.65	21	90.33	4.08	21	8.0%	0.90 [0.27 , 1.54]
Kalam 2025	6.2	1.93	10	4.36	2.01	11	6.4%	0.90 [-0.01 , 1.80]
Ng 2025	71.43	15.06	10	54.29	23.13	10	6.3%	0.84 [-0.08 , 1.76]
Ramsamooj 2025	75.2	3.2	21	71.1	4	41	8.4%	1.08 [0.52 , 1.64]
Sahin 2025	81.8	11.5	19	69.4	11.5	16	7.5%	1.05 [0.34 , 1.77]
Shi 2025	74.3	14.8	50	77.6	14.9	50	9.4%	-0.22 [-0.61 , 0.17]
Svendsen 2024	7.7	2	15	5.9	2.5	16	7.4%	0.77 [0.04 , 1.51]
Wu 2024	86.44	5.59	31	77.86	4.16	30	8.2%	1.71 [1.12 , 2.31]
**Total (HKSJ [a])**			**469**			**486**	**100.0%**	**0.53 [0.13 , 0.94]**
95% prediction interval								[-0.81 , 1.88]

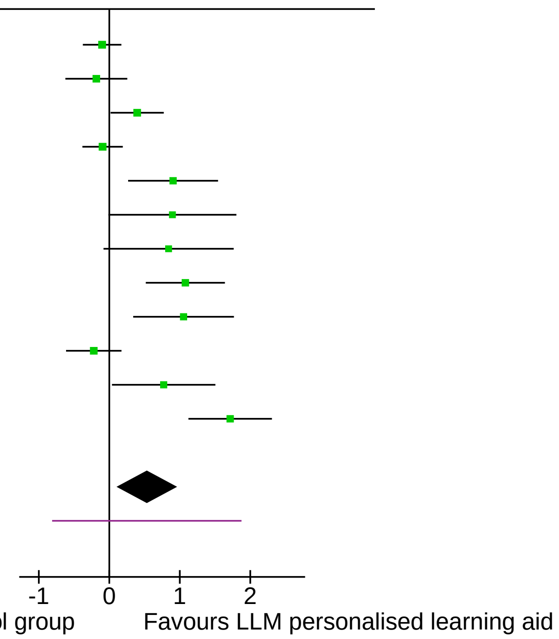

Test for overall effect: T = 2.90, df = 11 (P = 0.01)

Heterogeneity: Tau^2 (REML [b]) = 0.34; Chi^2 = 67.95, df = 11 (P < 0.00001); I^2 = 86%

Footnotes

[a] CI calculated by Hartung-Knapp-Sidik-Jonkman (HKSJ) method.

[b] Tau^2 calculated by Restricted Maximum-Likelihood method.

Analysis 1.32: Kirkpatrick level 2: theoretical knowledge (non-LLM AI moderated adaptive learning platform vs control)

Study or Subgroup	AI-moderated adaptive learning platform (non-LLM) Mean [SMD]	SD [SMD]	Total	Control group Mean [SMD]	SD [SMD]	Total	Weight	Std. Mean Difference IV, Random, 95% CI [SMD]
Chen 2025	84.47	3.48	20	81.72	4.37	20	100.0%	0.68 [0.04 , 1.32]
**Total**			**20**			**20**	**100.0%**	**0.68 [0.04 , 1.32]**
95% prediction interval								[0.04 , 1.32]

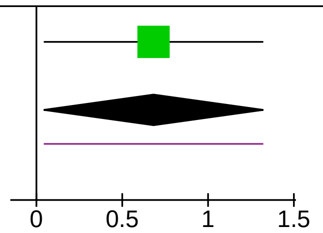

Test for overall effect: Z = 2.09 (P = 0.04)

Heterogeneity: Not applicable

Analysis 1.33: Kirkpatrick level 2: theoretical knowledge (NLP rule-based chatbot vs control)

Study or Subgroup	NLP rule-based chatbot (non-LLM) Mean [SMD]	SD [SMD]	Total	Control group Mean [SMD]	SD [SMD]	Total	Weight	Std. Mean Difference IV, Random, 95% CI [SMD]
Al-Kahf 2023	12.5	1.8	170	12.1	1.9	252	34.7%	0.21 [0.02 , 0.41]
Han 2025	7.32	1.22	31	6.72	1.33	29	33.5%	0.46 [-0.05 , 0.98]
Hsu 2024	82.69	8.46	24	61.48	7.4	24	31.7%	2.62 [1.84 , 3.41]
**Total (HKSJ [a])**			**225**			**305**	**100.0%**	**1.06 [-2.19 , 4.32]**
95% prediction interval								[-5.28 , 7.41]

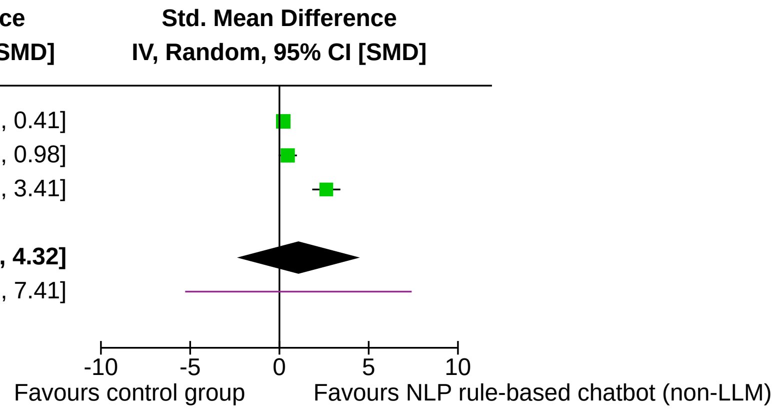

Test for overall effect: T = 1.41, df = 2 (P = 0.29)

Heterogeneity: Tau² (REML [b]) = 1.60; Chi² = 34.05, df = 2 (P < 0.00001); I² = 97%

Footnotes

[a] CI calculated by Hartung-Knapp-Sidik-Jonkman (HKSJ) method.

[b] Tau² calculated by Restricted Maximum-Likelihood method.

Analysis 1.34: Kirkpatrick level 2: theoretical knowledge (non-LLM AI imaging diagnostic aid vs control)

Study or Subgroup	AI imaging diagnostic aid (non LLM) Mean [SMD]	SD [SMD]	Total	Control group Mean [SMD]	SD [SMD]	Total	Weight	Std. Mean Difference IV, Random, 95% CI [SMD]
Li 2023	32.33	5.86	15	20.31	6.55	16	47.1%	1.88 [1.02 , 2.74]
Wu 2020	13.39	2.4	19	11.38	3.17	19	52.9%	0.70 [0.04 , 1.36]
**Total (HKSJ [a])**			**34**			**35**	**100.0%**	**1.26 [-6.23 , 8.74]**
95% prediction interval								[-10.73 , 13.24]

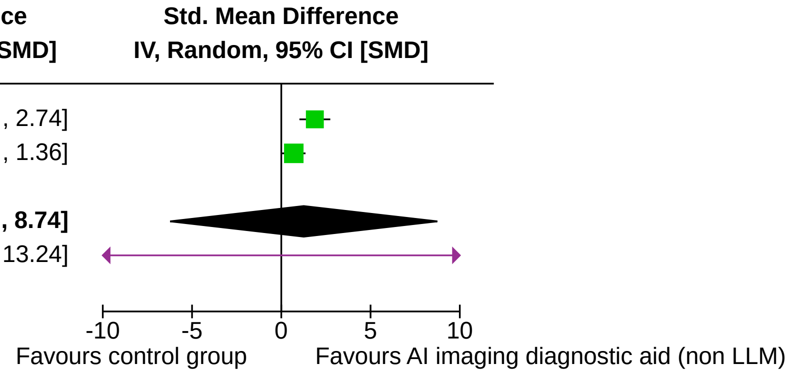

Test for overall effect: T = 2.13, df = 1 (P = 0.28)

Heterogeneity: Tau² (REML [b]) = 0.54; Chi² = 4.54, df = 1 (P = 0.03); I² = 78%

Footnotes

[a] CI calculated by Hartung-Knapp-Sidik-Jonkman (HKSJ) method.

[b] Tau² calculated by Restricted Maximum-Likelihood method.

Analysis 1.35: Kirkpatrick level 2: theoretical knowledge (non-LLM AI-VR virtual doctor vs control)

Study or Subgroup	AI-VR virtual doctor (non-LLM) Mean [SMD]	SD [SMD]	Total	Control group Mean [SMD]	SD [SMD]	Total	Weight	Std. Mean Difference IV, Random, 95% CI [SMD]
Liaw 2023	9.06	1.78	32	7.75	2.08	32	100.0%	0.67 [0.16 , 1.17]
**Total**			**32**			**32**	**100.0%**	**0.67 [0.16 , 1.17]**
95% prediction interval								[0.16 , 1.17]

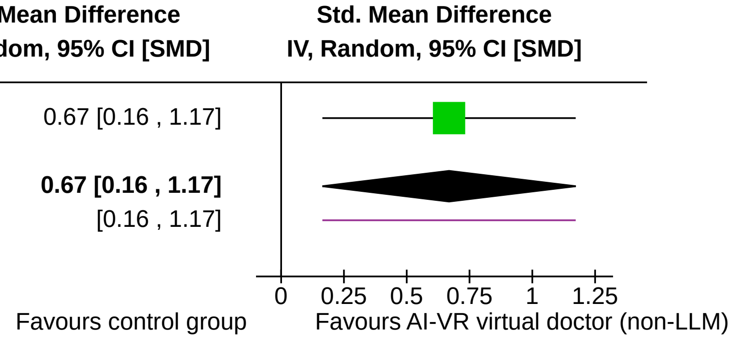

Test for overall effect: Z = 2.60 (P = 0.009)

Heterogeneity: Not applicable

Analysis 1.36: Kirkpatrick level 2: theoretical knowledge (proportion with grade A or B: non-LLM AI gamification tool vs control)

Study or Subgroup	AI gamification tool (non-LLM) Events	AI gamification tool (non-LLM) Total	Control group Events	Control group Total	Weight	Risk Ratio IV, Random, 95% CI
Mahrous 2023	31	36	24	37	100.0%	1.33 [1.01 , 1.74]
**Total**		**36**		**37**	**100.0%**	**1.33 [1.01 , 1.74]**
95% prediction interval						[1.01 , 1.74]
Total events:	31		24			
Test for overall effect: Z = 2.05 (P = 0.04)						
Heterogeneity: Not applicable						

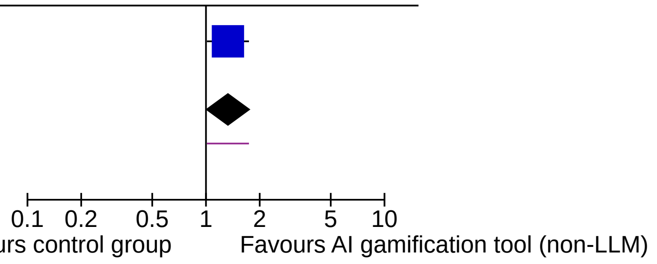

Analysis 1.37: Kirkpatrick level 2: theoretical knowledge (subgroup: field of study)

Study or Subgroup	AI Mean [SMD]	AI SD [SMD]	AI Total	Control group Mean [SMD]	Control group SD [SMD]	Control group Total	Weight	Std. Mean Difference IV, Random, 95% CI [SMD]
**1.37.1 Medicine**								
Al-Kahf 2023	12.5	1.8	170	12.1	1.9	252	4.9%	0.21 [0.02 , 0.41]
Aneesh 2025	12.83	3.47	103	13.18	3.36	103	4.8%	-0.10 [-0.38 , 0.17]
Chen 2025	84.47	3.48	20	81.72	4.37	20	4.0%	0.68 [0.04 , 1.32]
Digiacomo 2025	10	11.11	41	12	10.37	39	4.5%	-0.18 [-0.62 , 0.26]
Gan 2024	141.2	26.68	54	130.8	25.56	56	4.6%	0.40 [0.02 , 0.77]
Hui 2025	93.9	3.65	21	90.33	4.08	21	4.0%	0.90 [0.27 , 1.54]
Jiang 2024	15.87	3.4	31	13.2	3.57	30	4.3%	0.76 [0.24 , 1.28]
Kalam 2025	6.2	1.93	10	4.36	2.01	11	3.3%	0.90 [-0.01 , 1.80]
Li 2023	32.33	5.86	15	20.31	6.55	16	3.4%	1.88 [1.02 , 2.74]
Ng 2025	71.43	15.06	10	54.29	23.13	10	3.2%	0.84 [-0.08 , 1.76]
Ramsamooj 2025	75.2	3.2	21	71.1	4	41	4.2%	1.08 [0.52 , 1.64]
Wu 2020	13.39	2.4	19	11.38	3.17	19	3.9%	0.70 [0.04 , 1.36]
Wu 2024	86.44	5.59	31	77.86	4.16	30	4.1%	1.71 [1.12 , 2.31]
**Subtotal (HKSJ [a])**			**546**			**648**	**53.2%**	**0.69 [0.32 , 1.06]**
Test for overall effect: T = 4.10, df = 12 (P = 0.001)								
Heterogeneity: Tau² (REML [b]) = 0.29; Chi² = 66.65, df = 12 (P < 0.00001); I² = 84%								
**1.37.2 Nursing**								
Akutay 2024	7.43	1.58	94	6.9	1.74	94	4.8%	0.32 [0.03 , 0.61]
Doner 2025	67.87	12.68	33	57.85	12.85	28	4.3%	0.78 [0.25 , 1.30]
Han 2025	7.32	1.22	31	6.72	1.33	29	4.3%	0.46 [-0.05 , 0.98]
Hsu 2024	82.69	8.46	24	61.48	7.4	24	3.6%	2.62 [1.84 , 3.41]
Liaw 2023	9.06	1.78	32	7.75	2.08	32	4.3%	0.67 [0.16 , 1.17]
Shi 2025	74.3	14.8	50	77.6	14.9	50	4.6%	-0.22 [-0.61 , 0.17]
Wang 2025	95.83	5.22	24	91.22	6.21	24	4.1%	0.79 [0.20 , 1.38]
**Subtotal (HKSJ [a])**			**288**			**281**	**30.0%**	**0.73 [-0.06 , 1.52]**
Test for overall effect: T = 2.26, df = 6 (P = 0.06)								
Heterogeneity: Tau² (REML [b]) = 0.61; Chi² = 44.71, df = 6 (P < 0.00001); I² = 91%								
**1.37.3 Dentistry**								
Huang 2025	41.24	8.19	94	42.03	8.34	93	4.8%	-0.10 [-0.38 , 0.19]
Yilmaz 2025	11.4	1.1	55	9	1.4	55	4.5%	1.89 [1.44 , 2.35]
**Subtotal (HKSJ [a])**			**149**			**148**	**9.2%**	**0.89 [-11.74 , 13.52]**
Test for overall effect: T = 0.90, df = 1 (P = 0.53)								
Heterogeneity: Tau² (REML [b]) = 1.94; Chi² = 52.94, df = 1 (P < 0.00001); I² = 98%								
**1.37.4 Pharmacy**								
Svendsen 2024	7.7	2	15	5.9	2.5	16	3.7%	0.77 [0.04 , 1.51]
**Subtotal**			**15**			**16**	**3.7%**	**0.77 [0.04 , 1.51]**
Test for overall effect: Z = 2.06 (P = 0.04)								
Heterogeneity: Not applicable								
**1.37.5 Physiotherapy**								
Sahin 2025	81.8	11.5	19	69.4	11.5	16	3.8%	1.05 [0.34 , 1.77]
**Subtotal**			**19**			**16**	**3.8%**	**1.05 [0.34 , 1.77]**
Test for overall effect: Z = 2.89 (P = 0.004)								
Heterogeneity: Not applicable								
**1.37.6 Health Sciences**								
**Subtotal**			**0**			**0**		**Not estimable**
Test for overall effect: Not applicable								
Heterogeneity: Not applicable								
**1.37.7 Optometry**								
**Subtotal**			**0**			**0**		**Not estimable**

Std. Mean Difference IV, Random, 95% CI [SMD]

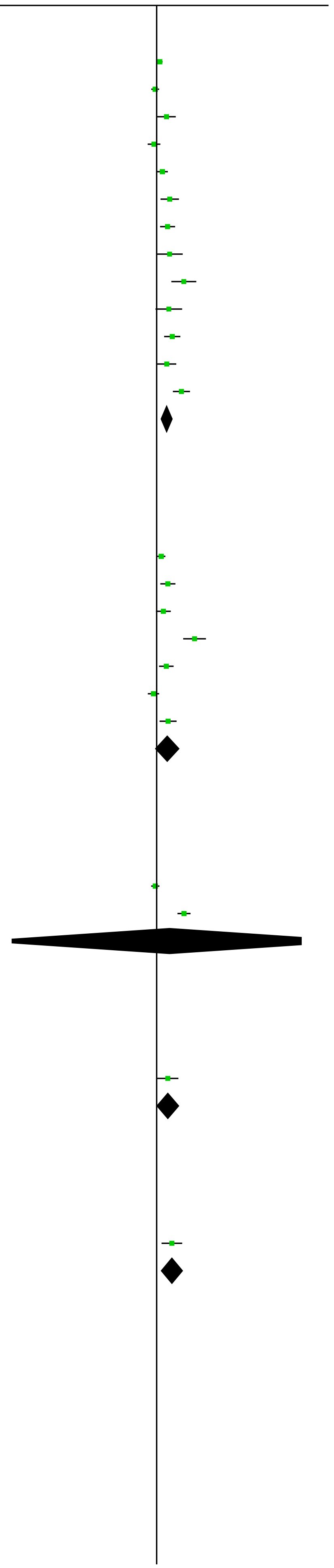

Test for overall effect: Not applicable
Heterogeneity: Not applicable

1.37.8 Others

**Subtotal**	**0**	**0**		**Not estimable**

Test for overall effect: Not applicable
Heterogeneity: Not applicable

**Total (HKSJ [a])**	**1017**	**1109**	**100.0%**	**0.74 [0.44 , 1.03]**
95% prediction interval				[-0.60 , 2.07]

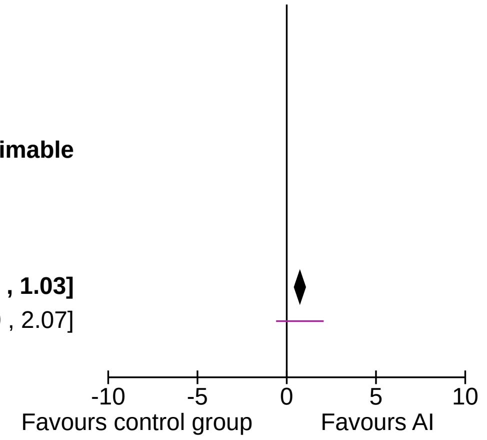

Test for overall effect: T = 5.21, df = 23 (P < 0.0001)
Test for subgroup differences: Chi² = 0.84, df = 4 (P = 0.93), I² = 0%
Heterogeneity: Tau² (REML [b]) = 0.40; Chi² = 169.19, df = 23 (P < 0.00001); I² = 89%

Footnotes
[a] CI calculated by Hartung-Knapp-Sidik-Jonkman (HKSJ) method.
[b] Tau² calculated by Restricted Maximum-Likelihood method.

Analysis 1.38: Kirkpatrick level 2: theoretical knowledge (subgroup: region of study)

Study or Subgroup	AI Mean [SMD]	AI SD [SMD]	AI Total	Control group Mean [SMD]	Control group SD [SMD]	Control group Total	Weight	Std. Mean Difference IV, Random, 95% CI [SMD]
**1.38.1 Asia**								
Aneesh 2025	12.83	3.47	103	13.18	3.36	103	4.8%	-0.10 [-0.38 , 0.17]
Chen 2025	84.47	3.48	20	81.72	4.37	20	4.0%	0.68 [0.04 , 1.32]
Gan 2024	141.2	26.68	54	130.8	25.56	56	4.6%	0.40 [0.02 , 0.77]
Han 2025	7.32	1.22	31	6.72	1.33	29	4.3%	0.46 [-0.05 , 0.98]
Hsu 2024	82.69	8.46	24	61.48	7.4	24	3.6%	2.62 [1.84 , 3.41]
Huang 2025	41.24	8.19	94	42.03	8.34	93	4.8%	-0.10 [-0.38 , 0.19]
Hui 2025	93.9	3.65	21	90.33	4.08	21	4.0%	0.90 [0.27 , 1.54]
Jiang 2024	15.87	3.4	31	13.2	3.57	30	4.3%	0.76 [0.24 , 1.28]
Li 2023	32.33	5.86	15	20.31	6.55	16	3.4%	1.88 [1.02 , 2.74]
Liaw 2023	9.06	1.78	32	7.75	2.08	32	4.3%	0.67 [0.16 , 1.17]
Shi 2025	74.3	14.8	50	77.6	14.9	50	4.6%	-0.22 [-0.61 , 0.17]
Wang 2025	95.83	5.22	24	91.22	6.21	24	4.1%	0.79 [0.20 , 1.38]
Wu 2020	13.39	2.4	19	11.38	3.17	19	3.9%	0.70 [0.04 , 1.36]
Wu 2024	86.44	5.59	31	77.86	4.16	30	4.1%	1.71 [1.12 , 2.31]
**Subtotal (HKSJ [a])**			**549**			**547**	**58.8%**	**0.75 [0.29 , 1.20]**
Test for overall effect: T = 3.57, df = 13 (P = 0.003)								
Heterogeneity: Tau² (REML [b]) = 0.51; Chi² = 103.34, df = 13 (P < 0.00001); I² = 90%								
**1.38.2 North America**								
Kalam 2025	6.2	1.93	10	4.36	2.01	11	3.3%	0.90 [-0.01 , 1.80]
Ramsamooj 2025	75.2	3.2	21	71.1	4	41	4.2%	1.08 [0.52 , 1.64]
**Subtotal (HKSJ [a])**			**31**			**52**	**7.5%**	**1.03 [-0.01 , 2.07]**
Test for overall effect: T = 12.53, df = 1 (P = 0.05)								
Heterogeneity: Tau² (REML [b]) = 0.00; Chi² = 0.11, df = 1 (P = 0.74); I² = 0%								
**1.38.3 South America**								
**Subtotal**			**0**			**0**		**Not estimable**
Test for overall effect: Not applicable								
Heterogeneity: Not applicable								
**1.38.4 Central America**								
**Subtotal**			**0**			**0**		**Not estimable**
Test for overall effect: Not applicable								
Heterogeneity: Not applicable								
**1.38.5 Africa**								
**Subtotal**			**0**			**0**		**Not estimable**
Test for overall effect: Not applicable								
Heterogeneity: Not applicable								
**1.38.6 Europe**								
Akutay 2024	7.43	1.58	94	6.9	1.74	94	4.8%	0.32 [0.03 , 0.61]
Al-Kahf 2023	12.5	1.8	170	12.1	1.9	252	4.9%	0.21 [0.02 , 0.41]
Digiacomo 2025	10	11.11	41	12	10.37	39	4.5%	-0.18 [-0.62 , 0.26]
Doner 2025	67.87	12.68	33	57.85	12.85	28	4.3%	0.78 [0.25 , 1.30]
Ng 2025	71.43	15.06	10	54.29	23.13	10	3.2%	0.84 [-0.08 , 1.76]
Sahin 2025	81.8	11.5	19	69.4	11.5	16	3.8%	1.05 [0.34 , 1.77]
Svendsen 2024	7.7	2	15	5.9	2.5	16	3.7%	0.77 [0.04 , 1.51]
Yilmaz 2025	11.4	1.1	55	9	1.4	55	4.5%	1.89 [1.44 , 2.35]
**Subtotal (HKSJ [a])**			**437**			**510**	**33.7%**	**0.68 [0.14 , 1.22]**
Test for overall effect: T = 2.99, df = 7 (P = 0.02)								
Heterogeneity: Tau² (REML [b]) = 0.37; Chi² = 59.14, df = 7 (P < 0.00001); I² = 89%								
**1.38.7 Oceania**								
**Subtotal**			**0**			**0**		**Not estimable**

Std. Mean Difference
IV, Random, 95% CI [SMD]

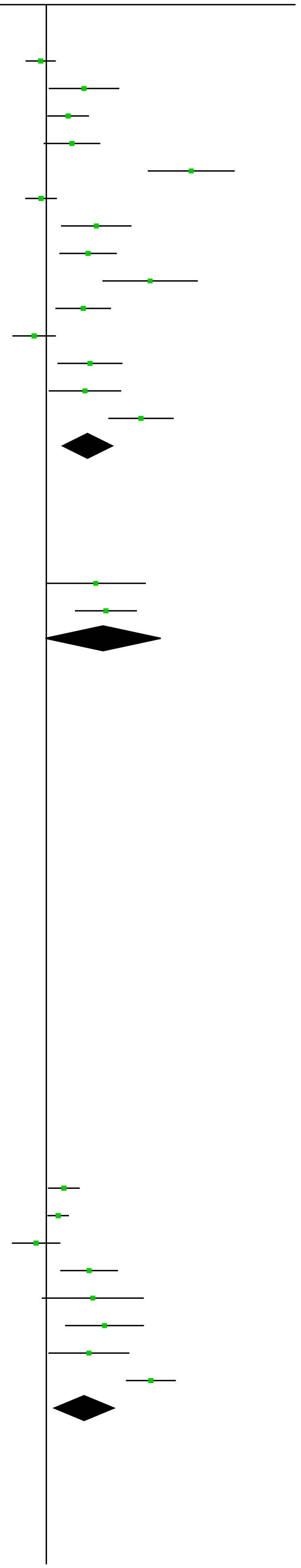

Test for overall effect: Not applicable
Heterogeneity: Not applicable

1.38.8 Multi-continents

**Subtotal**	**0**	**0**		**Not estimable**

Test for overall effect: Not applicable
Heterogeneity: Not applicable

**Total (HKSJ [a])**	**1017**	**1109**	**100.0%**	**0.74 [0.44 , 1.03]**
95% prediction interval				[-0.60 , 2.07]

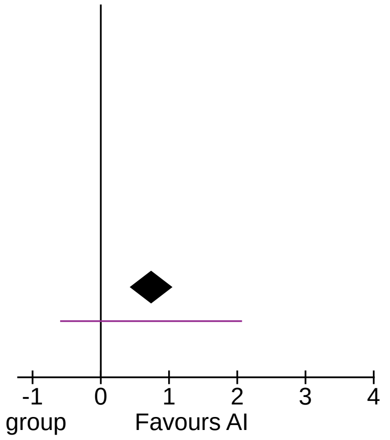

Test for overall effect: T = 5.21, df = 23 (P < 0.0001)
Test for subgroup differences: Chi² = 3.21, df = 2 (P = 0.20), I² = 37.6%
Heterogeneity: Tau² (REML [b]) = 0.40; Chi² = 169.19, df = 23 (P < 0.00001); I² = 89%

Footnotes
[a] CI calculated by Hartung-Knapp-Sidik-Jonkman (HKSJ) method.
[b] Tau² calculated by Restricted Maximum-Likelihood method.

Analysis 1.39: Kirkpatrick level 2: theoretical knowledge (subgroup: LLM vs non-LLM)

Study or Subgroup	AI Mean [SMD]	AI SD [SMD]	AI Total	Control group Mean [SMD]	Control group SD [SMD]	Control group Total	Weight	Std. Mean Difference IV, Random, 95% CI [SMD]
**1.39.1 LLM**								
Akutay 2024	7.43	1.58	94	6.9	1.74	94	4.8%	0.32 [0.03 , 0.61]
Aneesh 2025	12.83	3.47	103	13.18	3.36	103	4.8%	-0.10 [-0.38 , 0.17]
Digiacomo 2025	10	11.11	41	12	10.37	39	4.5%	-0.18 [-0.62 , 0.26]
Doner 2025	67.87	12.68	33	57.85	12.85	28	4.3%	0.78 [0.25 , 1.30]
Gan 2024	141.2	26.68	54	130.8	25.56	56	4.6%	0.40 [0.02 , 0.77]
Huang 2025	41.24	8.19	94	42.03	8.34	93	4.8%	-0.10 [-0.38 , 0.19]
Hui 2025	93.9	3.65	21	90.33	4.08	21	4.0%	0.90 [0.27 , 1.54]
Kalam 2025	6.2	1.93	10	4.36	2.01	11	3.3%	0.90 [-0.01 , 1.80]
Ng 2025	71.43	15.06	10	54.29	23.13	10	3.2%	0.84 [-0.08 , 1.76]
Ramsamooj 2025	75.2	3.2	21	71.1	4	41	4.2%	1.08 [0.52 , 1.64]
Sahin 2025	81.8	11.5	19	69.4	11.5	16	3.8%	1.05 [0.34 , 1.77]
Shi 2025	74.3	14.8	50	77.6	14.9	50	4.6%	-0.22 [-0.61 , 0.17]
Svendsen 2024	7.7	2	15	5.9	2.5	16	3.7%	0.77 [0.04 , 1.51]
Wang 2025	95.83	5.22	24	91.22	6.21	24	4.1%	0.79 [0.20 , 1.38]
Wu 2024	86.44	5.59	31	77.86	4.16	30	4.1%	1.71 [1.12 , 2.31]
Yilmaz 2025	11.4	1.1	55	9	1.4	55	4.5%	1.89 [1.44 , 2.35]
**Subtotal (HKSJ [a])**			**675**			**687**	**67.3%**	**0.64 [0.29 , 0.99]**
Test for overall effect: T = 3.95, df = 15 (P = 0.001)								
Heterogeneity: Tau² (REML [b]) = 0.37; Chi² = 119.75, df = 15 (P < 0.00001); I² = 87%								
**1.39.2 Non-LLM**								
Al-Kahf 2023	12.5	1.8	170	12.1	1.9	252	4.9%	0.21 [0.02 , 0.41]
Chen 2025	84.47	3.48	20	81.72	4.37	20	4.0%	0.68 [0.04 , 1.32]
Han 2025	7.32	1.22	31	6.72	1.33	29	4.3%	0.46 [-0.05 , 0.98]
Hsu 2024	82.69	8.46	24	61.48	7.4	24	3.6%	2.62 [1.84 , 3.41]
Jiang 2024	15.87	3.4	31	13.2	3.57	30	4.3%	0.76 [0.24 , 1.28]
Li 2023	32.33	5.86	15	20.31	6.55	16	3.4%	1.88 [1.02 , 2.74]
Liaw 2023	9.06	1.78	32	7.75	2.08	32	4.3%	0.67 [0.16 , 1.17]
Wu 2020	13.39	2.4	19	11.38	3.17	19	3.9%	0.70 [0.04 , 1.36]
**Subtotal (HKSJ [a])**			**342**			**422**	**32.7%**	**0.94 [0.28 , 1.60]**
Test for overall effect: T = 3.36, df = 7 (P = 0.01)								
Heterogeneity: Tau² (REML [b]) = 0.50; Chi² = 48.01, df = 7 (P < 0.00001); I² = 89%								
**Total (HKSJ [a])**			**1017**			**1109**	**100.0%**	**0.74 [0.44 , 1.03]**
95% prediction interval								[-0.60 , 2.07]

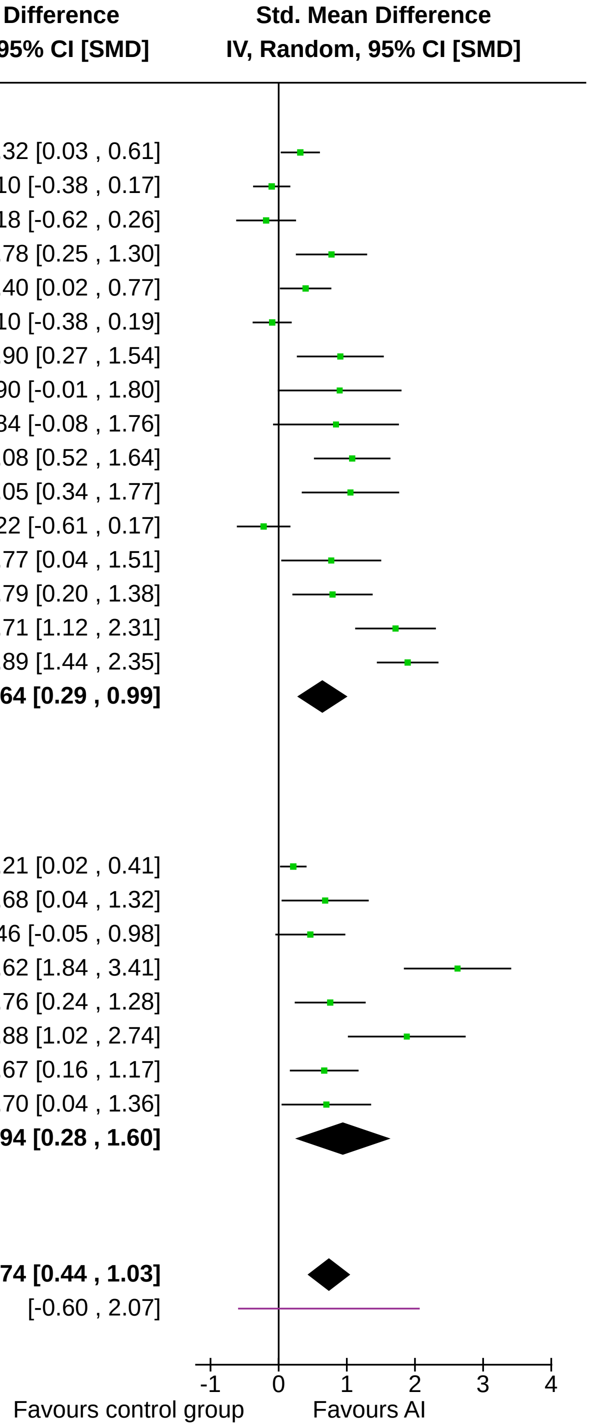

Test for overall effect: T = 5.21, df = 23 (P < 0.0001)
Test for subgroup differences: Chi² = 0.86, df = 1 (P = 0.35), I² = 0%
Heterogeneity: Tau² (REML [b]) = 0.40; Chi² = 169.19, df = 23 (P < 0.00001); I² = 89%

Footnotes

[a] CI calculated by Hartung-Knapp-Sidik-Jonkman (HKSJ) method.
[b] Tau² calculated by Restricted Maximum-Likelihood method.

Analysis 1.40: Kirkpatrick level 2: theoretical knowledge (subgroup: predominant function of application - teaching learning vs assessment)

	AI			Control group				Std. Mean Difference
Study or Subgroup	Mean [SMD]	SD [SMD]	Total	Mean [SMD]	SD [SMD]	Total	Weight	IV, Random, 95% CI [SMD]
**1.40.1 Teaching and learning**								
Akutay 2024	7.43	1.58	94	6.9	1.74	94	4.8%	0.32 [0.03 , 0.61]
Al-Kahf 2023	12.5	1.8	170	12.1	1.9	252	4.9%	0.21 [0.02 , 0.41]
Aneesh 2025	12.83	3.47	103	13.18	3.36	103	4.8%	-0.10 [-0.38 , 0.17]
Chen 2025	84.47	3.48	20	81.72	4.37	20	4.0%	0.68 [0.04 , 1.32]
Digiacomo 2025	10	11.11	41	12	10.37	39	4.5%	-0.18 [-0.62 , 0.26]
Doner 2025	67.87	12.68	33	57.85	12.85	28	4.3%	0.78 [0.25 , 1.30]
Gan 2024	141.2	26.68	54	130.8	25.56	56	4.6%	0.40 [0.02 , 0.77]
Han 2025	7.32	1.22	31	6.72	1.33	29	4.3%	0.46 [-0.05 , 0.98]
Hsu 2024	82.69	8.46	24	61.48	7.4	24	3.6%	2.62 [1.84 , 3.41]
Huang 2025	41.24	8.19	94	42.03	8.34	93	4.8%	-0.10 [-0.38 , 0.19]
Hui 2025	93.9	3.65	21	90.33	4.08	21	4.0%	0.90 [0.27 , 1.54]
Jiang 2024	15.87	3.4	31	13.2	3.57	30	4.3%	0.76 [0.24 , 1.28]
Ng 2025	71.43	15.06	10	54.29	23.13	10	3.2%	0.84 [-0.08 , 1.76]
Ramsamooj 2025	75.2	3.2	21	71.1	4	41	4.2%	1.08 [0.52 , 1.64]
Sahin 2025	81.8	11.5	19	69.4	11.5	16	3.8%	1.05 [0.34 , 1.77]
Shi 2025	74.3	14.8	50	77.6	14.9	50	4.6%	-0.22 [-0.61 , 0.17]
Svendsen 2024	7.7	2	15	5.9	2.5	16	3.7%	0.77 [0.04 , 1.51]
Wang 2025	95.83	5.22	24	91.22	6.21	24	4.1%	0.79 [0.20 , 1.38]
Wu 2020	13.39	2.4	19	11.38	3.17	19	3.9%	0.70 [0.04 , 1.36]
Wu 2024	86.44	5.59	31	77.86	4.16	30	4.1%	1.71 [1.12 , 2.31]
Yilmaz 2025	11.4	1.1	55	9	1.4	55	4.5%	1.89 [1.44 , 2.35]
**Subtotal (HKSJ [a])**			**960**			**1050**	**89.0%**	**0.69 [0.37 , 1.01]**
Test for overall effect: T = 4.54, df = 20 (P = 0.0002)								
Heterogeneity: Tau² (REML [b]) = 0.41; Chi² = 156.15, df = 20 (P < 0.00001); I² = 90%								
**1.40.2 Assessment**								
**Subtotal**			**0**			**0**		**Not estimable**
Test for overall effect: Not applicable								
Heterogeneity: Not applicable								
**1.40.3 Teaching-learning and assessment**								
Kalam 2025	6.2	1.93	10	4.36	2.01	11	3.3%	0.90 [-0.01 , 1.80]
Li 2023	32.33	5.86	15	20.31	6.55	16	3.4%	1.88 [1.02 , 2.74]
Liaw 2023	9.06	1.78	32	7.75	2.08	32	4.3%	0.67 [0.16 , 1.17]
**Subtotal (HKSJ [a])**			**57**			**59**	**11.0%**	**1.10 [-0.49 , 2.68]**
Test for overall effect: T = 2.98, df = 2 (P = 0.10)								
Heterogeneity: Tau² (REML [b]) = 0.26; Chi² = 5.66, df = 2 (P = 0.06); I² = 64%								
**Total (HKSJ [a])**			**1017**			**1109**	**100.0%**	**0.74 [0.44 , 1.03]**
95% prediction interval								[-0.60 , 2.07]

Std. Mean Difference
IV, Random, 95% CI [SMD]

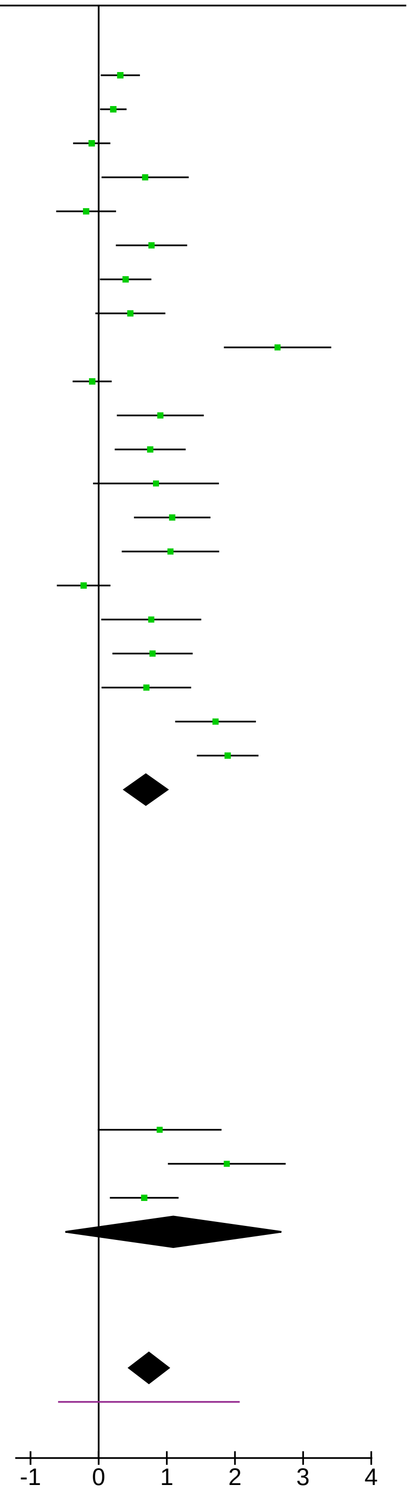

Test for overall effect: T = 5.21, df = 23 (P < 0.0001)
Test for subgroup differences: Chi² = 1.02, df = 1 (P = 0.31), I² = 2.4%
Heterogeneity: Tau² (REML [b]) = 0.40; Chi² = 169.19, df = 23 (P < 0.00001); I² = 89%

Footnotes
[a] CI calculated by Hartung-Knapp-Sidik-Jonkman (HKSJ) method.
[b] Tau² calculated by Restricted Maximum-Likelihood method.

Analysis 1.41: Kirkpatrick level 2: theoretical knowledge (subgroup: single vs multiple sessions)

Study or Subgroup	AI Mean [SMD]	AI SD [SMD]	AI Total	Control group Mean [SMD]	Control group SD [SMD]	Control group Total	Weight	Std. Mean Difference IV, Random, 95% CI [SMD]
**1.41.1 Single session**								
Digiacomo 2025	10	11.11	41	12	10.37	39	4.7%	-0.18 [-0.62 , 0.26]
Jiang 2024	15.87	3.4	31	13.2	3.57	30	4.5%	0.76 [0.24 , 1.28]
Ng 2025	71.43	15.06	10	54.29	23.13	10	3.4%	0.84 [-0.08 , 1.76]
Ramsamooj 2025	75.2	3.2	21	71.1	4	41	4.4%	1.08 [0.52 , 1.64]
Svendsen 2024	7.7	2	15	5.9	2.5	16	3.9%	0.77 [0.04 , 1.51]
Wu 2020	13.39	2.4	19	11.38	3.17	19	4.1%	0.70 [0.04 , 1.36]
**Subtotal (HKSJ [a])**			**137**			**155**	**25.0%**	**0.62 [0.13 , 1.12]**
Test for overall effect: T = 3.23, df = 5 (P = 0.02)								
Heterogeneity: Tau² (REML [b]) = 0.16; Chi² = 15.66, df = 5 (P = 0.008); I² = 64%								
**1.41.2 Multiple sessions**								
Akutay 2024	7.43	1.58	94	6.9	1.74	94	5.0%	0.32 [0.03 , 0.61]
Al-Kahf 2023	12.5	1.8	170	12.1	1.9	252	5.1%	0.21 [0.02 , 0.41]
Aneesh 2025	12.83	3.47	103	13.18	3.36	103	5.0%	-0.10 [-0.38 , 0.17]
Chen 2025	84.47	3.48	20	81.72	4.37	20	4.2%	0.68 [0.04 , 1.32]
Doner 2025	67.87	12.68	33	57.85	12.85	28	4.5%	0.78 [0.25 , 1.30]
Gan 2024	141.2	26.68	54	130.8	25.56	56	4.8%	0.40 [0.02 , 0.77]
Hsu 2024	82.69	8.46	24	61.48	7.4	24	3.8%	2.62 [1.84 , 3.41]
Huang 2025	41.24	8.19	94	42.03	8.34	93	5.0%	-0.10 [-0.38 , 0.19]
Hui 2025	93.9	3.65	21	90.33	4.08	21	4.2%	0.90 [0.27 , 1.54]
Kalam 2025	6.2	1.93	10	4.36	2.01	11	3.5%	0.90 [-0.01 , 1.80]
Li 2023	32.33	5.86	15	20.31	6.55	16	3.6%	1.88 [1.02 , 2.74]
Liaw 2023	9.06	1.78	32	7.75	2.08	32	4.5%	0.67 [0.16 , 1.17]
Sahin 2025	81.8	11.5	19	69.4	11.5	16	4.0%	1.05 [0.34 , 1.77]
Shi 2025	74.3	14.8	50	77.6	14.9	50	4.8%	-0.22 [-0.61 , 0.17]
Wang 2025	95.83	5.22	24	91.22	6.21	24	4.3%	0.79 [0.20 , 1.38]
Wu 2024	86.44	5.59	31	77.86	4.16	30	4.3%	1.71 [1.12 , 2.31]
Yilmaz 2025	11.4	1.1	55	9	1.4	55	4.6%	1.89 [1.44 , 2.35]
**Subtotal (HKSJ [a])**			**849**			**925**	**75.0%**	**0.80 [0.40 , 1.20]**
Test for overall effect: T = 4.20, df = 16 (P = 0.0007)								
Heterogeneity: Tau² (REML [b]) = 0.53; Chi² = 152.66, df = 16 (P < 0.00001); I² = 92%								
**Total (HKSJ [a])**			**986**			**1080**	**100.0%**	**0.75 [0.45 , 1.06]**
95% prediction interval								[-0.62 , 2.12]

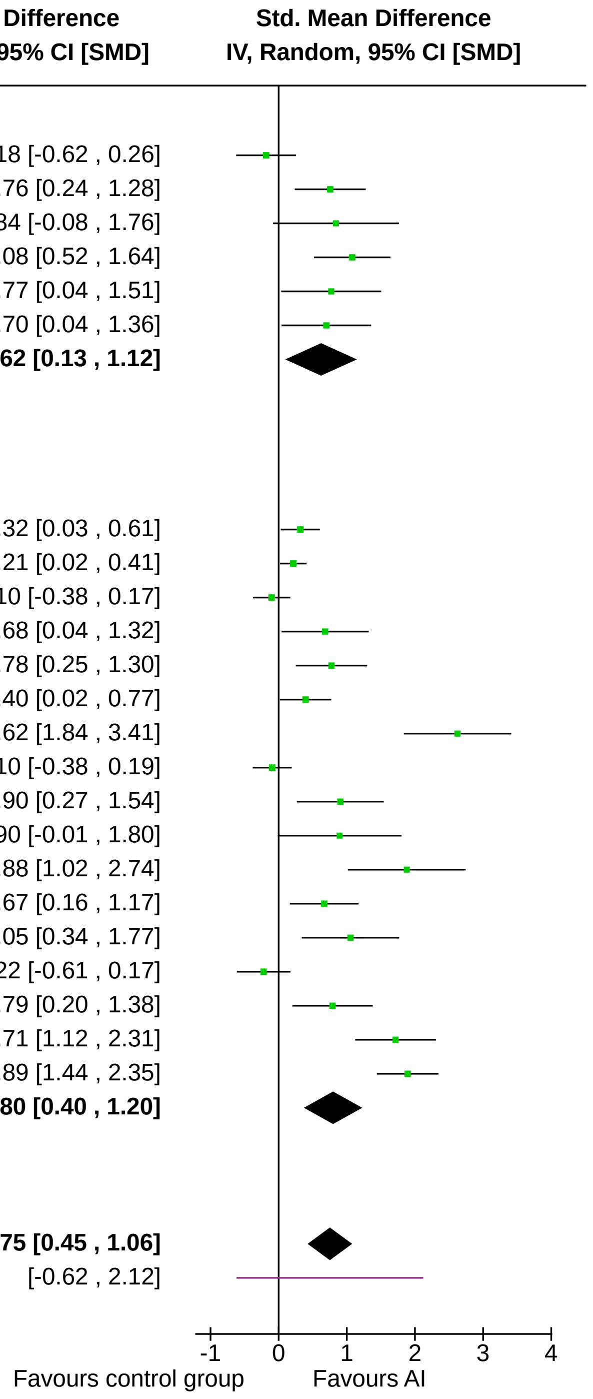

Test for overall effect: T = 5.10, df = 22 (P < 0.0001)
Test for subgroup differences: Chi² = 0.42, df = 1 (P = 0.52), I² = 0%
Heterogeneity: Tau² (REML [b]) = 0.41; Chi² = 169.18, df = 22 (P < 0.00001); I² = 89%

Footnotes

[a] CI calculated by Hartung-Knapp-Sidik-Jonkman (HKSJ) method.
[b] Tau² calculated by Restricted Maximum-Likelihood method.

Analysis 1.42: Kirkpatrick level 2: clinical skills (LLM content generator vs control)

Study or Subgroup	LLM content generator Mean [SMD]	SD [SMD]	Total	Control group Mean [SMD]	SD [SMD]	Total	Weight	Std. Mean Difference IV, Random, 95% CI [SMD]
Çiçek 2024	74.7	15.1	56	78.5	20.6	59	49.8%	-0.21 [-0.57 , 0.16]
Saatçi 2024	75.36	12.24	91	57.57	16.19	89	50.2%	1.24 [0.92 , 1.56]
**Total (HKSJ [a])**			**147**			**148**	**100.0%**	**0.52 [-8.66 , 9.69]**
95% prediction interval								[-15.22 , 16.25]

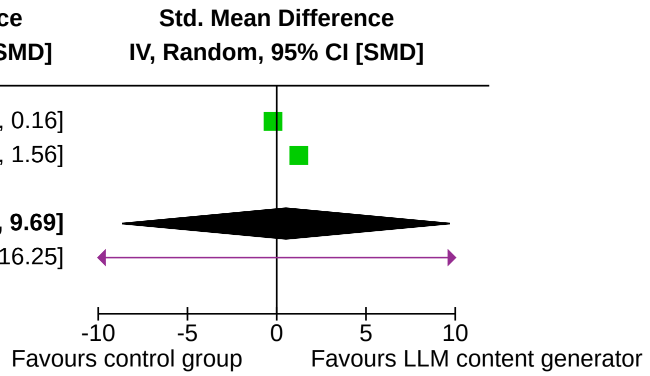

Test for overall effect: T = 0.72, df = 1 (P = 0.60)

Heterogeneity: Tau² (REML [b]) = 1.01; Chi² = 33.89, df = 1 (P < 0.00001); I² = 97%

Footnotes

[a] CI calculated by Hartung-Knapp-Sidik-Jonkman (HKSJ) method.

[b] Tau² calculated by Restricted Maximum-Likelihood method.

Analysis 1.43: Kirkpatrick level 2: clinical skills (LLM personalised learning aid vs control)

Study or Subgroup	LLM personalised learning aid Mean [SMD]	SD [SMD]	Total	Control group Mean [SMD]	SD [SMD]	Total	Weight	Std. Mean Difference IV, Random, 95% CI [SMD]
Benfatah 2024	4.8	0.5	20	4.2	0.6	20	10.4%	1.06 [0.40 , 1.73]
Benfatah 2026	5.4	0.6	30	4.7	0.8	30	11.5%	0.98 [0.44 , 1.51]
Hui 2025	6.14	1.15	21	5.14	1.06	21	10.7%	0.89 [0.25 , 1.52]
Montagna 2025	3.75	0.55	5	3.8	0.51	11	7.5%	-0.09 [-1.15 , 0.97]
Nissen 2025	51.8	22	77	55.8	22.8	77	13.1%	-0.18 [-0.49 , 0.14]
Sahin 2025	38	13.1	19	31.3	18.5	16	10.4%	0.41 [-0.26 , 1.09]
Shi 2025	18.2	1.76	50	19.3	2.1	50	12.5%	-0.56 [-0.96 , -0.16]
Wang 2024	33.5	7.78	50	27	4.81	51	12.4%	1.00 [0.58 , 1.41]
Wu 2024	83.84	6.13	31	79.12	4.27	30	11.6%	0.88 [0.35 , 1.41]
**Total (HKSJ [a])**			**303**			**306**	**100.0%**	**0.49 [0.00 , 0.97]**
95% prediction interval								[-0.93 , 1.90]

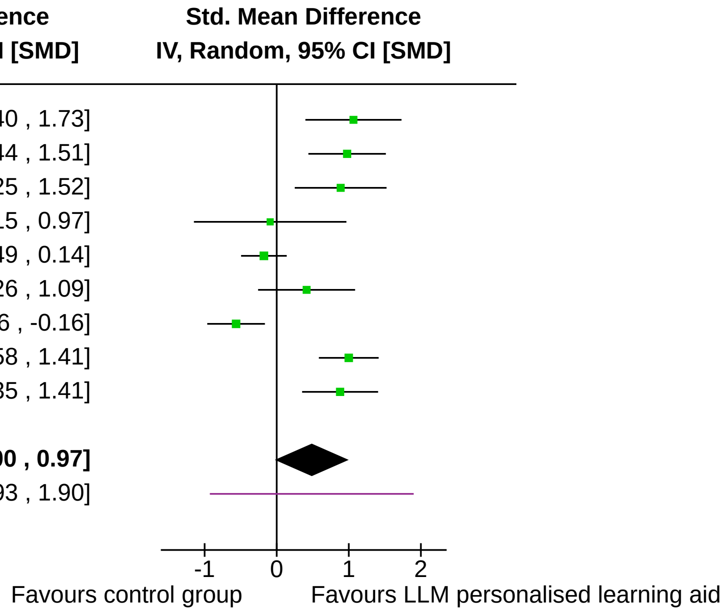

Test for overall effect: T = 2.32, df = 8 (P = 0.05)

Heterogeneity: Tau² (REML [b]) = 0.33; Chi² = 57.07, df = 8 (P < 0.00001); I² = 83%

Footnotes

[a] CI calculated by Hartung-Knapp-Sidik-Jonkman (HKSJ) method.

[b] Tau² calculated by Restricted Maximum-Likelihood method.

Analysis 1.44: Kirkpatrick level 2: clinical skills (LLM virtual patient vs control)

Study or Subgroup	LLM virtual patient Mean [SMD]	SD [SMD]	Total	Control group Mean [SMD]	SD [SMD]	Total	Weight	Std. Mean Difference IV, Random, 95% CI [SMD]
Wang 2025a	86.79	5.46	28	73.64	4.76	28	100.0%	2.53 [1.82 , 3.25]
**Total**			**28**			**28**	**100.0%**	**2.53 [1.82 , 3.25]**
95% prediction interval								[1.82 , 3.25]

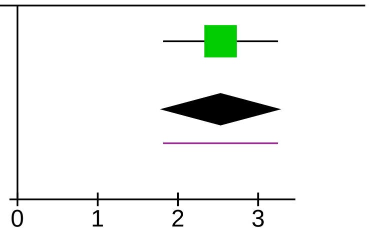

Test for overall effect: Z = 6.94 (P < 0.00001)

Heterogeneity: Not applicable

Analysis 1.45: Kirkpatrick level 2: clinical skills (LLM content generator + LLM virtual patient + LLM personalised learning aid vs control)

Study or Subgroup	LLM content generator + LLM virtual patient + LLM personalised learning aid Mean [SMD]	SD [SMD]	Total	Control group Mean [SMD]	SD [SMD]	Total	Weight	Std. Mean Difference IV, Random, 95% CI [SMD]
Ali 2025	12.98	5.15	44	13.54	5	44	100.0%	-0.11 [-0.53 , 0.31]
**Total**			**44**			**44**	**100.0%**	**-0.11 [-0.53 , 0.31]**
95% prediction interval								[-0.53 , 0.31]

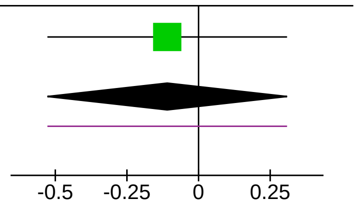

Test for overall effect: Z = 0.51 (P = 0.61)

Heterogeneity: Not applicable

Analysis 1.46: Kirkpatrick level 2: clinical skills (LLM virtual patient + LLM personalised learning aid vs control)

Study or Subgroup	LLM virtual patient + LLM personalised learning aid Mean [SMD]	SD [SMD]	Total	Control group Mean [SMD]	SD [SMD]	Total	Weight	Std. Mean Difference IV, Random, 95% CI [SMD]
Brügge 2024	3.6	0.13	10	3.02	0.12	11	30.4%	4.46 [2.74 , 6.18]
Lee 2025	57.7	12.1	9	55.4	12.9	10	34.2%	0.18 [-0.73 , 1.08]
Luo 2025	64.62	9.52	42	54.12	8.8	42	35.4%	1.13 [0.67 , 1.60]
**Total (HKSJ [a])**			**61**			**63**	**100.0%**	**1.82 [-3.63 , 7.27]**
95% prediction interval								[-8.61 , 12.24]

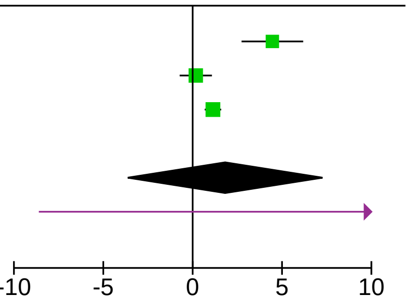

Test for overall effect: T = 1.43, df = 2 (P = 0.29)

Heterogeneity: Tau^2 (REML [b]) = 4.26; Chi^2 = 18.63, df = 2 (P < 0.0001); I^2 = 95%

Footnotes

[a] CI calculated by Hartung-Knapp-Sidik-Jonkman (HKSJ) method.

[b] Tau^2 calculated by Restricted Maximum-Likelihood method.

Analysis 1.47: Kirkpatrick level 2: clinical skills (non-LLM AI imaging diagnostic aid vs control)

Study or Subgroup	AI imaging diagnostic aid (non LLM) Mean [SMD]	SD [SMD]	Total	Control group Mean [SMD]	SD [SMD]	Total	Weight	Std. Mean Difference IV, Random, 95% CI [SMD]
Ayan 2024	0.89	0.02	20	0.86	0.04	20	24.4%	0.93 [0.27 , 1.59]
Cheng 2020	88.87	5.51	15	78.66	14.53	15	21.6%	0.90 [0.15 , 1.66]
Dao 2022	76.67	13.22	16	78.33	13.22	16	23.3%	-0.12 [-0.82 , 0.57]
Schropp 2023	57.7	21.6	37	55.3	18.9	37	30.7%	0.12 [-0.34 , 0.57]
**Total (HKSJ [a])**			**88**			**88**	**100.0%**	**0.43 [-0.41 , 1.27]**
95% prediction interval								[-1.13 , 1.99]

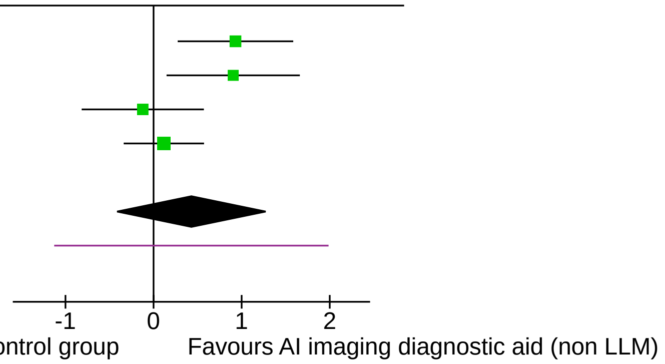

Test for overall effect: T = 1.62, df = 3 (P = 0.20)

Heterogeneity: Tau² (REML [b]) = 0.17; Chi² = 7.83, df = 3 (P = 0.05); I² = 62%

Footnotes

[a] CI calculated by Hartung-Knapp-Sidik-Jonkman (HKSJ) method.

[b] Tau² calculated by Restricted Maximum-Likelihood method.

Analysis 1.48: Kirkpatrick level 2: clinical skills (non-LLM AI moderated adaptive learning platform vs control)

Study or Subgroup	AI-moderated adaptive learning platform (non-LLM) Mean [SMD]	SD [SMD]	Total	Control group Mean [SMD]	SD [SMD]	Total	Weight	Std. Mean Difference IV, Random, 95% CI [SMD]
Simsek-Cetinkaya 2023	59.71	12.01	52	73.72	15.53	51	50.7%	-1.00 [-1.41 , -0.59]
Vannaprathip 2025	21.11	2.32	18	15.39	2.67	18	49.3%	2.24 [1.38 , 3.09]
**Total (HKSJ [a])**			**70**			**69**	**100.0%**	**0.59 [-19.98 , 21.17]**
95% prediction interval								[-34.78 , 35.97]

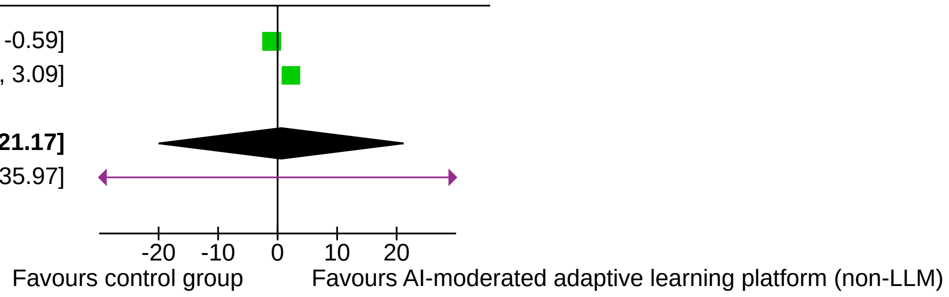

Test for overall effect: T = 0.37, df = 1 (P = 0.78)

Heterogeneity: Tau² (REML [b]) = 5.13; Chi² = 45.03, df = 1 (P < 0.00001); I² = 98%

Footnotes

[a] CI calculated by Hartung-Knapp-Sidik-Jonkman (HKSJ) method.

[b] Tau² calculated by Restricted Maximum-Likelihood method.

Analysis 1.49: Kirkpatrick level 2: clinical skills (NLP rule-based virtual patient vs control)

Study or Subgroup	Rule-based virtual patient (non-LLM) Mean [SMD]	SD [SMD]	Total	Control group Mean [SMD]	SD [SMD]	Total	Weight	Std. Mean Difference IV, Random, 95% CI [SMD]
Kestel 2025	84.84	6.79	38	74.05	3.24	41	100.0%	2.03 [1.49 , 2.58]
**Total**			**38**			**41**	**100.0%**	**2.03 [1.49 , 2.58]**
95% prediction interval								[1.49 , 2.58]

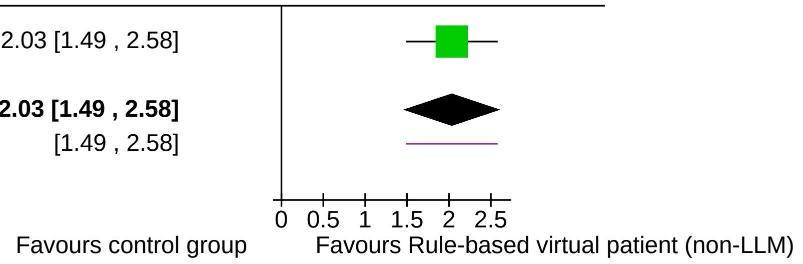

Test for overall effect: Z = 7.27 (P < 0.00001)

Heterogeneity: Not applicable

Analysis 1.50: Kirkpatrick level 2: clinical skills (NLP rule-based chatbot+ virtual patient vs control)

Study or Subgroup	NLP rule-based chatbot + NLP rule-based virtual patient Mean [SMD]	SD [SMD]	Total	Control group Mean [SMD]	SD [SMD]	Total	Weight	Std. Mean Difference IV, Random, 95% CI [SMD]
Jiang 2024	86.2	17.3	31	81.7	19.6	30	100.0%	0.24 [-0.26 , 0.74]
**Total**			**31**			**30**	**100.0%**	**0.24 [-0.26 , 0.74]**
95% prediction interval								[-0.26 , 0.74]

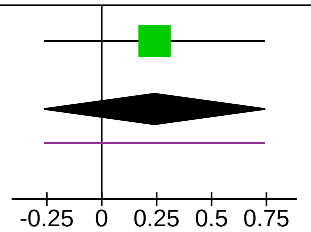

Test for overall effect: Z = 0.94 (P = 0.35)

Heterogeneity: Not applicable

Analysis 1.51: Kirkpatrick level 2: clinical skills (AI-VR virtual doctor vs control)

Study or Subgroup	AI-VR virtual doctor (non-LLM) Mean [SMD]	SD [SMD]	Total	Control group Mean [SMD]	SD [SMD]	Total	Weight	Std. Mean Difference IV, Random, 95% CI [SMD]
Liaw 2023	13.63	4.23	32	12.75	3.85	32	100.0%	0.21 [-0.28 , 0.71]
**Total**			**32**			**32**	**100.0%**	**0.21 [-0.28 , 0.71]**
95% prediction interval								[-0.28 , 0.71]

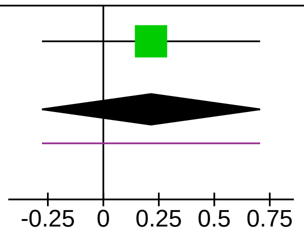

Test for overall effect: Z = 0.86 (P = 0.39)

Heterogeneity: Not applicable

Analysis 1.52: Kirkpatrick level 2: clinical skills (AI procedure assistant + AI-moderated adaptive learning platform vs control)

Study or Subgroup	AI procedure assistant + AI-moderated adaptive learning platform Mean [SMD]	SD [SMD]	Total	Control group Mean [SMD]	SD [SMD]	Total	Weight	Std. Mean Difference IV, Random, 95% CI [SMD]
Usta 2025	76.1	4.58	10	70.6	8.07	10	100.0%	0.80 [-0.12 , 1.72]
**Total**			**10**			**10**	**100.0%**	**0.80 [-0.12 , 1.72]**
95% prediction interval								[-0.12 , 1.72]

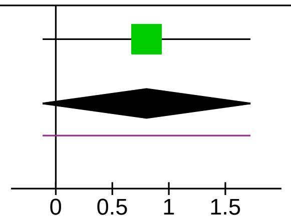

Test for overall effect: Z = 1.71 (P = 0.09)

Heterogeneity: Not applicable

Analysis 1.53: Kirkpatrick level 2: clinical skills (subgroup: field of study)

Study or Subgroup	AI Mean [SMD]	AI SD [SMD]	AI Total	Control group Mean [SMD]	Control group SD [SMD]	Control group Total	Weight	Std. Mean Difference IV, Random, 95% CI [SMD]
**1.53.1 Medicine**								
Brügge 2024	3.6	0.13	10	3.02	0.12	11	2.2%	4.46 [2.74 , 6.18]
Cheng 2020	88.87	5.51	15	78.66	14.53	15	3.7%	0.90 [0.15 , 1.66]
Çiçek 2024	74.7	15.1	56	78.5	20.6	59	4.2%	-0.21 [-0.57 , 0.16]
Dao 2022	76.67	13.22	16	78.33	13.22	16	3.8%	-0.12 [-0.82 , 0.57]
Hui 2025	6.14	1.15	21	5.14	1.06	21	3.9%	0.89 [0.25 , 1.52]
Jiang 2024	86.2	17.3	31	81.7	19.6	30	4.0%	0.24 [-0.26 , 0.74]
Lee 2025	57.7	12.1	9	55.4	12.9	10	3.5%	0.18 [-0.73 , 1.08]
Luo 2025	64.62	9.52	42	54.12	8.8	42	4.1%	1.13 [0.67 , 1.60]
Montagna 2025	3.75	0.55	5	3.8	0.51	11	3.2%	-0.09 [-1.15 , 0.97]
Nissen 2025	51.8	22	77	55.8	22.8	77	4.2%	-0.18 [-0.49 , 0.14]
Wang 2024	33.5	7.78	50	27	4.81	51	4.1%	1.00 [0.58 , 1.41]
Wang 2025a	86.79	5.46	28	73.64	4.76	28	3.8%	2.53 [1.82 , 3.25]
Wu 2024	83.84	6.13	31	79.12	4.27	30	4.0%	0.88 [0.35 , 1.41]
**Subtotal (HKSJ [a])**			**391**			**401**	**48.7%**	**0.79 [0.09 , 1.49]**
Test for overall effect: T = 2.44, df = 12 (P = 0.03)								
Heterogeneity: Tau² (REML [b]) = 0.96; Chi² = 106.27, df = 12 (P < 0.00001); I² = 93%								
**1.53.2 Nursing**								
Benfatah 2024	4.8	0.5	20	4.2	0.6	20	3.8%	1.06 [0.40 , 1.73]
Benfatah 2026	5.4	0.6	30	4.7	0.8	30	4.0%	0.98 [0.44 , 1.51]
Kestel 2025	84.84	6.79	38	74.05	3.24	41	4.0%	2.03 [1.49 , 2.58]
Liaw 2023	13.63	4.23	32	12.75	3.85	32	4.1%	0.21 [-0.28 , 0.71]
Saatçi 2024	75.36	12.24	91	57.57	16.19	89	4.2%	1.24 [0.92 , 1.56]
Shi 2025	18.2	1.76	50	19.3	2.1	50	4.2%	-0.56 [-0.96 , -0.16]
Simsek-Cetinkaya 2023	59.71	12.01	52	73.72	15.53	51	4.1%	-1.00 [-1.41 , -0.59]
**Subtotal (HKSJ [a])**			**313**			**313**	**28.4%**	**0.56 [-0.44 , 1.55]**
Test for overall effect: T = 1.37, df = 6 (P = 0.22)								
Heterogeneity: Tau² (REML [b]) = 1.10; Chi² = 135.87, df = 6 (P < 0.00001); I² = 95%								
**1.53.3 Dentistry**								
Ayan 2024	0.89	0.02	20	0.86	0.04	20	3.8%	0.93 [0.27 , 1.59]
Schropp 2023	57.7	21.6	37	55.3	18.9	37	4.1%	0.12 [-0.34 , 0.57]
Usta 2025	76.1	4.58	10	70.6	8.07	10	3.4%	0.80 [-0.12 , 1.72]
Vannaprathip 2025	21.11	2.32	18	15.39	2.67	18	3.5%	2.24 [1.38 , 3.09]
**Subtotal (HKSJ [a])**			**85**			**85**	**14.9%**	**0.98 [-0.43 , 2.39]**
Test for overall effect: T = 2.22, df = 3 (P = 0.11)								
Heterogeneity: Tau² (REML [b]) = 0.65; Chi² = 19.27, df = 3 (P = 0.0002); I² = 84%								
**1.53.4 Pharmacy**								
Ali 2025	12.98	5.15	44	13.54	5	44	4.1%	-0.11 [-0.53 , 0.31]
**Subtotal**			**44**			**44**	**4.1%**	**-0.11 [-0.53 , 0.31]**
Test for overall effect: Z = 0.51 (P = 0.61)								
Heterogeneity: Not applicable								
**1.53.5 Physiotherapy**								
Sahin 2025	38	13.1	19	31.3	18.5	16	3.8%	0.41 [-0.26 , 1.09]
**Subtotal**			**19**			**16**	**3.8%**	**0.41 [-0.26 , 1.09]**
Test for overall effect: Z = 1.21 (P = 0.23)								
Heterogeneity: Not applicable								
**1.53.6 Health Sciences**								
**Subtotal**			**0**			**0**		**Not estimable**
Test for overall effect: Not applicable								
Heterogeneity: Not applicable								

Std. Mean Difference
IV, Random, 95% CI [SMD]

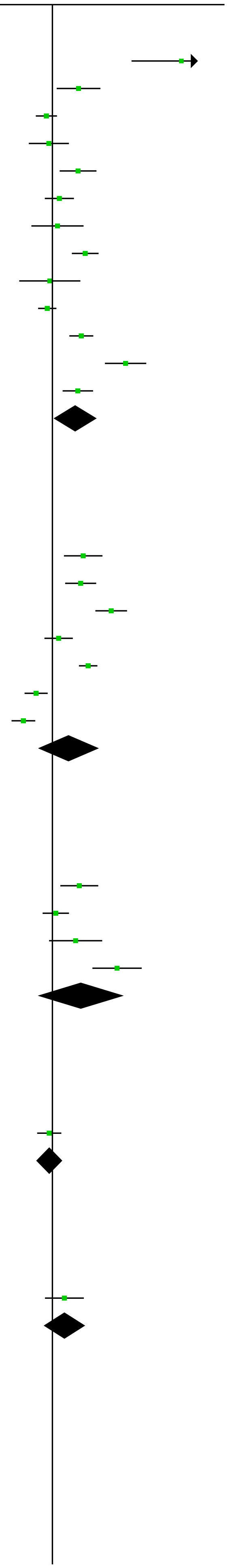

**1.53.7 Optometry**				
**Subtotal**	**0**	**0**		**Not estimable**
Test for overall effect: Not applicable				
Heterogeneity: Not applicable				
**1.53.8 Others**				
**Subtotal**	**0**	**0**		**Not estimable**
Test for overall effect: Not applicable				
Heterogeneity: Not applicable				
**Total (HKSJ [a])**	**852**	**859**	**100.0%**	**0.69 [0.28 , 1.11]**
95% prediction interval				[-1.21 , 2.60]

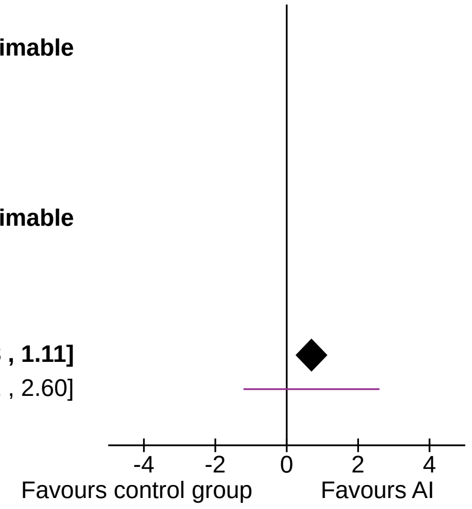

Test for overall effect: T = 3.46, df = 25 (P = 0.002)
Test for subgroup differences: Chi² = 8.78, df = 4 (P = 0.07), I² = 54.5%
Heterogeneity: Tau² (REML [b]) = 0.81; Chi² = 270.69, df = 25 (P < 0.00001); I² = 92%

Footnotes
[a] CI calculated by Hartung-Knapp-Sidik-Jonkman (HKSJ) method.
[b] Tau² calculated by Restricted Maximum-Likelihood method.

Analysis 1.54: Kirkpatrick level 2: clinical skills (subgroup: region of study)

Study or Subgroup	AI Mean [SMD]	AI SD [SMD]	AI Total	Control group Mean [SMD]	Control group SD [SMD]	Control group Total	Weight	Std. Mean Difference IV, Random, 95% CI [SMD]
**1.54.1 Asia**								
Ali 2025	12.98	5.15	44	13.54	5	44	4.1%	-0.11 [-0.53 , 0.31]
Cheng 2020	88.87	5.51	15	78.66	14.53	15	3.7%	0.90 [0.15 , 1.66]
Hui 2025	6.14	1.15	21	5.14	1.06	21	3.9%	0.89 [0.25 , 1.52]
Jiang 2024	86.2	17.3	31	81.7	19.6	30	4.0%	0.24 [-0.26 , 0.74]
Lee 2025	57.7	12.1	9	55.4	12.9	10	3.5%	0.18 [-0.73 , 1.08]
Liaw 2023	13.63	4.23	32	12.75	3.85	32	4.1%	0.21 [-0.28 , 0.71]
Luo 2025	64.62	9.52	42	54.12	8.8	42	4.1%	1.13 [0.67 , 1.60]
Shi 2025	18.2	1.76	50	19.3	2.1	50	4.2%	-0.56 [-0.96 , -0.16]
Vannaprathip 2025	21.11	2.32	18	15.39	2.67	18	3.5%	2.24 [1.38 , 3.09]
Wang 2024	33.5	7.78	50	27	4.81	51	4.1%	1.00 [0.58 , 1.41]
Wang 2025a	86.79	5.46	28	73.64	4.76	28	3.8%	2.53 [1.82 , 3.25]
Wu 2024	83.84	6.13	31	79.12	4.27	30	4.0%	0.88 [0.35 , 1.41]
**Subtotal (HKSJ [a])**			**371**			**371**	**47.0%**	**0.77 [0.20 , 1.33]**
Test for overall effect: T = 2.97, df = 11 (P = 0.01)								
Heterogeneity: Tau^2 (REML [b]) = 0.69; Chi^2 = 101.84, df = 11 (P < 0.00001); I^2 = 90%								
**1.54.2 North America**								
Dao 2022	76.67	13.22	16	78.33	13.22	16	3.8%	-0.12 [-0.82 , 0.57]
**Subtotal**			**16**			**16**	**3.8%**	**-0.12 [-0.82 , 0.57]**
Test for overall effect: Z = 0.35 (P = 0.73)								
Heterogeneity: Not applicable								
**1.54.3 South America**								
**Subtotal**			**0**			**0**		**Not estimable**
Test for overall effect: Not applicable								
Heterogeneity: Not applicable								
**1.54.4 Central America**								
**Subtotal**			**0**			**0**		**Not estimable**
Test for overall effect: Not applicable								
Heterogeneity: Not applicable								
**1.54.5 Africa**								
Benfatah 2024	4.8	0.5	20	4.2	0.6	20	3.8%	1.06 [0.40 , 1.73]
Benfatah 2026	5.4	0.6	30	4.7	0.8	30	4.0%	0.98 [0.44 , 1.51]
**Subtotal (HKSJ [a])**			**50**			**50**	**7.8%**	**1.01 [0.47 , 1.56]**
Test for overall effect: T = 23.60, df = 1 (P = 0.03)								
Heterogeneity: Tau^2 (REML [b]) = 0.00; Chi^2 = 0.04, df = 1 (P = 0.84); I^2 = 0%								
**1.54.6 Europe**								
Ayan 2024	0.89	0.02	20	0.86	0.04	20	3.8%	0.93 [0.27 , 1.59]
Brügge 2024	3.6	0.13	10	3.02	0.12	11	2.2%	4.46 [2.74 , 6.18]
Çiçek 2024	74.7	15.1	56	78.5	20.6	59	4.2%	-0.21 [-0.57 , 0.16]
Kestel 2025	84.84	6.79	38	74.05	3.24	41	4.0%	2.03 [1.49 , 2.58]
Montagna 2025	3.75	0.55	5	3.8	0.51	11	3.2%	-0.09 [-1.15 , 0.97]
Nissen 2025	51.8	22	77	55.8	22.8	77	4.2%	-0.18 [-0.49 , 0.14]
Saatçi 2024	75.36	12.24	91	57.57	16.19	89	4.2%	1.24 [0.92 , 1.56]
Sahin 2025	38	13.1	19	31.3	18.5	16	3.8%	0.41 [-0.26 , 1.09]
Schropp 2023	57.7	21.6	37	55.3	18.9	37	4.1%	0.12 [-0.34 , 0.57]
Simsek-Cetinkaya 2023	59.71	12.01	52	73.72	15.53	51	4.1%	-1.00 [-1.41 , -0.59]
Usta 2025	76.1	4.58	10	70.6	8.07	10	3.4%	0.80 [-0.12 , 1.72]
**Subtotal (HKSJ [a])**			**415**			**422**	**41.4%**	**0.66 [-0.24 , 1.57]**
Test for overall effect: T = 1.64, df = 10 (P = 0.13)								
Heterogeneity: Tau^2 (REML [b]) = 1.41; Chi^2 = 154.38, df = 10 (P < 0.00001); I^2 = 96%								

Std. Mean Difference IV, Random, 95% CI [SMD]

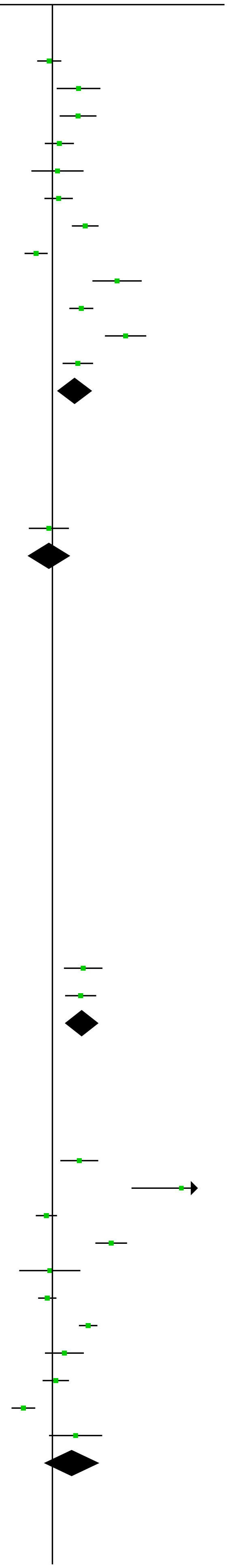

	AI Total	Control Total	Weight	Std. Mean Difference
**1.54.7 Oceania**				
**Subtotal**	**0**	**0**		**Not estimable**
Test for overall effect: Not applicable				
Heterogeneity: Not applicable				
**1.54.8 Multi-continents**				
**Subtotal**	**0**	**0**		**Not estimable**
Test for overall effect: Not applicable				
Heterogeneity: Not applicable				
**Total (HKSJ [a])**	**852**	**859**	**100.0%**	**0.69 [0.28 , 1.11]**
95% prediction interval				[-1.21 , 2.60]

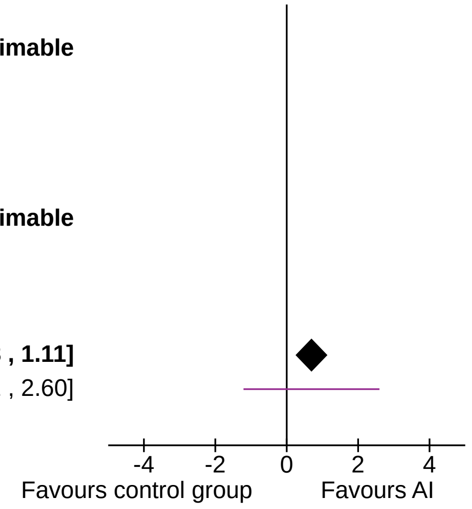

Test for overall effect: T = 3.46, df = 25 (P = 0.002)
Test for subgroup differences: Chi^2 = 11.52, df = 3 (P = 0.009), I^2 = 74.0%
Heterogeneity: Tau^2 (REML [b]) = 0.81; Chi^2 = 270.69, df = 25 (P < 0.00001); I^2 = 92%

Footnotes

[a] CI calculated by Hartung-Knapp-Sidik-Jonkman (HKSJ) method.

[b] Tau^2 calculated by Restricted Maximum-Likelihood method.

Analysis 1.55: Kirkpatrick level 2: clinical skills (subgroup: LLM vs non-LLM)

Study or Subgroup	AI Mean [SMD]	AI SD [SMD]	AI Total	Control group Mean [SMD]	Control group SD [SMD]	Control group Total	Weight	Std. Mean Difference IV, Random, 95% CI [SMD]
**1.55.1 LLM**								
Ali 2025	12.98	5.15	44	13.54	5	44	4.1%	-0.11 [-0.53 , 0.31]
Benfatah 2024	4.8	0.5	20	4.2	0.6	20	3.8%	1.06 [0.40 , 1.73]
Benfatah 2026	5.4	0.6	30	4.7	0.8	30	4.0%	0.98 [0.44 , 1.51]
Brügge 2024	3.6	0.13	10	3.02	0.12	11	2.2%	4.46 [2.74 , 6.18]
Çiçek 2024	74.7	15.1	56	78.5	20.6	59	4.2%	-0.21 [-0.57 , 0.16]
Hui 2025	6.14	1.15	21	5.14	1.06	21	3.9%	0.89 [0.25 , 1.52]
Lee 2025	57.7	12.1	9	55.4	12.9	10	3.5%	0.18 [-0.73 , 1.08]
Luo 2025	64.62	9.52	42	54.12	8.8	42	4.1%	1.13 [0.67 , 1.60]
Montagna 2025	3.75	0.55	5	3.8	0.51	11	3.2%	-0.09 [-1.15 , 0.97]
Nissen 2025	51.8	22	77	55.8	22.8	77	4.2%	-0.18 [-0.49 , 0.14]
Saatçi 2024	75.36	12.24	91	57.57	16.19	89	4.2%	1.24 [0.92 , 1.56]
Sahin 2025	38	13.1	19	31.3	18.5	16	3.8%	0.41 [-0.26 , 1.09]
Shi 2025	18.2	1.76	50	19.3	2.1	50	4.2%	-0.56 [-0.96 , -0.16]
Wang 2024	33.5	7.78	50	27	4.81	51	4.1%	1.00 [0.58 , 1.41]
Wang 2025a	86.79	5.46	28	73.64	4.76	28	3.8%	2.53 [1.82 , 3.25]
Wu 2024	83.84	6.13	31	79.12	4.27	30	4.0%	0.88 [0.35 , 1.41]
**Subtotal (HKSJ [a])**			**583**			**589**	**61.4%**	**0.75 [0.17 , 1.33]**
Test for overall effect: T = 2.77, df = 15 (P = 0.01)								
Heterogeneity: Tau² (REML [b]) = 0.86; Chi² = 162.71, df = 15 (P < 0.00001); I² = 93%								
**1.55.2 Non-LLM**								
Ayan 2024	0.89	0.02	20	0.86	0.04	20	3.8%	0.93 [0.27 , 1.59]
Cheng 2020	88.87	5.51	15	78.66	14.53	15	3.7%	0.90 [0.15 , 1.66]
Dao 2022	76.67	13.22	16	78.33	13.22	16	3.8%	-0.12 [-0.82 , 0.57]
Jiang 2024	86.2	17.3	31	81.7	19.6	30	4.0%	0.24 [-0.26 , 0.74]
Kestel 2025	84.84	6.79	38	74.05	3.24	41	4.0%	2.03 [1.49 , 2.58]
Liaw 2023	13.63	4.23	32	12.75	3.85	32	4.1%	0.21 [-0.28 , 0.71]
Schropp 2023	57.7	21.6	37	55.3	18.9	37	4.1%	0.12 [-0.34 , 0.57]
Simsek-Cetinkaya 2023	59.71	12.01	52	73.72	15.53	51	4.1%	-1.00 [-1.41 , -0.59]
Usta 2025	76.1	4.58	10	70.6	8.07	10	3.4%	0.80 [-0.12 , 1.72]
Vannaprathip 2025	21.11	2.32	18	15.39	2.67	18	3.5%	2.24 [1.38 , 3.09]
**Subtotal (HKSJ [a])**			**269**			**270**	**38.6%**	**0.61 [-0.09 , 1.31]**
Test for overall effect: T = 1.96, df = 9 (P = 0.08)								
Heterogeneity: Tau² (REML [b]) = 0.86; Chi² = 105.96, df = 9 (P < 0.00001); I² = 91%								
**Total (HKSJ [a])**			**852**			**859**	**100.0%**	**0.69 [0.28 , 1.11]**
95% prediction interval								[-1.21 , 2.60]

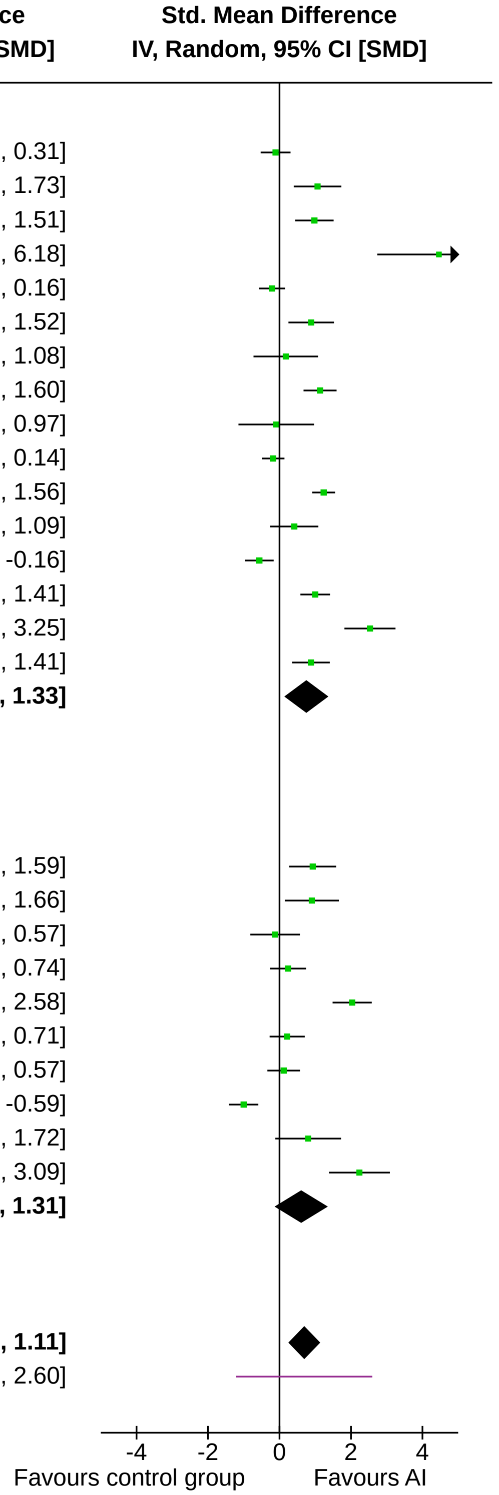

Test for overall effect: T = 3.46, df = 25 (P = 0.002)
Test for subgroup differences: Chi² = 0.12, df = 1 (P = 0.73), I² = 0%
Heterogeneity: Tau² (REML [b]) = 0.81; Chi² = 270.69, df = 25 (P < 0.00001); I² = 92%

Footnotes

[a] CI calculated by Hartung-Knapp-Sidik-Jonkman (HKSJ) method.

[b] Tau² calculated by Restricted Maximum-Likelihood method.

Analysis 1.56: Kirkpatrick level 2: clinical skills (subgroup: main function - teaching learning vs assessment)

Study or Subgroup	AI Mean [SMD]	AI SD [SMD]	AI Total	Control group Mean [SMD]	Control group SD [SMD]	Control group Total	Weight	Std. Mean Difference IV, Random, 95% CI [SMD]
**1.56.1 Teaching and learning**								
Ali 2025	12.98	5.15	44	13.54	5	44	4.1%	-0.11 [-0.53 , 0.31]
Benfatah 2024	4.8	0.5	20	4.2	0.6	20	3.8%	1.06 [0.40 , 1.73]
Benfatah 2026	5.4	0.6	30	4.7	0.8	30	4.0%	0.98 [0.44 , 1.51]
Brügge 2024	3.6	0.13	10	3.02	0.12	11	2.2%	4.46 [2.74 , 6.18]
Cheng 2020	88.87	5.51	15	78.66	14.53	15	3.7%	0.90 [0.15 , 1.66]
Çiçek 2024	74.7	15.1	56	78.5	20.6	59	4.2%	-0.21 [-0.57 , 0.16]
Dao 2022	76.67	13.22	16	78.33	13.22	16	3.8%	-0.12 [-0.82 , 0.57]
Hui 2025	6.14	1.15	21	5.14	1.06	21	3.9%	0.89 [0.25 , 1.52]
Jiang 2024	86.2	17.3	31	81.7	19.6	30	4.0%	0.24 [-0.26 , 0.74]
Kestel 2025	84.84	6.79	38	74.05	3.24	41	4.0%	2.03 [1.49 , 2.58]
Lee 2025	57.7	12.1	9	55.4	12.9	10	3.5%	0.18 [-0.73 , 1.08]
Luo 2025	64.62	9.52	42	54.12	8.8	42	4.1%	1.13 [0.67 , 1.60]
Montagna 2025	3.75	0.55	5	3.8	0.51	11	3.2%	-0.09 [-1.15 , 0.97]
Saatçi 2024	75.36	12.24	91	57.57	16.19	89	4.2%	1.24 [0.92 , 1.56]
Sahin 2025	38	13.1	19	31.3	18.5	16	3.8%	0.41 [-0.26 , 1.09]
Shi 2025	18.2	1.76	50	19.3	2.1	50	4.2%	-0.56 [-0.96 , -0.16]
Simsek-Cetinkaya 2023	59.71	12.01	52	73.72	15.53	51	4.1%	-1.00 [-1.41 , -0.59]
Usta 2025	76.1	4.58	10	70.6	8.07	10	3.4%	0.80 [-0.12 , 1.72]
Vannaprathip 2025	21.11	2.32	18	15.39	2.67	18	3.5%	2.24 [1.38 , 3.09]
Wang 2024	33.5	7.78	50	27	4.81	51	4.1%	1.00 [0.58 , 1.41]
Wang 2025a	86.79	5.46	28	73.64	4.76	28	3.8%	2.53 [1.82 , 3.25]
Wu 2024	83.84	6.13	31	79.12	4.27	30	4.0%	0.88 [0.35 , 1.41]
**Subtotal (HKSJ [a])**			**686**			**693**	**83.8%**	**0.78 [0.30 , 1.27]**
Test for overall effect: T = 3.36, df = 21 (P = 0.003)								
Heterogeneity: Tau² (REML [b]) = 0.95; Chi² = 247.18, df = 21 (P < 0.00001); I² = 93%								
**1.56.2 Assessment**								
**Subtotal**			**0**			**0**		**Not estimable**
Test for overall effect: Not applicable								
Heterogeneity: Not applicable								
**1.56.3 Teaching-learning and assessment**								
Ayan 2024	0.89	0.02	20	0.86	0.04	20	3.8%	0.93 [0.27 , 1.59]
Liaw 2023	13.63	4.23	32	12.75	3.85	32	4.1%	0.21 [-0.28 , 0.71]
Nissen 2025	51.8	22	77	55.8	22.8	77	4.2%	-0.18 [-0.49 , 0.14]
Schropp 2023	57.7	21.6	37	55.3	18.9	37	4.1%	0.12 [-0.34 , 0.57]
**Subtotal (HKSJ [a])**			**166**			**166**	**16.2%**	**0.21 [-0.50 , 0.92]**
Test for overall effect: T = 0.94, df = 3 (P = 0.42)								
Heterogeneity: Tau² (REML [b]) = 0.12; Chi² = 9.31, df = 3 (P = 0.03); I² = 70%								
**Total (HKSJ [a])**			**852**			**859**	**100.0%**	**0.69 [0.28 , 1.11]**
95% prediction interval								[-1.21 , 2.60]

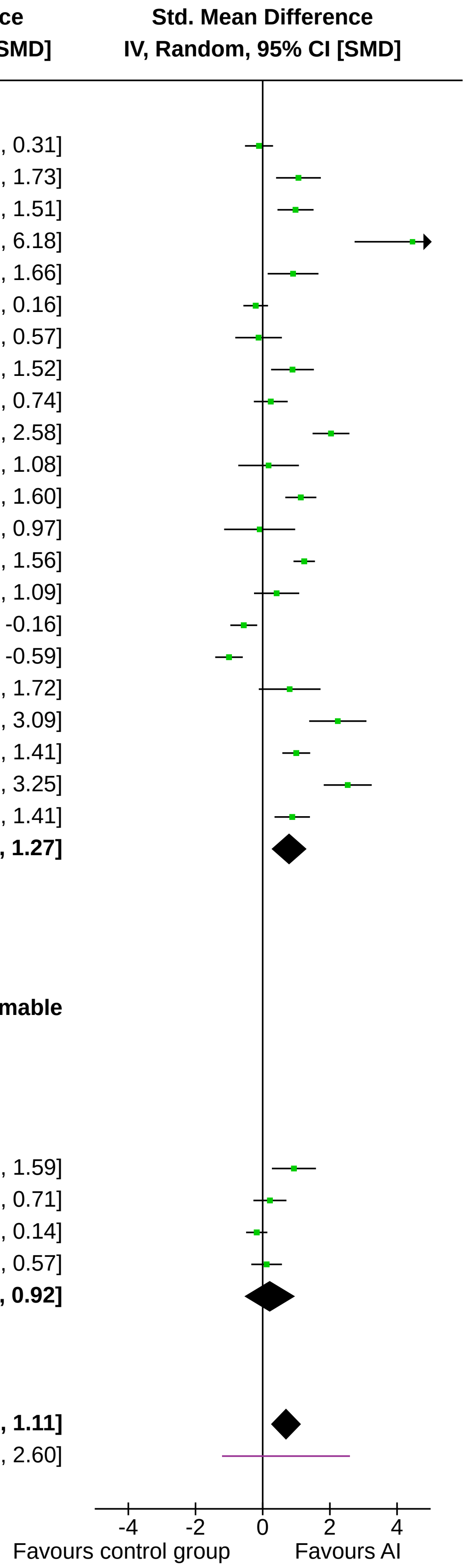

Test for overall effect: T = 3.46, df = 25 (P = 0.002)
Test for subgroup differences: Chi² = 3.19, df = 1 (P = 0.07), I² = 68.7%
Heterogeneity: Tau² (REML [b]) = 0.81; Chi² = 270.69, df = 25 (P < 0.00001); I² = 92%

Footnotes
[a] CI calculated by Hartung-Knapp-Sidik-Jonkman (HKSJ) method.
[b] Tau² calculated by Restricted Maximum-Likelihood method.

Analysis 1.57: Kirkpatrick level 2: clinical skills (subgroup: single versus multiple sessions)

Study or Subgroup	AI Mean [SMD]	AI SD [SMD]	AI Total	Control group Mean [SMD]	Control group SD [SMD]	Control group Total	Weight	Std. Mean Difference IV, Random, 95% CI [SMD]
**1.57.1 Single session**								
Ayan 2024	0.89	0.02	20	0.86	0.04	20	3.8%	0.93 [0.27 , 1.59]
Benfatah 2026	5.4	0.6	30	4.7	0.8	30	4.0%	0.98 [0.44 , 1.51]
Jiang 2024	86.2	17.3	31	81.7	19.6	30	4.0%	0.24 [-0.26 , 0.74]
Lee 2025	57.7	12.1	9	55.4	12.9	10	3.5%	0.18 [-0.73 , 1.08]
Luo 2025	64.62	9.52	42	54.12	8.8	42	4.1%	1.13 [0.67 , 1.60]
Montagna 2025	3.75	0.55	5	3.8	0.51	11	3.2%	-0.09 [-1.15 , 0.97]
Simsek-Cetinkaya 2023	59.71	12.01	52	73.72	15.53	51	4.1%	-1.00 [-1.41 , -0.59]
**Subtotal (HKSJ [a])**			**189**			**194**	**26.8%**	**0.35 [-0.38 , 1.07]**
Test for overall effect: T = 1.17, df = 6 (P = 0.28)								
Heterogeneity: Tau² (REML [b]) = 0.55; Chi² = 61.38, df = 6 (P < 0.00001); I² = 87%								
**1.57.2 Multiple sessions**								
Ali 2025	12.98	5.15	44	13.54	5	44	4.1%	-0.11 [-0.53 , 0.31]
Benfatah 2024	4.8	0.5	20	4.2	0.6	20	3.8%	1.06 [0.40 , 1.73]
Brügge 2024	3.6	0.13	10	3.02	0.12	11	2.2%	4.46 [2.74 , 6.18]
Cheng 2020	88.87	5.51	15	78.66	14.53	15	3.7%	0.90 [0.15 , 1.66]
Çiçek 2024	74.7	15.1	56	78.5	20.6	59	4.2%	-0.21 [-0.57 , 0.16]
Dao 2022	76.67	13.22	16	78.33	13.22	16	3.8%	-0.12 [-0.82 , 0.57]
Hui 2025	6.14	1.15	21	5.14	1.06	21	3.9%	0.89 [0.25 , 1.52]
Kestel 2025	84.84	6.79	38	74.05	3.24	41	4.0%	2.03 [1.49 , 2.58]
Liaw 2023	13.63	4.23	32	12.75	3.85	32	4.1%	0.21 [-0.28 , 0.71]
Nissen 2025	51.8	22	77	55.8	22.8	77	4.2%	-0.18 [-0.49 , 0.14]
Saatçi 2024	75.36	12.24	91	57.57	16.19	89	4.2%	1.24 [0.92 , 1.56]
Sahin 2025	38	13.1	19	31.3	18.5	16	3.8%	0.41 [-0.26 , 1.09]
Schropp 2023	57.7	21.6	37	55.3	18.9	37	4.1%	0.12 [-0.34 , 0.57]
Shi 2025	18.2	1.76	50	19.3	2.1	50	4.2%	-0.56 [-0.96 , -0.16]
Usta 2025	76.1	4.58	10	70.6	8.07	10	3.4%	0.80 [-0.12 , 1.72]
Vannaprathip 2025	21.11	2.32	18	15.39	2.67	18	3.5%	2.24 [1.38 , 3.09]
Wang 2024	33.5	7.78	50	27	4.81	51	4.1%	1.00 [0.58 , 1.41]
Wang 2025a	86.79	5.46	28	73.64	4.76	28	3.8%	2.53 [1.82 , 3.25]
Wu 2024	83.84	6.13	31	79.12	4.27	30	4.0%	0.88 [0.35 , 1.41]
**Subtotal (HKSJ [a])**			**663**			**665**	**73.2%**	**0.83 [0.30 , 1.36]**
Test for overall effect: T = 3.29, df = 18 (P = 0.004)								
Heterogeneity: Tau² (REML [b]) = 0.95; Chi² = 205.31, df = 18 (P < 0.00001); I² = 93%								
**Total (HKSJ [a])**			**852**			**859**	**100.0%**	**0.69 [0.28 , 1.11]**
95% prediction interval								[-1.21 , 2.60]

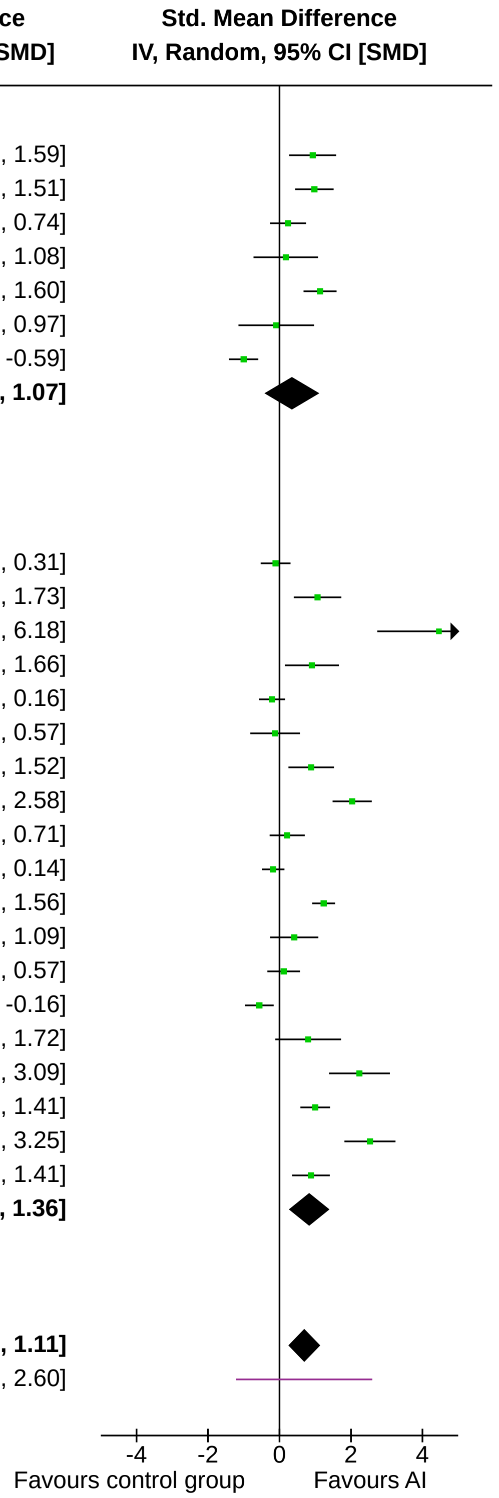

Test for overall effect: T = 3.46, df = 25 (P = 0.002)
Test for subgroup differences: Chi² = 1.55, df = 1 (P = 0.21), I² = 35.4%
Heterogeneity: Tau² (REML [b]) = 0.81; Chi² = 270.69, df = 25 (P < 0.00001); I² = 92%

Footnotes

[a] CI calculated by Hartung-Knapp-Sidik-Jonkman (HKSJ) method.
[b] Tau² calculated by Restricted Maximum-Likelihood method.

Analysis 1.58: Kirkpatrick level 2: practical skills (LLM personalised learning aid vs control)

Study or Subgroup	LLM personalised learning aid Mean [SMD]	SD [SMD]	Total	Control group Mean [SMD]	SD [SMD]	Total	Weight	Std. Mean Difference IV, Random, 95% CI [SMD]
Huang 2025	73.12	10.06	94	65.54	12.48	93	100.0%	0.67 [0.37 , 0.96]
**Total**			**94**			**93**	**100.0%**	**0.67 [0.37 , 0.96]**
95% prediction interval								[0.37 , 0.96]

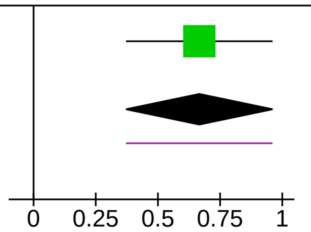

Test for overall effect: Z = 4.43 (P < 0.00001)

Heterogeneity: Not applicable

Analysis 1.59: Kirkpatrick level 2: practical skills (non-LLM AI procedure assistant vs control)

Study or Subgroup	AI procedure assistant (non-LLM) Mean [SMD]	SD [SMD]	Total	Control group Mean [SMD]	SD [SMD]	Total	Weight	Std. Mean Difference IV, Random, 95% CI [SMD]
Aronovitz 2024	26	2.22	21	22	4.07	39	16.8%	1.11 [0.55 , 1.68]
Chang 2024	0.94	0.06	20	1	0.01	20	16.2%	-1.37 [-2.06 , -0.67]
Fazlollahi 2022	4.63	1.39	23	4.4	1.29	24	16.8%	0.17 [-0.40 , 0.74]
Höhne 2025	8.98	1.42	25	9.09	1.27	25	16.8%	-0.08 [-0.63 , 0.47]
Lau 2025	13.24	3.38	21	14.89	3.13	22	16.6%	-0.50 [-1.11 , 0.11]
Yilmaz 2024	4.3	0.11	33	4.11	0.11	32	16.8%	1.71 [1.13 , 2.28]
**Total (HKSJ [a])**			**143**			**162**	**100.0%**	**0.18 [-0.97 , 1.34]**
95% prediction interval								[-2.77 , 3.13]

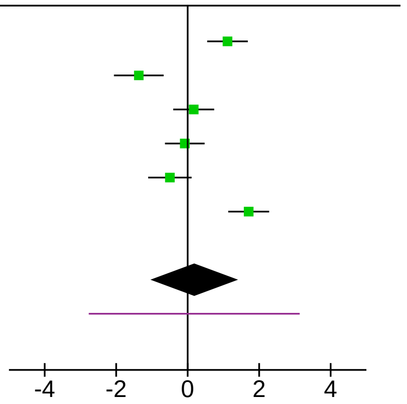

Test for overall effect: T = 0.41, df = 5 (P = 0.70)

Heterogeneity: Tau^2 (REML [b]) = 1.11; Chi^2 = 61.68, df = 5 (P < 0.00001); I^2 = 92%

Footnotes

[a] CI calculated by Hartung-Knapp-Sidik-Jonkman (HKSJ) method.

[b] Tau^2 calculated by Restricted Maximum-Likelihood method.

Analysis 1.60: Kirkpatrick level 2: practical skills (subgroup: field of study)

Study or Subgroup	AI Mean [SMD]	AI SD [SMD]	AI Total	Control group Mean [SMD]	Control group SD [SMD]	Control group Total	Weight	Std. Mean Difference IV, Random, 95% CI [SMD]
**1.60.1 Medicine**								
Aronovitz 2024	26	2.22	21	22	4.07	39	14.3%	1.11 [0.55 , 1.68]
Fazlollahi 2022	4.63	1.39	23	4.4	1.29	24	14.2%	0.17 [-0.40 , 0.74]
Höhne 2025	8.98	1.42	25	9.09	1.27	25	14.3%	-0.08 [-0.63 , 0.47]
Lau 2025	13.24	3.38	21	14.89	3.13	22	14.1%	-0.50 [-1.11 , 0.11]
Yilmaz 2024	4.3	0.11	33	4.11	0.11	32	14.2%	1.71 [1.13 , 2.28]
**Subtotal (HKSJ [a])**			**123**			**142**	**71.1%**	**0.48 [-0.64 , 1.61]**
Test for overall effect: T = 1.20, df = 4 (P = 0.30)								
Heterogeneity: Tau² (REML [b]) = 0.73; Chi² = 37.36, df = 4 (P < 0.00001); I² = 89%								
**1.60.2 Nursing**								
**Subtotal**			**0**			**0**		**Not estimable**
Test for overall effect: Not applicable								
Heterogeneity: Not applicable								
**1.60.3 Dentistry**								
Chang 2024	0.94	0.06	20	1	0.01	20	13.7%	-1.37 [-2.06 , -0.67]
Huang 2025	73.12	10.06	94	65.54	12.48	93	15.2%	0.67 [0.37 , 0.96]
**Subtotal (HKSJ [a])**			**114**			**113**	**28.9%**	**-0.33 [-13.24 , 12.59]**
Test for overall effect: T = 0.32, df = 1 (P = 0.80)								
Heterogeneity: Tau² (REML [b]) = 1.99; Chi² = 27.85, df = 1 (P < 0.00001); I² = 96%								
**1.60.4 Pharmacy**								
**Subtotal**			**0**			**0**		**Not estimable**
Test for overall effect: Not applicable								
Heterogeneity: Not applicable								
**1.60.5 Physiotherapy**								
**Subtotal**			**0**			**0**		**Not estimable**
Test for overall effect: Not applicable								
Heterogeneity: Not applicable								
**1.60.6 Health Sciences**								
**Subtotal**			**0**			**0**		**Not estimable**
Test for overall effect: Not applicable								
Heterogeneity: Not applicable								
**1.60.7 Optometry**								
**Subtotal**			**0**			**0**		**Not estimable**
Test for overall effect: Not applicable								
Heterogeneity: Not applicable								
**1.60.8 Others**								
**Subtotal**			**0**			**0**		**Not estimable**
Test for overall effect: Not applicable								
Heterogeneity: Not applicable								
**Total (HKSJ [a])**			**237**			**255**	**100.0%**	**0.26 [-0.68 , 1.20]**
95% prediction interval								[-2.29 , 2.81]

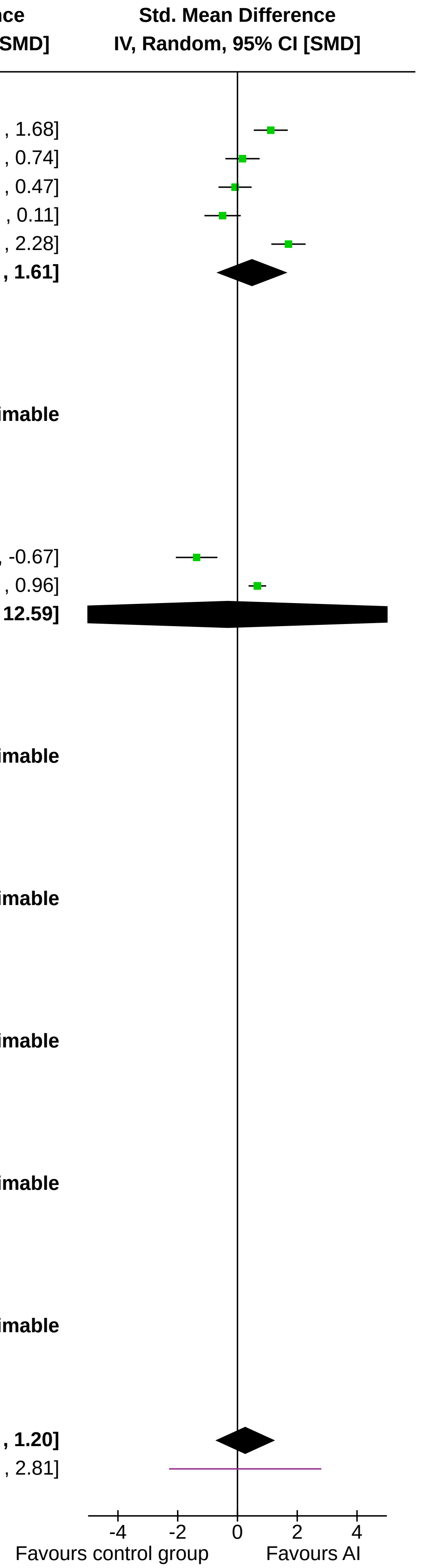

Test for overall effect: T = 0.67, df = 6 (P = 0.53)
Test for subgroup differences: Chi² = 0.55, df = 1 (P = 0.46), I² = 0%
Heterogeneity: Tau² (REML [b]) = 0.94; Chi² = 65.75, df = 6 (P < 0.00001); I² = 93%

Footnotes

[a] CI calculated by Hartung-Knapp-Sidik-Jonkman (HKSJ) method.

[b] Tau² calculated by Restricted Maximum-Likelihood method.

Analysis 1.61: Kirkpatrick level 2: practical skills (subgroup: region of study)

Study or Subgroup	AI Mean [SMD]	AI SD [SMD]	AI Total	Control group Mean [SMD]	Control group SD [SMD]	Control group Total	Weight	Std. Mean Difference IV, Random, 95% CI [SMD]
**1.61.1 Asia**								
Aronovitz 2024	26	2.22	21	22	4.07	39	14.3%	1.11 [0.55 , 1.68]
Huang 2025	73.12	10.06	94	65.54	12.48	93	15.2%	0.67 [0.37 , 0.96]
Lau 2025	13.24	3.38	21	14.89	3.13	22	14.1%	-0.50 [-1.11 , 0.11]
**Subtotal (HKSJ [a])**			**136**			**154**	**43.5%**	**0.44 [-1.59 , 2.47]**
Test for overall effect: T = 0.93, df = 2 (P = 0.45)								
Heterogeneity: Tau² (REML [b]) = 0.58; Chi² = 15.77, df = 2 (P = 0.0004); I² = 91%								
**1.61.2 North America**								
Chang 2024	0.94	0.06	20	1	0.01	20	13.7%	-1.37 [-2.06 , -0.67]
Fazlollahi 2022	4.63	1.39	23	4.4	1.29	24	14.2%	0.17 [-0.40 , 0.74]
Yilmaz 2024	4.3	0.11	33	4.11	0.11	32	14.2%	1.71 [1.13 , 2.28]
**Subtotal (HKSJ [a])**			**76**			**76**	**42.2%**	**0.18 [-3.63 , 3.99]**
Test for overall effect: T = 0.20, df = 2 (P = 0.86)								
Heterogeneity: Tau² (REML [b]) = 2.25; Chi² = 45.35, df = 2 (P < 0.00001); I² = 96%								
**1.61.3 South America**								
**Subtotal**			**0**			**0**		**Not estimable**
Test for overall effect: Not applicable								
Heterogeneity: Not applicable								
**1.61.4 Central America**								
**Subtotal**			**0**			**0**		**Not estimable**
Test for overall effect: Not applicable								
Heterogeneity: Not applicable								
**1.61.5 Africa**								
**Subtotal**			**0**			**0**		**Not estimable**
Test for overall effect: Not applicable								
Heterogeneity: Not applicable								
**1.61.6 Europe**								
Höhne 2025	8.98	1.42	25	9.09	1.27	25	14.3%	-0.08 [-0.63 , 0.47]
**Subtotal**			**25**			**25**	**14.3%**	**-0.08 [-0.63 , 0.47]**
Test for overall effect: Z = 0.28 (P = 0.78)								
Heterogeneity: Not applicable								
**1.61.7 Oceania**								
**Subtotal**			**0**			**0**		**Not estimable**
Test for overall effect: Not applicable								
Heterogeneity: Not applicable								
**1.61.8 Multi-continents**								
**Subtotal**			**0**			**0**		**Not estimable**
Test for overall effect: Not applicable								
Heterogeneity: Not applicable								
**Total (HKSJ [a])**			**237**			**255**	**100.0%**	**0.26 [-0.68 , 1.20]**
95% prediction interval								[-2.29 , 2.81]

Std. Mean Difference
IV, Random, 95% CI [SMD]

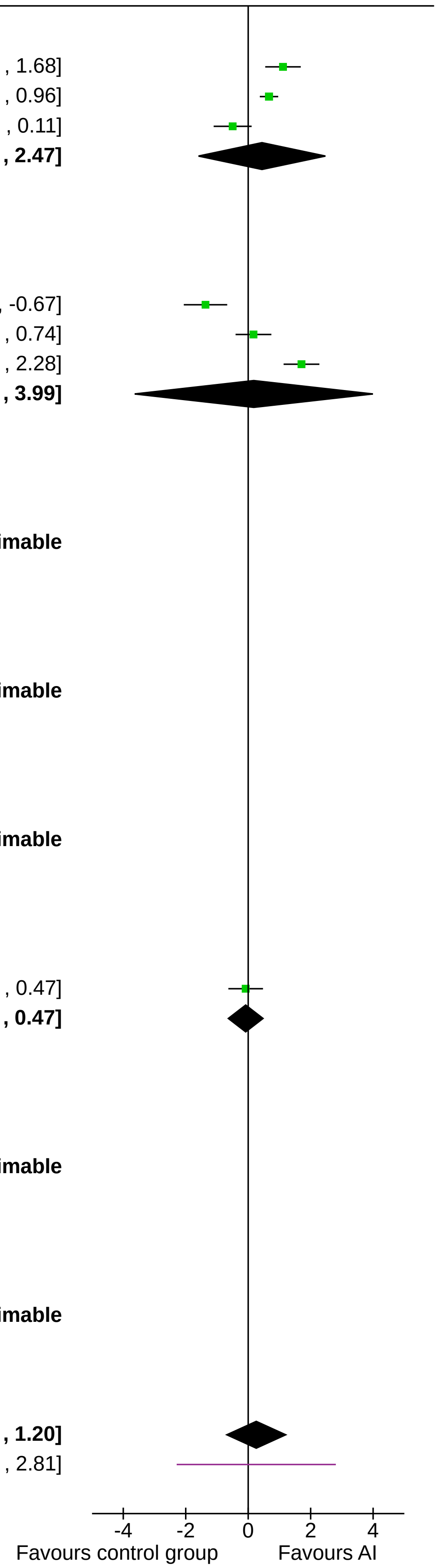

Test for overall effect: T = 0.67, df = 6 (P = 0.53)
Test for subgroup differences: Chi² = 0.91, df = 2 (P = 0.63), I² = 0%
Heterogeneity: Tau² (REML [b]) = 0.94; Chi² = 65.75, df = 6 (P < 0.00001); I² = 93%

Footnotes

[a] CI calculated by Hartung-Knapp-Sidik-Jonkman (HKSJ) method.

[b] Tau^2 calculated by Restricted Maximum-Likelihood method.

Analysis 1.62: Kirkpatrick level 2: practical skills (subgroup: LLM vs non-LLM)

Study or Subgroup	AI Mean [SMD]	AI SD [SMD]	AI Total	Control group Mean [SMD]	Control group SD [SMD]	Control group Total	Weight	Std. Mean Difference IV, Random, 95% CI [SMD]
**1.62.1 LLM**								
Huang 2025	73.12	10.06	94	65.54	12.48	93	15.2%	0.67 [0.37 , 0.96]
**Subtotal**			**94**			**93**	**15.2%**	**0.67 [0.37 , 0.96]**
Test for overall effect: Z = 4.43 (P < 0.00001)								
Heterogeneity: Not applicable								
**1.62.2 Non-LLM**								
Aronovitz 2024	26	2.22	21	22	4.07	39	14.3%	1.11 [0.55 , 1.68]
Chang 2024	0.94	0.06	20	1	0.01	20	13.7%	-1.37 [-2.06 , -0.67]
Fazlollahi 2022	4.63	1.39	23	4.4	1.29	24	14.2%	0.17 [-0.40 , 0.74]
Höhne 2025	8.98	1.42	25	9.09	1.27	25	14.3%	-0.08 [-0.63 , 0.47]
Lau 2025	13.24	3.38	21	14.89	3.13	22	14.1%	-0.50 [-1.11 , 0.11]
Yilmaz 2024	4.3	0.11	33	4.11	0.11	32	14.2%	1.71 [1.13 , 2.28]
**Subtotal (HKSJ [a])**			**143**			**162**	**84.8%**	**0.18 [-0.97 , 1.34]**
Test for overall effect: T = 0.41, df = 5 (P = 0.70)								
Heterogeneity: Tau^2 (REML [b]) = 1.11; Chi^2 = 61.68, df = 5 (P < 0.00001); I^2 = 92%								
**Total (HKSJ [a])**			**237**			**255**	**100.0%**	**0.26 [-0.68 , 1.20]**
95% prediction interval								[-2.29 , 2.81]

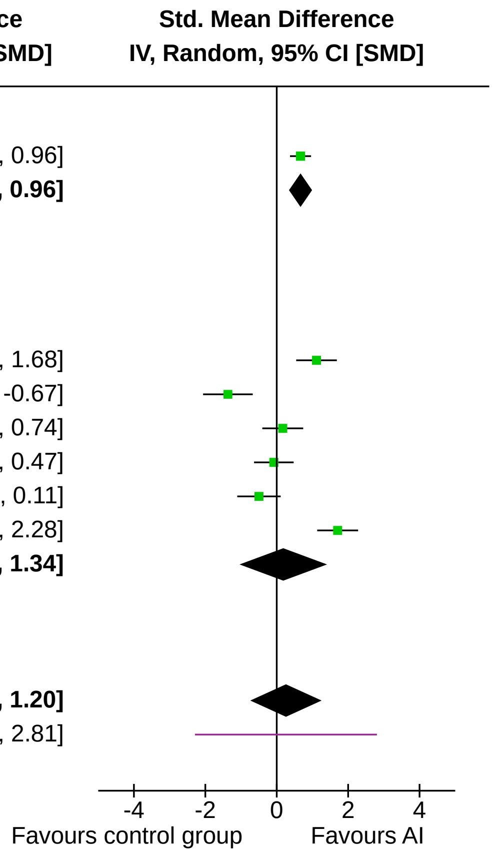

Test for overall effect: T = 0.67, df = 6 (P = 0.53)
Test for subgroup differences: Chi^2 = 1.04, df = 1 (P = 0.31), I^2 = 3.4%
Heterogeneity: Tau^2 (REML [b]) = 0.94; Chi^2 = 65.75, df = 6 (P < 0.00001); I^2 = 93%

Footnotes

[a] CI calculated by Hartung-Knapp-Sidik-Jonkman (HKSJ) method.
[b] Tau^2 calculated by Restricted Maximum-Likelihood method.

Analysis 1.63: Kirkpatrick level 2: practical skills (subgroup: main function of application - teaching learning vs assessment)

Study or Subgroup	AI Mean [SMD]	AI SD [SMD]	AI Total	Control group Mean [SMD]	Control group SD [SMD]	Control group Total	Weight	Std. Mean Difference IV, Random, 95% CI [SMD]
**1.63.1 Teaching and learning**								
Aronovitz 2024	26	2.22	21	22	4.07	39	14.3%	1.11 [0.55 , 1.68]
Chang 2024	0.94	0.06	20	1	0.01	20	13.7%	-1.37 [-2.06 , -0.67]
Höhne 2025	8.98	1.42	25	9.09	1.27	25	14.3%	-0.08 [-0.63 , 0.47]
Huang 2025	73.12	10.06	94	65.54	12.48	93	15.2%	0.67 [0.37 , 0.96]
Lau 2025	13.24	3.38	21	14.89	3.13	22	14.1%	-0.50 [-1.11 , 0.11]
**Subtotal (HKSJ [a])**			**181**			**199**	**71.5%**	**-0.01 [-1.21 , 1.19]**
Test for overall effect: T = 0.02, df = 4 (P = 0.98)								
Heterogeneity: Tau² (REML [b]) = 0.84; Chi² = 44.29, df = 4 (P < 0.00001); I² = 93%								
**1.63.2 Assessment**								
**Subtotal**			**0**			**0**		**Not estimable**
Test for overall effect: Not applicable								
Heterogeneity: Not applicable								
**1.63.3 Teaching-learning and assessment**								
Fazlollahi 2022	4.63	1.39	23	4.4	1.29	24	14.2%	0.17 [-0.40 , 0.74]
Yilmaz 2024	4.3	0.11	33	4.11	0.11	32	14.2%	1.71 [1.13 , 2.28]
**Subtotal (HKSJ [a])**			**56**			**56**	**28.5%**	**0.94 [-8.83 , 10.71]**
Test for overall effect: T = 1.22, df = 1 (P = 0.44)								
Heterogeneity: Tau² (REML [b]) = 1.10; Chi² = 13.84, df = 1 (P = 0.0002); I² = 93%								
**Total (HKSJ [a])**			**237**			**255**	**100.0%**	**0.26 [-0.68 , 1.20]**
95% prediction interval								[-2.29 , 2.81]

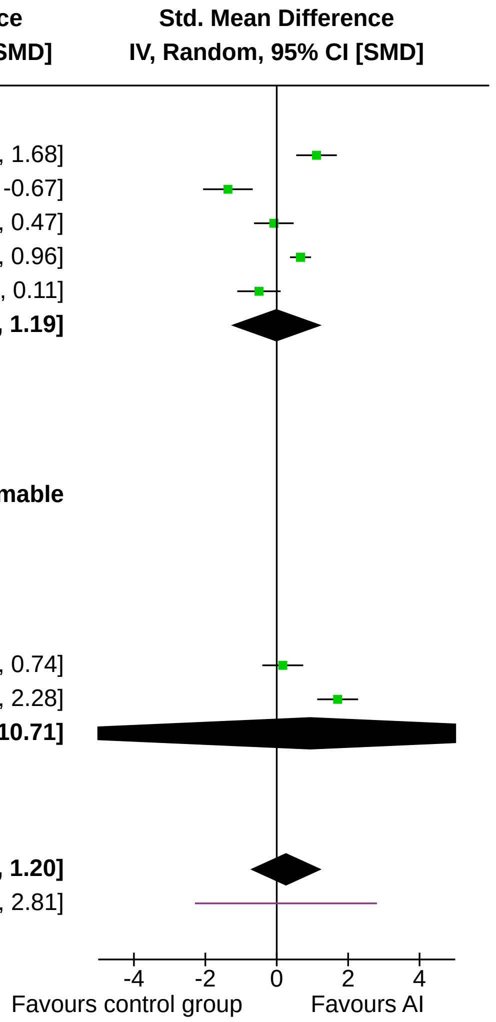

Test for overall effect: T = 0.67, df = 6 (P = 0.53)
Test for subgroup differences: Chi² = 1.15, df = 1 (P = 0.28), I² = 13.4%
Heterogeneity: Tau² (REML [b]) = 0.94; Chi² = 65.75, df = 6 (P < 0.00001); I² = 93%

Footnotes

[a] CI calculated by Hartung-Knapp-Sidik-Jonkman (HKSJ) method.
[b] Tau² calculated by Restricted Maximum-Likelihood method.

Analysis 1.64: Kirkpatrick level 2: practical skills (subgroup: single vs multiple sessions)

Study or Subgroup	AI Mean [SMD]	AI SD [SMD]	AI Total	Control group Mean [SMD]	Control group SD [SMD]	Control group Total	Weight	Std. Mean Difference IV, Random, 95% CI [SMD]
**1.64.1 Single session**								
Chang 2024	0.94	0.06	20	1	0.01	20	16.0%	-1.37 [-2.06 , -0.67]
Fazlollahi 2022	4.63	1.39	23	4.4	1.29	24	16.6%	0.17 [-0.40 , 0.74]
Lau 2025	13.24	3.38	21	14.89	3.13	22	16.4%	-0.50 [-1.11 , 0.11]
Yilmaz 2024	4.3	0.11	33	4.11	0.11	32	16.6%	1.71 [1.13 , 2.28]
**Subtotal (HKSJ [a])**			**97**			**98**	**65.7%**	**0.01 [-2.05 , 2.08]**
Test for overall effect: T = 0.02, df = 3 (P = 0.99)								
Heterogeneity: Tau² (REML [b]) = 1.58; Chi² = 51.01, df = 3 (P < 0.00001); I² = 94%								
**1.64.2 Multiple sessions**								
Höhne 2025	8.98	1.42	25	9.09	1.27	25	16.7%	-0.08 [-0.63 , 0.47]
Huang 2025	73.12	10.06	94	65.54	12.48	93	17.7%	0.67 [0.37 , 0.96]
**Subtotal (HKSJ [a])**			**119**			**118**	**34.3%**	**0.33 [-4.39 , 5.05]**
Test for overall effect: T = 0.89, df = 1 (P = 0.54)								
Heterogeneity: Tau² (REML [b]) = 0.23; Chi² = 5.43, df = 1 (P = 0.02); I² = 82%								
**Total (HKSJ [a])**			**216**			**216**	**100.0%**	**0.12 [-0.97 , 1.20]**
95% prediction interval								[-2.65 , 2.88]

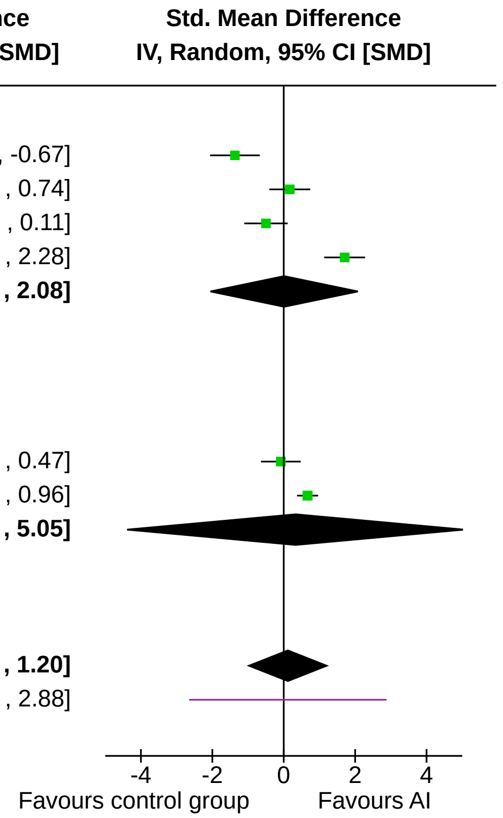

Test for overall effect: T = 0.27, df = 5 (P = 0.80)
Test for subgroup differences: Chi² = 0.18, df = 1 (P = 0.67), I² = 0%
Heterogeneity: Tau² (REML [b]) = 0.98; Chi² = 59.56, df = 5 (P < 0.00001); I² = 93%

Footnotes
[a] CI calculated by Hartung-Knapp-Sidik-Jonkman (HKSJ) method.
[b] Tau² calculated by Restricted Maximum-Likelihood method.

Analysis 1.65: Kirkpatrick level 2: task efficiency (LLM personalised learning aid vs control)

Study or Subgroup	LLM personalised learning aid Mean [SMD]	LLM personalised learning aid SD [SMD]	LLM personalised learning aid Total	Control group Mean [SMD]	Control group SD [SMD]	Control group Total	Weight	Std. Mean Difference IV, Random, 95% CI [SMD]
Castano-Villegas 2025	4.35	6.53	40	8.11	12.15	39	65.4%	-0.38 [-0.83 , 0.06]
Kalam 2025	140	64	10	121.55	56.41	11	34.6%	0.29 [-0.57 , 1.16]
**Total (HKSJ [a])**			**50**			**50**	**100.0%**	**-0.15 [-4.24 , 3.95]**
95% prediction interval								[-5.99 , 5.69]

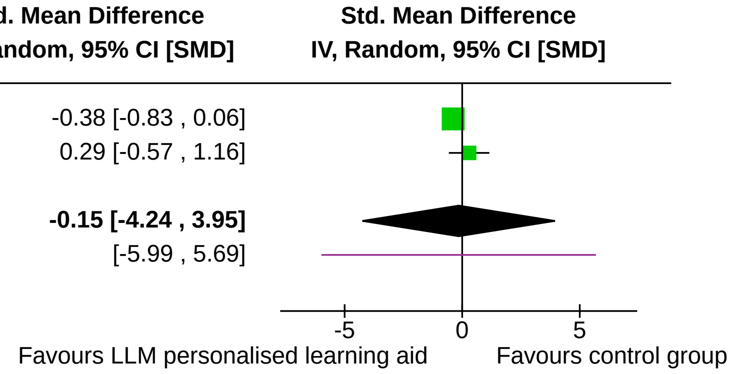

Test for overall effect: T = 0.46, df = 1 (P = 0.72)

Heterogeneity: Tau² (REML [b]) = 0.11; Chi² = 1.87, df = 1 (P = 0.17); I² = 47%

Footnotes
[a] CI calculated by Hartung-Knapp-Sidik-Jonkman (HKSJ) method.
[b] Tau² calculated by Restricted Maximum-Likelihood method.

Analysis 1.66: Kirkpatrick level 2: task efficiency (non-LLM AI imaging diagnostic aid vs control)

Study or Subgroup	AI imaging diagnostic aid (non LLM) Mean [SMD]	SD [SMD]	Total	Control group Mean [SMD]	SD [SMD]	Total	Weight	Std. Mean Difference IV, Random, 95% CI [SMD]
Ayan 2024	14.68	3.06	20	7.32	2.22	20	100.0%	2.70 [1.82 , 3.58]
**Total**			**20**			**20**	**100.0%**	**2.70 [1.82 , 3.58]**
95% prediction interval								[1.82 , 3.58]

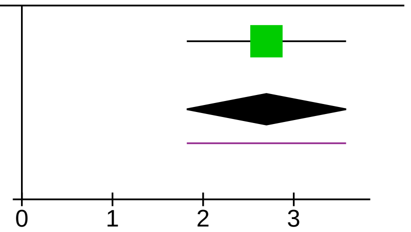

Test for overall effect: Z = 6.02 (P < 0.00001)

Heterogeneity: Not applicable

Analysis 1.67: Kirkpatrick level 2: task efficiency (non-LLM AI procedure assistant vs control)

Study or Subgroup	AI procedure assistant (non-LLM) Mean [SMD]	SD [SMD]	Total	Control group Mean [SMD]	SD [SMD]	Total	Weight	Std. Mean Difference IV, Random, 95% CI [SMD]
Chang 2024	45.99	41.38	20	158.01	54.45	20	52.3%	-2.27 [-3.08 , -1.46]
Lau 2025	114	46.6	4	135	140	8	47.7%	-0.16 [-1.36 , 1.04]
**Total (HKSJ [a])**			**24**			**28**	**100.0%**	**-1.26 [-14.65 , 12.12]**
95% prediction interval								[-23.49 , 20.96]

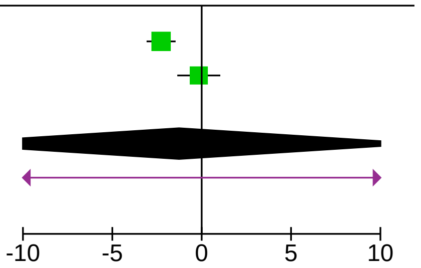

Test for overall effect: T = 1.20, df = 1 (P = 0.44)

Heterogeneity: Tau² (REML [b]) = 1.95; Chi² = 8.11, df = 1 (P = 0.004); I² = 88%

Footnotes

[a] CI calculated by Hartung-Knapp-Sidik-Jonkman (HKSJ) method.

[b] Tau² calculated by Restricted Maximum-Likelihood method.

Analysis 1.68: Kirkpatrick level 2: task efficiency (non-LLM AI procedure assistant + AI-moderated adaptive learning platform vs control)

Study or Subgroup	AI procedure assistant + AI-moderated adaptive learning platform Mean [SMD]	SD [SMD]	Total	Control group Mean [SMD]	SD [SMD]	Total	Weight	Std. Mean Difference IV, Random, 95% CI [SMD]
Usta 2025	18.49	2.8	10	21.44	3.62	10	100.0%	-0.87 [-1.80 , 0.05]
**Total**			**10**			**10**	**100.0%**	**-0.87 [-1.80 , 0.05]**
95% prediction interval								[-1.80 , 0.05]

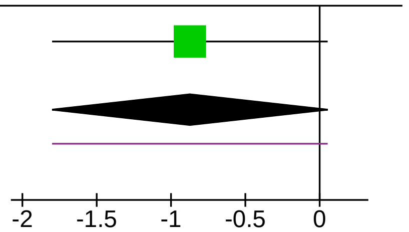

Test for overall effect: Z = 1.85 (P = 0.06)

Heterogeneity: Not applicable

Analysis 1.69: Kirkpatrick level 2: task efficiency (subgroup: field of study)

Study or Subgroup	AI Mean [SMD]	AI SD [SMD]	AI Total	Control group Mean [SMD]	Control group SD [SMD]	Control group Total	Weight	Std. Mean Difference IV, Random, 95% CI [SMD]
**1.69.1 Medicine**								
Castano-Villegas 2025	4.35	6.53	40	8.11	12.15	39	17.6%	-0.38 [-0.83 , 0.06]
Kalam 2025	140	64	10	121.55	56.41	11	16.7%	0.29 [-0.57 , 1.16]
Lau 2025	114	46.6	4	135	140	8	15.6%	-0.16 [-1.36 , 1.04]
**Subtotal (HKSJ [a])**			**54**			**58**	**50.0%**	**-0.20 [-1.05 , 0.66]**
Test for overall effect: T = 0.99, df = 2 (P = 0.43)								
Heterogeneity: Tau² (REML [b]) = 0.03; Chi² = 1.89, df = 2 (P = 0.39); I² = 16%								
**1.69.2 Nursing**								
**Subtotal**			**0**			**0**		**Not estimable**
Test for overall effect: Not applicable								
Heterogeneity: Not applicable								
**1.69.3 Dentistry**								
Ayan 2024	14.68	3.06	20	7.32	2.22	20	16.7%	2.70 [1.82 , 3.58]
Chang 2024	45.99	41.38	20	158.01	54.45	20	16.8%	-2.27 [-3.08 , -1.46]
Usta 2025	18.49	2.8	10	21.44	3.62	10	16.5%	-0.87 [-1.80 , 0.05]
**Subtotal (HKSJ [a])**			**50**			**50**	**50.0%**	**-0.15 [-6.52 , 6.22]**
Test for overall effect: T = 0.10, df = 2 (P = 0.93)								
Heterogeneity: Tau² (REML [b]) = 6.38; Chi² = 68.80, df = 2 (P < 0.00001); I² = 97%								
**1.69.4 Pharmacy**								
**Subtotal**			**0**			**0**		**Not estimable**
Test for overall effect: Not applicable								
Heterogeneity: Not applicable								
**1.69.5 Physiotherapy**								
**Subtotal**			**0**			**0**		**Not estimable**
Test for overall effect: Not applicable								
Heterogeneity: Not applicable								
**1.69.6 Health Sciences**								
**Subtotal**			**0**			**0**		**Not estimable**
Test for overall effect: Not applicable								
Heterogeneity: Not applicable								
**1.69.7 Optometry**								
**Subtotal**			**0**			**0**		**Not estimable**
Test for overall effect: Not applicable								
Heterogeneity: Not applicable								
**1.69.8 Others**								
**Subtotal**			**0**			**0**		**Not estimable**
Test for overall effect: Not applicable								
Heterogeneity: Not applicable								
**Total (HKSJ [a])**			**104**			**108**	**100.0%**	**-0.12 [-1.84 , 1.60]**
95% prediction interval								[-4.53 , 4.29]

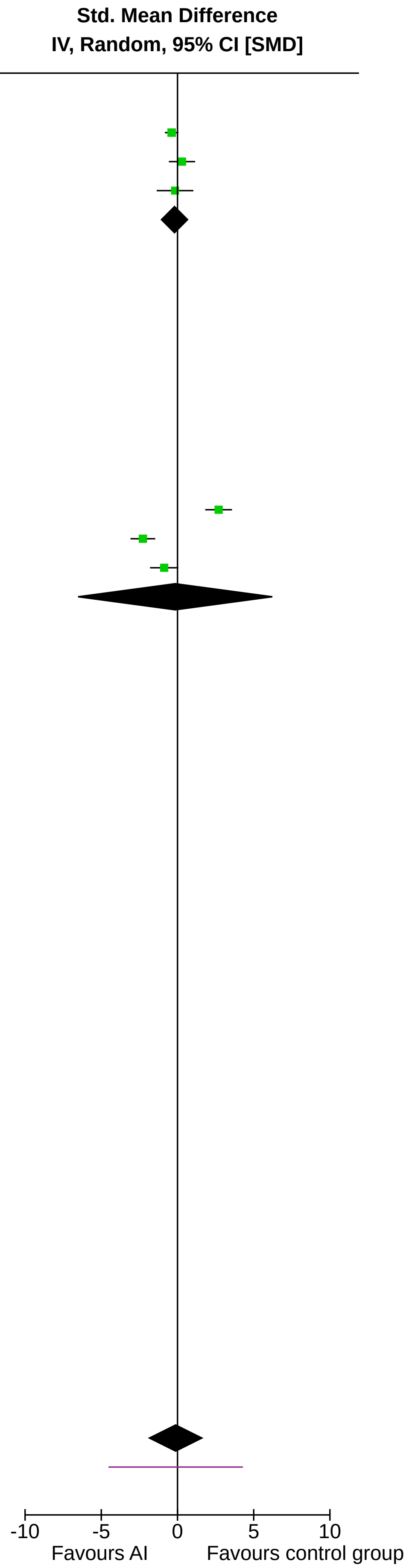

Test for overall effect: T = 0.18, df = 5 (P = 0.86)
Test for subgroup differences: Chi² = 0.00, df = 1 (P = 0.98), I² = 0%
Heterogeneity: Tau² (REML [b]) = 2.49; Chi² = 70.69, df = 5 (P < 0.00001); I² = 94%

Footnotes

[a] CI calculated by Hartung-Knapp-Sidik-Jonkman (HKSJ) method.
[b] Tau² calculated by Restricted Maximum-Likelihood method.

Analysis 1.70: Kirkpatrick level 2: task efficiency (subgroup: region of study)

Study or Subgroup	AI Mean [SMD]	AI SD [SMD]	AI Total	Control group Mean [SMD]	Control group SD [SMD]	Control group Total	Weight	Std. Mean Difference IV, Random, 95% CI [SMD]
**1.70.1 Asia**								
Lau 2025	114	46.6	4	135	140	8	15.6%	-0.16 [-1.36 , 1.04]
**Subtotal**			**4**			**8**	**15.6%**	**-0.16 [-1.36 , 1.04]**
Test for overall effect: Z = 0.26 (P = 0.79)								
Heterogeneity: Not applicable								
**1.70.2 North America**								
Chang 2024	45.99	41.38	20	158.01	54.45	20	16.8%	-2.27 [-3.08 , -1.46]
Kalam 2025	140	64	10	121.55	56.41	11	16.7%	0.29 [-0.57 , 1.16]
**Subtotal (HKSJ [a])**			**30**			**31**	**33.5%**	**-0.99 [-17.29 , 15.30]**
Test for overall effect: T = 0.77, df = 1 (P = 0.58)								
Heterogeneity: Tau^2 (REML [b]) = 3.11; Chi^2 = 18.03, df = 1 (P < 0.0001); I^2 = 94%								
**1.70.3 South America**								
Castano-Villegas 2025	4.35	6.53	40	8.11	12.15	39	17.6%	-0.38 [-0.83 , 0.06]
**Subtotal**			**40**			**39**	**17.6%**	**-0.38 [-0.83 , 0.06]**
Test for overall effect: Z = 1.69 (P = 0.09)								
Heterogeneity: Not applicable								
**1.70.4 Central America**								
**Subtotal**			**0**			**0**		**Not estimable**
Test for overall effect: Not applicable								
Heterogeneity: Not applicable								
**1.70.5 Africa**								
**Subtotal**			**0**			**0**		**Not estimable**
Test for overall effect: Not applicable								
Heterogeneity: Not applicable								
**1.70.6 Europe**								
Ayan 2024	14.68	3.06	20	7.32	2.22	20	16.7%	2.70 [1.82 , 3.58]
Usta 2025	18.49	2.8	10	21.44	3.62	10	16.5%	-0.87 [-1.80 , 0.05]
**Subtotal (HKSJ [a])**			**30**			**30**	**33.2%**	**0.92 [-21.77 , 23.61]**
Test for overall effect: T = 0.51, df = 1 (P = 0.70)								
Heterogeneity: Tau^2 (REML [b]) = 6.17; Chi^2 = 30.04, df = 1 (P < 0.00001); I^2 = 97%								
**1.70.7 Oceania**								
**Subtotal**			**0**			**0**		**Not estimable**
Test for overall effect: Not applicable								
Heterogeneity: Not applicable								
**1.70.8 Multi-continents**								
**Subtotal**			**0**			**0**		**Not estimable**
Test for overall effect: Not applicable								
Heterogeneity: Not applicable								
**Total (HKSJ [a])**			**104**			**108**	**100.0%**	**-0.12 [-1.84 , 1.60]**
95% prediction interval								[-4.53 , 4.29]

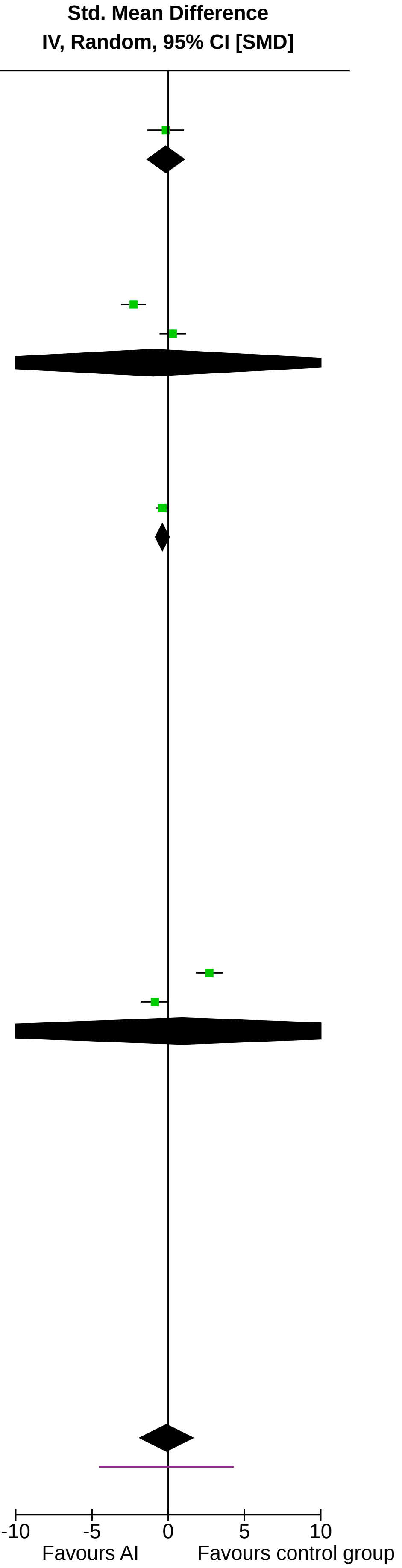

Test for overall effect: T = 0.18, df = 5 (P = 0.86)
Test for subgroup differences: Chi^2 = 0.87, df = 3 (P = 0.83), I^2 = 0%
Heterogeneity: Tau^2 (REML [b]) = 2.49; Chi^2 = 70.69, df = 5 (P < 0.00001); I^2 = 94%

Footnotes

[a] CI calculated by Hartung-Knapp-Sidik-Jonkman (HKSJ) method.

[b] Tau^2 calculated by Restricted Maximum-Likelihood method.

Analysis 1.71: Kirkpatrick level 2: task efficiency (subgroup: LLM vs non-LLM)

Study or Subgroup	AI Mean [SMD]	AI SD [SMD]	AI Total	Control group Mean [SMD]	Control group SD [SMD]	Control group Total	Weight	Std. Mean Difference IV, Random, 95% CI [SMD]
**1.71.1 LLM**								
Castano-Villegas 2025	4.35	6.53	40	8.11	12.15	39	17.6%	-0.38 [-0.83 , 0.06]
Kalam 2025	140	64	10	121.55	56.41	11	16.7%	0.29 [-0.57 , 1.16]
**Subtotal (HKSJ [a])**			**50**			**50**	**34.3%**	**-0.15 [-4.24 , 3.95]**
Test for overall effect: T = 0.46, df = 1 (P = 0.72)								
Heterogeneity: Tau² (REML [b]) = 0.11; Chi² = 1.87, df = 1 (P = 0.17); I² = 47%								
**1.71.2 Non-LLM**								
Ayan 2024	14.68	3.06	20	7.32	2.22	20	16.7%	2.70 [1.82 , 3.58]
Chang 2024	45.99	41.38	20	158.01	54.45	20	16.8%	-2.27 [-3.08 , -1.46]
Lau 2025	114	46.6	4	135	140	8	15.6%	-0.16 [-1.36 , 1.04]
Usta 2025	18.49	2.8	10	21.44	3.62	10	16.5%	-0.87 [-1.80 , 0.05]
**Subtotal (HKSJ [a])**			**54**			**58**	**65.7%**	**-0.15 [-3.50 , 3.19]**
Test for overall effect: T = 0.15, df = 3 (P = 0.89)								
Heterogeneity: Tau² (REML [b]) = 4.24; Chi² = 68.81, df = 3 (P < 0.00001); I² = 95%								
**Total (HKSJ [a])**			**104**			**108**	**100.0%**	**-0.12 [-1.84 , 1.60]**
95% prediction interval								[-4.53 , 4.29]

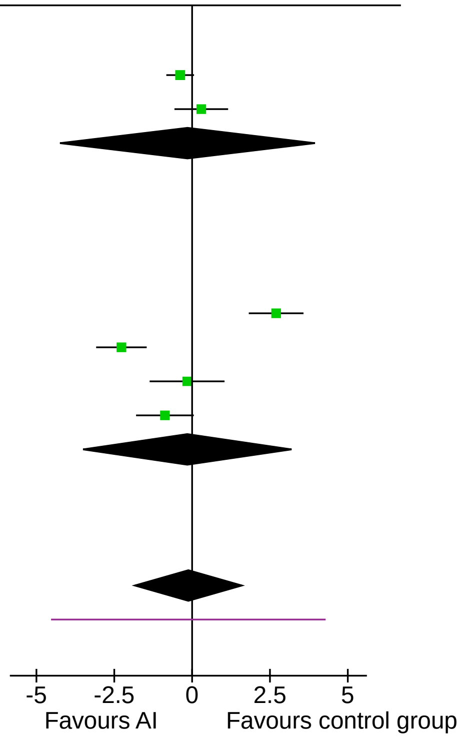

Test for overall effect: T = 0.18, df = 5 (P = 0.86)
Test for subgroup differences: Chi² = 0.00, df = 1 (P = 1.00), I² = 0%
Heterogeneity: Tau² (REML [b]) = 2.49; Chi² = 70.69, df = 5 (P < 0.00001); I² = 94%

Footnotes

[a] CI calculated by Hartung-Knapp-Sidik-Jonkman (HKSJ) method.
[b] Tau² calculated by Restricted Maximum-Likelihood method.

Analysis 1.72: Kirkpatrick level 2: task efficiency (subgroup: main function of application - teaching learning vs assessment)

	AI			Control group				Std. Mean Difference
**Study or Subgroup**	**Mean [SMD]**	**SD [SMD]**	**Total**	**Mean [SMD]**	**SD [SMD]**	**Total**	**Weight**	**IV, Random, 95% CI [SMD]**
**1.72.1 Teaching and learning**								
Castano-Villegas 2025	4.35	6.53	40	8.11	12.15	39	17.6%	-0.38 [-0.83 , 0.06]
Chang 2024	45.99	41.38	20	158.01	54.45	20	16.8%	-2.27 [-3.08 , -1.46]
Lau 2025	114	46.6	4	135	140	8	15.6%	-0.16 [-1.36 , 1.04]
Usta 2025	18.49	2.8	10	21.44	3.62	10	16.5%	-0.87 [-1.80 , 0.05]
**Subtotal (HKSJ [a])**			**74**			**77**	**66.6%**	**-0.94 [-2.44 , 0.57]**
Test for overall effect: T = 1.98, df = 3 (P = 0.14)								
Heterogeneity: Tau² (REML [b]) = 0.72; Chi² = 17.06, df = 3 (P = 0.0007); I² = 82%								
**1.72.2 Assessment**								
**Subtotal**			**0**			**0**		**Not estimable**
Test for overall effect: Not applicable								
Heterogeneity: Not applicable								
**1.72.3 Teaching-learning and assessment**								
Ayan 2024	14.68	3.06	20	7.32	2.22	20	16.7%	2.70 [1.82 , 3.58]
Kalam 2025	140	64	10	121.55	56.41	11	16.7%	0.29 [-0.57 , 1.16]
**Subtotal (HKSJ [a])**			**30**			**31**	**33.4%**	**1.50 [-13.78 , 16.77]**
Test for overall effect: T = 1.24, df = 1 (P = 0.43)								
Heterogeneity: Tau² (REML [b]) = 2.69; Chi² = 14.65, df = 1 (P = 0.0001); I² = 93%								
**Total (HKSJ [a])**			**104**			**108**	**100.0%**	**-0.12 [-1.84 , 1.60]**
95% prediction interval								[-4.53 , 4.29]

Test for overall effect: T = 0.18, df = 5 (P = 0.86)
Test for subgroup differences: Chi² = 3.55, df = 1 (P = 0.06), I² = 71.8%
Heterogeneity: Tau² (REML [b]) = 2.49; Chi² = 70.69, df = 5 (P < 0.00001); I² = 94%

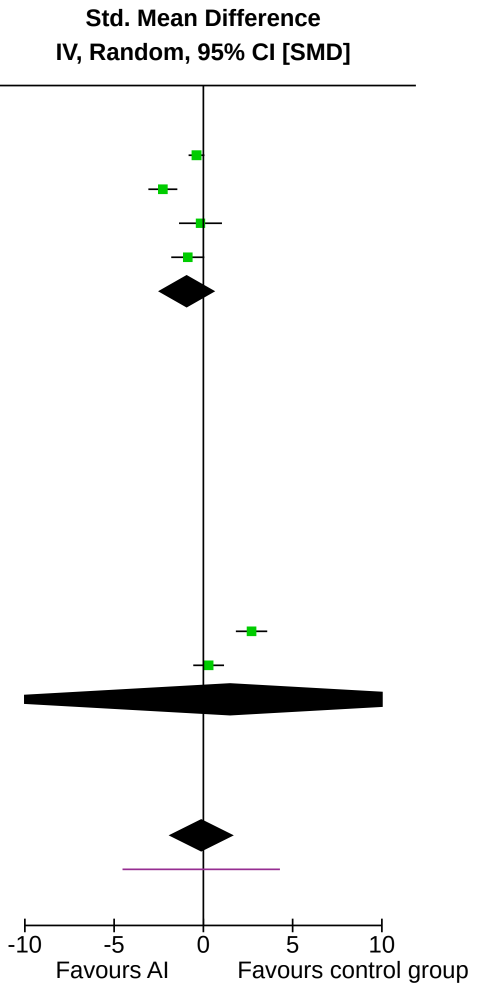

Footnotes

[a] CI calculated by Hartung-Knapp-Sidik-Jonkman (HKSJ) method.
[b] Tau² calculated by Restricted Maximum-Likelihood method.

Analysis 1.73: Kirkpatrick level 2: task efficiency (subgroup: single vs multiple sessions)

	AI			Control group				Std. Mean Difference
Study or Subgroup	Mean [SMD]	SD [SMD]	Total	Mean [SMD]	SD [SMD]	Total	Weight	IV, Random, 95% CI [SMD]
**1.73.1 Single session**								
Ayan 2024	14.68	3.06	20	7.32	2.22	20	16.7%	2.70 [1.82 , 3.58]
Castano-Villegas 2025	4.35	6.53	40	8.11	12.15	39	17.6%	-0.38 [-0.83 , 0.06]
Chang 2024	45.99	41.38	20	158.01	54.45	20	16.8%	-2.27 [-3.08 , -1.46]
Lau 2025	114	46.6	4	135	140	8	15.6%	-0.16 [-1.36 , 1.04]
**Subtotal (HKSJ [a])**			**84**			**87**	**66.8%**	**-0.04 [-3.30 , 3.23]**
Test for overall effect: T = 0.03, df = 3 (P = 0.97)								
Heterogeneity: Tau² (REML [b]) = 4.02; Chi² = 67.42, df = 3 (P < 0.00001); I² = 96%								
**1.73.2 Multiple sessions**								
Kalam 2025	140	64	10	121.55	56.41	11	16.7%	0.29 [-0.57 , 1.16]
Usta 2025	18.49	2.8	10	21.44	3.62	10	16.5%	-0.87 [-1.80 , 0.05]
**Subtotal (HKSJ [a])**			**20**			**21**	**33.2%**	**-0.28 [-7.69 , 7.14]**
Test for overall effect: T = 0.47, df = 1 (P = 0.72)								
Heterogeneity: Tau² (REML [b]) = 0.47; Chi² = 3.27, df = 1 (P = 0.07); I² = 69%								
**Total (HKSJ [a])**			**104**			**108**	**100.0%**	**-0.12 [-1.84 , 1.60]**
95% prediction interval								[-4.53 , 4.29]

Std. Mean Difference IV, Random, 95% CI [SMD]

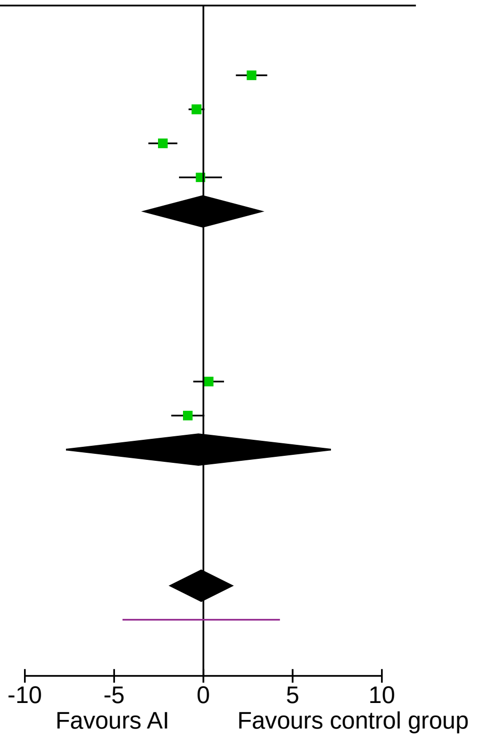

Test for overall effect: T = 0.18, df = 5 (P = 0.86)
Test for subgroup differences: Chi² = 0.04, df = 1 (P = 0.84), I² = 0%
Heterogeneity: Tau² (REML [b]) = 2.49; Chi² = 70.69, df = 5 (P < 0.00001); I² = 94%

Footnotes
[a] CI calculated by Hartung-Knapp-Sidik-Jonkman (HKSJ) method.
[b] Tau² calculated by Restricted Maximum-Likelihood method.

Analysis 1.74: Kirkpatrick level 2: generic or personal skills (LLM personalised learning aid vs control)

	LLM personalised learning aid			Control group				Std. Mean Difference
Study or Subgroup	Mean [SMD]	SD [SMD]	Total	Mean [SMD]	SD [SMD]	Total	Weight	IV, Random, 95% CI [SMD]
Wang 2024	33.5	11.63	50	29	8.15	51	100.0%	0.45 [0.05 , 0.84]
**Total**			**50**			**51**	**100.0%**	**0.45 [0.05 , 0.84]**
95% prediction interval								[0.05 , 0.84]

Std. Mean Difference IV, Random, 95% CI [SMD]

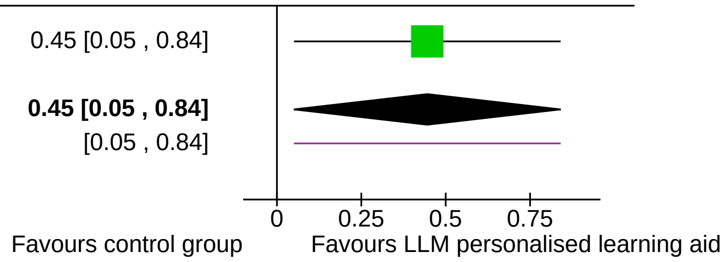

Test for overall effect: Z = 2.21 (P = 0.03)

Heterogeneity: Not applicable

Analysis 1.75: Kirkpatrick level 2: generic or personal skills (LLM virtual patient vs control)

	LLM virtual patient			Control group				Std. Mean Difference
Study or Subgroup	Mean [SMD]	SD [SMD]	Total	Mean [SMD]	SD [SMD]	Total	Weight	IV, Random, 95% CI [SMD]
Ferrer-Peña 2025	7.3	0.89	4	7.3	0.96	23	100.0%	0.00 [-1.06 , 1.06]
**Total**			**4**			**23**	**100.0%**	**0.00 [-1.06 , 1.06]**
95% prediction interval								[-1.06 , 1.06]

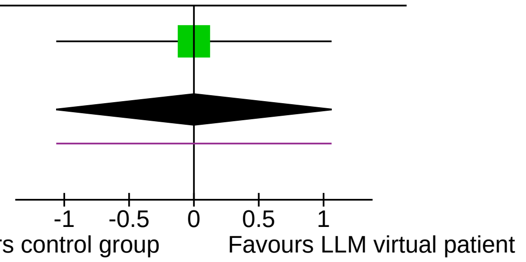

Test for overall effect: Z = 0.00 (P = 1.00)

Heterogeneity: Not applicable

Analysis 1.76: Kirkpatrick level 2: generic or personal skills (LLM-integrated curriculum vs control)

	LLM-integrated curriculum			Control group				Std. Mean Difference
Study or Subgroup	Mean [SMD]	SD [SMD]	Total	Mean [SMD]	SD [SMD]	Total	Weight	IV, Random, 95% CI [SMD]
Arkan 2025	100.5	12.98	48	90.7	18.92	48	100.0%	0.60 [0.19 , 1.01]
**Total**			**48**			**48**	**100.0%**	**0.60 [0.19 , 1.01]**
95% prediction interval								[0.19 , 1.01]

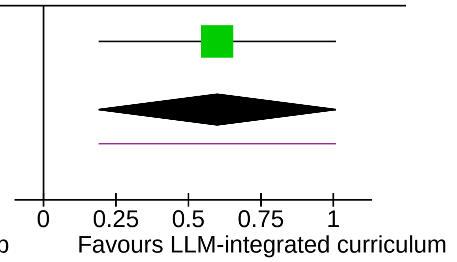

Test for overall effect: Z = 2.87 (P = 0.004)

Heterogeneity: Not applicable

Analysis 1.77: Kirkpatrick level 2: generic or personal skills (non-LLM AI communication analysis vs control)

	AI communication analysis (non-LLM)			Control group				Std. Mean Difference
Study or Subgroup	Mean [SMD]	SD [SMD]	Total	Mean [SMD]	SD [SMD]	Total	Weight	IV, Random, 95% CI [SMD]
Kobayashi 2022	9.1	6.6	13	-1.3	3.8	12	100.0%	1.85 [0.88 , 2.81]
**Total**			**13**			**12**	**100.0%**	**1.85 [0.88 , 2.81]**
95% prediction interval								[0.88 , 2.81]

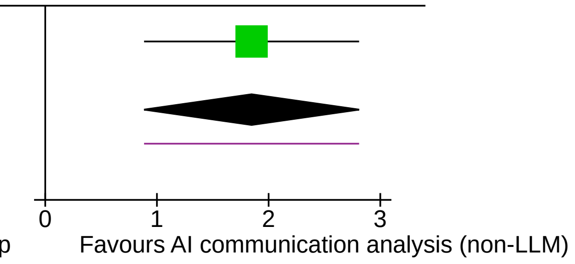

Test for overall effect: Z = 3.76 (P = 0.0002)

Heterogeneity: Not applicable

Analysis 1.78: Kirkpatrick level 2: generic or personal skills (AI-VR virtual doctor vs control)

	AI-VR virtual doctor (non-LLM)			Control group				Std. Mean Difference
Study or Subgroup	Mean [SMD]	SD [SMD]	Total	Mean [SMD]	SD [SMD]	Total	Weight	IV, Random, 95% CI [SMD]
Liaw 2023	29.34	8.37	32	27.06	5.69	32	100.0%	0.31 [-0.18 , 0.81]
**Total**			**32**			**32**	**100.0%**	**0.31 [-0.18 , 0.81]**
95% prediction interval								[-0.18 , 0.81]

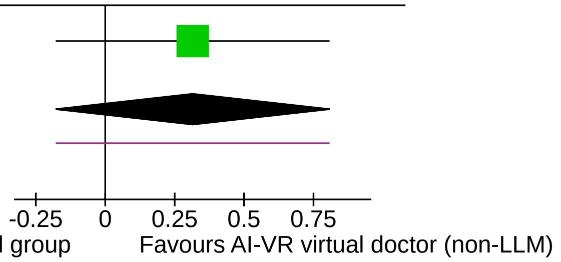

Test for overall effect: Z = 1.25 (P = 0.21)

Heterogeneity: Not applicable

Analysis 1.79: Kirkpatrick level 2: generic or personal skills (subgroup: field of study)

Study or Subgroup	AI Mean [SMD]	AI SD [SMD]	AI Total	Control group Mean [SMD]	Control group SD [SMD]	Control group Total	Weight	Std. Mean Difference IV, Random, 95% CI [SMD]
**1.79.1 Medicine**								
Wang 2024	33.5	11.63	50	29	8.15	51	28.3%	0.45 [0.05 , 0.84]
**Subtotal**			**50**			**51**	**28.3%**	**0.45 [0.05 , 0.84]**
Test for overall effect: Z = 2.21 (P = 0.03)								
Heterogeneity: Not applicable								
**1.79.2 Nursing**								
Arkan 2025	100.5	12.98	48	90.7	18.92	48	27.7%	0.60 [0.19 , 1.01]
Kobayashi 2022	9.1	6.6	13	-1.3	3.8	12	10.8%	1.85 [0.88 , 2.81]
Liaw 2023	29.34	8.37	32	27.06	5.69	32	23.9%	0.31 [-0.18 , 0.81]
**Subtotal (HKSJ [a])**			**93**			**92**	**62.4%**	**0.82 [-1.07 , 2.72]**
Test for overall effect: T = 1.87, df = 2 (P = 0.20)								
Heterogeneity: Tau² (REML [b]) = 0.41; Chi² = 7.73, df = 2 (P = 0.02); I² = 83%								
**1.79.3 Dentistry**								
**Subtotal**			**0**			**0**		**Not estimable**
Test for overall effect: Not applicable								
Heterogeneity: Not applicable								
**1.79.4 Pharmacy**								
**Subtotal**			**0**			**0**		**Not estimable**
Test for overall effect: Not applicable								
Heterogeneity: Not applicable								
**1.79.5 Physiotherapy**								
Ferrer-Peña 2025	7.3	0.89	4	7.3	0.96	23	9.3%	0.00 [-1.06 , 1.06]
**Subtotal**			**4**			**23**	**9.3%**	**0.00 [-1.06 , 1.06]**
Test for overall effect: Z = 0.00 (P = 1.00)								
Heterogeneity: Not applicable								
**1.79.6 Health Sciences**								
**Subtotal**			**0**			**0**		**Not estimable**
Test for overall effect: Not applicable								
Heterogeneity: Not applicable								
**1.79.7 Optometry**								
**Subtotal**			**0**			**0**		**Not estimable**
Test for overall effect: Not applicable								
Heterogeneity: Not applicable								
**1.79.8 Others**								
**Subtotal**			**0**			**0**		**Not estimable**
Test for overall effect: Not applicable								
Heterogeneity: Not applicable								
**Total (HKSJ [a])**			**147**			**166**	**100.0%**	**0.57 [-0.09 , 1.23]**
95% prediction interval								[-0.47 , 1.60]

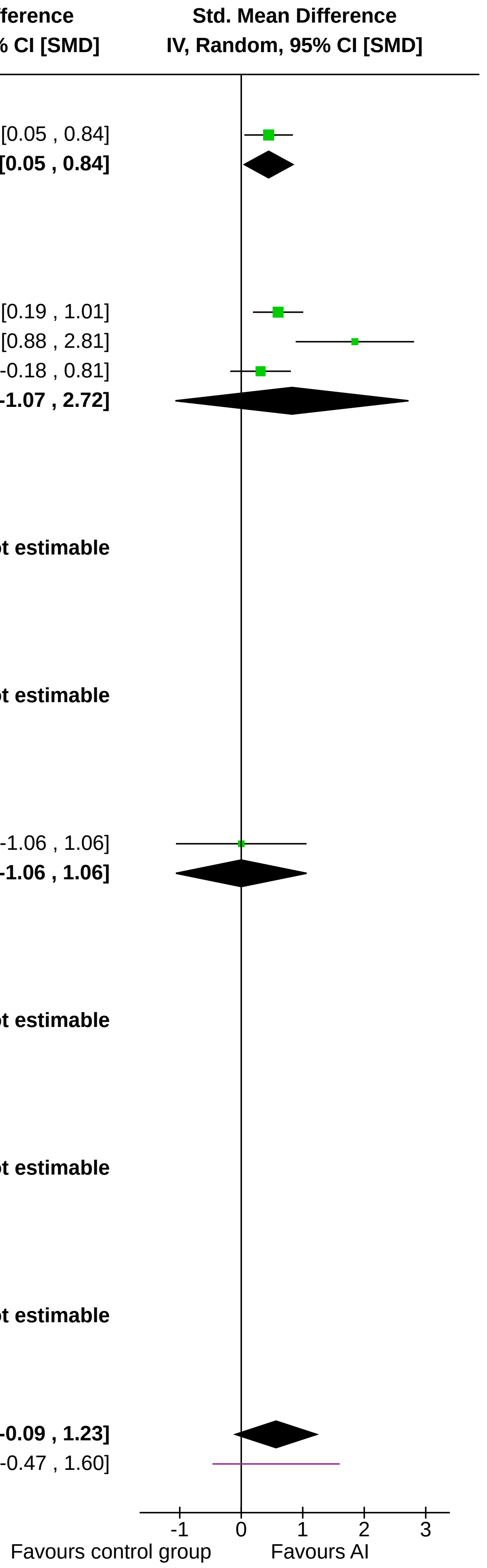

Test for overall effect: T = 2.38, df = 4 (P = 0.08)
Test for subgroup differences: Chi² = 1.41, df = 2 (P = 0.49), I² = 0%
Heterogeneity: Tau² (REML [b]) = 0.08; Chi² = 9.17, df = 4 (P = 0.06); I² = 51%

Footnotes

[a] CI calculated by Hartung-Knapp-Sidik-Jonkman (HKSJ) method.
[b] Tau² calculated by Restricted Maximum-Likelihood method.

Analysis 1.80: Kirkpatrick level 2: generic or personal skills (subgroup: region of study)

	AI			Control group				Std. Mean Difference
**Study or Subgroup**	**Mean [SMD]**	**SD [SMD]**	**Total**	**Mean [SMD]**	**SD [SMD]**	**Total**	**Weight**	**IV, Random, 95% CI [SMD]**
**1.80.1 Asia**								
Kobayashi 2022	9.1	6.6	13	-1.3	3.8	12	10.8%	1.85 [0.88 , 2.81]
Liaw 2023	29.34	8.37	32	27.06	5.69	32	23.9%	0.31 [-0.18 , 0.81]
Wang 2024	33.5	11.63	50	29	8.15	51	28.3%	0.45 [0.05 , 0.84]
**Subtotal (HKSJ [a])**			**95**			**95**	**63.0%**	**0.77 [-1.21 , 2.76]**
Test for overall effect: T = 1.68, df = 2 (P = 0.23)								
Heterogeneity: Tau² (REML [b]) = 0.46; Chi² = 8.10, df = 2 (P = 0.02); I² = 85%								
**1.80.2 North America**								
**Subtotal**			**0**			**0**		**Not estimable**
Test for overall effect: Not applicable								
Heterogeneity: Not applicable								
**1.80.3 South America**								
**Subtotal**			**0**			**0**		**Not estimable**
Test for overall effect: Not applicable								
Heterogeneity: Not applicable								
**1.80.4 Central America**								
**Subtotal**			**0**			**0**		**Not estimable**
Test for overall effect: Not applicable								
Heterogeneity: Not applicable								
**1.80.5 Africa**								
**Subtotal**			**0**			**0**		**Not estimable**
Test for overall effect: Not applicable								
Heterogeneity: Not applicable								
**1.80.6 Europe**								
Arkan 2025	100.5	12.98	48	90.7	18.92	48	27.7%	0.60 [0.19 , 1.01]
Ferrer-Peña 2025	7.3	0.89	4	7.3	0.96	23	9.3%	0.00 [-1.06 , 1.06]
**Subtotal (HKSJ [a])**			**52**			**71**	**37.0%**	**0.51 [-2.23 , 3.24]**
Test for overall effect: T = 2.36, df = 1 (P = 0.26)								
Heterogeneity: Tau² (REML [b]) = 0.01; Chi² = 1.07, df = 1 (P = 0.30); I² = 6%								
**1.80.7 Oceania**								
**Subtotal**			**0**			**0**		**Not estimable**
Test for overall effect: Not applicable								
Heterogeneity: Not applicable								
**1.80.8 Multi-continents**								
**Subtotal**			**0**			**0**		**Not estimable**
Test for overall effect: Not applicable								
Heterogeneity: Not applicable								
**Total (HKSJ [a])**			**147**			**166**	**100.0%**	**0.57 [-0.09 , 1.23]**
95% prediction interval								[-0.47 , 1.60]

Std. Mean Difference
IV, Random, 95% CI [SMD]

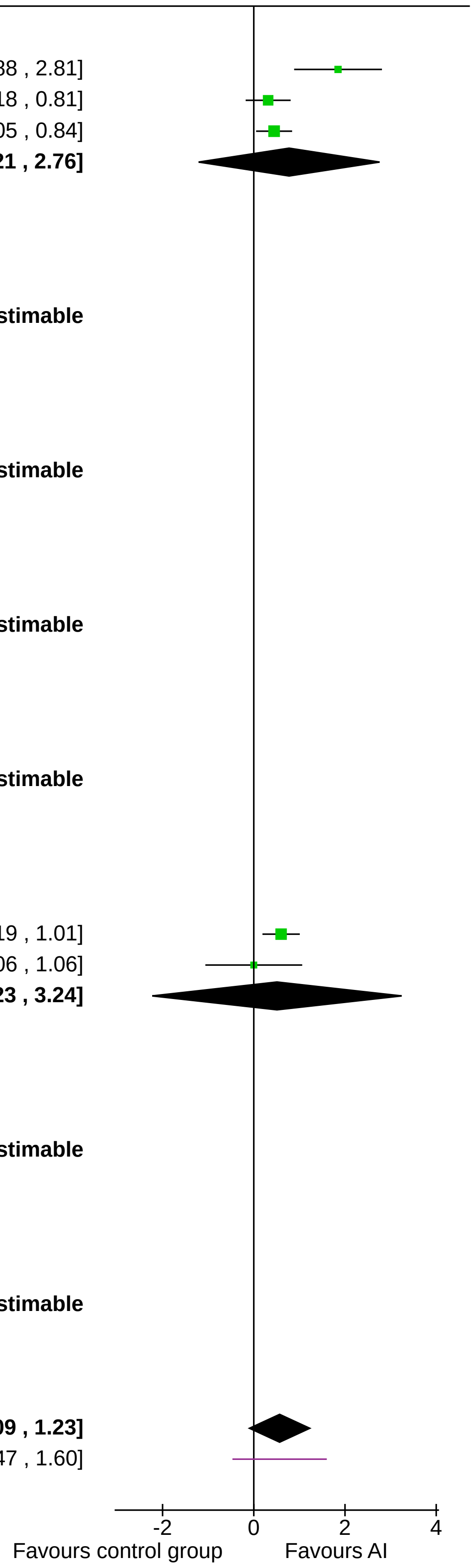

Test for overall effect: T = 2.38, df = 4 (P = 0.08)
Test for subgroup differences: Chi² = 0.27, df = 1 (P = 0.60), I² = 0%
Heterogeneity: Tau² (REML [b]) = 0.08; Chi² = 9.17, df = 4 (P = 0.06); I² = 51%

Footnotes
[a] CI calculated by Hartung-Knapp-Sidik-Jonkman (HKSJ) method.
[b] Tau² calculated by Restricted Maximum-Likelihood method.

Analysis 1.81: Kirkpatrick level 2: generic or personal skills (subgroup: LLM vs non-LLM)

	AI			Control group				Std. Mean Difference
**Study or Subgroup**	**Mean [SMD]**	**SD [SMD]**	**Total**	**Mean [SMD]**	**SD [SMD]**	**Total**	**Weight**	**IV, Random, 95% CI [SMD]**
**1.81.1 LLM**								
Ferrer-Peña 2025	7.3	0.89	4	7.3	0.96	23	9.3%	0.00 [-1.06 , 1.06]
Wang 2024	33.5	11.63	50	29	8.15	51	28.3%	0.45 [0.05 , 0.84]
**Subtotal (HKSJ [a])**			**54**			**74**	**37.6%**	**0.39 [-1.46 , 2.24]**
Test for overall effect: T = 2.69, df = 1 (P = 0.23)								
Heterogeneity: Tau² (REML [b]) = 0.00; Chi² = 0.59, df = 1 (P = 0.44); I² = 0%								
**1.81.2 Non-LLM**								
Arkan 2025	100.5	12.98	48	90.7	18.92	48	27.7%	0.60 [0.19 , 1.01]
Kobayashi 2022	9.1	6.6	13	-1.3	3.8	12	10.8%	1.85 [0.88 , 2.81]
Liaw 2023	29.34	8.37	32	27.06	5.69	32	23.9%	0.31 [-0.18 , 0.81]
**Subtotal (HKSJ [a])**			**93**			**92**	**62.4%**	**0.82 [-1.07 , 2.72]**
Test for overall effect: T = 1.87, df = 2 (P = 0.20)								
Heterogeneity: Tau² (REML [b]) = 0.41; Chi² = 7.73, df = 2 (P = 0.02); I² = 83%								
**Total (HKSJ [a])**			**147**			**166**	**100.0%**	**0.57 [-0.09 , 1.23]**
95% prediction interval								[-0.47 , 1.60]

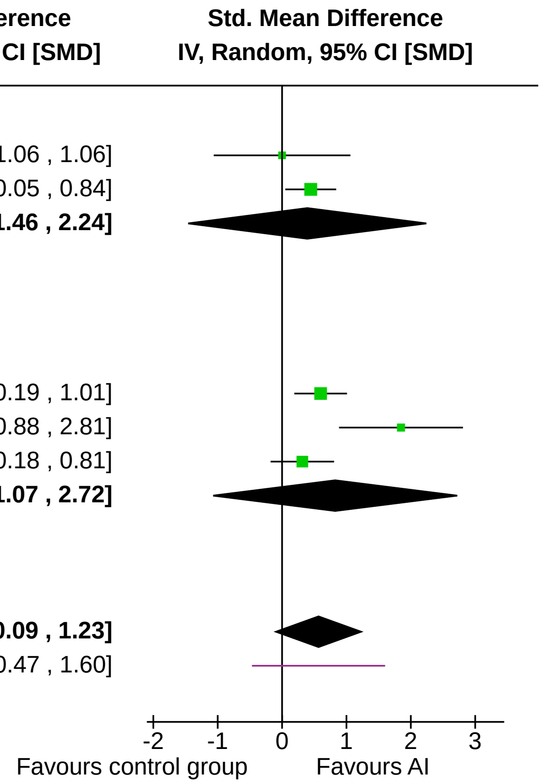

Test for overall effect: T = 2.38, df = 4 (P = 0.08)
Test for subgroup differences: Chi² = 0.87, df = 1 (P = 0.35), I² = 0%
Heterogeneity: Tau² (REML [b]) = 0.08; Chi² = 9.17, df = 4 (P = 0.06); I² = 51%

Footnotes

[a] CI calculated by Hartung-Knapp-Sidik-Jonkman (HKSJ) method.
[b] Tau² calculated by Restricted Maximum-Likelihood method.

Analysis 1.82: Kirkpatrick level 2: generic or personal skills (subgroup: main function of application - teaching learning vs assessment)

	AI			Control group				Std. Mean Difference	Std. Mean Difference
**Study or Subgroup**	**Mean [SMD]**	**SD [SMD]**	**Total**	**Mean [SMD]**	**SD [SMD]**	**Total**	**Weight**	**IV, Random, 95% CI [SMD]**	**IV, Random, 95% CI [SMD]**
**1.82.1 Teaching and learning**									
Arkan 2025	100.5	12.98	48	90.7	18.92	48	27.7%	0.60 [0.19 , 1.01]	
Ferrer-Peña 2025	7.3	0.89	4	7.3	0.96	23	9.3%	0.00 [-1.06 , 1.06]	
Wang 2024	33.5	11.63	50	29	8.15	51	28.3%	0.45 [0.05 , 0.84]	
**Subtotal (HKSJ [a])**			**102**			**122**	**65.3%**	**0.48 [0.03 , 0.94]**	
Test for overall effect: T = 4.59, df = 2 (P = 0.04)									
Heterogeneity: Tau² (REML [b]) = 0.00; Chi² = 1.14, df = 2 (P = 0.57); I² = 0%									
**1.82.2 Assessment**									
Kobayashi 2022	9.1	6.6	13	-1.3	3.8	12	10.8%	1.85 [0.88 , 2.81]	
**Subtotal**			**13**			**12**	**10.8%**	**1.85 [0.88 , 2.81]**	
Test for overall effect: Z = 3.76 (P = 0.0002)									
Heterogeneity: Not applicable									
**1.82.3 Teaching-learning and assessment**									
Liaw 2023	29.34	8.37	32	27.06	5.69	32	23.9%	0.31 [-0.18 , 0.81]	
**Subtotal**			**32**			**32**	**23.9%**	**0.31 [-0.18 , 0.81]**	
Test for overall effect: Z = 1.25 (P = 0.21)									
Heterogeneity: Not applicable									
**Total (HKSJ [a])**			**147**			**166**	**100.0%**	**0.57 [-0.09 , 1.23]**	
95% prediction interval								[-0.47 , 1.60]	

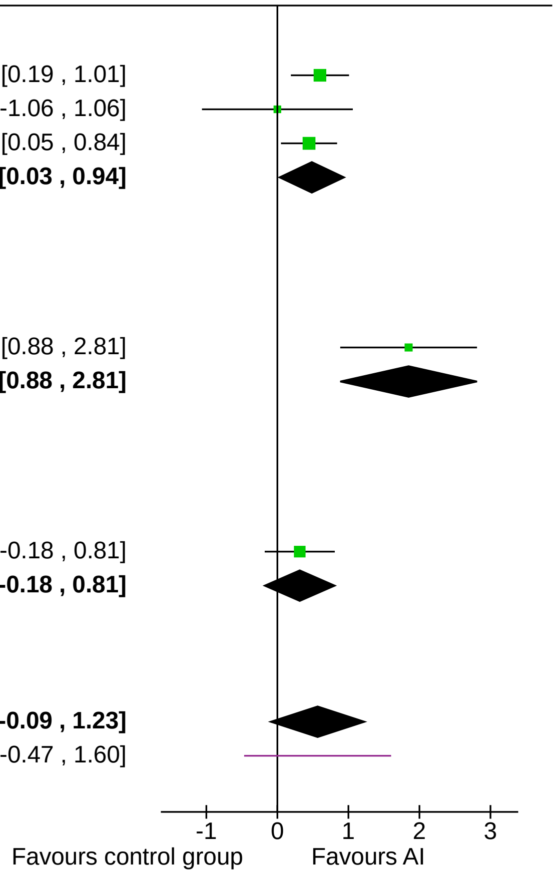

Test for overall effect: T = 2.38, df = 4 (P = 0.08)
Test for subgroup differences: Chi² = 8.07, df = 2 (P = 0.02), I² = 75.2%
Heterogeneity: Tau² (REML [b]) = 0.08; Chi² = 9.17, df = 4 (P = 0.06); I² = 51%

Footnotes

[a] CI calculated by Hartung-Knapp-Sidik-Jonkman (HKSJ) method.

[b] Tau² calculated by Restricted Maximum-Likelihood method.

Analysis 1.83: Kirkpatrick level 2: generic or personal skills (subgroup: single vs multiple sessions)

Study or Subgroup	AI Mean [SMD]	AI SD [SMD]	AI Total	Control group Mean [SMD]	Control group SD [SMD]	Control group Total	Weight	Std. Mean Difference IV, Random, 95% CI [SMD]
**1.83.1 Single session**								
Kobayashi 2022	9.1	6.6	13	-1.3	3.8	12	10.8%	1.85 [0.88 , 2.81]
**Subtotal**			**13**			**12**	**10.8%**	**1.85 [0.88 , 2.81]**
Test for overall effect: Z = 3.76 (P = 0.0002)								
Heterogeneity: Not applicable								
**1.83.2 Multiple sessions**								
Arkan 2025	100.5	12.98	48	90.7	18.92	48	27.7%	0.60 [0.19 , 1.01]
Ferrer-Peña 2025	7.3	0.89	4	7.3	0.96	23	9.3%	0.00 [-1.06 , 1.06]
Liaw 2023	29.34	8.37	32	27.06	5.69	32	23.9%	0.31 [-0.18 , 0.81]
Wang 2024	33.5	11.63	50	29	8.15	51	28.3%	0.45 [0.05 , 0.84]
**Subtotal (HKSJ [a])**			**134**			**154**	**89.2%**	**0.44 [0.17 , 0.72]**
Test for overall effect: T = 5.16, df = 3 (P = 0.01)								
Heterogeneity: Tau² (REML [b]) = 0.00; Chi² = 1.49, df = 3 (P = 0.69); I² = 0%								
**Total (HKSJ [a])**			**147**			**166**	**100.0%**	**0.57 [-0.09 , 1.23]**
95% prediction interval								[-0.47 , 1.60]

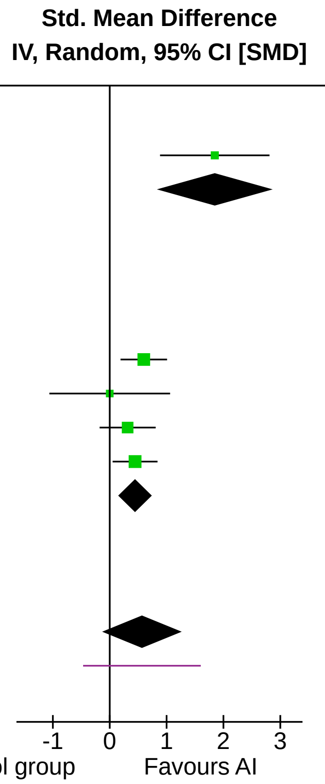

Test for overall effect: T = 2.38, df = 4 (P = 0.08)
Test for subgroup differences: Chi² = 7.91, df = 1 (P = 0.005), I² = 87.4%
Heterogeneity: Tau² (REML [b]) = 0.08; Chi² = 9.17, df = 4 (P = 0.06); I² = 51%

Footnotes

[a] CI calculated by Hartung-Knapp-Sidik-Jonkman (HKSJ) method.

[b] Tau² calculated by Restricted Maximum-Likelihood method.
