# Supplementary material for: The Effectiveness of Artificial Intelligence in Undergraduate Health Professions Education: Systematic Review and Meta-Analysis of Randomized Controlled Trials
Source: JMIR Med Educ. 2026 May 5;12:e88933. doi: 10.2196/88933 (PMC13151782; doi:10.2196/88933)
Supplement: Checklist 1 [file mededu-v12-e88933-s003.docx]

**Abstract**

| **Checklist item** |
| --- |
| **TITLE** |
| Identify the report as a systematic review. ✓ |
| **BACKGROUND** |
| Provide an explicit statement of the main objective(s) or question(s) the review addresses. ✓ |
| **METHODS** |
| Specify the inclusion and exclusion criteria for the review. ✓ |
| Specify the information sources (e.g. databases, registers) used to identify studies and the date when each was last searched. ✓ |
| Specify the methods used to assess risk of bias in the included studies. ✓ |
| Specify the methods used to present and synthesise results. ✓ |
| **RESULTS** |
| Give the total number of included studies and participants and summarise relevant characteristics of studies. ✓ |
| Present results for main outcomes, preferably indicating the number of included studies and participants for each. If meta-analysis was done, report the summary estimate and confidence/credible interval. If comparing groups, indicate the direction of the effect (i.e. which group is favoured). ✓ |
| **DISCUSSION** |
| Provide a brief summary of the limitations of the evidence included in the review (e.g. study risk of bias, inconsistency and imprecision). ✓ |
| Provide a general interpretation of the results and important implications. ✓ |
| **OTHER** |
| Specify the primary source of funding for the review. ✓ |
| Provide the register name and registration number. ✓ |

**Main paper**

| **Section and Topic** | **Item #** | **Checklist item** | **Location where item**  **is reported** |
| --- | --- | --- | --- |
| **TITLE** | | |  |
| Title | 1 | Identify the report as a systematic review. | Title |
| **ABSTRACT** | | |  |
| Abstract | 2 | See the PRISMA 2020 for Abstracts checklist. | Abstract (see PRISMA abstract checklist above) |
| **INTRODUCTION** | | |  |
| Rationale | 3 | Describe the rationale for the review in the context of existing knowledge. | Introduction, paragraphs 1-4 (specifically addressed in paragraph 4) |
| Objectives | 4 | Provide an explicit statement of the objective(s) or question(s) the review addresses. | Introduction, paragraph 4, lines 13-15. |
| **METHODS** | | |  |
| Eligibility criteria | 5 | Specify the inclusion and exclusion criteria for the review and how studies were grouped for the syntheses. | Methods, Inclusion criteria |
| Information sources | 6 | Specify all databases, registers, websites, organisations, reference lists and other sources searched or consulted to identify studies. Specify the date when each source was last searched or consulted. | Methods, Search strategies, Appendix 3: Search strategies |
| Search strategy | 7 | Present the full search strategies for all databases, registers and websites, including any filters and limits used. | Appendix 3 |
| Selection process | 8 | Specify the methods used to decide whether a study met the inclusion criteria of the review, including how many reviewers screened each record and each report retrieved, whether they worked independently, and if applicable, details of automation tools used in the process. | Methods, Screening and selection, data extraction and risk-of-bias assessment, Appendix 2: Data selection, extraction and coding |
| Data collection process | 9 | Specify the methods used to collect data from reports, including how many reviewers collected data from each report, whether they worked independently, any processes for obtaining or confirming data from study investigators, and if applicable, details of automation tools used in the process. | Methods, Screening and selection, data extraction and risk-of-bias assessment, Appendix 2: Data selection, extraction and coding |
| Data items | 10a | List and define all outcomes for which data were sought. Specify whether all results that were compatible with each outcome domain in each study were sought (e.g. for all measures, time points, analyses), and if not, the methods used to decide which results to collect. | Methods, Inclusion criteria, paragraph 3 Outcomes, Appendix 2: Main outcomes |
|  | 10b | List and define all other variables for which data were sought (e.g. participant and intervention characteristics, funding sources). Describe any assumptions made about any missing or unclear information. | Methods, Inclusion criteria, Appendix 2: Inclusion criteria- population, intervention, comparison, Dealing with missing data. |
| Study risk of bias assessment | 11 | Specify the methods used to assess risk of bias in the included studies, including details of the tool(s) used, how many reviewers assessed each study and whether they worked independently, and if applicable, details of automation tools used in the process. | Methods, Screening and selection, data extraction and risk-of-bias assessment, Appendix 2: Risk-of-bias assessment |
| Effect measures | 12 | Specify for each outcome the effect measure(s) (e.g. risk ratio, mean difference) used in the synthesis or presentation of results. | Methods, Data synthesis and certainty-of-evidence rating, Appendix 2: Strategy for data synthesis. |
| Synthesis methods | 13a | Describe the processes used to decide which studies were eligible for each synthesis (e.g. tabulating the study intervention characteristics and comparing against the planned groups for each synthesis (item #5)). | Methods, Data synthesis; Appendix 2: Strategy for data synthesis, paragraph 1. |
|  | 13b | Describe any methods required to prepare the data for presentation or synthesis, such as handling of missing summary statistics, or data conversions. | Methods, Data synthesis; Appendix 2: Strategy for data synthesis, paragraphs 3 and 4. |
|  | 13c | Describe any methods used to tabulate or visually display results of individual studies and syntheses. | Methods, Data synthesis; Appendix 2, Strategy for data synthesis. |
|  | 13d | Describe any methods used to synthesize results and provide a rationale for the choice(s). If meta-analysis was performed, describe the model(s), method(s) to identify the presence and extent of statistical heterogeneity, and software package(s) used. | Methods, Assessment of heterogeneity, data synthesis and certainty-of-evidence rating, Appendix 2: Strategy for dealing with heterogeneity; Strategy for data synthesis |
|  | 13e | Describe any methods used to explore possible causes of heterogeneity among study results (e.g. subgroup analysis, meta-regression). | Methods, Assessment of heterogeneity, data synthesis and certainty-of-evidence rating, Appendix 2: Strategy for dealing with heterogeneity. |
|  | 13f | Describe any sensitivity analyses conducted to assess robustness of the synthesized results. | Appendix 2: Sensitivity analysis. |
| Reporting bias assessment | 14 | Describe any methods used to assess risk of bias due to missing results in a synthesis (arising from reporting biases). | Methods, Assessment of missing data, publication and reporting biases; Appendix 2: Assessment of reporting bias (we included a statement stating that we did not specifically assess risk-of-bias of missing evidence using tools such as RoB-ME). |
| Certainty assessment | 15 | Describe any methods used to assess certainty (or confidence) in the body of evidence for an outcome. | Methods, Certainty-of-evidence rating, Appendix 2: Assessment of the certainty-of-evidence. |

| **RESULTS** | | |  |
| --- | --- | --- | --- |
| Study selection | 16a | Describe the results of the search and selection process, from the number of records identified in the search to the number of studies included in the review, ideally using a flow diagram. | Results: paragraph 1, Figure 1. |
|  | 16b | Cite studies that might appear to meet the inclusion criteria, but which were excluded, and explain why they were excluded. | Results, paragraph 1, Figure 1, Appendix 5. |
| Study characteristics | 17 | Cite each included study and present its characteristics. | Results, Included studies, Appendix 6 (Table 1). |
| Risk of bias in studies | 18 | Present assessments of risk of bias for each included study. | Results, risk-of-bias assessment, Appendix 7 |
| Results of individual studies | 19 | For all outcomes, present, for each study: (a) summary statistics for each group (where appropriate) and (b) an effect estimate and its precision (e.g. confidence/credible interval), ideally using structured tables or plots. | Results, Effect estimates. |
| Results of syntheses | 20a | For each synthesis, briefly summarise the characteristics and risk of bias among contributing studies. | Results, Effect estimates. |
|  | 20b | Present results of all statistical syntheses conducted. If meta-analysis was done, present for each the summary estimate and its precision (e.g. confidence/credible interval) and measures of statistical heterogeneity. If comparing groups, describe the direction of the effect. | Results, Effect estimates. |
|  | 20c | Present results of all investigations of possible causes of heterogeneity among study results. | Results: Effect estimates, |
|  | 20d | Present results of all sensitivity analyses conducted to assess the robustness of the synthesized results. | Methods: Sensitivity analysis (unable to be conducted), Results: NA |
| Reporting biases | 21 | Present assessments of risk of bias due to missing results (arising from reporting biases) for each synthesis assessed. | Methods: Assessment of publication or reporting bias (RoB-ME not used). |
| Certainty of evidence | 22 | Present assessments of certainty (or confidence) in the body of evidence for each outcome assessed. | Results, Effect estimates; Appendix 4. |
| **DISCUSSION** | | |  |
| Discussion | 23a | Provide a general interpretation of the results in the context of other evidence. | Discussion, paragraph 1 |
|  | 23b | Discuss any limitations of the evidence included in the review. | Discussion, paragraph 1 |
|  | 23c | Discuss any limitations of the review processes used. | Discussion, paragraph 3 |
|  | 23d | Discuss implications of the results for practice, policy, and future research. | Conclusions |
| **OTHER INFORMATION** | | |  |
| Registration and protocol | 24a | Provide registration information for the review, including register name and registration number, or state that the review was not registered. | Methods, paragraph 1 |
|  | 24b | Indicate where the review protocol can be accessed, or state that a protocol was not prepared. | Methods, paragraph 1 |
|  | 24c | Describe and explain any amendments to information provided at registration or in the protocol. | Appendix 2 (at the end of each section where applicable, under the heading of ‘Differences between review and protocol”). |
| Support | 25 | Describe sources of financial or non-financial support for the review, and the role of the funders or sponsors in the review. | Acknowledgment |
| Competing interests | 26 | Declare any competing interests of review authors. | Declaration of competing interest |
| Availability of data, code and other materials | 27 | Report which of the following are publicly available and where they can be found: template data collection forms; data extracted from included studies; data used for all analyses; analytic code; any other materials used in the review. | Data sharing statement |

*From:* Page MJ, McKenzie JE, Bossuyt PM, Boutron I, Hoffmann TC, Mulrow CD, et al. The PRISMA 2020 statement: an updated guideline for reporting systematic reviews. BMJ 2021;372:n71. doi: 10.1136/bmj.n71

For more information, visit: <http://www.prisma-statement.org/>
